# Resolving the phylogenetic position of Darwin's extinct ground sloth (*Mylodon darwinii*) using mitogenomic and nuclear exon data

**DOI:** 10.1098/rspb.2018.0214

**Published:** 2018-05-16

**Authors:** Frédéric Delsuc, Melanie Kuch, Gillian C. Gibb, Jonathan Hughes, Paul Szpak, John Southon, Jacob Enk, Ana T. Duggan, Hendrik N. Poinar

**Affiliations:** 1 ISEM, Université de Montpellier, CNRS, IRD, EPHE, Montpellier, France; 2 McMaster Ancient DNA Centre, Department of Anthropology, McMaster University, Hamilton, ON, Canada; 3 Ecology Group, Institute of Agriculture and Environment, Massey University, Palmerston North, New Zealand; 4 Department of Anthropology, Trent University, Peterborough, ON, Canada; 5 Keck Carbon Cycle Accelerator Mass Spectrometer, Earth Systems Science Department, University of California, Irvine, CA, USA; 6 MYcroarray, Ann Arbor, MI, USA

**Keywords:** *Mylodon darwinii*, Xenarthra, ancient DNA, mitochondrial genomes, nuclear data, phylogenetics

## Abstract

*Mylodon darwinii* is the extinct giant ground sloth named after Charles Darwin, who first collected its remains in South America. We have successfully obtained a high-quality mitochondrial genome at 99-fold coverage using an Illumina shotgun sequencing of a 12 880-year-old bone fragment from Mylodon Cave in Chile. Low level of DNA damage showed that this sample was exceptionally well preserved for an ancient subfossil, probably the result of the dry and cold conditions prevailing within the cave. Accordingly, taxonomic assessment of our shotgun metagenomic data showed a very high percentage of endogenous DNA with 22% of the assembled metagenomic contigs assigned to Xenarthra. Additionally, we enriched over 15 kb of sequence data from seven nuclear exons, using target sequence capture designed against a wide xenarthran dataset. Phylogenetic and dating analyses of the mitogenomic dataset including all extant species of xenarthrans and the assembled nuclear supermatrix unambiguously place *Mylodon darwinii* as the sister-group of modern two-fingered sloths, from which it diverged around 22 million years ago. These congruent results from both the mitochondrial and nuclear data support the diphyly of the two modern sloth lineages, implying the convergent evolution of their unique suspensory behaviour as an adaption to arboreality. Our results offer promising perspectives for whole-genome sequencing of this emblematic extinct taxon.

## 1. Background

Darwin's extinct ground sloth (*Mylodon darwinii*) was named by Richard Owen in honour of Charles Darwin who discovered its early remains in South America during the voyage of the *Beagle* [1]. Like the vast majority of the Pleistocene megafauna, *M. darwinii* went extinct at the Pleistocene/Holocene boundary, approximately 10 000 years ago [2]. Numerous subfossils of *M. darwinii* have been found across the South American southern cone [3], including the famous Mylodon Cave (Cueva del Milodón, Ultima Esperanza, Chile). This cave derives its name from the numerous and exquisitely preserved remains of this ground sloth found inside. The constant cold and dry conditions of the cave have enabled the exceptional preservation of *M. darwinii* remains in the form of palaeofaeces, bones, claws and even large pieces of mummified skin covered with blond fur, riddled with osteoderms [4]. These subfossils were the first non-human samples yielding genuine ancient DNA [5]. Short overlapping mitochondrial DNA fragments of 12S and 16S MT-rRNA [5] and MT-CYTB [6] (550–650 base pairs (bp)), have been PCR-amplified, cloned and sequenced from *M. darwinii* bones, and shorter MT-CYTB sequences of 150 bp have even been recovered from hairs embedded in a palaeofaecal sample [7].

Advances in sequencing technology that relies predominantly on short DNA fragments have been a boon for the ancient DNA field, greatly facilitating the assembly of whole mitochondrial genomes in particular [8]. Shotgun Illumina sequencing has been successfully applied to a diversity of Pleistocene subfossil bones containing enough endogenous DNA to reconstruct complete mitogenomes. These studies have helped elucidate the phylogenetic affinities of extinct taxa such as Columbian and woolly mammoths [9], steppe bison [10], giant lemurs [11], and South American equids [12] and camelids [13]. Shotgun sequencing of paleofeces has also proven useful for reconstructing the phylogenetic position of the extinct cave hyena and providing insights into its diet [14]. Recently, Slater *et al*. [15] have reported a partial and composite *M. darwinii* mitogenome reconstructed by mixing reads obtained by DNA target sequence capture from a bone and a paleofeces both sampled at Mylodon Cave. However, apart from endogenous retroviral sequences [15], no phylogenetically informative nuclear DNA has been obtained to date for this extinct taxon.

Previously published mitochondrial data have suggested a close phylogenetic relationship between *Mylodon* and modern two-fingered sloths of the genus *Choloepus* [5,6,15]. Here, we used Illumina shotgun sequencing to obtain a high-quality, ancient mitogenome from *M. darwinii*, significantly improving upon a previously published one [15]. In addition, and importantly, using target sequence capture, we assembled a complementary supermatrix of seven nuclear exons totalling 15 kilobases (kb) for representatives of all xenarthran genera including the extinct *Mylodon* and all modern sloth species. Our refined phylogenetic and dating analyses of congruent mitogenomic and nuclear data encompassing the full diversity of modern xenarthrans corroborated the close relationship of Darwin's ground sloth with two-fingered sloths (*Choloepus*) from which it is estimated to have diverged around 22 Ma.

## 2. Materials and methods

### (a) Sample, DNA extraction, library preparation and shotgun sequencing

The Mylodon bone sample used here stems from the collection the Natural History Museum, London, UK (NHMUK PV M8758), and is a postcranial element from Mylodon Cave (Ultima Esperanza, Chile) originally analysed at the Max Planck Institute for Evolutionary Anthropology (Leipzig, Germany) with laboratory number MPI SP57. Collagen was extracted and purified from a subsample of the specimen at the University of Western Ontario and AMS radiocarbon-dated at the Keck Carbon Cycle AMS facility of the University of California, Irvine (USA) [16].

All manipulations took place in the dedicated ancient DNA facilities of the McMaster Ancient DNA Centre of McMaster University. Following subsampling, 300 mg of bone material were reduced to small particle sizes ranging from 1 to 5 mm using a hammer and chisel. The subsample was then demineralized with 0.5 ml of 0.5 M EDTA pH 8 at room temperature for 24 h with agitation, and the supernatant removed following centrifugation. The pellet was then digested with 0.5 ml of a Tris–HCl-based proteinase K solution with 20 mM Tris–Cl pH 8, 0.5% sodium lauryl sarcosine (SDS, Fisher Scientific), 1% polyvinylpyrrolidone (PVP, Fisher scientific), 50 mM dithiothreitol (DTT), 2.5 mM N-phenacyl thiazolium bromide (PTB, Prime Organics), 2.5 mM calcium chloride (CaCl_2_) and 250 µg ml^−1^ proteinase K. Proteinase digestion was performed at room temperature for 24 h, with agitation. Following centrifugation the digestion supernatant was removed and pooled with the demineralization supernatant. We repeated this process three more times for a total of four rounds of demineralization and digestion. Organics were then extracted from the pooled supernatants using phenol : chloroform : isoamyl alcohol (PCI, 25 : 24 : 1), and the resulting post centrifugation aqueous solution was extracted with chloroform. We then concentrated the final aqueous phase with 10 kDA Amicon Ultra-4 centrifugal filters (Millipore) at 4000*g*, with four washes using 0.1 × TE buffer pH 8 to provide a concentrate of 50 µl. This concentrate was purified using the MinElute PCR Purification kit (QIAGEN) with two washes of 700 µl Buffer PE and eluted in 50 µl Buffer EB and 0.05% Tween-20. An extraction blank, which represents an aliquot of the extraction buffer minus any sample, was carried alongside the *Mylodon* sample during the entire extraction procedure to monitor for possible external contamination during handling.

We used 25 µl of the DNA extract and of the extraction blank in the Illumina library preparation as described elsewhere [17] replacing all SPRI bead cleanups with MinElute purification to 20 µl Buffer EB. We did not heat-deactivate the Bst polymerase following the fill-in step and instead purified the reaction with MinElute into 20 µl Buffer EB. The libraries were then index amplified using the common P5 and a set of unique P7 indexing primers [17] in 50 ml reactions consisting of 1 PCR buffer II, 2.5 mM MgCl_2_, 250 mM deoxynucleotide (dNTP) mix, 200 nM each forward (P5) and reverse (P7) primer, 2.5 U AmpliTaq Gold DNA Polymerase (ThermoFisher Science), and 2 ml (100 ng) of template library. Thermal cycling conditions were as follows: initial denaturation at 95°C for 4 min, 12 cycles of 95°C for 30 s, 60°C for 30 s, 72°C for 30 s and a final extension at 72°C for 10 min. The amplification was performed using a MJ thermocycler (Bio-Rad). The indexed libraries were finally purified with MinElute to 15 µl Buffer EB. A qPCR using 16S MT-rRNA sloth-specific primers was performed on both the *Mylodon* and the blank extract libraries, which we have previously shown to be sensitive to approximately 10 starting copies. The *Mylodon* library had over 10 000 sloth-specific starting copies whereas the blank library did not show any significant sloth-specific amplification, which is not surprising given the strict conditions under which the experiments were conducted. The *Mylodon* library was sequenced at McMaster Genomics Facility as part of an Illumina HiSeq 1500 lane using single-end 72 bp reads.

### (b) Target sequence capture and sequencing

Baits for DNA sequence capture were designed using xenarthran sequences obtained in previous studies [18–20] for the following seven targeted nuclear exons: ADORA3 (321 bp), APOB (2420 bp), BCHE (987 bp), BRCA1 (2835 bp), BRCA2 (3983 bp), RAG2 (441 bp) and TTN (4437 bp). For each exon, 80mer baits were generated with a 4× tiling density. This yielded approximately 20 bp probe spacing, or 60 bp probe overlap. Tiling was flexible to ensure even distribution of baits across the loci, as most loci were not perfect multiples of 20. All baits were then BLASTed against the two-fingered sloth (*Choloepus hoffmanni*; NCBI assembly GCA_000164785.2) and nine-banded armadillo (*Dasypus novemcinctus*; NCBI assembly GCA_000208655.2) genome sequences. Baits with more than one hit and a Tm outside the range 35–40°C were conservatively excluded. This generated a final set of 4381 baits that were synthetized as a myBaits kit by MYcroarray (Ann Arbor, MI, USA). Twenty-two of the modern xenarthran libraries previously prepared by Gibb *et al*. [21], were enriched with the designed bait set in order to capture target sequences representing the seven loci of interest for a representative diversity of Xenarthra (electronic supplementary material, table S1). Enrichment for the *Mylodon* library using the same bait set was conducted in a completely separate ancient DNA facility to avoid contamination. For all libraries, we performed two rounds of enrichments using the methodology previously described in Enk *et al*. [22].

In order to re-amplify the captured sequences, a LibQ Master Mix was prepared. This contained 20 µl of KAPA SYBR FAST qPCR Master Mix (2×), 0.60 µl Forward Primer 1469 (150 nM) and 0.60 µl Reverse Primer 1470 (150 nM) per reaction. The LibQ Mix was added to the 18.8 µl of captured template and amplified on a CFX. Amplification cycling protocols were as follows: 95°C for 5 min; cycle 12 times through 95°C for 30 s, 60°C for 45 s; finally hold at 60°C for 3 min. Following this, the supernatant was removed and saved, yielding the captured library. This was purified using a MinElute PCR Purification Kit (Qiagen) using their standard protocol, yielding a final enriched and purified library suspended in 15 µl of Buffer EB.

All libraries to be sequenced were pooled together at varying concentrations with the aim of creating a single solution containing approximately 250 pM of DNA post size selection. In general, each library was calculated to ideally produce one million reads for sequencing. Libraries then underwent size selection to decrease the amount of non-target DNA and increase sequencing efficiency. Size selection was carried out on a 2% gel (50 ml agarose/1× TAE with 2 µl EtBr). Loading dye equivalent to one-fifth of the library volume was added and samples were then run through the gel for 30 min at 100 V. A 50 bp ladder was used for determining DNA position and size, and the area from approximately 50 to 150 bp was excised. The excised gel was then purified using a MinElute Gel Extraction Kit (Qiagen) column eluted into 60 µl of Buffer EB. Final pool concentrations prior to sequencing were verified using a 2100 Bioanalyzer (Agilent). Sequencing of the enrichment set was performed at McMaster Genomics Facility on an Illumina MiSeq instrument using 150 bp paired-end reads.

### (c) Mitogenome and nuclear exons assemblies

Shotgun sequenced raw reads obtained from the *Mylodon* library were adapter-trimmed using cutadapt v. 1.1 [23] with a quality score cut-off of 30. Contigs were created by *de novo* assembly of the cleaned reads using ABySS v. 1.3.4 [24] with default parameters and a range of increasing kmers. The resulting 480 662 non-redundant contigs were mapped to the *Choloepus didactylus* reference mitogenome (NC_006924) using the ‘medium low sensitivity’ settings in Geneious R9 [25]. Iterative mapping of the reads using the more stringent ‘low sensitivity’ settings was subsequently used to fill the gaps in the assembly of the 174 successfully mapped contigs. The consensus sequence was called using 50% read agreement and all reads were remapped on the consensus to estimate the depth of coverage. Repeating the same procedure using *Bradypus variegatus* (NC_028501) instead of *C. didactylus* as a reference resulted in the same 174 contigs being mapped and the exact same *Mylodon* mitogenome being reconstructed. The final *Mylodon* mitogenome was then annotated by alignment to the *C. didactylus* reference genome.

Raw reads containing imperfect index combinations were discarded. Index and adapter sequences were removed and overlapping pairs merged with leeHom [26], and then mapped to all xenarthran reference exon sequences available with a modified version of BWA [27,28] with a maximum edit distance of 0.01 (-*n* 0.01), allowing a maximum of two gap openings (-o 2) and with seeding effectively disabled (-l 16569). Mapped reads were additionally filtered to those that were either merged or properly paired [29], had unique 5′ and 3′ mapping coordinates [30], and then restricted to reads of at least 24 bp with SAMtools [31]. The bam files were then imported into Geneious for careful assessment by eye for enrichment success and selection of the best assembly for each sequence depending on the most successful reference sequence. Consensus sequences were called with a 50% threshold and a minimum coverage of 2× with ambiguous nucleotides called at sites where the two reads disagreed and there was no third read. As expected, nuclear capture success was variable among both taxa and loci with sloths and anteaters being successfully enriched for all loci, whereas armadillos presented some loci for which the coverage was insufficient to confidently call a consensus sequence. The capture experiment nevertheless enabled us to produce a total of 144 newly assembled xenarthran sequences for the seven nuclear exons targeted (electronic supplementary material, table S1).

### (d) DNA damage and metagenomic analyses

Analyses of post-mortem C to T and G to A mutations in the 53 550 reads mapping to the reconstructed *Mylodon* mitogenome were conducted using mapDamage 2.0 [32]. For comparisons, four modern xenarthran species from Gibb *et al*. [21] and the extinct glyptodont *Doedicurus* [33] were also analysed. For metagenomic analyses, Megahit v. 1.1.1 [34] was used to assemble the *Mylodon* shotgun reads. The resulting 385 contigs of more than 200 bp were then subjected to similarity searches against the GenBank nucleotide database (version of 28 April 2017) using Megablast followed by taxonomic assignation using MEGAN 6 with default LCA parameters [35] and subsequent graphical representation with Krona [36].

### (e) Phylogenetic and dating analyses

For constructing the mitogenomic supermatrix, we chose 31 representative living xenarthran species from the dataset of Gibb *et al*. [21] plus three afrotherian outgroups (electronic supplementary material, table S1). We then added *M. darwinii* sequences (excluding the variable control region) and aligned each gene with MAFFT [37] within Geneious guided by translation for the protein-coding genes. We removed ambiguously aligned sites on each dataset with Gblocks [38] using default relaxed parameters. The final mitogenomic matrix contained 15 222 sites for 35 taxa representing all living xenarthran species plus the extinct *M. darwinii*.

For assembling the seven nuclear exons supermatrix, the newly obtained *Mylodon* and modern xenarthran sequences were added to available sequences plus the same three afrotherian outgroups (electronic supplementary material, table S1). Each exon was then aligned by translation with MAFFT within Geneious. We removed ambiguously aligned codons on each alignment with Gblocks using relaxed default parameters. The final nuclear exons matrix contained 15 216 sites for 28 taxa encompassing all living xenarthran genera and the extinct *M. darwinii* with an overall percentage of missing data of only 16%. Importantly, *Mylodon* was represented at 10 238 unambiguous sites (67.28% of the total) of the nuclear concatenated dataset.

The best-fitting partition schemes were determined for both datasets using PartitionFinder v. 1.1.1 [39]. For the mitogenomic dataset, we used the greedy algorithm on 42 *a priori* partitions corresponding to codon positions, 12S MT-rRNA, 16S MT-rRNA and all tRNAs, with unlinked branch lengths, and using the Bayesian information criterion (BIC) for model selection (electronic supplementary material, table S2). For the nuclear dataset, we used the greedy algorithm on 21 *a priori* partitions corresponding to codon positions, with linked branch lengths, and the BIC for model selection (electronic supplementary material, table S3). For both datasets, ML partitioned reconstruction was conducted with RAxML 8.2.8 [40] using the best-fitting scheme with parameters unlinked across partitions. Maximum-likelihood bootstrap values (BP_PART_) were obtained after 100 replicates. Bayesian phylogenetic inference under a mixed model was performed using MrBayes 3.2.3 [41] using the best-fitting scheme with parameters unlinked across partitions. Two independent sets of four MCMCs were run for 1 000 000 generations sampling every 1000 generations. After a burn-in of 25%, the 50% majority-rule consensus tree and associated clade posterior probabilities (PP_PART_) were computed from the 1500 trees combined in the two independent runs. Bayesian phylogenetic reconstruction was also conducted under the CAT-GTR + G_4_ mixture model using PhyloBayes MPI 1.7b [42]. Two independent MCMCs were run for 50 000 cycles sampling every 10 cycles during 2 750 000 tree generations. After a burn-in of 10%, the 50% majority-rule consensus tree and associated clade posterior probabilities (PP_CAT_) were computed from the 9000 combined trees of the two runs using bpcomp.

Molecular dating analyses were conducted on both datasets using PhyloBayes 3.3f [43] under the CAT-GTR+G_4_ mixture model and a log-normal autocorrelated relaxed clock with a birth–death prior on divergence times combined with soft fossil calibrations. We used the same best-fitting relaxed clock model, six fossil calibrations, and priors as in Gibb *et al*. [21] so that the divergence dates obtained could be directly compared between the two studies. Calculations were conducted in each case by running two independent MCMCs for a total 50 000 cycles sampling every 10 cycles. The first 500 samples (10%) were excluded as burn-in after convergence diagnostics. Posterior estimates of divergence dates were computed from the remaining 4500 samples of each MCMC using readdiv.

## 3. Results and discussion

### (a) A new high quality *Mylodon* mitogenome

**Figure 1.**
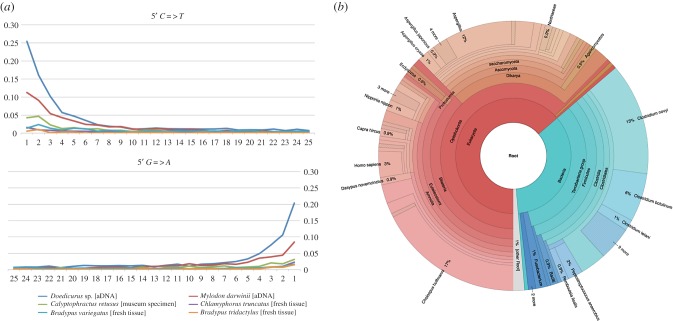
(*a*) DNA damage profiles from the *Mylodon* bone sample (12 880 ± 35 ^14^C yrpb) compared to a fossil glyptodont sample (*Doedicurus* sp.) dated at (12 015 ± 50 ^14^C yrpb), a 40-year-old museum specimen of greater fairy armadillo (*Calyptophractus retusus*) and three modern xenarthran samples. (*b*) Taxonomic assignation of 223 contigs assembled from *Mylodon* shotgun reads represented with Krona.

The radiocarbon date we obtained for the *Mylodon* bone sample NHMUK PV M8758 is 12 880 ± 35 ^14^C yrbp (radiocarbon years before present), which is fully congruent with previous estimates for other samples from Mylodon Cave [2]. Illumina sequencing of the *Mylodon* shotgun library produced a total of 28 020 236 single-end 72 bp reads. Low, yet consistent DNA damage on those reads shows that our ancient *Mylodon* sample was exceptionally well preserved and thus helps support its authenticity (figure 1*a*; electronic supplementary material, figures S1 and S2). Estimates of C to T and G to A transitions caused by post-mortem mutations (up to 11% and 8% respectively) were intermediate between those of the subfossil *Doedicurus* sp. osteoderm sample of the same age (up to 25% and 20%) and the *Calyptophractus* museum specimen (up to 4% and 3%), whereas modern xenarthran tissue samples exhibit values below 1%. These values argue in favour of the endogenous origin of the *Mylodon* shotgun reads and set our bone sample among some of the best ancient samples analysed so far [44]. The consistently cold and dry conditions encountered at Mylodon Cave [4] probably explain the exceptional preservation of samples coming from this location [5].

Metagenomic analyses indicate that 30% of the 222 taxonomically assigned contigs (out of a total of 385) are mammalian in origin: 22% are xenarthrans, with 17% matching specifically to the two-fingered sloth (*C. hoffmanni*) genome assembly (figure 1*b*). The only 3% of contigs assigned to *Homo sapiens* in fact match conserved portions of the mammalian 18S and 45S ribosomal RNA genes, and thus could not be considered to represent human-specific contamination. Moreover, 25% of the assigned contigs are fungal in origin, with *Aspergillus* mould representing 12%. Finally, 35% of the assigned contigs belong to Bacteria, 28% being assigned to the genus *Clostridium* with dominant taxa such as *C. novyi* (13%) and *C. botulinum* (6%) that are commonly found in ancient DNA samples and soil. To further quantify possible contamination from humans or other mammals found in Mylodon Cave such as *Hippidion saldiasi*, *Lama guanicoe*, *Dusicyon avus* and *Panthera onca mesembrina* [4], we mapped our shotgun reads to available mitogenomes for these species, or to those of closely related ones, using the low sensitivity settings of Geneious. None of the mapping results were convincing apart from a few reads, which mapped to conserved regions of the mammalian mitogenome (data not shown). For instance, we had only 536 reads mapping to conserved regions of the human reference mitogenome (NC_012920), again supporting the authenticity of our sample.

The assembly of 53 550 shotgun reads allowed for the reconstruction of a high quality mitogenome for *M. darwinii* at an average depth of 99× (range 1× to 307×). As independent replication is an important component of the ancient DNA research agenda [45], we verified that our consensus sequence matched perfectly to previously obtained PCR fragments attributed to *Mylodon* for the mitochondrial 12S and 16S MT-rRNAs [5] and MT-CYTB [6] genes. However, a comparison with a recently published mitogenome sequence [15] revealed a number of discrepancies (electronic supplementary material, figure S11). The two sequences are identical at only 81% of total sites (89% when excluding ambiguous sites and the control region). The Slater *et al*. [15] sequence (KR336794) also contains 1383 Ns and presents a substantial number of substitutions in otherwise conserved regions of the sloth mitogenome when compared with *C. didactylus*, including frameshifting substitutions causing stop codons in 11 out of the 13 coding genes (electronic supplementary material, table S4). These differences probably represent errors that were incorporated into the assembly resulting from overall lower coverage depth of the composite mitogenome, reconstructed from captured sequences stemming from both bone and paleofecal material. Mapping the reads produced by Slater *et al*. [15] (SRA accession SRR2007674) to our *Mylodon* mitogenome confirmed that these divergent regions correspond to regions of low depth of coverage in their capture experiment (data not shown). These errors probably explain an artificially inflated branch length in the phylogenetic tree, potentially impacting the inference of the divergence date between the extinct *Mylodon* and modern sloths (electronic supplementary material, figure S12).

### (b) Nuclear data corroborate the phylogenetic position of *Mylodon darwinii*

**Figure 2.**
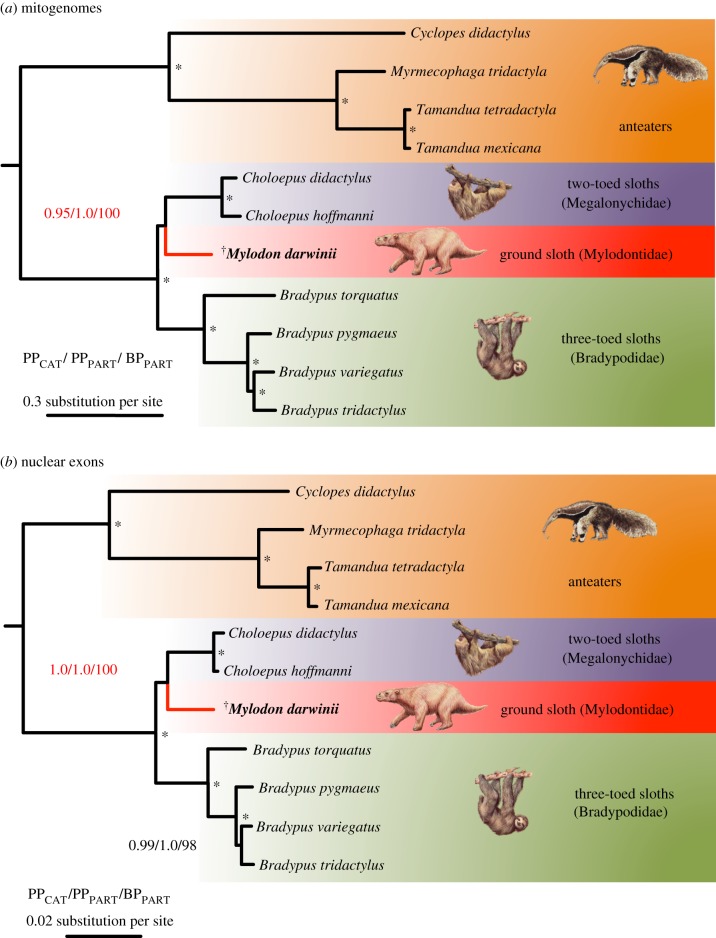
Bayesian consensus phylograms of Pilosa obtained under the site-heterogeneous CAT-GTR+G_4_ mixture model for the (*a*) mitogenomic and (*b*) nuclear datasets. Values at nodes represent clade posterior probabilities under the CAT model (PP_CAT_), mixed model (PP_PART_), and maximum-likelihood bootstrap percentages under a partitioned model (BP_ML_). Asterisks indicate maximum support from all statistical indices. The complete phylograms are available in electronic supplementary material, figures S3–S8. Graphical representation and taxon images derive from Gibb *et al*. [21].

Phylogenetic analyses of our complete xenarthran mitogenomic dataset using Bayesian and ML methods recovered the same strongly supported topology in which *Mylodon* is the sister-group of modern two-fingered sloths of the genus *Choloepus* (figure 2*a*). The statistical support for this phylogenetic position was high with the Bayesian mixture model (PP_CAT_ = 0.95) and maximal with the Bayesian and ML partitioned models (PP_PART_ = 1; BP_PART_ = 100). All other nodes within Pilosa received maximal support from all methods. These results obtained by including the full species diversity of modern sloths add support to the position of *M. darwinii* originally suggested by short mitochondrial PCR fragments [5,6] and recently supported by mitogenomic analyses including fewer taxa [15]. The phylogenetic picture provided by the mitochondrial genome alone could nevertheless be misleading in cases of mito-nuclear discordance caused by factors such as adaptive introgression or past hybridization events [46]. It was thus important to substantiate our mitogenomic results by nuclear data. In this case, the same phylogenetic analyses applied to the supermatrix of the concatenated seven nuclear exons yield identical results with maximum statistical support placing *Mylodon*, once again as the sister-group of two-fingered sloths (figure 2*b*). Such perfect topological congruence between mitochondrial genomes and nuclear markers provides clear and convincing evidence of the phylogenetic position of the extinct Darwin's ground sloth within the evolutionary history of sloths. These phylogenetic results provide further support for the diphyletic origin of modern sloths, implying an independent evolution of arboreality from terrestrial ancestors [47,48] and the independent evolution of their unique suspensory lifestyle, resulting in numerous convergent anatomical adaptations [49,50].

**Figure 3.**
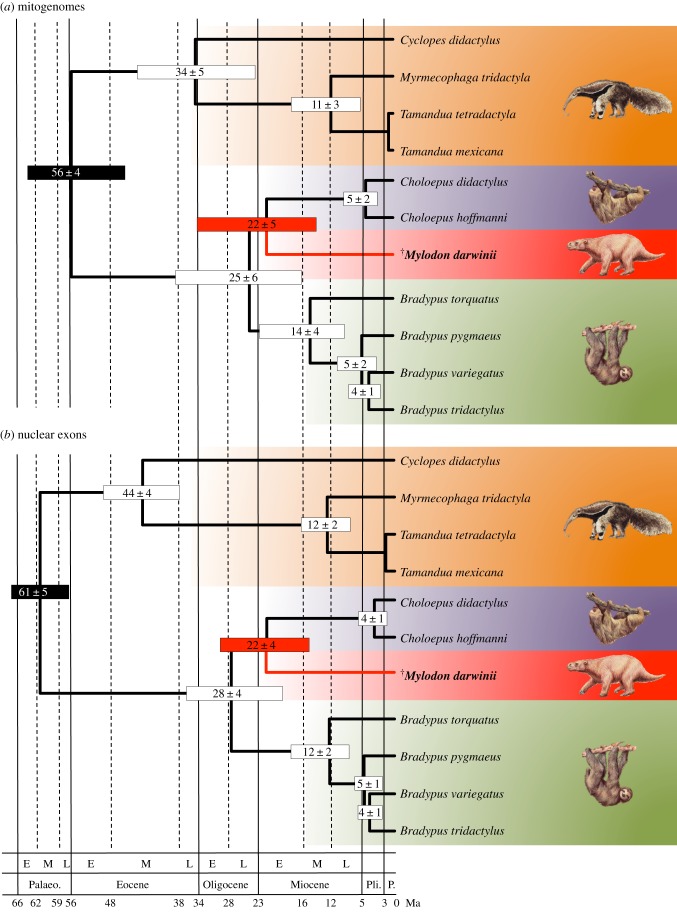
Bayesian chronograms of Pilosa obtained using a rate-autocorrelated log-normal relaxed molecular clock model under the CAT-GTR+G_4_ mixture model with a birth death prior on the diversification process, and six soft calibration constraints for the (*a*) mitogenomic and (*b*) nuclear datasets. Mean divergence dates and associated 95% credibility intervals are represented as node bars. Plain black node bars indicated calibration constraints. The main geological periods follow the geological time scale of the Geological Society of America (E, early; M, middle; L, late; Palaeo., Palaeocene; Pli., Pliocene; P., Pleistocene). The complete chronograms are available in the electronic supplementary material (electronic supplementary material, figures S9 and S10). Graphical representation and taxon images derive from Gibb *et al*. [21].

Relaxed molecular clock analyses of both the mitogenomic and nuclear datasets recover relatively ancient dates for the common ancestor of sloths (figure 3, table 1). The corresponding divergence between the two modern sloth genera *Bradypus* (Bradypodidae) and *Choloepus* (Megalonychidae) is estimated to be 25 ± 6 Ma with the mitogenomic dataset (figure 3*a*), and 28 ± 4 Ma with the nuclear supermatrix (figure 3*b*). These inferred dates are relatively older than previous estimates based on nuclear data but with reduced taxon sampling [19,51], further justifying their classification into distinct families. The difference with previous nuclear dates might have to do with the CAT-GTR+G4 mixture model which allows for a better correction of substitutional saturation combined with the inclusion of the internal xenarthran calibration points here provided by the earliest fossil cingulate skull [52].

**Table 1.** Divergence time estimates for the main xenarthran nodes inferred using the site-heterogeneous CAT-GTR+G_4_ substitution model and an autocorrelated log-normal (LN) relaxed molecular clock model. Mean posterior estimates, associated standard errors and 95% credibility intervals are expressed in million years ago (mean date ± s.d. [95% CredI]). s.d., standard deviation; 95% CredI, 95% credibility interval; MRCA, most recent common ancestor.

nodes	mitogenomes	nuclear exons
Xenarthra^a^	67.3 ± 3.2 [59.9–71.4]	69.3 ± 2.2 [63.5–71.8]
Pilosa MRCA^a^ (anteaters + sloths)	55.6 ± 4.4 [46.4–63.2]	61.2 ± 2.5 [55.4–65.0]
Folivora MRCA^a^ (sloths)	24.8 ± 6.2 [15.8–37.6]	27.7 ± 4.0 [19.6–35.9]
Mylodontidae + Megalonychidae (two-fingered sloths)	21.9 ± 5.7 [13.3–33.9]	22.5 ± 3.7 [15.5–30.6]
Megalonychidae MRCA (two-fingered sloths)	4.7 ± 1.5 [2.5–8.3]	3.8 ± 1.1 [2.2–6.5]
Bradypodidae MRCA (three-fingered sloths)	14.3 ± 4.1 [8.4–23.1]	12.1 ± 2.4 [7.8–17.5]
*B. pygmaeus*/others	5.4 ± 1.8 [2.8–9.6]	4.5 ± 1.1 [2.7–7.0]
*B. tridactylus*/*B. variegatus*	4.2 ± 1.5 [2.2–7.7]	3.9 ± 1.0 [2.3–6.2]
Vermilingua MRCA^a^ (anteaters)	34.2 ± 5.1 [23.9–44.2]	43.7 ± 3.2 [36.6–49.7]
Myrmecophaga/Tamandua	10.7 ± 3.0 [5.6–17.6]	11.9 ± 2.1 [8.4–16.7]
*T. mexicana*/*T. tetradactyla*	0.8 ± 0.3 [0.4–1.5]	2.0 ± 0.5 [1.2–3.2]
Cingulata MRCA (armadillos)	44.2 ± 3.5 [37.9–51.5]	42.3 ± 2.4 [37.7–47.2]
Dasypodidae MRCA (long-nosed armadillos)	11.5 ± 3.4 [7.2–20.4]	8.7 ± 1.6 [6.3–12.4]
Chlamyphoridae MRCA	36.6 ± 3.3 [31.2–44.1]	33.4 ± 2.1 [29.7–38.2]
Euphractinae MRCA (hairy armadillos)	10.3 ± 2.7 [6.4–16.6]	6.1 ± 1.3 [4.0–9.2]
Chlamyphorinae/Tolypeutinae	32.4 ± 3.1 [27.8–39.7]	31.5 ± 2.0 [28.0–36.0]
Chlamyphorinae MRCA (fairy armadillos)	19.7 ± 2.7 [15.5–26.3]	14.8 ± 2.4 [10.4–19.9]
Tolypeutinae MRCA^a^	25.8 ± 2.6 [22.5–32.5]	23.7 ± 1.3 [21.7–27.1]
*Tolypeutes*/*Cabassous*	22.5 ± 2.5 [19.1–28.8]	21.1 ± 1.5 [18.6–24.6]
*Tolypeutes* MRCA	13.7 ± 2.0 [10.7–18.4]	11.9 ± 1.5 [9.2–15.2]
*Cabassous chacoensis/C. unicinctus*	8.4 ± 1.5 [5.9–11.9]	6.5 ± 1.4 [4.1–9.4]

^a^Used as *a priori* calibration constraints.

Our results reveal that *Mylodon* separated from two-fingered sloths of the genus *Choloepus* early in sloth evolutionary history, with both datasets clearly agreeing upon a divergence date of about 22 Ma (figure 3 and table 1). The dating estimate obtained with our revised *Mylodon* mitogenome (22 ± 5 Ma; figure 3*a*) is somewhat comparable with previous estimates proposed by Slater *et al*. [15] based on a different approach. These authors used a recently developed dating method based on the fossilized birth–death (FBD) process that allows one to directly incorporate fossil taxa [53], but requires the use a topological constraint including both fossil and modern taxa. Because of the uncertainty associated with the phylogenetic position of some key sloth fossil taxa, Slater *et al*. [15] found that their divergence date estimates under the FBD process were highly sensitive to the treatment of ambiguous Deseadan fossil taxa as representing either stem or crown fossil folivorans. By contrast, using nodal calibrations and a better sampling of modern species, our results appear intermediate with those obtained with the two alternative treatments. In fact, the diversification of sloth lineages in the Early Miocene (25–22 Ma) corresponds with the end of the first major Bolivian tectonic event, when the Andes became the principal relief of South America, significantly influencing palaeoclimates [54]. This period led to a major shift in South American mammalian fossil communities including the Miocene radiation of ground sloths [55]. Our results based on an updated mitogenomic dataset and a new nuclear supermatrix corroborate *M. darwinii* as belonging to a distinct lineage of sloths (family Mylodontidae) originating more than 22 Ma and persisting until their extinction only some 10 000 years BP [56,57].

## 4. Conclusion and perspectives

Our study provides a high-quality complete mitochondrial genome as well as phylogenetically informative nuclear loci for the extinct Darwin's ground sloth. Analyses of these new data validate the phylogenetic position of *M. darwinii* as a member of a distinct lineage (Mylodontidae) and as a sister group to modern two-fingered sloths (genus *Choloepus*; Megalonychidae), originating about 22 Ma. The exceptional preservation of these cave-preserved *Mylodon* bone samples will enable complete genome sequencing of this emblematic extinct taxon, generating further insights into their unique features and ultimate extinction.

## Supplementary Material

Electronic supplementary material

## Data accessibility

*Mylodon darwinii* mitogenome: GenBank (MF061314). Nuclear exon data: European Nucleotide Archive (LT852562-LT852705). *Mylodon darwinii* shotgun Illumina reads: Sequence Read Archive (ERR1958375). Additional data, including bait design, alignments and trees: Dryad Digital Repository (http://dx.doi.org/10.5061/dryad.ft3k3) [58].
