# Supplementary material for: Resolving the phylogenetic position of Darwin's extinct ground sloth (Mylodon darwinii) using mitogenomic and nuclear exon data
Source: Proc Biol Sci. 2018 May 16;285(1878):20180214. doi: 10.1098/rspb.2018.0214 (PMC5966596; doi:10.1098/rspb.2018.0214)
Supplement: Electronic supplementary material [file rspb20180214supp1.pdf]

Resolving the phylogenetic position of Darwin's extinct ground sloth (*Mylodon darwinii*) using mitogenomic and nuclear exon data

Frédéric Delsuc, Melanie Kuch, Gillian C. Gibb, Jonathan Hughes, Paul Szpak, John Southon, Jacob Enk, Ana T. Duggan' and Hendrik N. Poinar

Supplementary Material

Table S1: Taxon sampling and sequence accession numbers.
Table S2: Detailed results of the PartitionFinder analysis for the mitogenomic dataset.
Table S3: Detailed results of the PartitionFinder analysis for the nuclear dataset.
Table S4: Detailed comparisons between our *Mylodon* mitogenome and the one produced by Slater et al. (2016).
Figure S1: mapDamage DNA fragmentation and nucleotide mis-incorporation plots.
Figure S2: mapDamage DNA fragment length distributions.
Figure S3: Bayesian mitogenomic tree under the CAT-GTR+G_4 mixture model (PhyloBayes).
Figure S4: Bayesian mitogenomic tree under a mixed model (MrBayes).
Figure S5: Maximum likelihood mitogenomic tree under a GTR+G+I mixed model (RAxML).
Figure S6: Bayesian nuclear tree under the CAT-GTR+G_4 mixture model (PhyloBayes).
Figure S7: Bayesian nuclear tree under a mixed model (MrBayes).
Figure S8: Maximum likelihood nuclear tree under a GTR+G mixed model (RAxML).
Figure S9: Bayesian mitogenomic chronogram under the CAT-GTR+G_4 mixture model and an autocorrelated lognormal model of clock relaxation (PhyloBayes).
Figure S10: Bayesian nuclear chronogram under the CAT-GTR+G_4 mixture model and an autocorrelated lognormal model of clock relaxation (PhyloBayes).
Figure S11: Comparison of the two *Mylodon* mitogenomes aligned with the *Choloepus didactylus* reference mitogenome.
Figure S12: Maximum likelihood tree of sloth mitogenomes under a single GTR+G model (RAxML).

Table S1. Detailed taxonomic sampling and corresponding sequence accession numbers of the mitochondrial and nuclear datasets. Newly acquired sequences from this study are indicated in bold.

Species	Common name	Mitogenomes	ADORA3	APOB	BCHE	BRCA1	BRCA2	RAG2	TTN
**Bradypus pygmaeus*	Pygmy sloth	KT818523	**LT852562**	**LT852583**	**LT852604**	**LT852627**	**LT852645**	**LT852662**	**LT852684**
**Bradypus torquatus*	Maned sloth	KT818524	**LT852563**	**LT852584**	**LT852605**	**LT852628**	**LT852646**	**LT852663**	**LT852685**
**Bradypus tridactylus*	Pale-throated three-toed sloth	KT818525	**LT852564**	**LT852585**	**LT852606**	**LT852629**	**LT852647**	**LT852664**	**LT852686**
**Bradypus variegatus*	Brown-throated three-toed sloth	NC_006923	**LT852565**	**LT852586**	**LT852607**	**LT852630**	**LT852648**	**LT852665**	**LT852687**
**Choloepus didactylus*	Southern two-toed sloth	NC_006924	**LT852570**	**LT852591**	**LT852613**	**LT852634**	**LT852651**	**LT852671**	**LT852693**
**Choloepus hoffmanni*	Hoffmann's two-toed sloth	KT818538	**LT852571**	**LT852592**	**LT852614**	**LT852635**	**LT852652**	**LT852672**	**LT852694**
**Mylodon darwinii*	Darwin's ground sloth	**MF061314**	**LT852575**	**LT852596**	**LT852619**	**LT852639**	**LT852656**	**LT852676**	**LT852698**
**Cyclopes didactylus*	Pygmy anteater	KT818539	**LT852572**	**LT852593**	**LT852615**	**LT852636**	**LT852653**	**LT852673**	**LT852695**
**Myrmecophaga tridactyla*	Giant anteater	KT818549	**LT852576**	**LT852597**	**LT852620**	**LT852640**	**LT852657**	**LT852677**	**LT852699**
**Tamandua mexicana*	Northern tamandua	KT818551	**LT852578**	**LT852599**	**LT852622**	**LT852641**	**LT852659**	**LT852679**	**LT852701**
**Tamandua tetradactyla*	Southern tamandua	NC_004032	**LT852579**	**LT852600**	**LT852623**	**LT852642**	**LT852660**	**LT852680**	**LT852702**
**Dasypus kappleri*	Greater long-nosed armadillo	KT818541	**LT852574**	**LT852595**	**LT852617**	**LT852638**	**LT852655**	**LT852675**	**LT852697**
**Dasypus hybridus*	Southern long-nosed armadillo	KT818540	**LT852573**	**LT852594**	**LT852616**	**LT852637**	**LT852654**	**LT852674**	**LT852696**
Dasypus septemcinctus	Seven-banded armadillo	KT818546	-	-	-	-	-	-	-
Dasypus novemcinctus US	Nine-banded armadillo	NC_001821	XM_004473603	JN413957	XM_012519796	AF484222	XM_012519803	XM_004483174	XM_012518926
Dasypus novemcinctus FG	Nine-banded armadillo	KT818542	-	-	-	-	-	-	-
Dasypus pilosus	Hairy long-nosed armadillo	KT818544	-	-	-	-	-	-	-

Dasypus sabanicola	Northern long-nosed armadillo	KT818545	-	-	-	-	-	-	-
Dasypus yepesi	Yunga's long-nosed armadillo	KT818547	-	-	-	-	-	-	-
**Chaetophractus vellerosus*	Screaming hairy armadillo	KT818533	**LT852568**	**LT852589**	**LT852611**	**LT852632**	NA	**LT852669**	**LT852691**
**Chaetophractus villosus*	Large hairy armadillo	KT818535	**LT852569**	**LT852590**	**LT852612**	**LT852633**	NA	**LT852670**	**LT852692**
**Euphractus sexcinctus*	Six-banded armadillo	KT818548	AY011193	NA	**LT852618**	AF484224	NA	AY011922	NA
**Zaedyus pichiy*	Pichi	KT818555	**LT852582**	**LT852603**	**LT852626**	**LT852644**	NA	**LT852683**	**LT852705**
**Calyptophractus retusus*	Greater fairy armadillo	KT818532	**LT852567**	**LT852588**	**LT852610**	FR821709	**LT852650**	**LT852668**	**LT852690**
Chlamyphorus truncatus	Pink fairy armadillo	KT818536	-	-	-	FR821708	-	-	-
**Priodontes maximus*	Giant armadillo	KT818550	**LT852577**	**LT852598**	**LT852621**	AF484225	**LT852658**	**LT852678**	**LT852700**
**Tolypeutes matacus*	Southern three-banded armadillo	KT818553	**LT852580**	**LT852601**	**LT852624**	AF484227	**LT852661**	**LT852681**	**LT852703**
**Tolypeutes tricinctus*	Brazilian three-banded armadillo	KT818554	**LT852581**	**LT852602**	**LT852625**	**LT852643**	NA	**LT852682**	**LT852704**
**Cabassous chacoensis*	Chacoan naked-tailed armadillo	KT818528	NA	NA	**LT852608**	**LT852631**	NA	**LT852666**	**LT852688**
**Cabassous unicinctus*	Southern naked-tailed armadillo	KT818531	**LT852566**	**LT852587**	**LT852609**	AF484228	**LT852649**	**LT852667**	**LT852689**
Cabassous centralis	Northern naked-tailed armadillo	KT818527	-	-	-	-	-	-	-
Cabassous tatouay	Greater naked-tailed armadillo	KT818529	-	-	-	-	-	-	-
Dugong dugon	Dugong	NC_003314	JN633415	KR827337	KR827307	AF284019	JN414242	JN633302	JN632896
Loxodonta africana	African elephant	NC_000934	AY011204	JN413956	XM_010596053	AF284021	JN414245	AY011932	XM_010586379
Orycteropus afer	Aardvark	NC_002078	AY011206	JN413950	XM_007948719	AF284030	JN414239	AY011935	JN632894

Notes -- *: Libraries selected for nuclear capture; NA: Not available because capture failed to provide enough coverage.

Table S2: Detailed results of the PartitionFinder analysis for the mitogenomic dataset.

Subset	Best Model	Subset Partitions
1	GTR+I+G	ATP6_p1, ATP8_p1, ATP8_p2, ATP8_p3, CYTB_p1, ND1_p1, ND2_p1, ND3_p1, ND4L_p1, ND4_p1, ND5_p1, ND6_p2, ND6_p3, rRNA_12S, rRNA_16S, tRNAs
2	GTR+I+G	ATP6_p2, COX1_p1, COX1_p2, COX2_p1, COX2_p2, COX3_p1, COX3_p2, CYTB_p2, ND1_p2, ND2_p2, ND3_p2, ND4L_p2, ND4_p2, ND5_p2
3	GTR+I+G	ATP6_p3, COX1_p3, COX2_p3, COX3_p3, CYTB_p3, ND1_p3, ND2_p3, ND3_p3, ND4L_p3, ND4_p3, ND5_p3, ND6_p1

Settings used

alignment : ./mylo_35mitos_concat_seqCat.phylip
branchlengths : unlinked
models : GTR+I+G, GTR+G
model_selection : bic
search : greedy

Best partitioning scheme

Scheme Name : step_39
Scheme lnL : -169349.5486
Scheme BIC : 340923.742014
Number of params : 231
Number of sites : 15222
Number of subsets : 3

RaxML-style partition definitions

DNA, p1 = 1-681\3, 682-876\3, 683-876\3, 684-876\3, 3883-5022\3, 5023-5976\3, 5977-7020\3, 7021-7365\3, 7366-7662\3, 7663-9039\3, 9040-10842\3, 10844-11367\3, 10845-11367\3, 11368-12305, 12306-13766, 13767-15222
DNA, p2 = 2-681\3, 877-2415\3, 878-2415\3, 2416-3099\3, 2417-3099\3, 3100-3882\3, 3101-3882\3, 3884-5022\3, 5024-5976\3, 5978-7020\3, 7022-7365\3, 7367-7662\3, 7664-9039\3, 9041-10842\3
DNA, p3 = 3-681\3, 879-2415\3, 2418-3099\3, 3102-3882\3, 3885-5022\3, 5025-5976\3, 5979-7020\3, 7023-7365\3, 7368-7662\3, 7665-9039\3, 9042-10842\3, 10843-11367\3

Table S3: Detailed results of the PartitionFinder analysis for the seven nuclear exons dataset.

Subset	Best Model	Subset Partitions
1	GTR+G	ADORA3_pos1, ADORA3_pos2, APOB_pos2
2	GTR+G	ADORA3_pos3, APOB_pos3, RAG2_pos3
3	GTR+G	APOB_pos1, BRCA1_pos1
4	GTR+G	BCHE_pos1, RAG2_pos1, TTN_pos1
5	GTR+I+G	BCHE_pos2, RAG2_pos2, TTN_pos2
6	GTR+G	BCHE_pos3, BRCA2_pos3
7	GTR+G	BRCA1_pos2, BRCA2_pos2
8	GTR+G	BRCA1_pos3, TTN_pos3
9	GTR+G	BRCA2_pos1

Settings used
alignment : ./Mylo_7exons_seqCat.phylip
branchlengths : linked
models : GTR+I+G, GTR+G
model_selection : bic
search : greedy

Best partitioning scheme
Scheme Name : step_12
Scheme lnL : -61830.71542
Scheme BIC : 125038.535538
Number of params : 143
Number of sites : 15216
Number of subsets : 9

RaxML-style partition definitions
DNA, p1 = 1-312\3, 2-312\3, 314-2724\3
DNA, p2 = 3-312\3, 315-2724\3, 10374-10797\3
DNA, p3 = 313-2724\3, 3706-6483\3
DNA, p4 = 2725-3705\3, 10372-10797\3, 10798-15216\3
DNA, p5 = 2726-3705\3, 10373-10797\3, 10799-15216\3
DNA, p6 = 2727-3705\3, 6486-10371\3
DNA, p7 = 3707-6483\3, 6485-10371\3
DNA, p8 = 3708-6483\3, 10800-15216\3
DNA, p9 = 6484-10371\3

Table S4: Detailed comparisons between our *Mylodon* mitogenome and the one produced by Slater et al. (2016).

Annotated regions	Length (bp)	Identity (%)	Problems in Slater et al. (2016) sequence (KR336794)
tRNA-Phe	70	100	None
12S rRNA	963	99.2	1 divergent region (12 bp) including a 4 bp insertion
tRNA-Val	68	100	None
16S rRNA	1575	98.7	1 substitution + 1 divergent region (26 bp) + 3 Ns
tRNA-Leu	75	100	None
ND1	957	88.7	4 substitutions + 3 divergent regions (26 bp, 14bp, 19bp) including missing start codon + 60 Ns (7 stop codons)
tRNA-Ile	69	100	None
tRNA-Gln	73	100	None
tRNA-Met	69	100	None
ND2	1044	91.0	2 substitutions + 4 divergent regions (88 bp, 41 bp, 51 bp, 6 bp) + 3 deletions + 1 insertion + 4 Ns (8 stop codons)
tRNA-Trp	69	100	None
tRNA-Ala	69	100	None
tRNA-Asn	73	100	None
Origin of L-strand replication	34	100	None
tRNA-Cys	67	100	None
tRNA-Tyr	66	100	None
COX1	1542	90.5	3 substitutions + 43 Ns
tRNA-Ser	69	100	None
tRNA-Asp	68	97.1	1 substitution + 1 insertion
COX2	684	94.2	21 substitutions + 2 divergent regions (10 bp, 19 bp)
tRNA-Lys	69	100	None
ATP8	204	52.5	3 substitutions + 94 gaps (1 stop codon)
ATP6	681	41.8	13 substitutions + 3 divergent regions (39 bp, 29 bp, 34 bp) + 175 Ns + 259 gaps (1 stop codon)
COX3	784	96.4	2 substitutions + 1 divergent region (23 bp) + 1 insertion (10 stop codons)
tRNA-Gly	70	100	None
ND3	346	54.3	7 substitutions + 151 Ns
tRNA-Arg	70	100	None
ND4L	297	59.1	1 divergent region (14 bp) + 110 Ns
ND4	1378	85.7	187 Ns + 2 insertions (29 stop codons)
tRNA-His	69	100	None
tRNA-Ser	60	100	None

tRNA-Leu	70	100	None
ND5	1824	53.5	13 substitutions + 2 divergent regions (10 bp, 3 bp) + 809 gaps + 16 Ns (9 stop codons)
ND6	528	72.2	6 substitutions + 1 divergent region (13 bp) + 4 gaps + 126 Ns (2 stop codons)
tRNA-Glu	69	100	None
CYTB	1140	98.9	11 substitutions + 2 insertions (27 stop codons)
tRNA-Thr	67	29.4	22 gaps + 26 Ns
tRNA-Pro	70	0	70 Ns
Control Region	993	60.0	48 gaps + 310 Ns

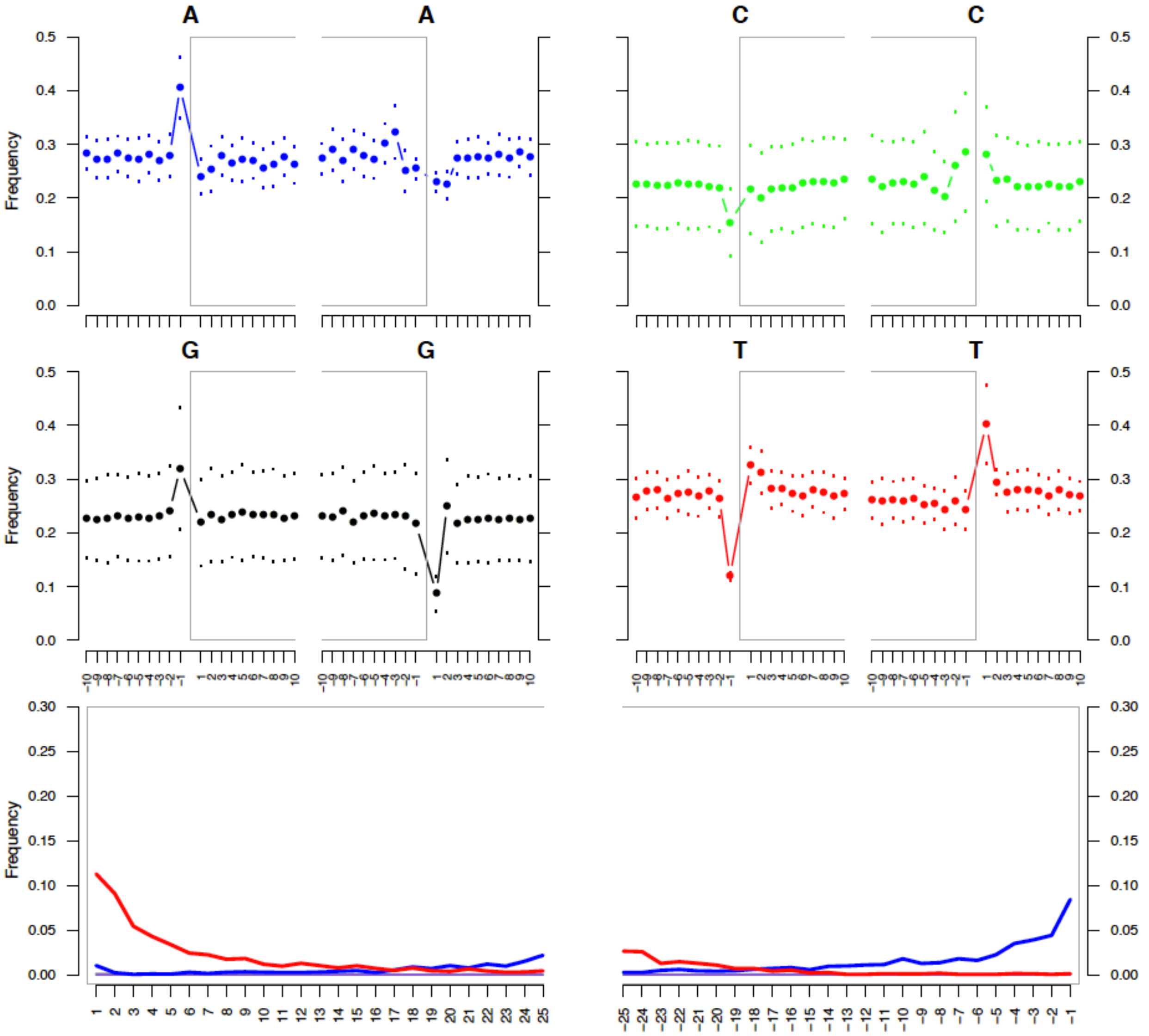

Figure S1. mapDamage DNA fragmentation and nucleotide mis-incorporation plots of the 53,550 cleaned shotgun Illumina reads mapping to the *Mylodon darwinii* SP57 mitogenome. 1st and 2nd rows: DNA fragmentation patterns for the 10 bp preceding read starts (positions -1 to -10 on the left panels of each base composition profile) and the 10 bp following read ends (positions 1 to 10 on the right panels of each base composition profile). 3rd row: Nucleotide mis-incorporation patterns for the first 25 nucleotides and the last 25 sequenced, respectively, with C=>T substitution frequencies (red) and G=>A substitution frequencies (blue).

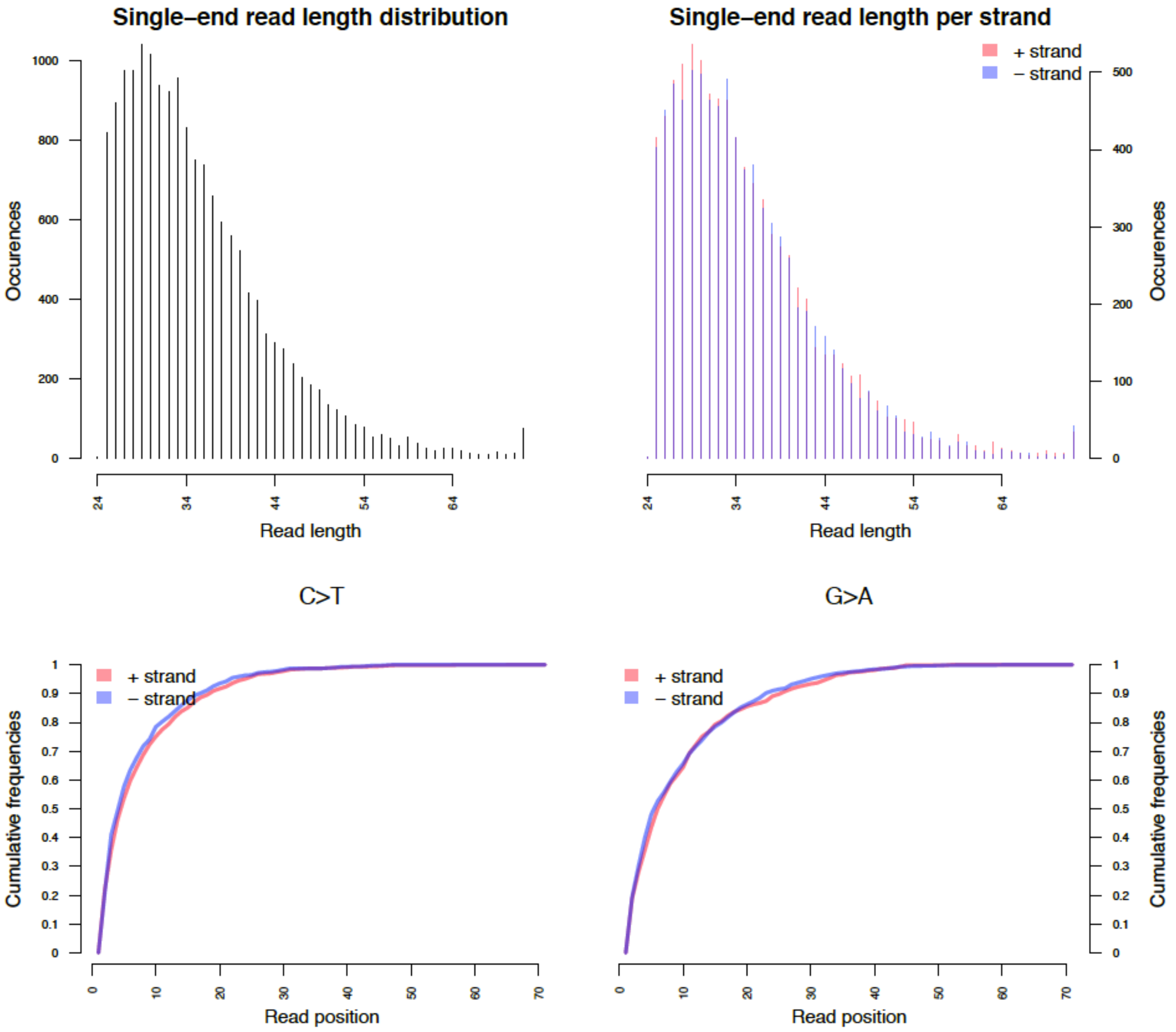

Figure S2. mapDamage fragment length distribution of the 53,550 shotgun cleaned Illumina reads mapping to the *Mylodon darwinii* SP57 mitogenome. Mean fragment length was 30 base pairs.

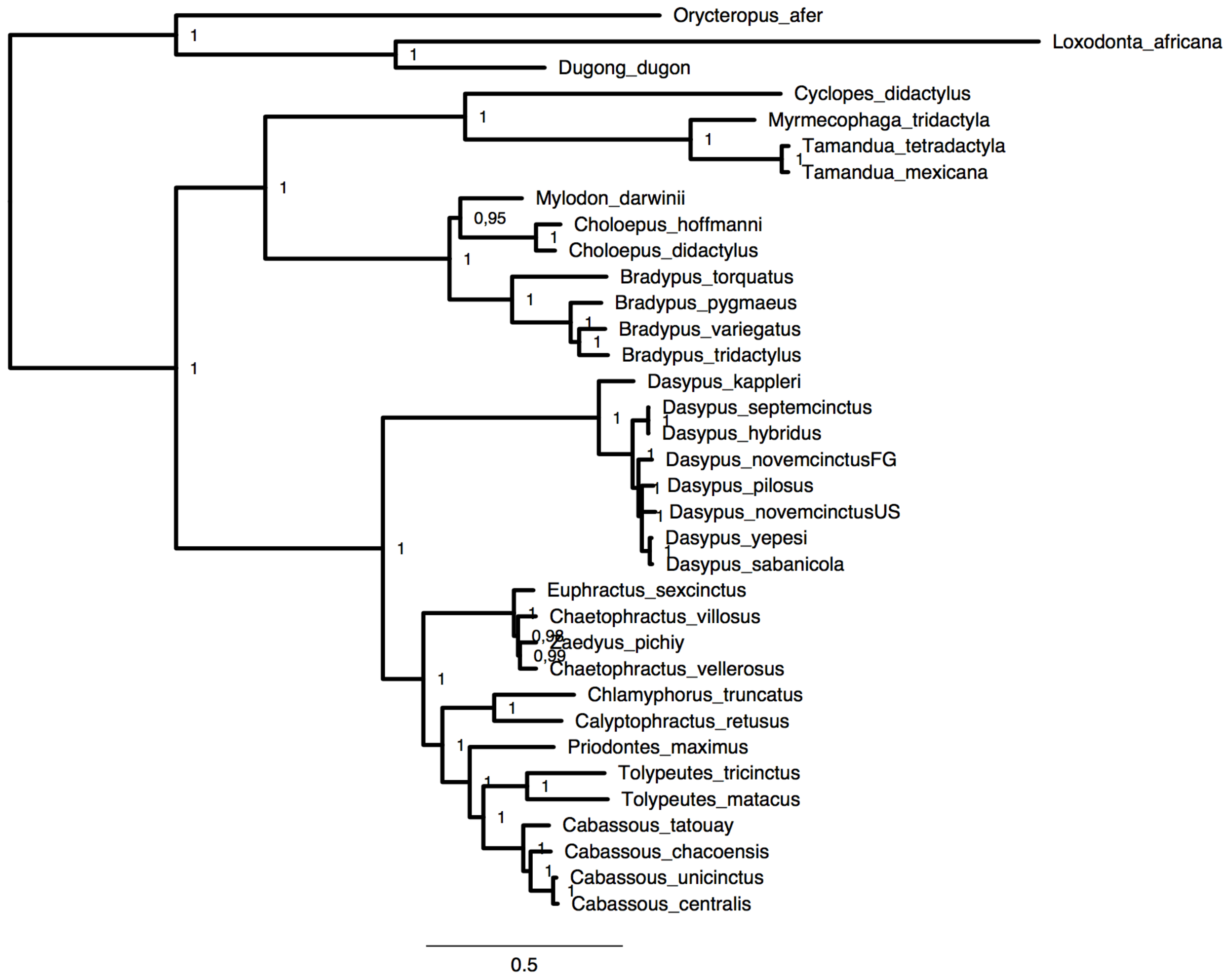

Figure S3: Bayesian consensus mitogenomic tree obtained with PhyloBayes under the CAT-GTR+G_4 mixture model. Bayesian posterior probabilities are indicated at nodes. Branch lengths represent mean number of substitutions per site.

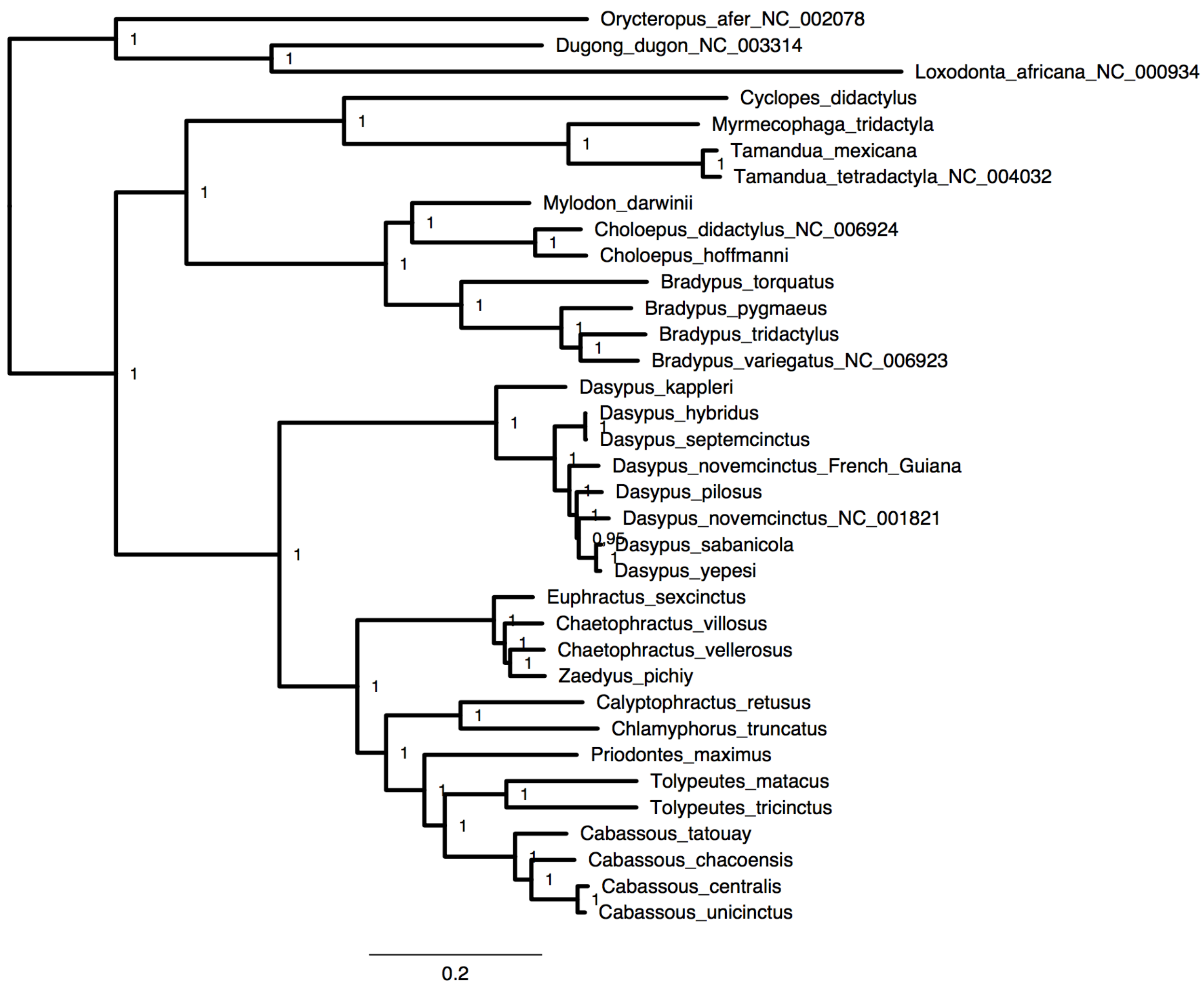

Figure S4: Bayesian consensus mitogenomic tree obtained with MrBayes under a mixed model for each best-fitting partition as estimated by PartitionFinder. Bayesian posterior probabilities are indicated at nodes. Branch lengths represent mean number of substitutions per site.

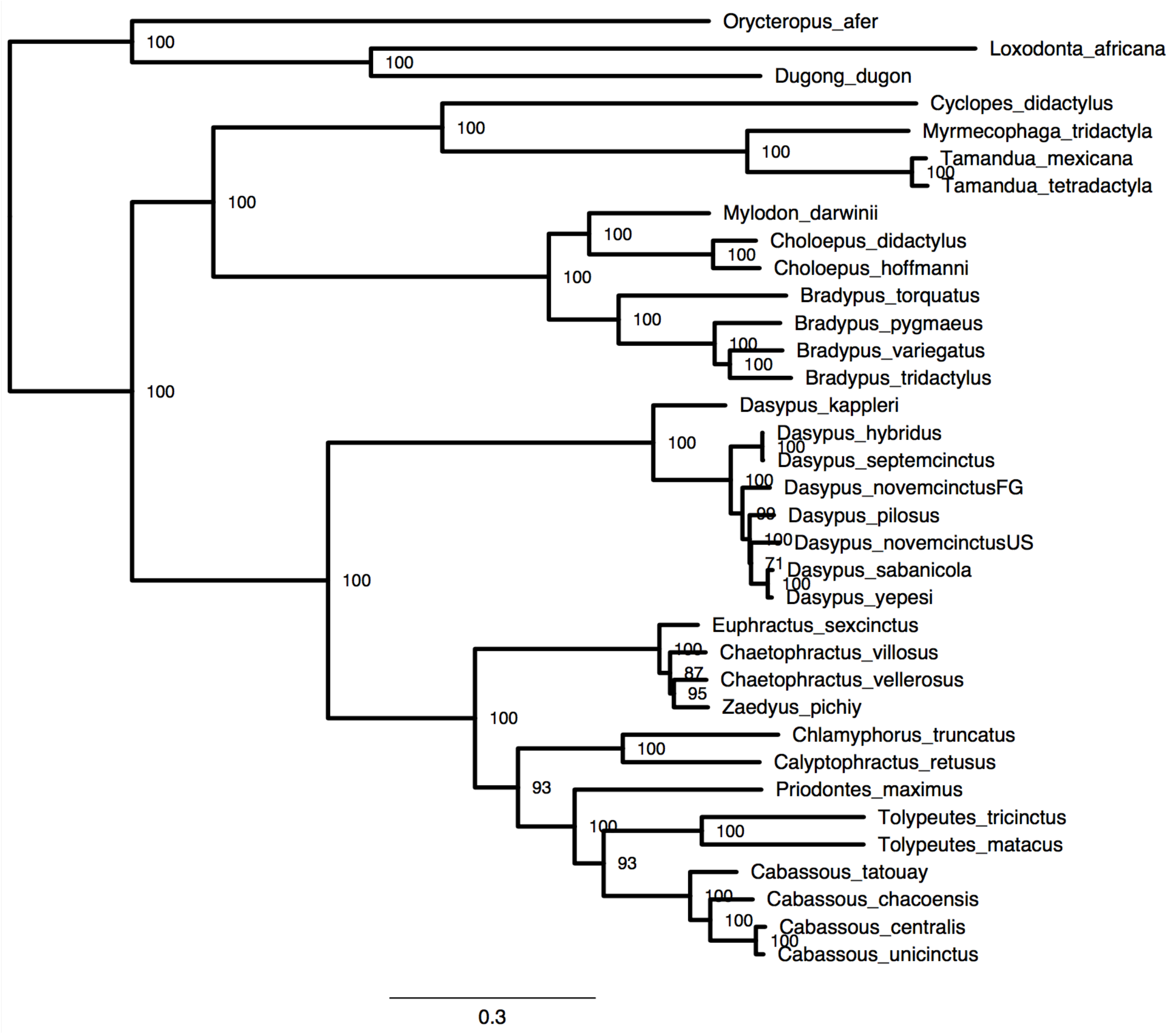

Figure S5: Maximum likelihood mitogenomic tree obtained with RAxML under a GTR+G+I mixed model for each best-fitting partition as estimated by PartitionFinder. Bootstrap percentages are indicated at nodes. Branch lengths represent mean number of substitutions per site.

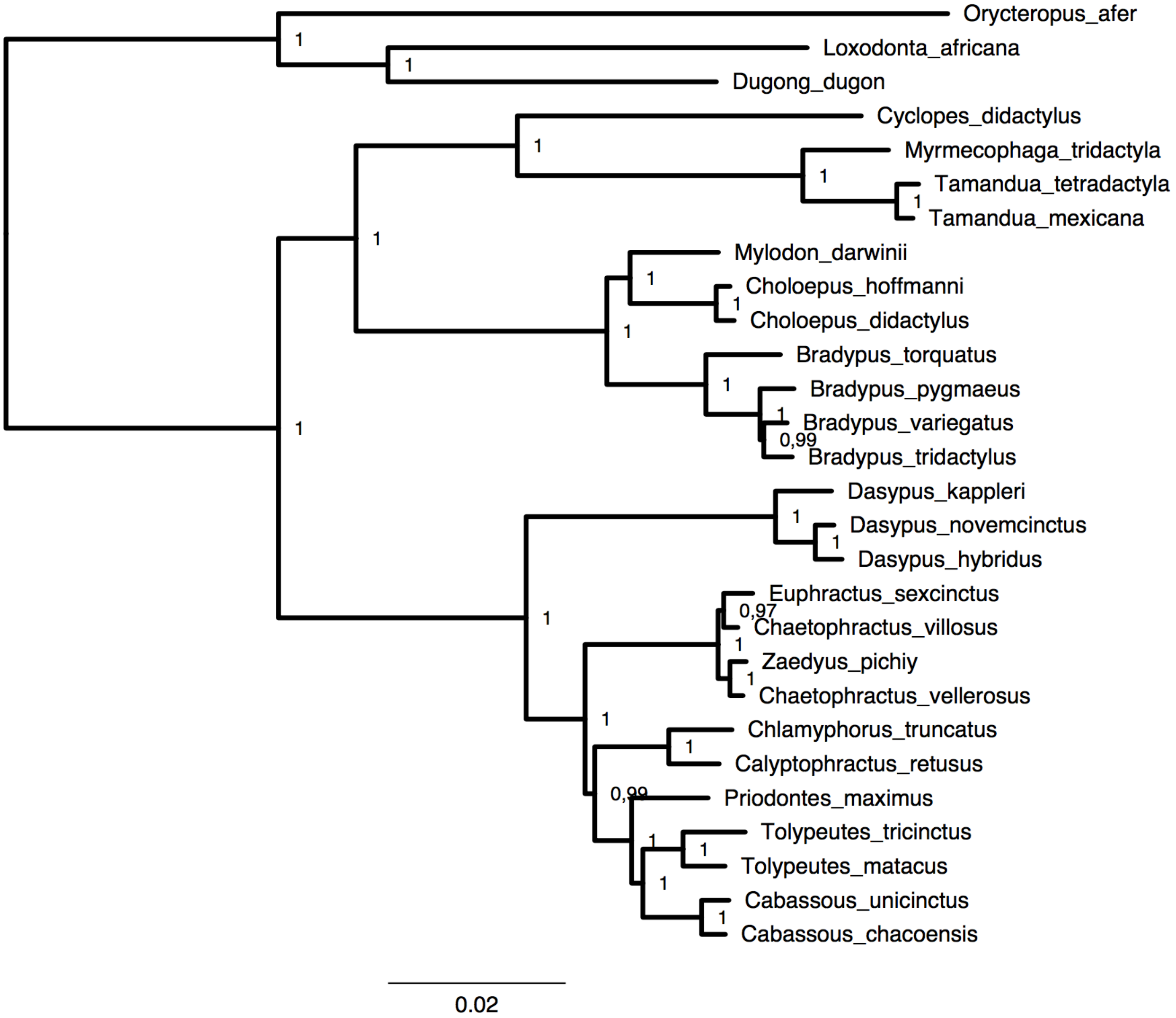

Figure S6: Bayesian consensus nuclear tree (concatenation of 7 exons) obtained with PhyloBayes under the CAT-GTR+G_4 mixture model. Bayesian posterior probabilities are indicated at nodes. Branch lengths represent mean number of substitutions per site.

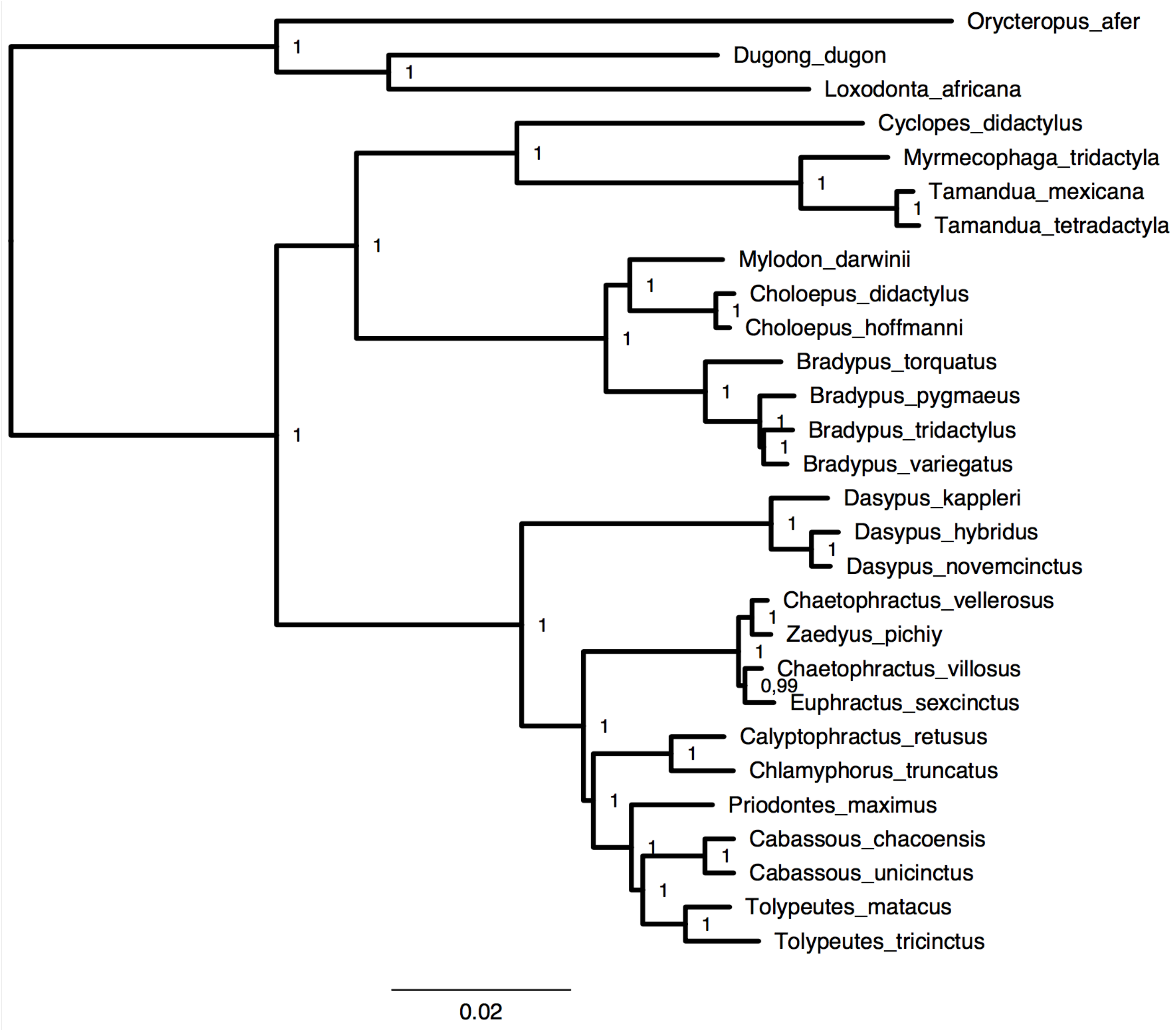

Figure S7: Bayesian consensus nuclear tree (concatenation of 7 exons) obtained with MrBayes under a mixed model for each best-fitting partition as estimated by PartitionFinder. Bayesian posterior probabilities are indicated at nodes. Branch lengths represent mean number of substitutions per site.

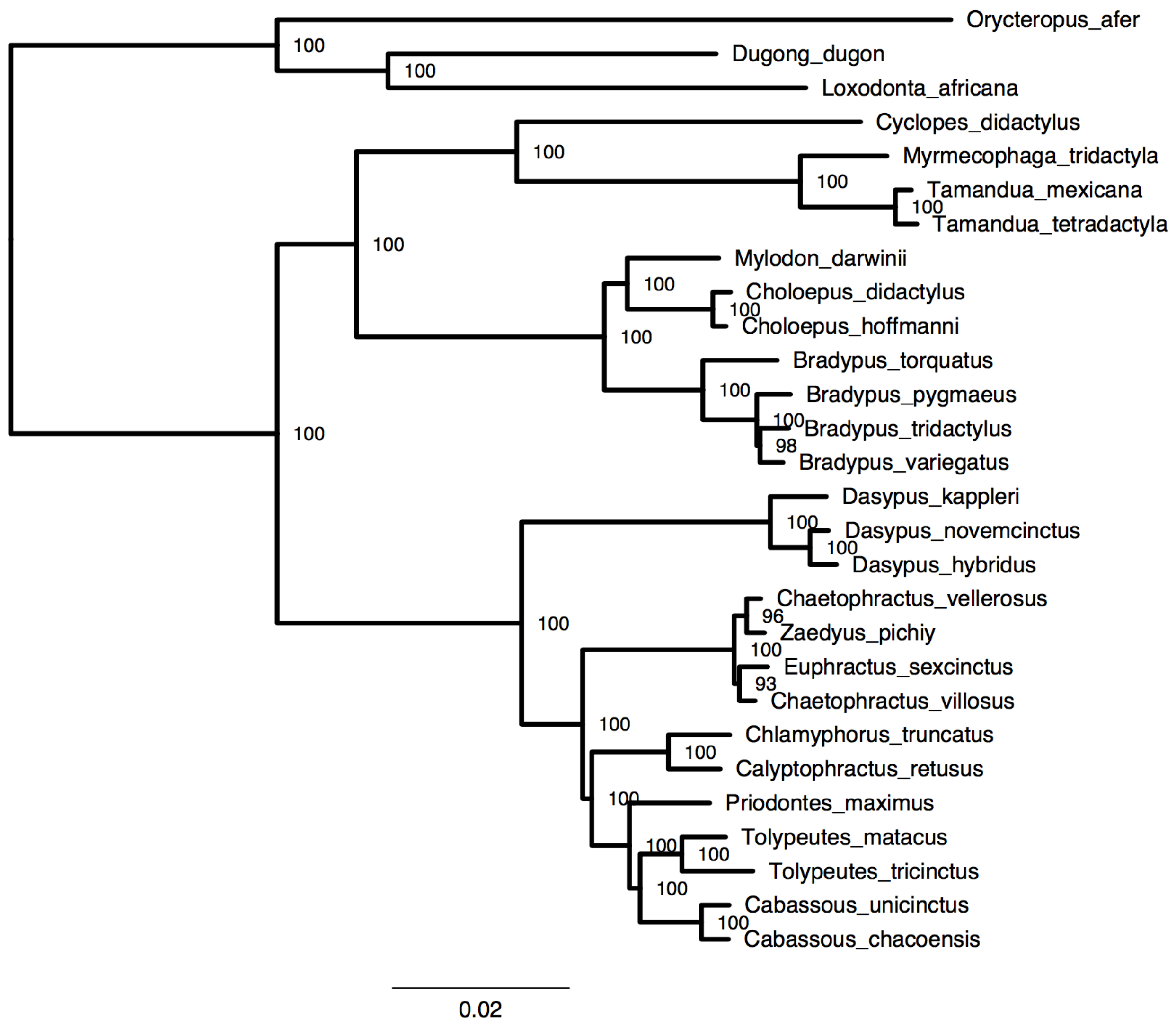

Figure S8: Maximum likelihood nuclear tree (concatenation of 7 exons) obtained with RAxML under a GTR+G mixed model for each best-fitting partition as estimated by PartitionFinder. Bootstrap percentages are indicated at nodes. Branch lengths represent mean number of substitutions per site.

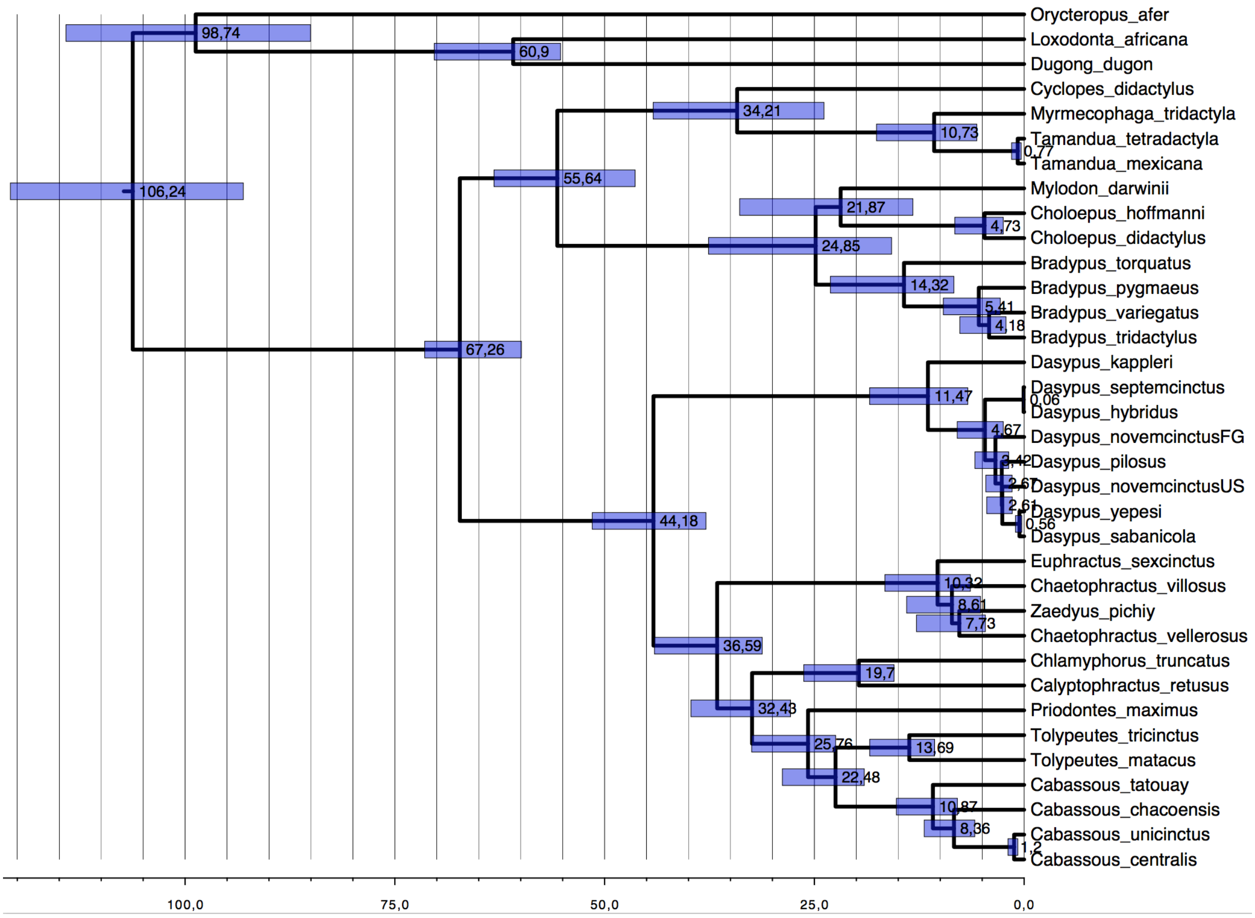

Figure S9: Bayesian mitogenomic chronogram obtained with PhyloBayes under the CAT-GTR+G_4 mixture model and an autocorrelated lognormal model of clock relaxation. Timescale is in million years ago.

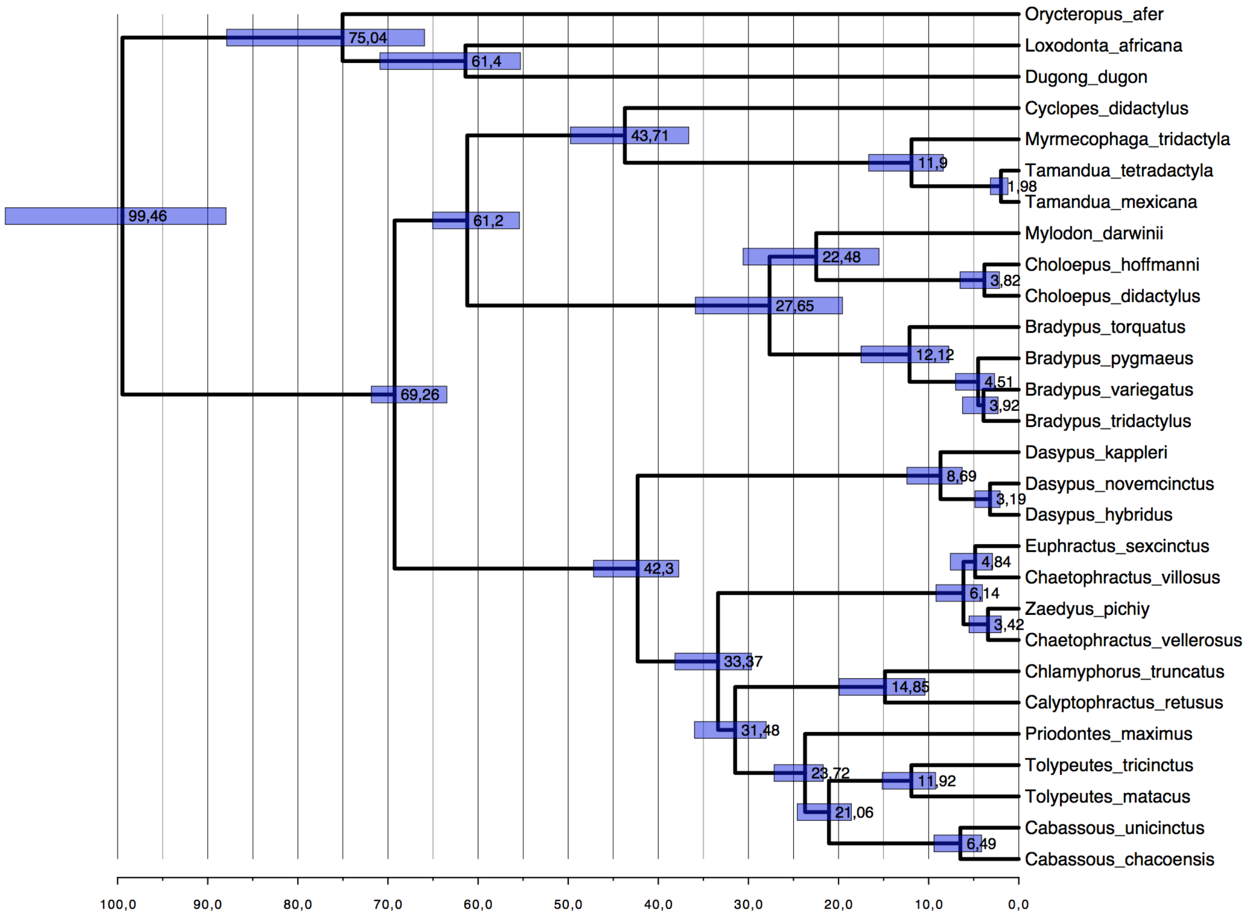

Figure S10: Bayesian nuclear chronogram (concatenation of 7 exons) obtained with PhyloBayes under the CAT-GTR+G_4 mixture model and an autocorrelated lognormal model of clock relaxation. Timescale is in million years ago.

Figure S11: Comparison of available *Mylodon* mitogenomes with the *Choloepus didactylus* reference mitogenome.

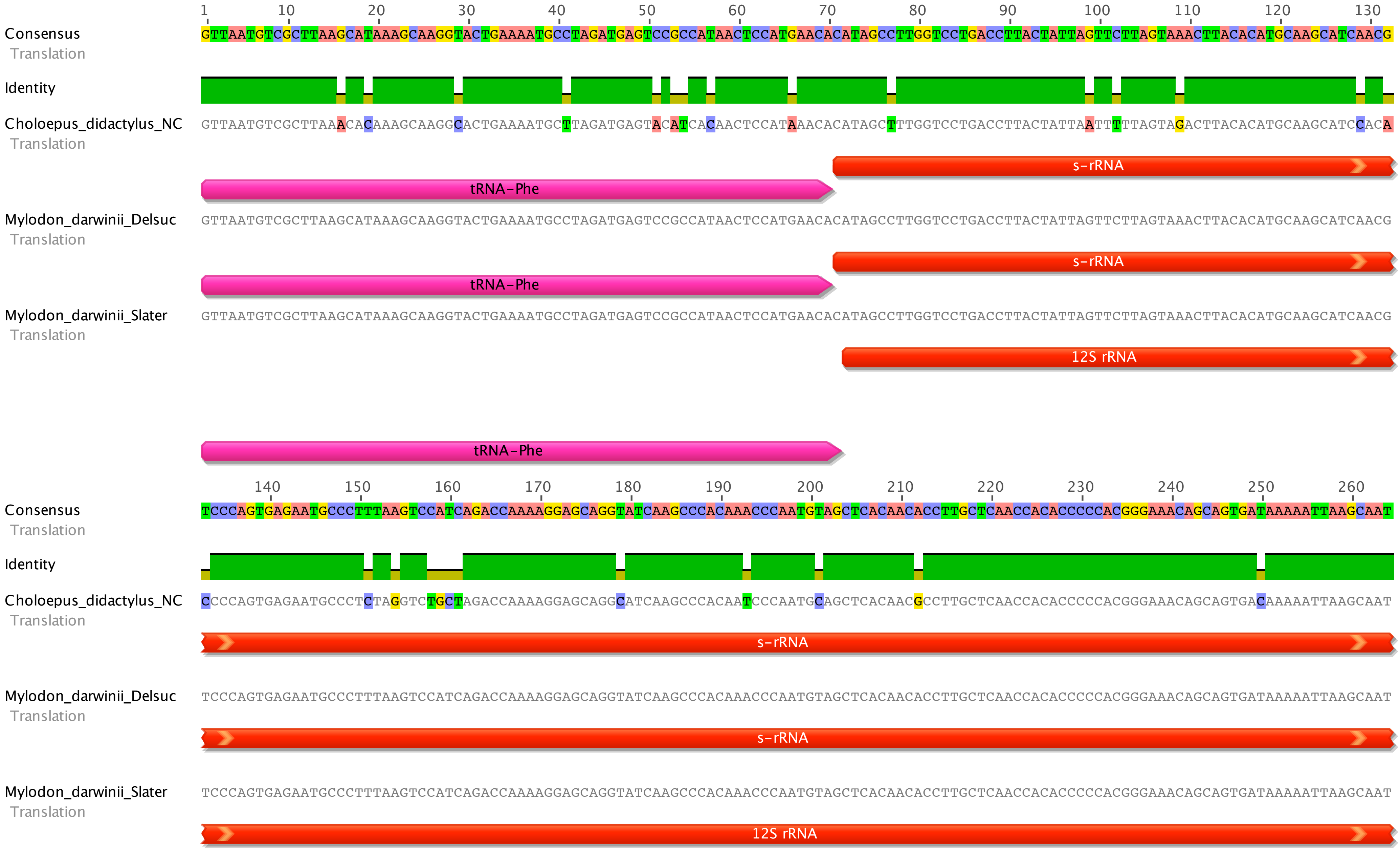
1 10 20 30 40 50 60 70 80 90 100 110 120 130
Consensus
Translation
GTTAATGTCGCTTAAGCATAAAGCAAGGTACTGAAAATGCCTAGATGAGTCCGCCATAACTCCATGAACACATAGCCTTGGTCCTGACCTTACTATTAGTTCTTAGTAAACTTACACATGCAAGCATCAACG
Identity
Choloepus_didactylus_NC
Translation
GTTAATGTCGCTTAAACACAAAGCAAGGCACTGAAAATGCTTAGATGAGTACATCACAACTCCATAAACACATAGCTTTGGTCCTGACCTTACTATTAATTTTTAGTAGACTTACACATGCAAGCATCCACA
s-rRNA
tRNA-Phe
Mylodon_darwinii_Delsuc
Translation
GTTAATGTCGCTTAAGCATAAAGCAAGGTACTGAAAATGCCTAGATGAGTCCGCCATAACTCCATGAACACATAGCCTTGGTCCTGACCTTACTATTAGTTCTTAGTAAACTTACACATGCAAGCATCAACG
s-rRNA
tRNA-Phe
Mylodon_darwinii_Slater
Translation
GTTAATGTCGCTTAAGCATAAAGCAAGGTACTGAAAATGCCTAGATGAGTCCGCCATAACTCCATGAACACATAGCCTTGGTCCTGACCTTACTATTAGTTCTTAGTAAACTTACACATGCAAGCATCAACG
12S rRNA
tRNA-Phe
140 150 160 170 180 190 200 210 220 230 240 250 260
Consensus
Translation
TCCCAGTGAGAATGCCCTTTAAGTCCATCAGACCAAAAGGAGCAGGTATCAAGCCCACAAACCCAATGTAGCTCACAACACCTTGCTCAACCACACCCCCACGGGAAACAGCAGTGATAAAAATTAAGCAAT
Identity
Choloepus_didactylus_NC
Translation
CCCCAGTGAGAATGCCCTCTAGGTCTGCTAGACCAAAAGGAGCAGGCATCAAGCCCACAATCCCAATGCAGCTCACAACGCCTTGCTCAACCACACCCCCACGGGAAACAGCAGTGACAAAAATTAAGCAAT
s-rRNA
Mylodon_darwinii_Delsuc
Translation
TCCCAGTGAGAATGCCCTTTAAGTCCATCAGACCAAAAGGAGCAGGTATCAAGCCCACAAACCCAATGTAGCTCACAACACCTTGCTCAACCACACCCCCACGGGAAACAGCAGTGATAAAAATTAAGCAAT
s-rRNA
Mylodon_darwinii_Slater
Translation
TCCCAGTGAGAATGCCCTTTAAGTCCATCAGACCAAAAGGAGCAGGTATCAAGCCCACAAACCCAATGTAGCTCACAACACCTTGCTCAACCACACCCCCACGGGAAACAGCAGTGATAAAAATTAAGCAAT
12S rRNA

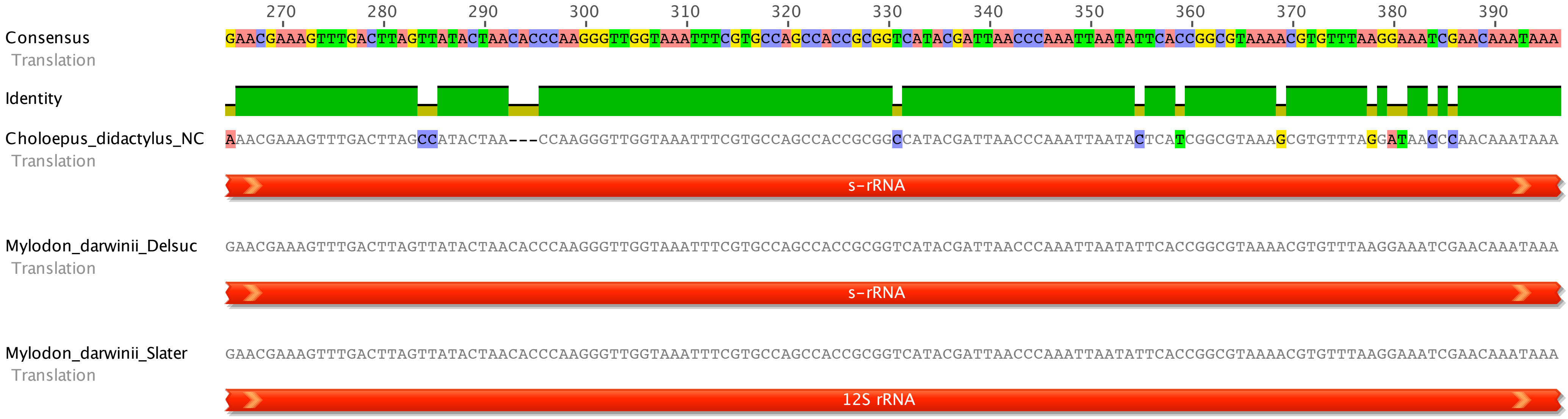

270 280 290 300 310 320 330 340 350 360 370 380 390
Consensus GAACGAAAGTTTGACTTAGTTATACTAACACCCAAGGGTTGGTAAATTTCGTGCCAGCCACCGCGGTCATACGATTAACCCAAATTAATATTCACCGGCGTAAAACGTGTTTAAGGAAATCGAACAAATAAA
Translation
Identity
Choloepus_didactylus_NC AAACGAAAGTTTGACTTAGCCATACTAA---CCAAGGGTTGGTAAATTTCGTGCCAGCCACCGCGGCCATACGATTAACCCAAATTAATACTCATCGGCGTAAAGCGTGTTTAGGATAACCCAACAAATAAA
Translation
s-rRNA
Mylodon_darwinii_Delsuc GAACGAAAGTTTGACTTAGTTATACTAACACCCAAGGGTTGGTAAATTTCGTGCCAGCCACCGCGGTCATACGATTAACCCAAATTAATATTCACCGGCGTAAAACGTGTTTAAGGAAATCGAACAAATAAA
Translation
s-rRNA
Mylodon_darwinii_Slater GAACGAAAGTTTGACTTAGTTATACTAACACCCAAGGGTTGGTAAATTTCGTGCCAGCCACCGCGGTCATACGATTAACCCAAATTAATATTCACCGGCGTAAAACGTGTTTAAGGAAATCGAACAAATAAA
Translation
12S rRNA

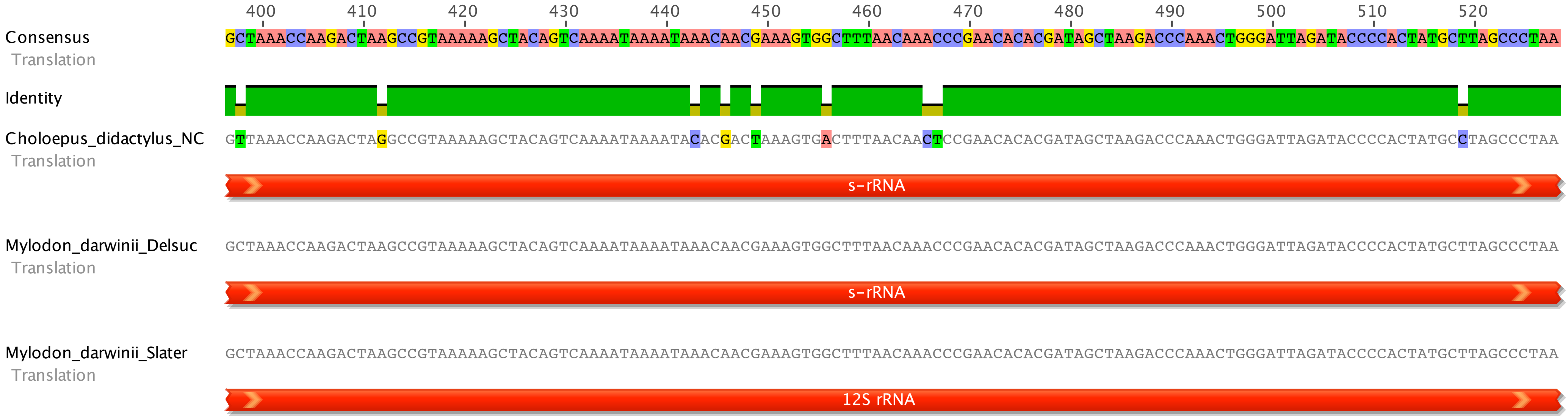

400 410 420 430 440 450 460 470 480 490 500 510 520
Consensus GCTAAACCAAGACTAAGCCGTAAAAAGCTACAGTCAAAATAAAATAAACAACGAAAGTGGCTTTAACAAACCCGAACACACGATAGCTAAGACCCAAACTGGGATTAGATACCCCACTATGCTTAGCCCTAA
Translation
Identity
Choloepus_didactylus_NC GTTAAACCAAGACTAGGCCGTAAAAAGCTACAGTCAAAATAAAATACACGACTAAAGTGACTTTAACAACTCCGAACACACGATAGCTAAGACCCAAACTGGGATTAGATACCCCACTATGCCTAGCCCTAA
Translation
s-rRNA
Mylodon_darwinii_Delsuc GCTAAACCAAGACTAAGCCGTAAAAAGCTACAGTCAAAATAAAATAAACAACGAAAGTGGCTTTAACAAACCCGAACACACGATAGCTAAGACCCAAACTGGGATTAGATACCCCACTATGCTTAGCCCTAA
Translation
s-rRNA
Mylodon_darwinii_Slater GCTAAACCAAGACTAAGCCGTAAAAAGCTACAGTCAAAATAAAATAAACAACGAAAGTGGCTTTAACAAACCCGAACACACGATAGCTAAGACCCAAACTGGGATTAGATACCCCACTATGCTTAGCCCTAA
Translation
12S rRNA

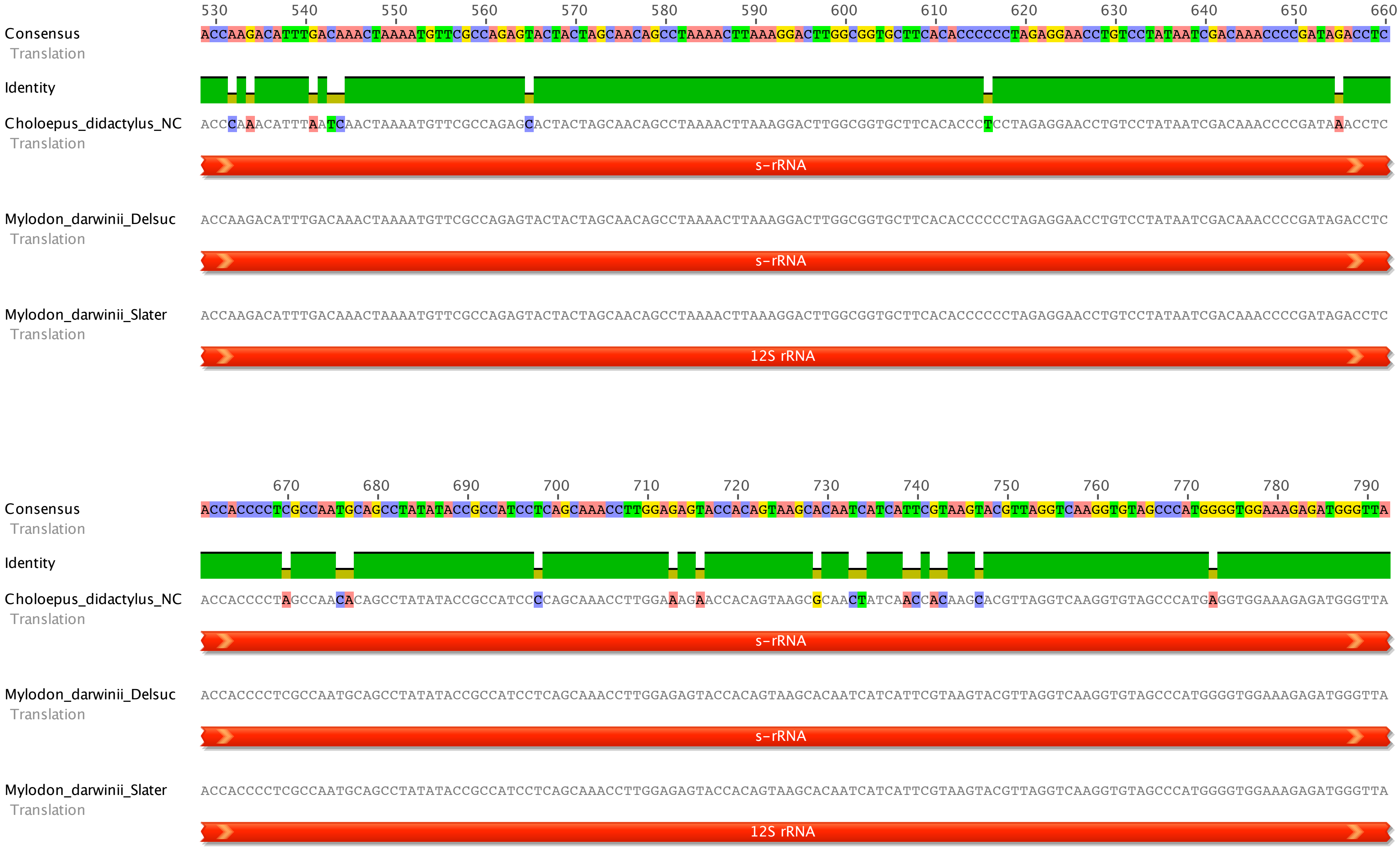
530 540 550 560 570 580 590 600 610 620 630 640 650 660
Consensus
Translation
Identity
Choloepus_didactylus_NC
Translation
s-rRNA
Mylodon_darwinii_Delsuc
Translation
s-rRNA
Mylodon_darwinii_Slater
Translation
12S rRNA
670 680 690 700 710 720 730 740 750 760 770 780 790
Consensus
Translation
Identity
Choloepus_didactylus_NC
Translation
s-rRNA
Mylodon_darwinii_Delsuc
Translation
s-rRNA
Mylodon_darwinii_Slater
Translation
12S rRNA

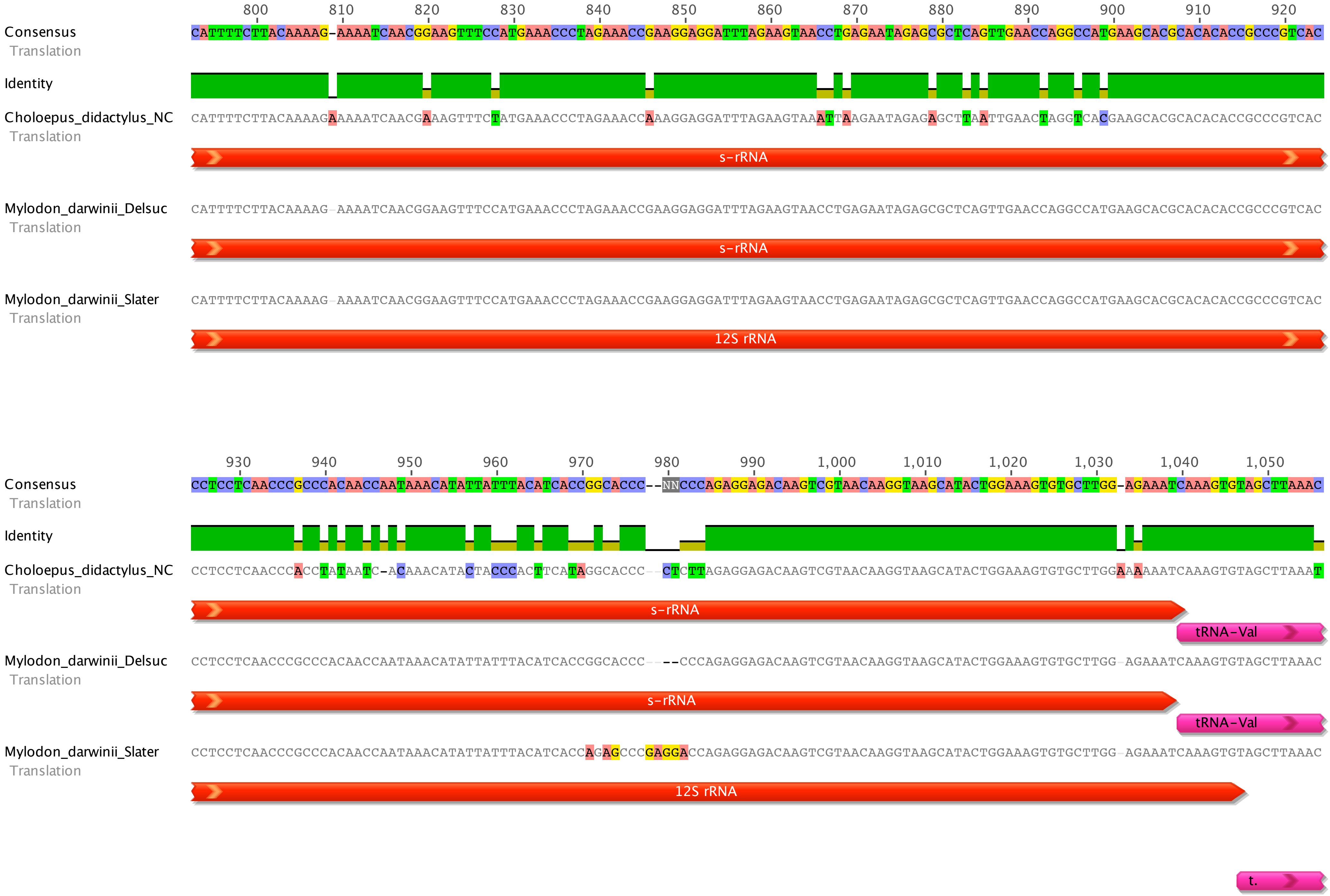

Consensus
Translation
Identity
Choloepus_didactylus_NC
Translation
s-rRNA
Mylodon_darwinii_Delsuc
Translation
s-rRNA
Mylodon_darwinii_Slater
Translation
12S rRNA
Consensus
Translation
Identity
Choloepus_didactylus_NC
Translation
s-rRNA
tRNA-Val
Mylodon_darwinii_Delsuc
Translation
s-rRNA
tRNA-Val
Mylodon_darwinii_Slater
Translation
12S rRNA
t.

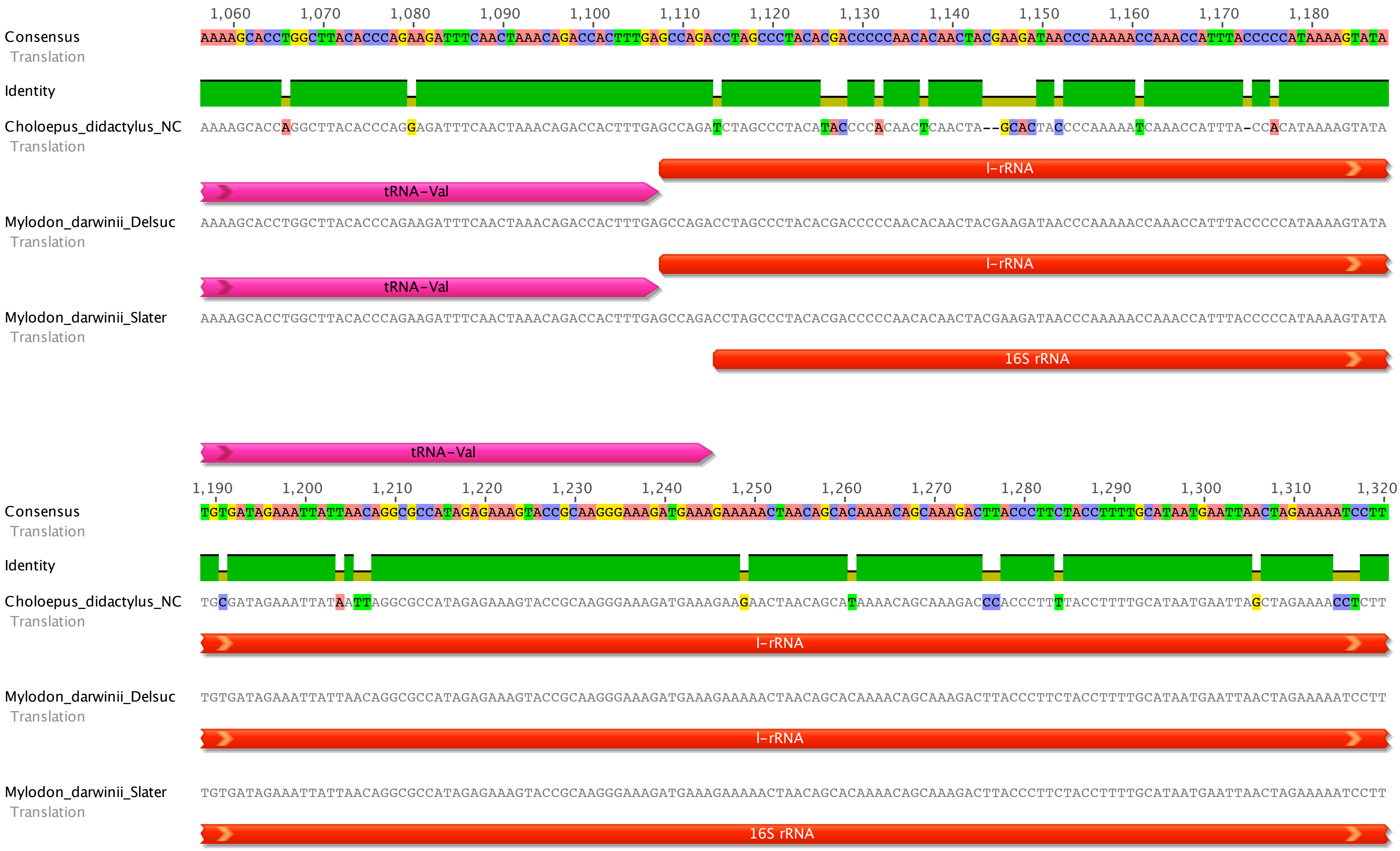

1,060 1,070 1,080 1,090 1,100 1,110 1,120 1,130 1,140 1,150 1,160 1,170 1,180
Consensus
Translation
Identity
Choloepus_didactylus_NC
AAAAGCACCAGGCTTACACCCAGGAGATTTCAACTAAACAGACCACTTTGAGCCAGATCTAGCCCTACATACCCCACAACTCAACTA--GCACTACCCCAAAAATCAAACCATTTA-CCACATAAAAGTATA
Translation
l-rRNA
tRNA-Val
Mylodon_darwinii_Delsuc
AAAAGCACCTGGCTTACACCCAGAAGATTTCAACTAAACAGACCACTTTGAGCCAGACCTAGCCCTACACGACCCCCAACACAACTACGAAGATAACCCAAAAACCAAACCATTTACCCCCATAAAAGTATA
Translation
l-rRNA
tRNA-Val
Mylodon_darwinii_Slater
AAAAGCACCTGGCTTACACCCAGAAGATTTCAACTAAACAGACCACTTTGAGCCAGACCTAGCCCTACACGACCCCCAACACAACTACGAAGATAACCCAAAAACCAAACCATTTACCCCCATAAAAGTATA
Translation
16S rRNA
tRNA-Val
1,190 1,200 1,210 1,220 1,230 1,240 1,250 1,260 1,270 1,280 1,290 1,300 1,310 1,320
Consensus
Translation
Identity
Choloepus_didactylus_NC
TGCGATAGAAATTATAATTAGGCGCCATAGAGAAAGTACCGCAAGGGAAAGATGAAAGAAGAACTAACAGCATAAAACAGCAAAGACCCACCCTTTTACCTTTTGCATAATGAATTAGCTAGAAAACCTCTT
Translation
l-rRNA
Mylodon_darwinii_Delsuc
TGTGATAGAAATTATTAACAGGCGCCATAGAGAAAGTACCGCAAGGGAAAGATGAAAGAAAAACTAACAGCACAAAACAGCAAAGACTTACCCTTCTACCTTTTGCATAATGAATTAACTAGAAAAATCCTT
Translation
l-rRNA
Mylodon_darwinii_Slater
TGTGATAGAAATTATTAACAGGCGCCATAGAGAAAGTACCGCAAGGGAAAGATGAAAGAAAAACTAACAGCACAAAACAGCAAAGACTTACCCTTCTACCTTTTGCATAATGAATTAACTAGAAAAATCCTT
Translation
16S rRNA

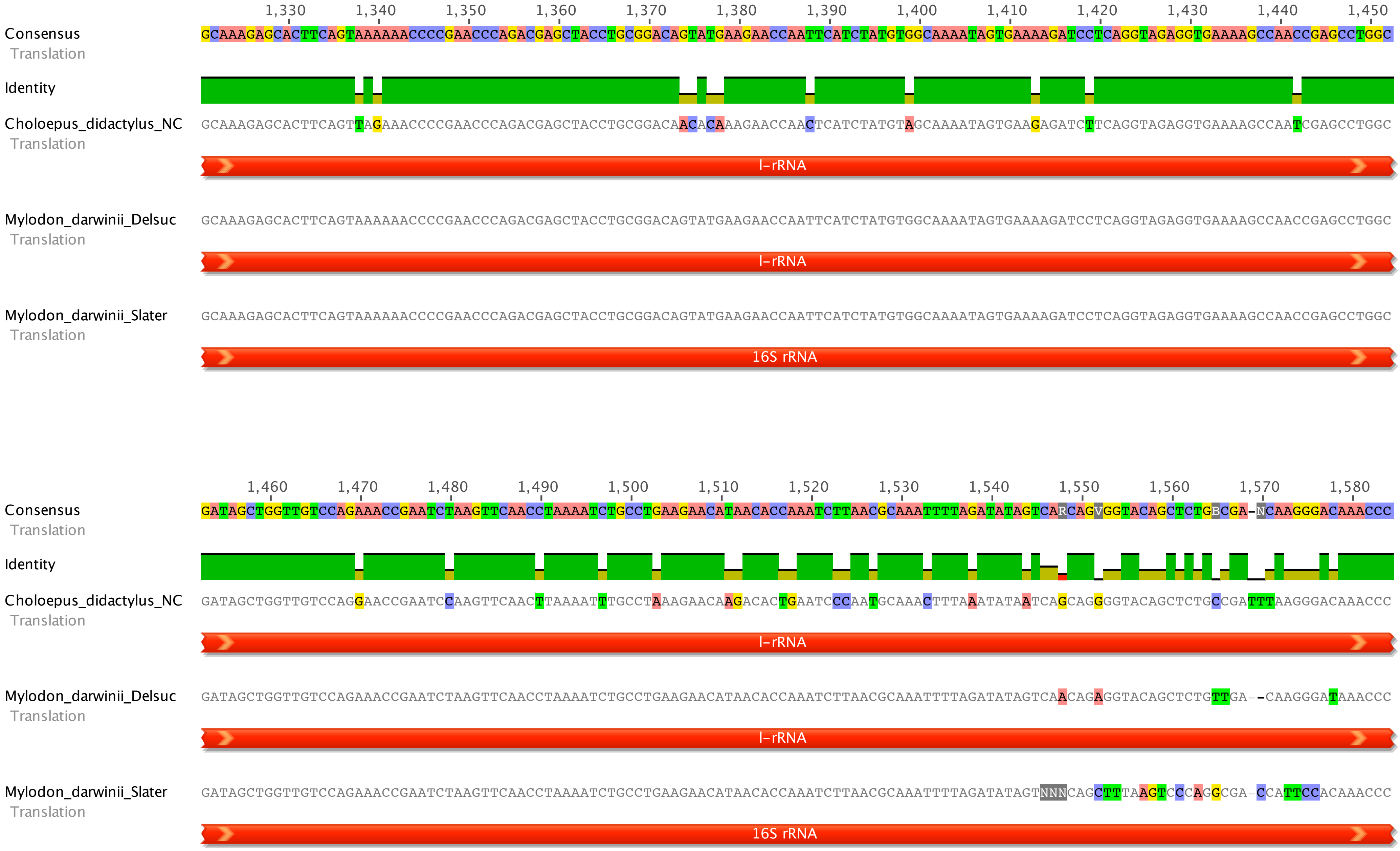
1,330 1,340 1,350 1,360 1,370 1,380 1,390 1,400 1,410 1,420 1,430 1,440 1,450
Consensus GCAAAGAGCACTTCAGTAAAAAACCCCGAACCCAGACGAGCTACCTGCGGACAGTATGAAGAACCAATTCATCTATGTGGCAAAATAGTGAAAAGATCCTCAGGTAGAGGTGAAAAGCCAACCGAGCCTGGC
Translation
Identity
Choloepus_didactylus_NC GCAAAGAGCACTTCAGTTAGAAACCCCGAACCCAGACGAGCTACCTGCGGACAACACAAAGAACCAACTCATCTATGTAGCAAAATAGTGAAGAGATCTTCAGGTAGAGGTGAAAAGCCAATCGAGCCTGGC
Translation
l-rRNA
Mylodon_darwinii_Delsuc GCAAAGAGCACTTCAGTAAAAAACCCCGAACCCAGACGAGCTACCTGCGGACAGTATGAAGAACCAATTCATCTATGTGGCAAAATAGTGAAAAGATCCTCAGGTAGAGGTGAAAAGCCAACCGAGCCTGGC
Translation
l-rRNA
Mylodon_darwinii_Slater GCAAAGAGCACTTCAGTAAAAAACCCCGAACCCAGACGAGCTACCTGCGGACAGTATGAAGAACCAATTCATCTATGTGGCAAAATAGTGAAAAGATCCTCAGGTAGAGGTGAAAAGCCAACCGAGCCTGGC
Translation
16S rRNA
1,460 1,470 1,480 1,490 1,500 1,510 1,520 1,530 1,540 1,550 1,560 1,570 1,580
Consensus GATAGCTGGTTGTCCAGAAACCGAATCTAAGTTCAACCTAAAATCTGCCTGAAGAACATAACACCAAATCTTAACGCAAATTTTAGATATAGTCARCAGVGGTACAGCTCTGBCGA-NCAAGGGACAAACCC
Translation
Identity
Choloepus_didactylus_NC GATAGCTGGTTGTCCAGGAACCGAATCCAAGTTCAACTTAAAATTTGCCTAAAGAACAAGACACTGAATCCCAATGCAAACTTTAAATATAATCAGCAGGGGTACAGCTCTGCCGATTTAAGGGACAAACCC
Translation
l-rRNA
Mylodon_darwinii_Delsuc GATAGCTGGTTGTCCAGAAACCGAATCTAAGTTCAACCTAAAATCTGCCTGAAGAACATAACACCAAATCTTAACGCAAATTTTAGATATAGTCAACAGAGGTACAGCTCTGTTGA -CAAGGGATAAACCC
Translation
l-rRNA
Mylodon_darwinii_Slater GATAGCTGGTTGTCCAGAAACCGAATCTAAGTTCAACCTAAAATCTGCCTGAAGAACATAACACCAAATCTTAACGCAAATTTTAGATATAGTNNNCAGCTTTAAGTCCCAGGCGA CCATTCCACAAACCC
Translation
16S rRNA

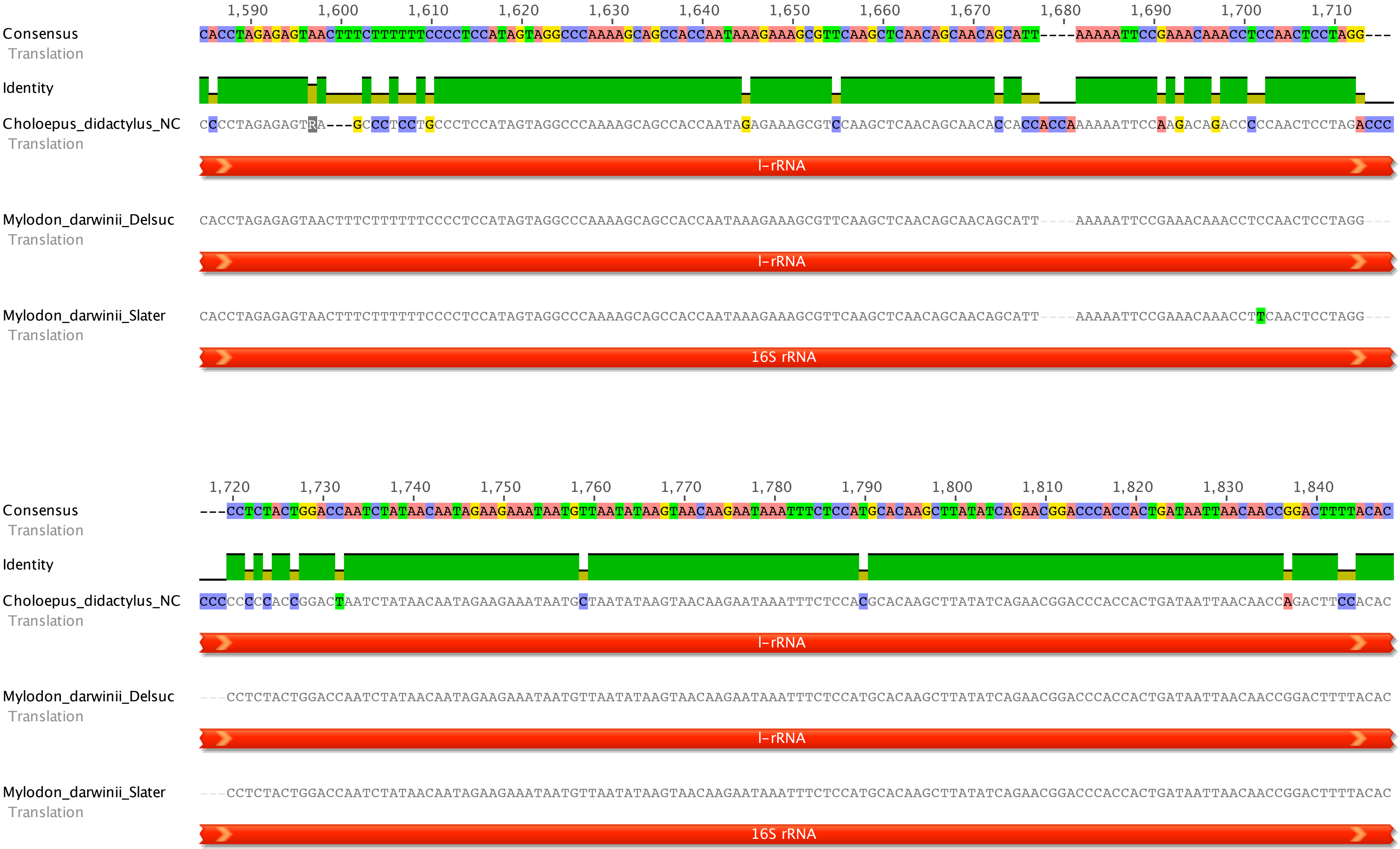
1,590 1,600 1,610 1,620 1,630 1,640 1,650 1,660 1,670 1,680 1,690 1,700 1,710
Consensus
CACCTAGAGAGTAACTTTCTTTTTTCCCCTCCATAGTAGGCCCAAAAGCAGCCACCAATAAAGAAAGCGTTCAAGCTCAACAGCAACAGCATT----AAAAATTCCGAAACAAACCTCCAACTCCTAGG---
Translation
Identity
Choloepus_didactylus_NC
CCCCTAGAGAGTRA---GCCCTCCTGCCCTCCATAGTAGGCCCAAAAGCAGCCACCAATAGAGAAAGCGTCCAAGCTCAACAGCAACACCACCACCAAAAAATTCCAAGACAGACCCCCAACTCCTAGACCC
Translation
l-rRNA
Mylodon_darwinii_Delsuc
CACCTAGAGAGTAACTTTCTTTTTTCCCCTCCATAGTAGGCCCAAAAGCAGCCACCAATAAAGAAAGCGTTCAAGCTCAACAGCAACAGCATT----AAAAATTCCGAAACAAACCTCCAACTCCTAGG---
Translation
l-rRNA
Mylodon_darwinii_Slater
CACCTAGAGAGTAACTTTCTTTTTTCCCCTCCATAGTAGGCCCAAAAGCAGCCACCAATAAAGAAAGCGTTCAAGCTCAACAGCAACAGCATT----AAAAATTCCGAAACAAACCTTCAACTCCTAGG---
Translation
16S rRNA
1,720 1,730 1,740 1,750 1,760 1,770 1,780 1,790 1,800 1,810 1,820 1,830 1,840
Consensus
---CCTCTACTGGACCAATCTATAACAATAGAAGAAATAATGTTAATATAAGTAACAAGAATAAATTTCTCCATGCACAAGCTTATATCAGAACGGACCCACCACTGATAATTAACAACCGGACTTTTACAC
Translation
Identity
Choloepus_didactylus_NC
CCCCCCCCACCGGACTAATCTATAACAATAGAAGAAATAATGCTAATATAAGTAACAAGAATAAATTTCTCCACGCACAAGCTTATATCAGAACGGACCCACCACTGATAATTAACAACCAGACTTCCACAC
Translation
l-rRNA
Mylodon_darwinii_Delsuc
---CCTCTACTGGACCAATCTATAACAATAGAAGAAATAATGTTAATATAAGTAACAAGAATAAATTTCTCCATGCACAAGCTTATATCAGAACGGACCCACCACTGATAATTAACAACCGGACTTTTACAC
Translation
l-rRNA
Mylodon_darwinii_Slater
---CCTCTACTGGACCAATCTATAACAATAGAAGAAATAATGTTAATATAAGTAACAAGAATAAATTTCTCCATGCACAAGCTTATATCAGAACGGACCCACCACTGATAATTAACAACCGGACTTTTACAC
Translation
16S rRNA

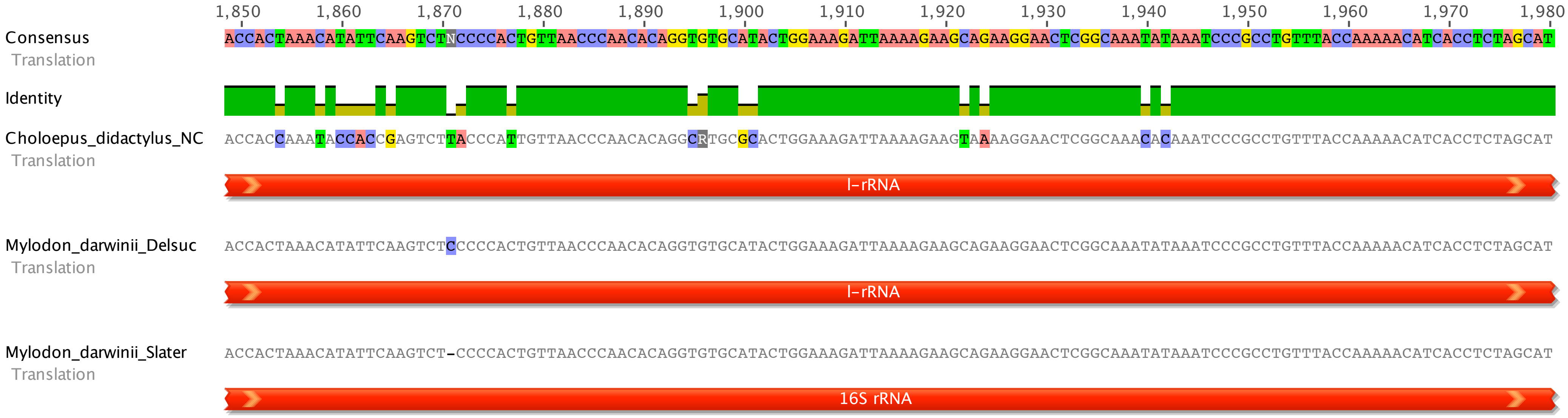
1,850 1,860 1,870 1,880 1,890 1,900 1,910 1,920 1,930 1,940 1,950 1,960 1,970 1,980
Consensus
ACCACTAAACATATTCAAGTCTNCCCCACTGTTAACCCAACACAGGTGTGCATACTGGAAAGATTAAAAGAAGCAGAAGGAACTCGGCAAATATAAATCCCGCCTGTTTACCAAAAACATCACCTCTAGCAT
Translation
Identity
Choloepus_didactylus_NC
ACCACCAAATACCACCGAGTCTTACCCATTGTTAACCCAACACAGGCRTGCGCACTGGAAAGATTAAAAGAAGTAAAAGGAACTCGGCAAACACAAATCCCGCCTGTTTACCAAAAACATCACCTCTAGCAT
Translation
l-rRNA
Mylodon_darwinii_Delsuc
ACCACTAAACATATTCAAGTCTCCCCCACTGTTAACCCAACACAGGTGTGCATACTGGAAAGATTAAAAGAAGCAGAAGGAACTCGGCAAATATAAATCCCGCCTGTTTACCAAAAACATCACCTCTAGCAT
Translation
l-rRNA
Mylodon_darwinii_Slater
ACCACTAAACATATTCAAGTCT-CCCCACTGTTAACCCAACACAGGTGTGCATACTGGAAAGATTAAAAGAAGCAGAAGGAACTCGGCAAATATAAATCCCGCCTGTTTACCAAAAACATCACCTCTAGCAT
Translation
16S rRNA

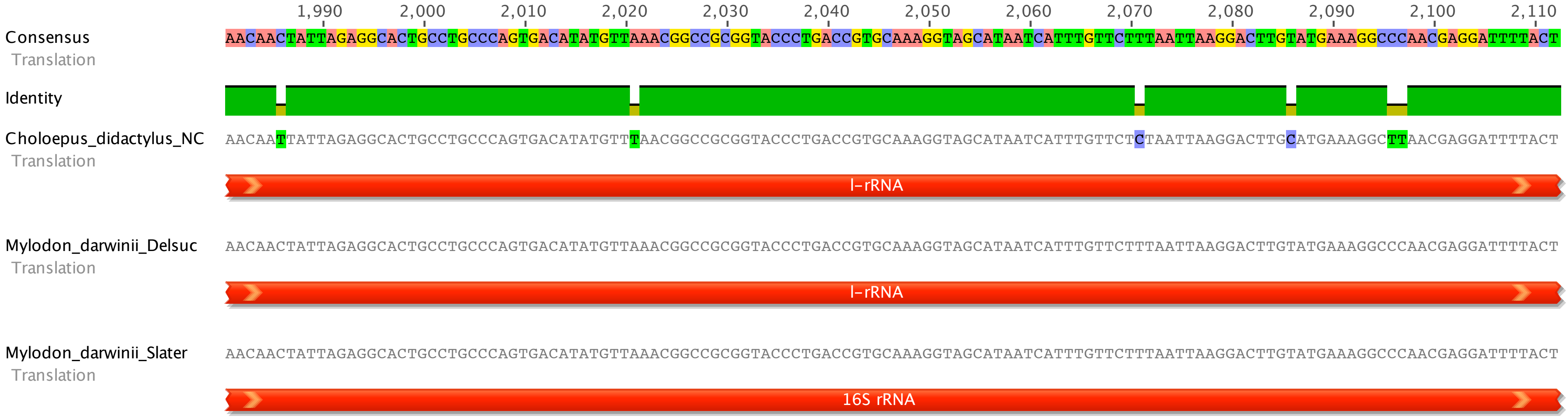
1,990 2,000 2,010 2,020 2,030 2,040 2,050 2,060 2,070 2,080 2,090 2,100 2,110
Consensus
AACAACTATTAGAGGCACTGCCTGCCCAGTGACATATGTTAAACGGCCGCGGTACCCTGACCGTGCAAAGGTAGCATAATCATTTGTTCTTTAATTAAGGACTTGTATGAAAGGCCCAACGAGGATTTTACT
Translation
Identity
Choloepus_didactylus_NC
AACAATTATTAGAGGCACTGCCTGCCCAGTGACATATGTTTAACGGCCGCGGTACCCTGACCGTGCAAAGGTAGCATAATCATTTGTTCTCTAATTAAGGACTTGCATGAAAGGCTTAACGAGGATTTTACT
Translation
l-rRNA
Mylodon_darwinii_Delsuc
AACAACTATTAGAGGCACTGCCTGCCCAGTGACATATGTTAAACGGCCGCGGTACCCTGACCGTGCAAAGGTAGCATAATCATTTGTTCTTTAATTAAGGACTTGTATGAAAGGCCCAACGAGGATTTTACT
Translation
l-rRNA
Mylodon_darwinii_Slater
AACAACTATTAGAGGCACTGCCTGCCCAGTGACATATGTTAAACGGCCGCGGTACCCTGACCGTGCAAAGGTAGCATAATCATTTGTTCTTTAATTAAGGACTTGTATGAAAGGCCCAACGAGGATTTTACT
Translation
16S rRNA

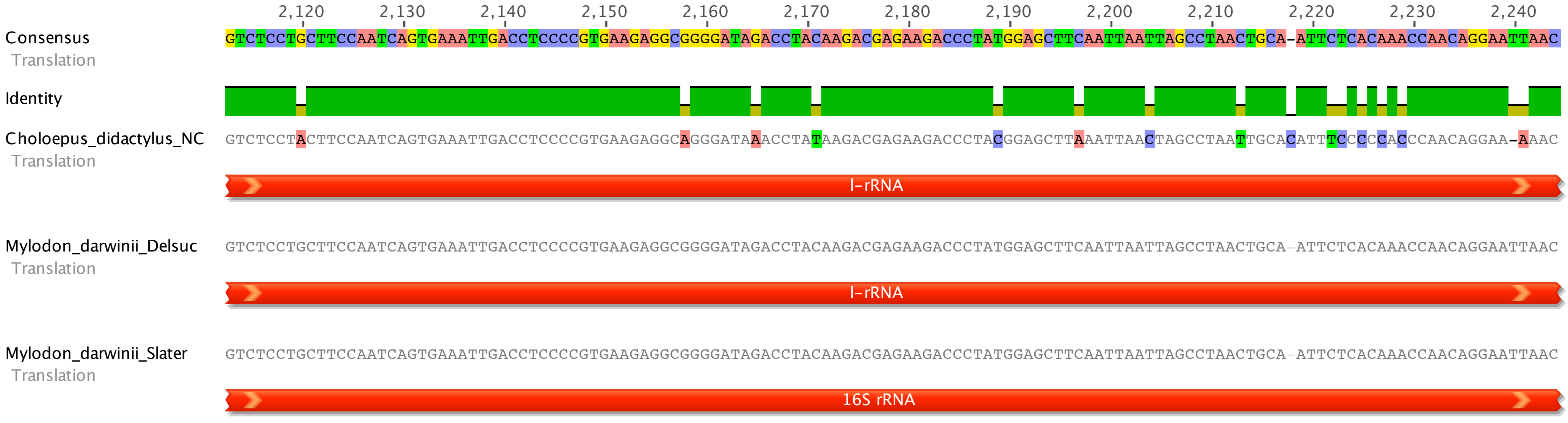
2,120 2,130 2,140 2,150 2,160 2,170 2,180 2,190 2,200 2,210 2,220 2,230 2,240
Consensus
GTCTCCTGCTTCCAATCAGTGAAATTGACCTCCCCGTGAAGAGGCGGGGATAGACCTACAAGACGAGAAGACCCTATGGAGCTTCAATTAATTAGCCTAACTGCA-ATTCTCACAAACCAACAGGAATTAAC
Translation
Identity
Choloepus_didactylus_NC
GTCTCCTACTTCCAATCAGTGAAATTGACCTCCCCGTGAAGAGGCAGGGATAAACCTATAAGACGAGAAGACCCTACGGAGCTTAAATTAACTAGCCTAATTGCACATTTCCCCCACCCAACAGGAA-AAAC
Translation
l-rRNA
Mylodon_darwinii_Delsuc
GTCTCCTGCTTCCAATCAGTGAAATTGACCTCCCCGTGAAGAGGCGGGGATAGACCTACAAGACGAGAAGACCCTATGGAGCTTCAATTAATTAGCCTAACTGCA-ATTCTCACAAACCAACAGGAATTAAC
Translation
l-rRNA
Mylodon_darwinii_Slater
GTCTCCTGCTTCCAATCAGTGAAATTGACCTCCCCGTGAAGAGGCGGGGATAGACCTACAAGACGAGAAGACCCTATGGAGCTTCAATTAATTAGCCTAACTGCA-ATTCTCACAAACCAACAGGAATTAAC
Translation
16S rRNA

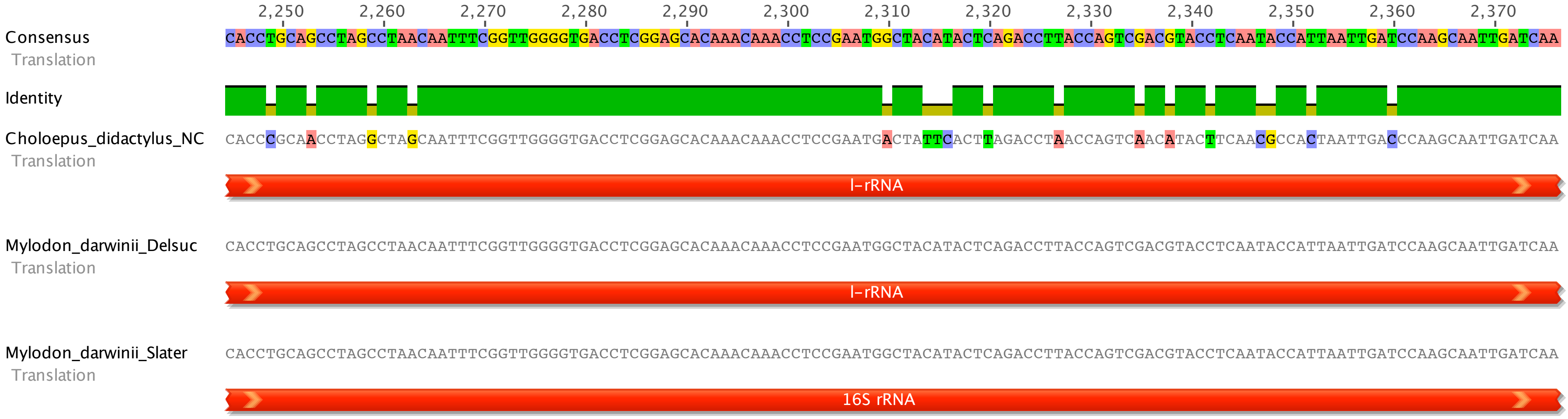
2,250 2,260 2,270 2,280 2,290 2,300 2,310 2,320 2,330 2,340 2,350 2,360 2,370
Consensus
CACCTGCAGCCTAGCCTAACAATTTCGGTTGGGGTGACCTCGGAGCACAAACAAACCTCCGAATGGCTACATACTCAGACCTTACCAGTCGACGTACCTCAATACCATTAATTGATCCAAGCAATTGATCAA
Translation
Identity
Choloepus_didactylus_NC
CACCCGCAACCTAGGCTAGCAATTTCGGTTGGGGTGACCTCGGAGCACAAACAAACCTCCGAATGACTATTCACTTAGACCTAACCAGTCAACATACTTCAACGCCACTAATTGACCCAAGCAATTGATCAA
Translation
l-rRNA
Mylodon_darwinii_Delsuc
CACCTGCAGCCTAGCCTAACAATTTCGGTTGGGGTGACCTCGGAGCACAAACAAACCTCCGAATGGCTACATACTCAGACCTTACCAGTCGACGTACCTCAATACCATTAATTGATCCAAGCAATTGATCAA
Translation
l-rRNA
Mylodon_darwinii_Slater
CACCTGCAGCCTAGCCTAACAATTTCGGTTGGGGTGACCTCGGAGCACAAACAAACCTCCGAATGGCTACATACTCAGACCTTACCAGTCGACGTACCTCAATACCATTAATTGATCCAAGCAATTGATCAA
Translation
16S rRNA

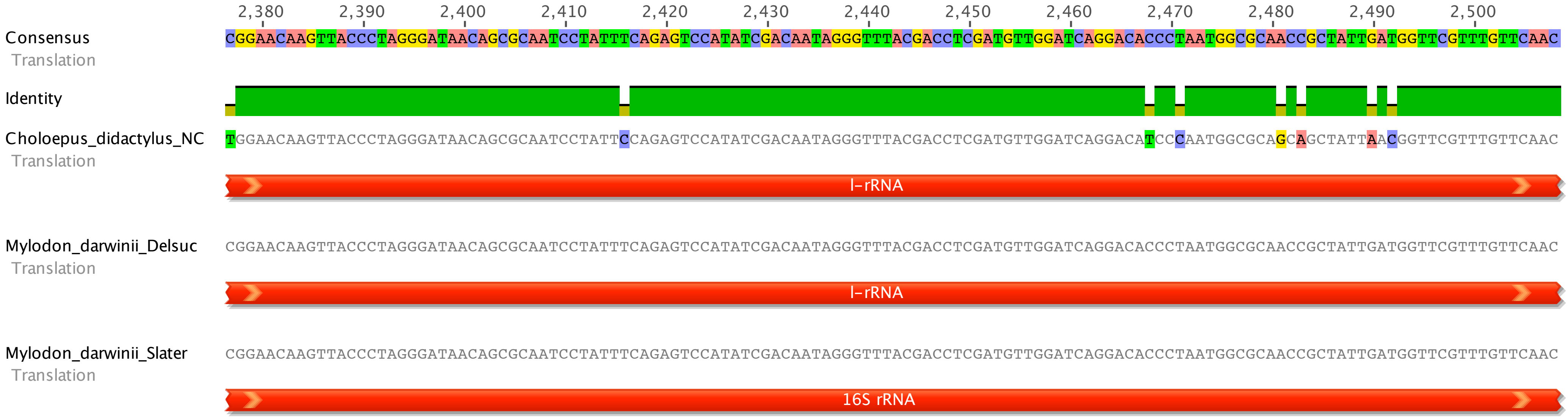
2,380 2,390 2,400 2,410 2,420 2,430 2,440 2,450 2,460 2,470 2,480 2,490 2,500
Consensus
CGGAACAAGTTACCCTAGGGATAACAGCGCAATCCTATTTCAGAGTCCATATCGACAATAGGGTTTACGACCTCGATGTTGGATCAGGACACCCTAATGGCGCAACCGCTATTGATGGTTCGTTTGTTCAAC
Translation
Identity
Choloepus_didactylus_NC
TGGAACAAGTTACCCTAGGGATAACAGCGCAATCCTATTCCAGAGTCCATATCGACAATAGGGTTTACGACCTCGATGTTGGATCAGGACATCCCAATGGCGCAGCAGCTATTAACGGTTCGTTTGTTCAAC
Translation
l-rRNA
Mylodon_darwinii_Delsuc
CGGAACAAGTTACCCTAGGGATAACAGCGCAATCCTATTTCAGAGTCCATATCGACAATAGGGTTTACGACCTCGATGTTGGATCAGGACACCCTAATGGCGCAACCGCTATTGATGGTTCGTTTGTTCAAC
Translation
l-rRNA
Mylodon_darwinii_Slater
CGGAACAAGTTACCCTAGGGATAACAGCGCAATCCTATTTCAGAGTCCATATCGACAATAGGGTTTACGACCTCGATGTTGGATCAGGACACCCTAATGGCGCAACCGCTATTGATGGTTCGTTTGTTCAAC
Translation
16S rRNA

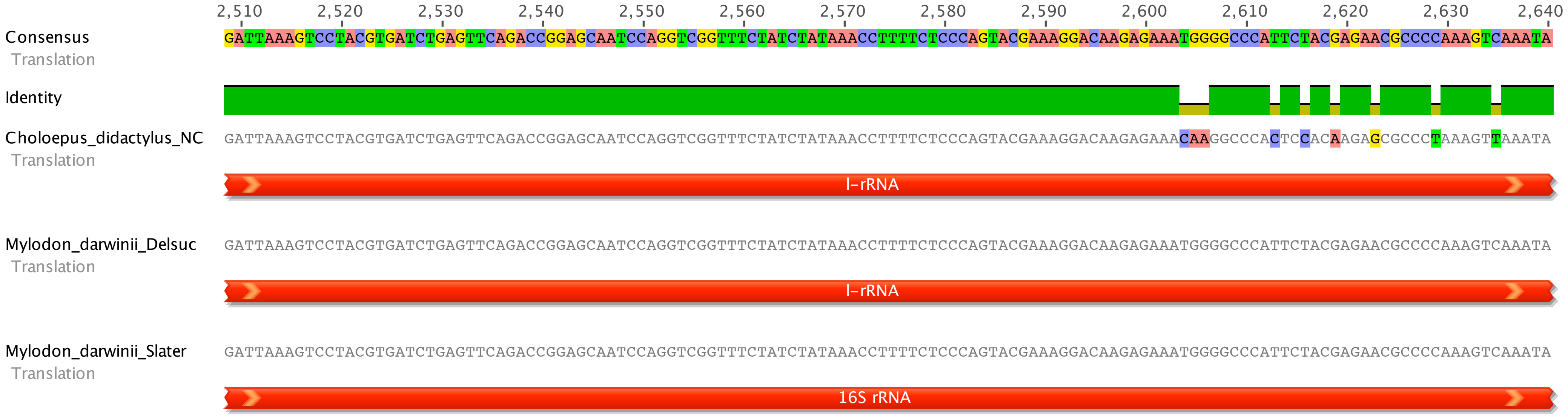
2,510 2,520 2,530 2,540 2,550 2,560 2,570 2,580 2,590 2,600 2,610 2,620 2,630 2,640
Consensus
GATTAAAGTCCTACGTGATCTGAGTTCAGACCGGAGCAATCCAGGTCGGTTTCTATCTATAAACCTTTTCTCCCAGTACGAAAGGACAAGAGAAATGGGGCCCATTCTACGAGAACGCCCCAAAGTCAAATA
Translation
Identity
Choloepus_didactylus_NC
GATTAAAGTCCTACGTGATCTGAGTTCAGACCGGAGCAATCCAGGTCGGTTTCTATCTATAAACCTTTTCTCCCAGTACGAAAGGACAAGAGAAACAAGGCCCACTCCACAAGAGCGCCCTAAAGTTAAATA
Translation
l-rRNA
Mylodon_darwinii_Delsuc
GATTAAAGTCCTACGTGATCTGAGTTCAGACCGGAGCAATCCAGGTCGGTTTCTATCTATAAACCTTTTCTCCCAGTACGAAAGGACAAGAGAAATGGGGCCCATTCTACGAGAACGCCCCAAAGTCAAATA
Translation
l-rRNA
Mylodon_darwinii_Slater
GATTAAAGTCCTACGTGATCTGAGTTCAGACCGGAGCAATCCAGGTCGGTTTCTATCTATAAACCTTTTCTCCCAGTACGAAAGGACAAGAGAAATGGGGCCCATTCTACGAGAACGCCCCAAAGTCAAATA
Translation
16S rRNA

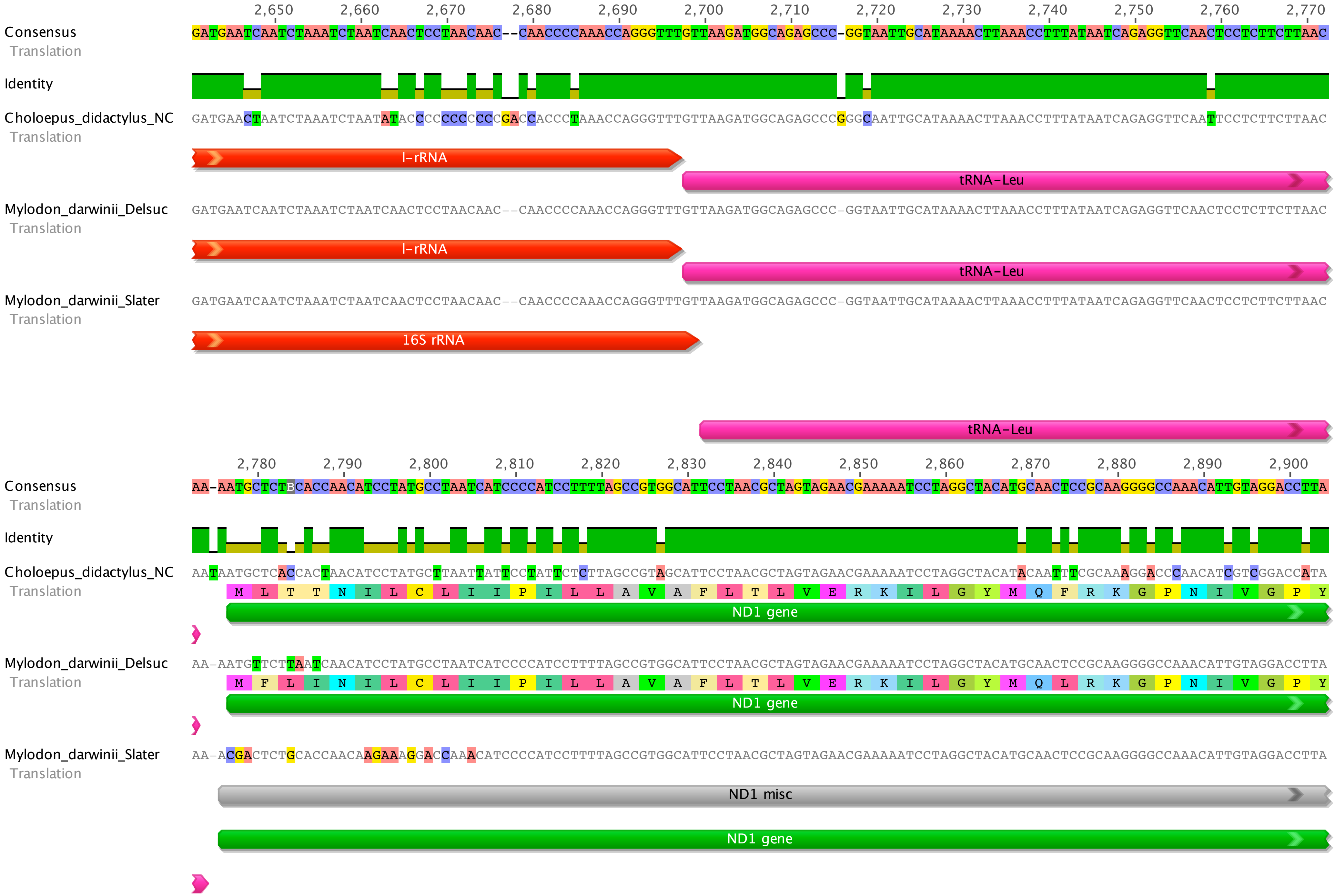
2,650 2,660 2,670 2,680 2,690 2,700 2,710 2,720 2,730 2,740 2,750 2,760 2,770
Consensus
GATGAATCAATCTAAATCTAATCAACTCCTAACAAC--CAACCCCAAACCAGGGTTTGTTAAGATGGCAGAGCCC-GGTAATTGCATAAAACTTAAACCTTTATAATCAGAGGTTCAACTCCTCTTCTTAAC
Translation
Identity
Choloepus_didactylus_NC
GATGAACTAATCTAAATCTAATATACCCCCCCCCCGACCACCCTAAACCAGGGTTTGTTAAGATGGCAGAGCCCGGGCAATTGCATAAAACTTAAACCTTTATAATCAGAGGTTCAATTCCTCTTCTTAAC
Translation
l-rRNA
tRNA-Leu
Mylodon_darwinii_Delsuc
GATGAATCAATCTAAATCTAATCAACTCCTAACAAC--CAACCCCAAACCAGGGTTTGTTAAGATGGCAGAGCCC-GGTAATTGCATAAAACTTAAACCTTTATAATCAGAGGTTCAACTCCTCTTCTTAAC
Translation
l-rRNA
tRNA-Leu
Mylodon_darwinii_Slater
GATGAATCAATCTAAATCTAATCAACTCCTAACAAC--CAACCCCAAACCAGGGTTTGTTAAGATGGCAGAGCCC-GGTAATTGCATAAAACTTAAACCTTTATAATCAGAGGTTCAACTCCTCTTCTTAAC
Translation
16S rRNA
tRNA-Leu
2,780 2,790 2,800 2,810 2,820 2,830 2,840 2,850 2,860 2,870 2,880 2,890 2,900
Consensus
AA-AATGCTCTBCACCAACATCCTATGCCTAATCATCCCCATCCTTTTAGCCGTGGCATTCCTAACGCTAGTAGAACGAAAAATCCTAGGCTACATGCAACTCCGCAAGGGGCCAAACATTGTAGGACCTTA
Translation
Identity
Choloepus_didactylus_NC
AATAATGCTCACCACTAACATCCTATGCTTAATTATTCCTATTCTCTTAGCCGTAGCATTCCTAACGCTAGTAGAACGAAAAATCCTAGGCTACATACAATTTCGCAAAGGACCCAACATCGTCGGACCATA
Translation
M L T T N I L C L I I P I L L A V A F L T L V E R K I L G Y M Q F R K G P N I V G P Y
ND1 gene
Mylodon_darwinii_Delsuc
AA-AATGTTCTTAATCAACATCCTATGCCTAATCATCCCCATCCTTTTAGCCGTGGCATTCCTAACGCTAGTAGAACGAAAAATCCTAGGCTACATGCAACTCCGCAAGGGGCCAAACATTGTAGGACCTTA
Translation
M F L I N I L C L I I P I L L A V A F L T L V E R K I L G Y M Q L R K G P N I V G P Y
ND1 gene
Mylodon_darwinii_Slater
AA-ACGACTCTGCACCAACAAGAAAGGACCAAACATCCCCATCCTTTTAGCCGTGGCATTCCTAACGCTAGTAGAACGAAAAATCCTAGGCTACATGCAACTCCGCAAGGGGCCAAACATTGTAGGACCTTA
Translation
ND1 misc
ND1 gene

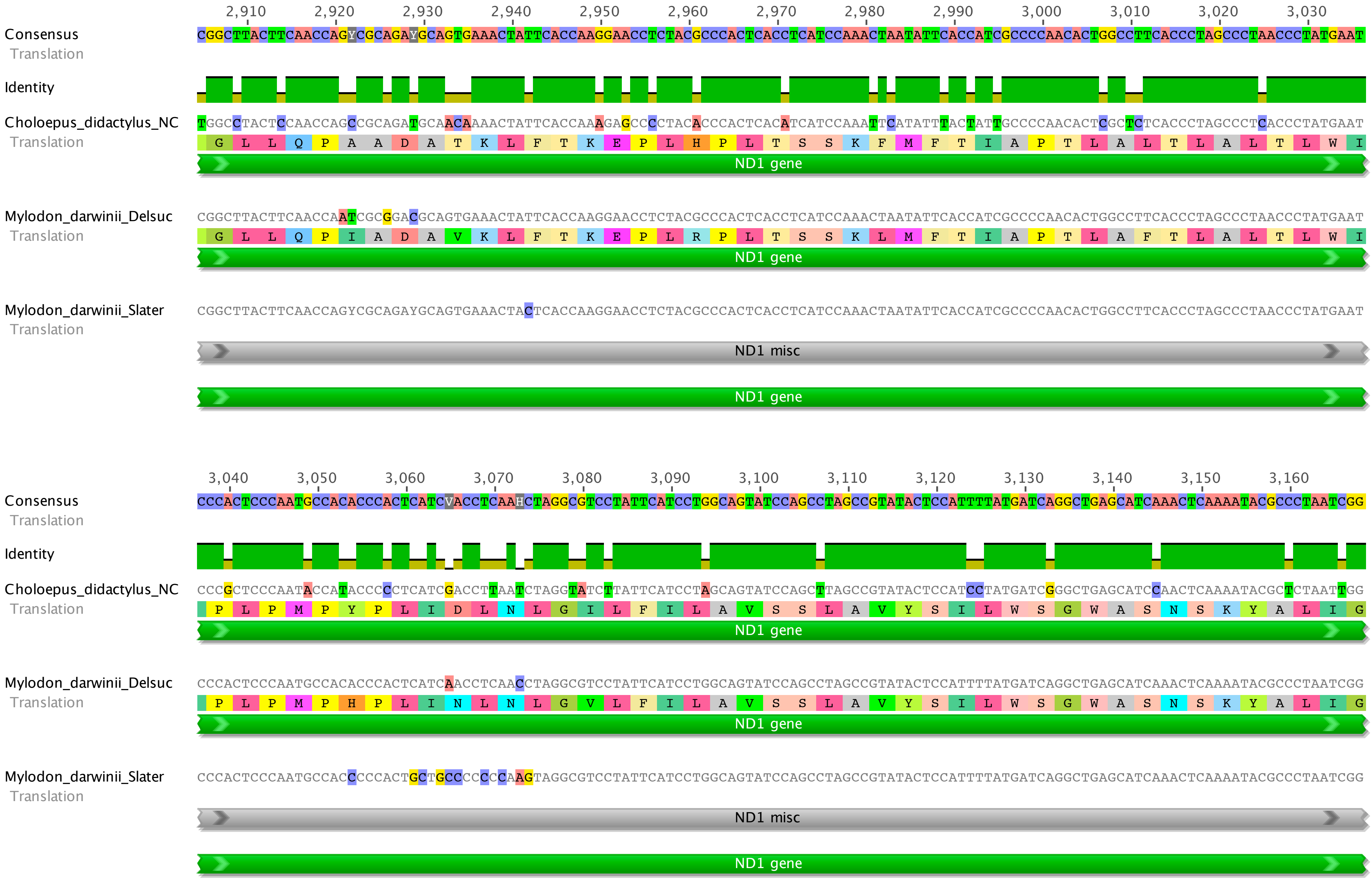
2,910 2,920 2,930 2,940 2,950 2,960 2,970 2,980 2,990 3,000 3,010 3,020 3,030
Consensus
CGGCTTACTTCAACCAGYCGCAGAYGCAGTGAAACTATTCACCAAGGAACCTCTACGCCCACTCACCTCATCCAAACTAATATTCACCATCGCCCCAACACTGGCCTTCACCCTAGCCCTAACCCTATGAAT
Translation
Identity
Choloepus_didactylus_NC
TGGCCTACTCCAACCAGCCGCAGATGCAACAAAACTATTCACCAAAGAGCCCCTACACCCACTCACATCATCCAAATTCATATTTACTATTGCCCCAACACTCGCTCTCACCCTAGCCCTCACCCTATGAAT
Translation
G L L Q P A A D A T K L F T K E P L H P L T S S K F M F T I A P T L A L T L A L T L W I
ND1 gene
Mylodon_darwinii_Delsuc
CGGCTTACTTCAACCAATCGCGGACGCAGTGAAACTATTCACCAAGGAACCTCTACGCCCACTCACCTCATCCAAACTAATATTCACCATCGCCCCAACACTGGCCTTCACCCTAGCCCTAACCCTATGAAT
Translation
G L L Q P I A D A V K L F T K E P L R P L T S S K L M F T I A P T L A F T L A L T L W I
ND1 gene
Mylodon_darwinii_Slater
CGGCTTACTTCAACCAGYCGCAGAYGCAGTGAAACTACTCACCAAGGAACCTCTACGCCCACTCACCTCATCCAAACTAATATTCACCATCGCCCCAACACTGGCCTTCACCCTAGCCCTAACCCTATGAAT
Translation
ND1 misc
ND1 gene
3,040 3,050 3,060 3,070 3,080 3,090 3,100 3,110 3,120 3,130 3,140 3,150 3,160
Consensus
CCCACTCCCAATGCCACACCCACTCATCVACCTCAAHCTAGGCGTCCTATTCATCCTGGCAGTATCCAGCCTAGCCGTATACTCCATTTTATGATCAGGCTGAGCATCAAACTCAAAATACGCCCTAATCGG
Translation
Identity
Choloepus_didactylus_NC
CCCGCTCCCAATACCATACCCCCTCATCGACCTTAATCTAGGTATCTTATTCATCCTAGCAGTATCCAGCTTAGCCGTATACTCCATCCTATGATCGGGCTGAGCATCCAACTCAAAATACGCTCTAATTGG
Translation
P L P M P Y P L I D L N L G I L F I L A V S S L A V Y S I L W S G W A S N S K Y A L I G
ND1 gene
Mylodon_darwinii_Delsuc
CCCACTCCCAATGCCACACCCACTCATCAACCTCAACCTAGGCGTCCTATTCATCCTGGCAGTATCCAGCCTAGCCGTATACTCCATTTTATGATCAGGCTGAGCATCAAACTCAAAATACGCCCTAATCGG
Translation
P L P M P H P L I N L N L G V L F I L A V S S L A V Y S I L W S G W A S N S K Y A L I G
ND1 gene
Mylodon_darwinii_Slater
CCCACTCCCAATGCCACCCCCACTGCTGCCCCCCCAAGTAGGCGTCCTATTCATCCTGGCAGTATCCAGCCTAGCCGTATACTCCATTTTATGATCAGGCTGAGCATCAAACTCAAAATACGCCCTAATCGG
Translation
ND1 misc
ND1 gene

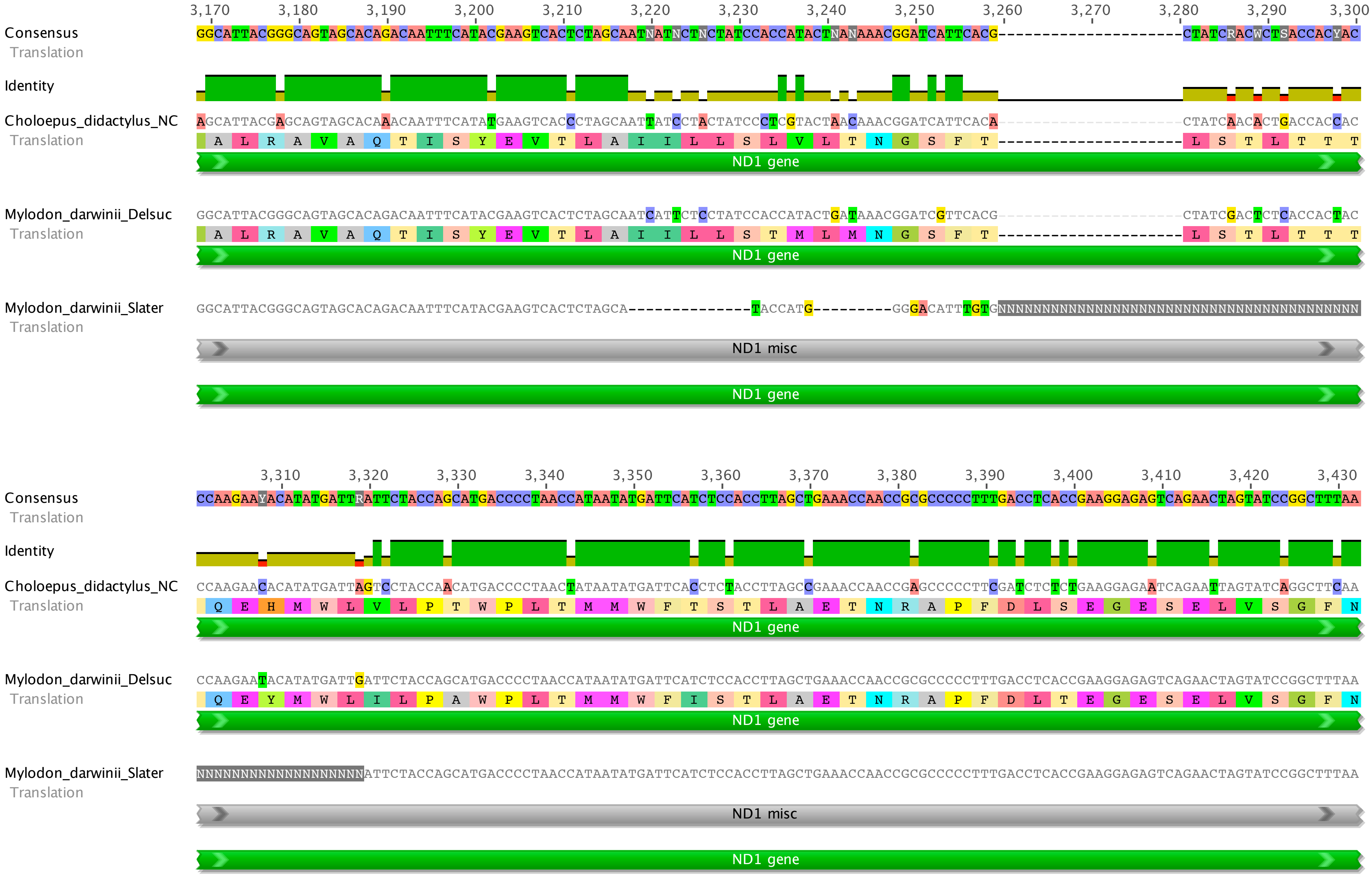
3,170 3,180 3,190 3,200 3,210 3,220 3,230 3,240 3,250 3,260 3,270 3,280 3,290 3,300
Consensus
Translation
Identity
Choloepus_didactylus_NC
Translation
ND1 gene
Mylodon_darwinii_Delsuc
Translation
ND1 gene
Mylodon_darwinii_Slater
Translation
ND1 misc
ND1 gene
3,310 3,320 3,330 3,340 3,350 3,360 3,370 3,380 3,390 3,400 3,410 3,420 3,430
Consensus
Translation
Identity
Choloepus_didactylus_NC
Translation
ND1 gene
Mylodon_darwinii_Delsuc
Translation
ND1 gene
Mylodon_darwinii_Slater
Translation
ND1 misc
ND1 gene

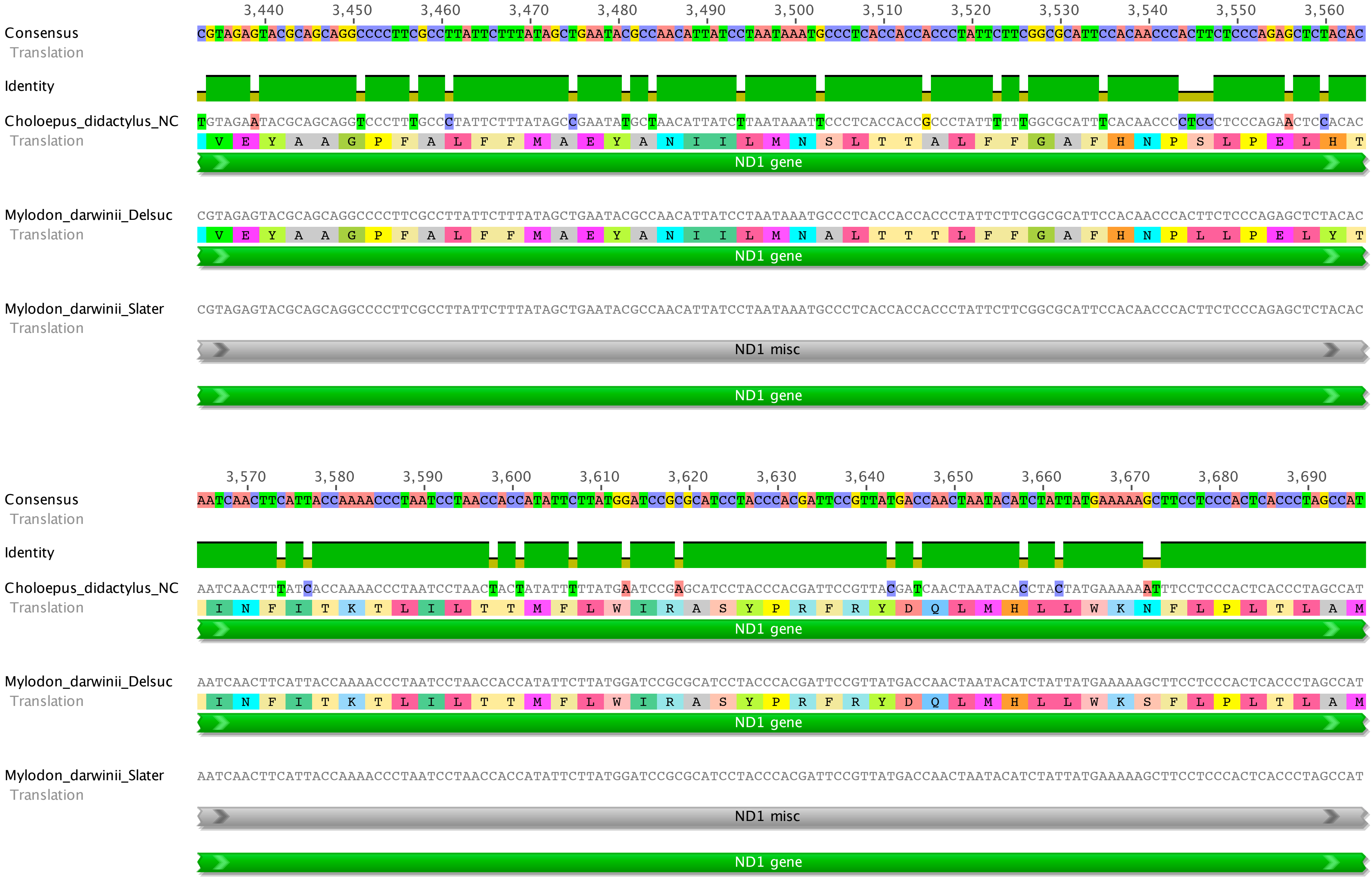
3,440 3,450 3,460 3,470 3,480 3,490 3,500 3,510 3,520 3,530 3,540 3,550 3,560
Consensus
CGTAGAGTACGCAGCAGGCCCCTTCGCCTTATTCTTTATAGCTGAATACGCCAACATTATCCTAATAAATGCCCTCACCACCACCCTATTCTTCGGCGCATTCCACAACCCACTTCTCCCAGAGCTCTACAC
Translation
Identity
Choloepus_didactylus_NC
TGTAGAATACGCAGCAGGTCCCTTTGCCCTATTCTTTATAGCCGAATATGCTAACATTATCTTAATAAATTCCCTCACCACCGCCCTATTTTTTGGCGCATTTCACAACCCCTCCCTCCCAGAACTCCACAC
Translation
V E Y A A G P F A L F F M A E Y A N I I L M N S L T T A L F F G A F H N P S L P E L H T
ND1 gene
Mylodon_darwinii_Delsuc
CGTAGAGTACGCAGCAGGCCCCTTCGCCTTATTCTTTATAGCTGAATACGCCAACATTATCCTAATAAATGCCCTCACCACCACCCTATTCTTCGGCGCATTCCACAACCCACTTCTCCCAGAGCTCTACAC
Translation
V E Y A A G P F A L F F M A E Y A N I I L M N A L T T T L F F G A F H N P L L P E L Y T
ND1 gene
Mylodon_darwinii_Slater
CGTAGAGTACGCAGCAGGCCCCTTCGCCTTATTCTTTATAGCTGAATACGCCAACATTATCCTAATAAATGCCCTCACCACCACCCTATTCTTCGGCGCATTCCACAACCCACTTCTCCCAGAGCTCTACAC
Translation
ND1 misc
ND1 gene
3,570 3,580 3,590 3,600 3,610 3,620 3,630 3,640 3,650 3,660 3,670 3,680 3,690
Consensus
AATCAACTTCATTACCAAAACCCTAATCCTAACCACCATATTCTTATGGATCCGCGCATCCTACCCACGATTCCGTTATGACCAACTAATACATCTATTATGAAAAAGCTTCCTCCCACTCACCCTAGCCAT
Translation
Identity
Choloepus_didactylus_NC
AATCAACTTTATCACCAAAACCCTAATCCTAACTACTATATTTTTATGAATCCGAGCATCCTACCCACGATTCCGTTACGATCAACTAATACACCTACTATGAAAAATTTCCTCCCACTCACCCTAGCCAT
Translation
I N F I T K T L I L T T M F L W I R A S Y P R F R Y D Q L M H L L W K N F L P L T L A M
ND1 gene
Mylodon_darwinii_Delsuc
AATCAACTTCATTACCAAAACCCTAATCCTAACCACCATATTCTTATGGATCCGCGCATCCTACCCACGATTCCGTTATGACCAACTAATACATCTATTATGAAAAAGCTTCCTCCCACTCACCCTAGCCAT
Translation
I N F I T K T L I L T T M F L W I R A S Y P R F R Y D Q L M H L L W K S F L P L T L A M
ND1 gene
Mylodon_darwinii_Slater
AATCAACTTCATTACCAAAACCCTAATCCTAACCACCATATTCTTATGGATCCGCGCATCCTACCCACGATTCCGTTATGACCAACTAATACATCTATTATGAAAAAGCTTCCTCCCACTCACCCTAGCCAT
Translation
ND1 misc
ND1 gene

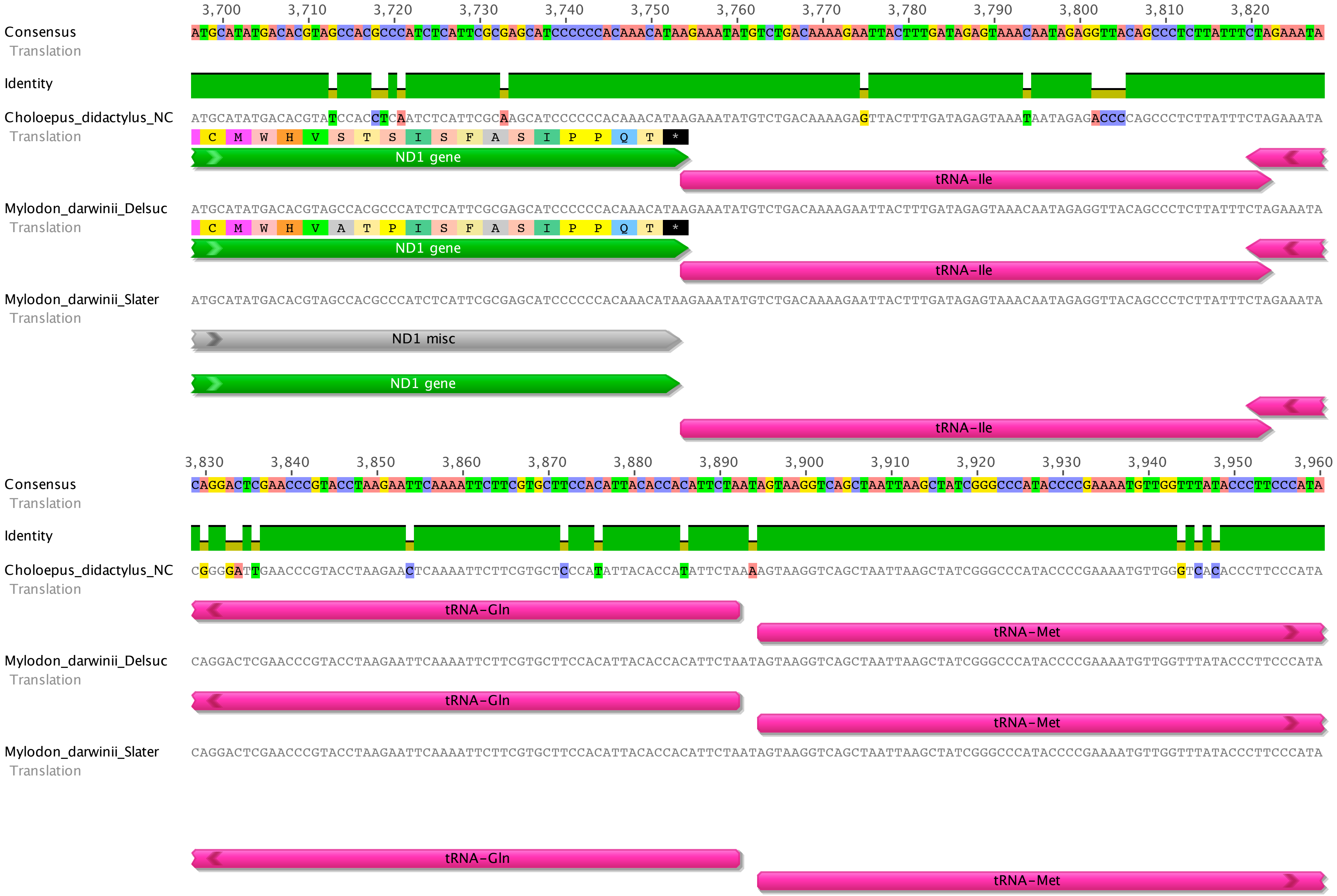

3,700 3,710 3,720 3,730 3,740 3,750 3,760 3,770 3,780 3,790 3,800 3,810 3,820
Consensus
ATGCATATGACACGTAGCCACGCCCATCTCATTCGCGAGCATCCCCCCACAAACATAAGAAATATGTCTGACAAAAGAATTACTTTGATAGAGTAAACAATAGAGGTTACAGCCCTCTTATTTCTAGAAATA
Translation
Identity
Choloepus_didactylus_NC
ATGCATATGACACGTATCCACCTCAATCTCATTCGCAAGCATCCCCCCACAAACATAAGAAATATGTCTGACAAAAGAGTTACTTTGATAGAGTAAATAATAGAGACCCCAGCCCTCTTATTTCTAGAAATA
Translation
C M W H V S T S I S F A S I P P Q T *
ND1 gene
tRNA-Ile
Mylodon_darwinii_Delsuc
ATGCATATGACACGTAGCCACGCCCATCTCATTCGCGAGCATCCCCCCACAAACATAAGAAATATGTCTGACAAAAGAATTACTTTGATAGAGTAAACAATAGAGGTTACAGCCCTCTTATTTCTAGAAATA
Translation
C M W H V A T P I S F A S I P P Q T *
ND1 gene
tRNA-Ile
Mylodon_darwinii_Slater
ATGCATATGACACGTAGCCACGCCCATCTCATTCGCGAGCATCCCCCCACAAACATAAGAAATATGTCTGACAAAAGAATTACTTTGATAGAGTAAACAATAGAGGTTACAGCCCTCTTATTTCTAGAAATA
Translation
ND1 misc
ND1 gene
tRNA-Ile
3,830 3,840 3,850 3,860 3,870 3,880 3,890 3,900 3,910 3,920 3,930 3,940 3,950 3,960
Consensus
CAGGACTCGAACCCGTACCTAAGAATTCAAAATTCTTCGTGCTTCCACATTACACCACATTCTAATAGTAAGGTCAGCTAATTAAGCTATCGGGCCCATACCCCGAAAATGTTGGTTTATACCCTTCCCATA
Translation
Identity
Choloepus_didactylus_NC
CGGGGATTGAACCCGTACCTAAGAACTCAAAATTCTTCGTGCTCCCATATTACACCATATTCTAAAAGTAAGGTCAGCTAATTAAGCTATCGGGCCCATACCCCGAAAATGTTGGGTCACACCCTTCCCATA
Translation
tRNA-Gln
tRNA-Met
Mylodon_darwinii_Delsuc
CAGGACTCGAACCCGTACCTAAGAATTCAAAATTCTTCGTGCTTCCACATTACACCACATTCTAATAGTAAGGTCAGCTAATTAAGCTATCGGGCCCATACCCCGAAAATGTTGGTTTATACCCTTCCCATA
Translation
tRNA-Gln
tRNA-Met
Mylodon_darwinii_Slater
CAGGACTCGAACCCGTACCTAAGAATTCAAAATTCTTCGTGCTTCCACATTACACCACATTCTAATAGTAAGGTCAGCTAATTAAGCTATCGGGCCCATACCCCGAAAATGTTGGTTTATACCCTTCCCATA
Translation
tRNA-Gln
tRNA-Met

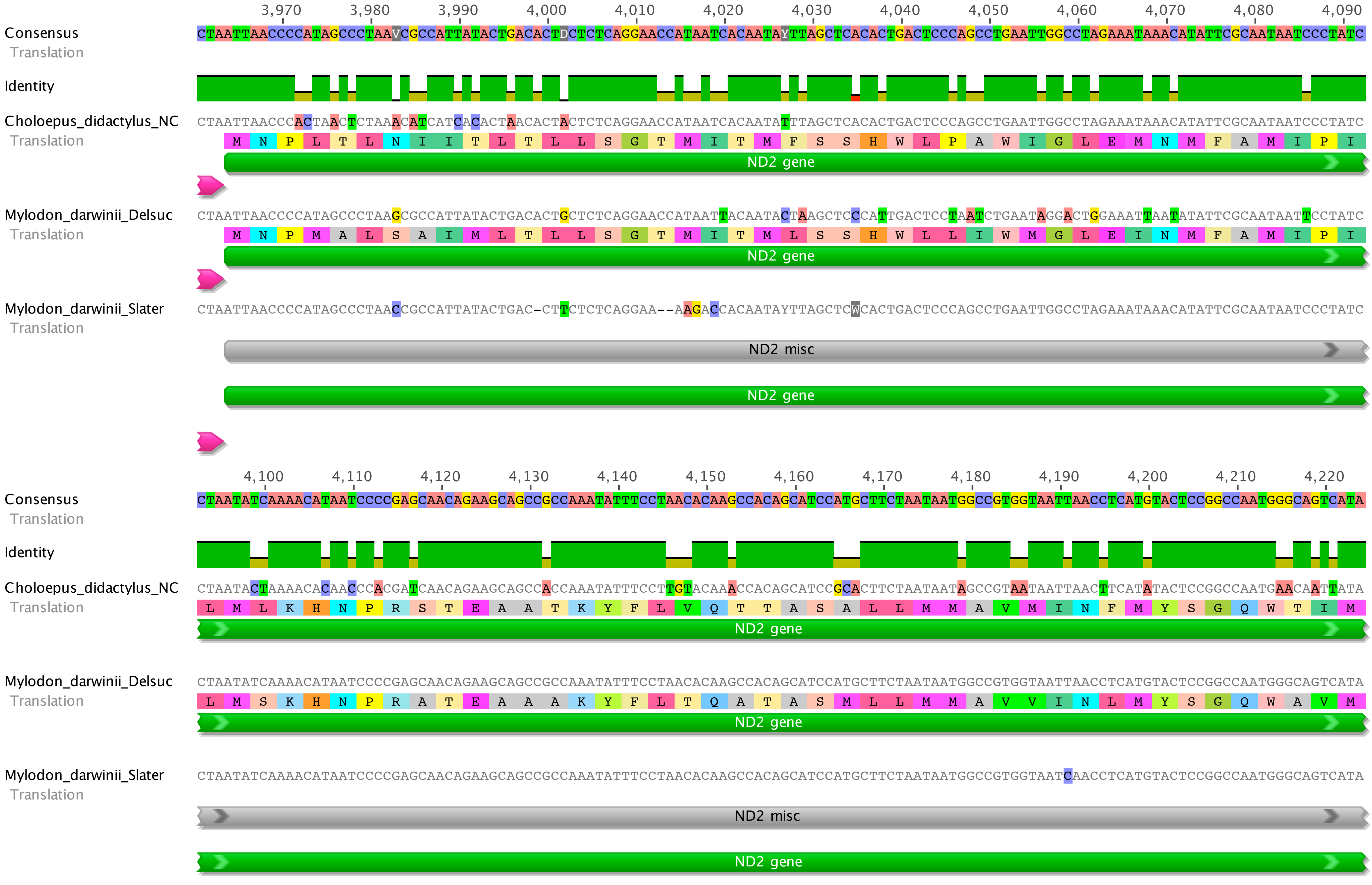
3,970 3,980 3,990 4,000 4,010 4,020 4,030 4,040 4,050 4,060 4,070 4,080 4,090
Consensus
CTAATTAACCCCATAGCCCTAAVCGCCATTATACTGACACTDCTCTCAGGAACCATAATCACAATAYTTAGCTCACACTGACTCCCAGCCTGAATTGGCCTAGAAATAAACATATTCGCAATAATCCCTATC
Translation
Identity
Choloepus_didactylus_NC
CTAATTAACCCACTAACTCTAAACATCATCACACTAACACTACTCTCAGGAACCATAATCACAATATTTAGCTCACACTGACTCCCAGCCTGAATTGGCCTAGAAATAAACATATTCGCAATAATCCCTATC
Translation
M N P L T L N I I T L T L L S G T M I T M F S S H W L P A W I G L E M N M F A M I P I
ND2 gene
Mylodon_darwinii_Delsuc
CTAATTAACCCCATAGCCCTAAGCGCCATTATACTGACACTGCTCTCAGGAACCATAATTACAATACTAAGCTCCCATTGACTCCTAATCTGAATAGGACTGGAAATTAATATATTCGCAATAATTCCTATC
Translation
M N P M A L S A I M L T L L S G T M I T M L S S H W L L I W M G L E I N M F A M I P I
ND2 gene
Mylodon_darwinii_Slater
CTAATTAACCCCATAGCCCTAACCGCCATTATACTGAC-CTTCTCTCAGGAA--AAGACCACAATAYTTAGCTCWCACTGACTCCCAGCCTGAATTGGCCTAGAAATAAACATATTCGCAATAATCCCTATC
Translation
ND2 misc
ND2 gene
4,100 4,110 4,120 4,130 4,140 4,150 4,160 4,170 4,180 4,190 4,200 4,210 4,220
Consensus
CTAATATCAAAACATAATCCCCGAGCAACAGAAGCAGCCGCCAAATATTTCCTAACACAAGCCACAGCATCCATGCTTCTAATAATGGCCGTGGTAATTAACCTCATGTACTCCGGCCAATGGGCAGTCATA
Translation
Identity
Choloepus_didactylus_NC
CTAATACTAAAACACAACCCACGATCAACAGAAGCAGCCACCAAATATTTCCTTGTACAAACCACAGCATCCGCACTTCTAATAATAGCCGTAATAATTAACTTCATAATACTCCGGCCAATGAACAATTATA
Translation
L M L K H N P R S T E A A T K Y F L V Q T T A S A L L M M A V M I N F M Y S G Q W T I M
ND2 gene
Mylodon_darwinii_Delsuc
CTAATATCAAAACATAATCCCCGAGCAACAGAAGCAGCCGCCAAATATTTCCTAACACAAGCCACAGCATCCATGCTTCTAATAATGGCCGTGGTAATTAACCTCATGTACTCCGGCCAATGGGCAGTCATA
Translation
L M S K H N P R A T E A A A K Y F L T Q A T A S M L L M M A V V I N L M Y S G Q W A V M
ND2 gene
Mylodon_darwinii_Slater
CTAATATCAAAACATAATCCCCGAGCAACAGAAGCAGCCGCCAAATATTTCCTAACACAAGCCACAGCATCCATGCTTCTAATAATGGCCGTGGTAATCAACCTCATGTACTCCGGCCAATGGGCAGTCATA
Translation
ND2 misc
ND2 gene

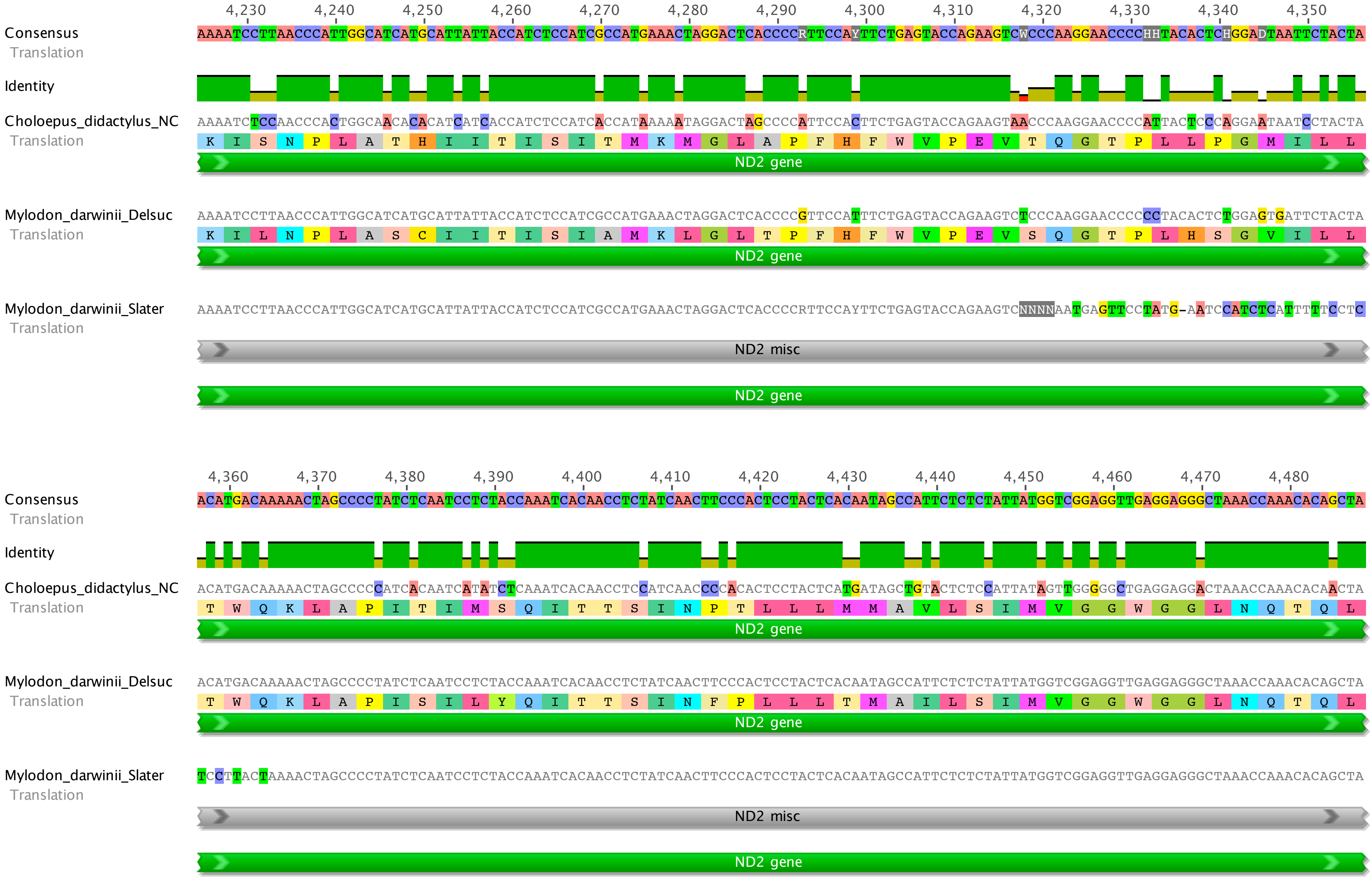
4,230 4,240 4,250 4,260 4,270 4,280 4,290 4,300 4,310 4,320 4,330 4,340 4,350
Consensus
Translation
Identity
Choloepus_didactylus_NC
Translation
ND2 gene
Mylodon_darwinii_Delsuc
Translation
ND2 gene
Mylodon_darwinii_Slater
Translation
ND2 misc
ND2 gene
4,360 4,370 4,380 4,390 4,400 4,410 4,420 4,430 4,440 4,450 4,460 4,470 4,480
Consensus
Translation
Identity
Choloepus_didactylus_NC
Translation
ND2 gene
Mylodon_darwinii_Delsuc
Translation
ND2 gene
Mylodon_darwinii_Slater
Translation
ND2 misc
ND2 gene

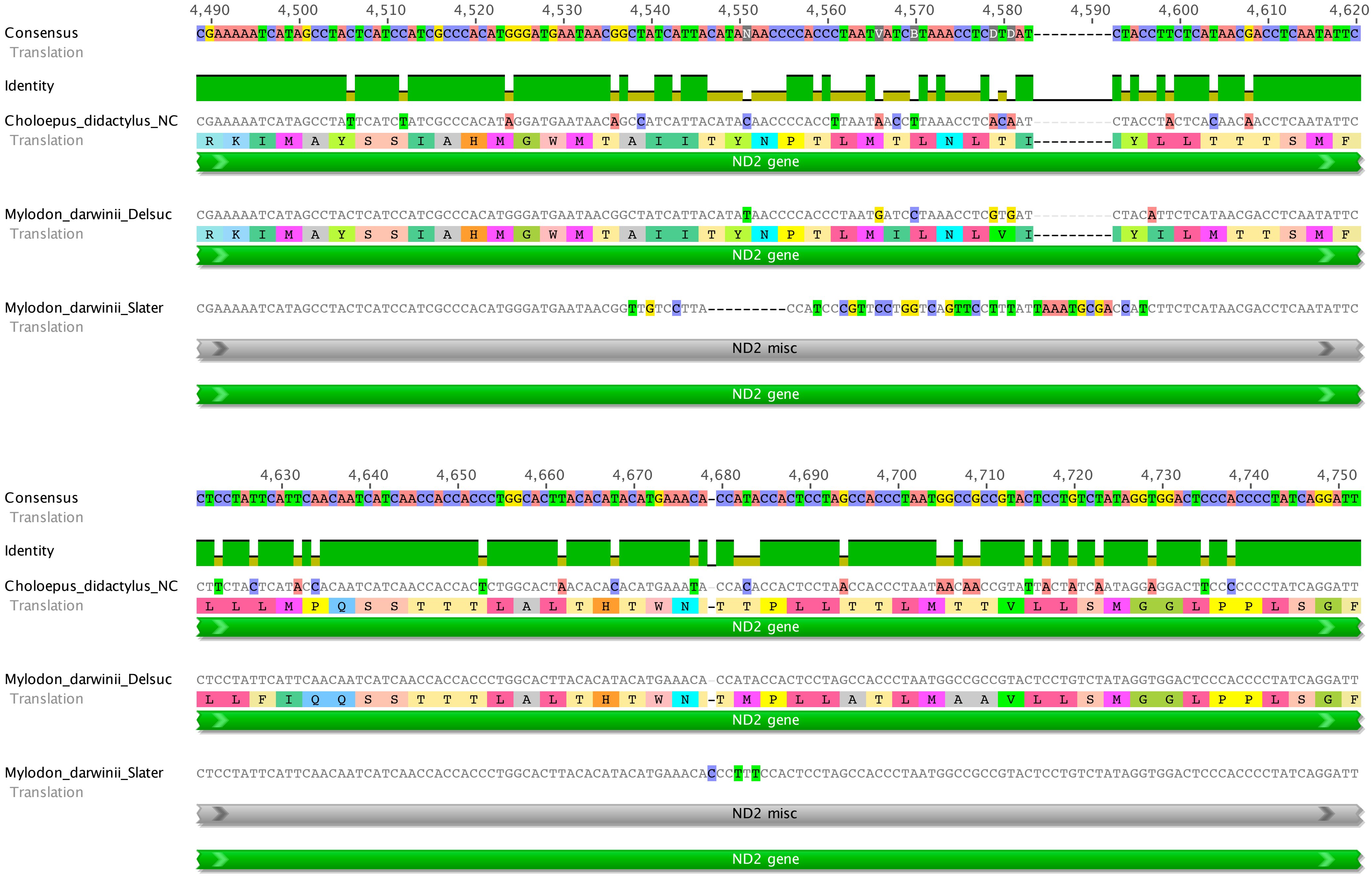
4,490
4,500
4,510
4,520
4,530
4,540
4,550
4,560
4,570
4,580
4,590
4,600
4,610
4,620
Consensus
Translation
Identity
Choloepus_didactylus_NC
Translation
ND2 gene
Mylodon_darwinii_Delsuc
Translation
ND2 gene
Mylodon_darwinii_Slater
Translation
ND2 misc
ND2 gene
4,630
4,640
4,650
4,660
4,670
4,680
4,690
4,700
4,710
4,720
4,730
4,740
4,750
Consensus
Translation
Identity
Choloepus_didactylus_NC
Translation
ND2 gene
Mylodon_darwinii_Delsuc
Translation
ND2 gene
Mylodon_darwinii_Slater
Translation
ND2 misc
ND2 gene

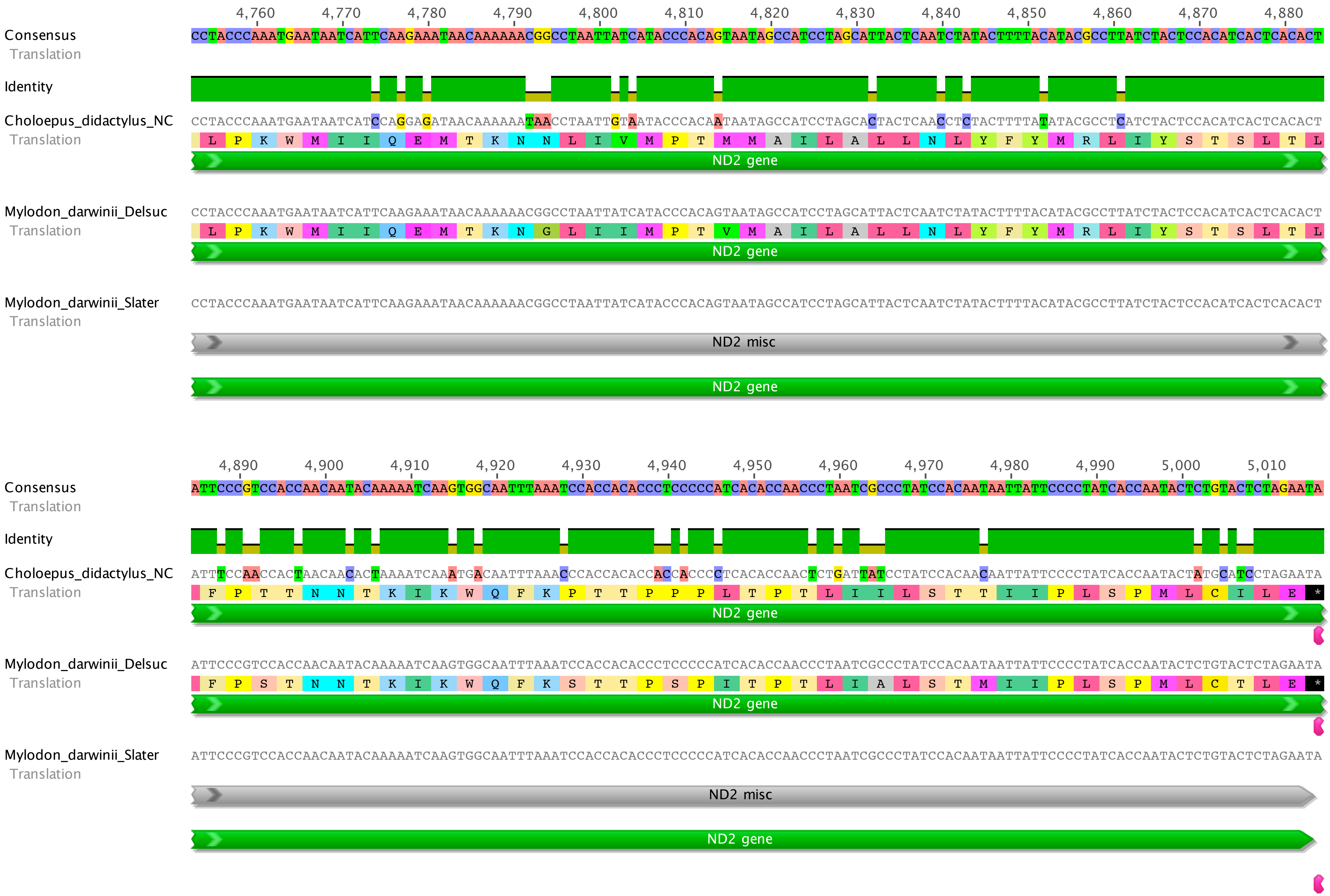
4,760 4,770 4,780 4,790 4,800 4,810 4,820 4,830 4,840 4,850 4,860 4,870 4,880
Consensus
Translation
Identity
Choloepus_didactylus_NC
Translation
ND2 gene
Mylodon_darwinii_Delsuc
Translation
ND2 gene
Mylodon_darwinii_Slater
Translation
ND2 misc
ND2 gene
4,890 4,900 4,910 4,920 4,930 4,940 4,950 4,960 4,970 4,980 4,990 5,000 5,010
Consensus
Translation
Identity
Choloepus_didactylus_NC
Translation
ND2 gene
Mylodon_darwinii_Delsuc
Translation
ND2 gene
Mylodon_darwinii_Slater
Translation
ND2 misc
ND2 gene

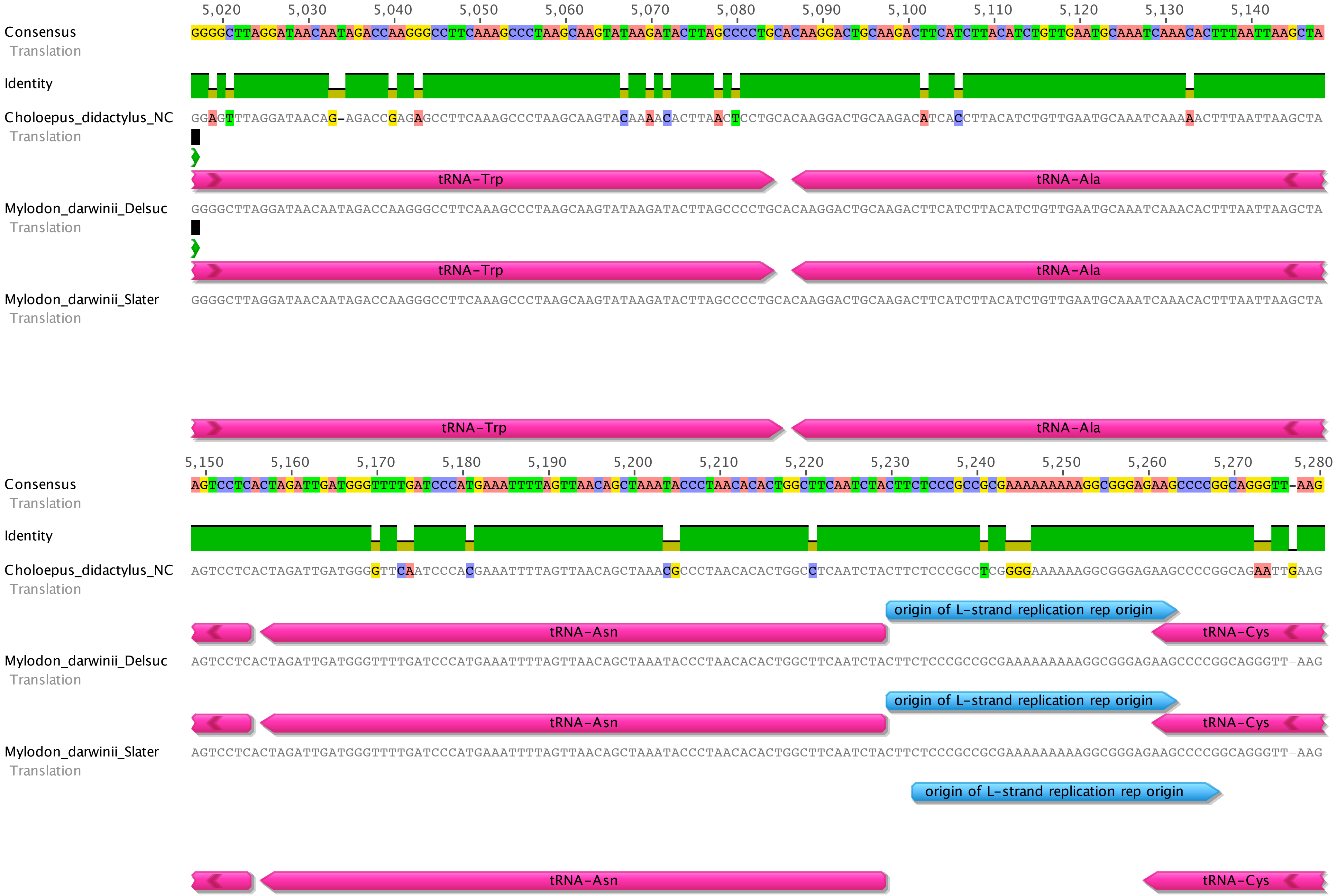
5,020 5,030 5,040 5,050 5,060 5,070 5,080 5,090 5,100 5,110 5,120 5,130 5,140
Consensus GGGGCTTAGGATAACAATAGACCAAGGGCCTTCAAAGCCCTAAGCAAGTATAAGATACTTAGCCCCTGCACAAGGACTGCAAGACTTCATCTTACATCTGTTGAATGCAAATCAAACACTTTAATTAAGCTA
Translation
Identity
Choloepus_didactylus_NC GGAGTTTAGGATAACAG-AGACCGAGAGCCTTCAAAGCCCTAAGCAAGTACAAAACACTTAACTCCTGCACAAGGACTGCAAGACATCACCTTACATCTGTTGAATGCAAATCAAAAACTTTAATTAAGCTA
Translation
tRNA-Trp
tRNA-Ala
Mylodon_darwinii_Delsuc GGGGCTTAGGATAACAATAGACCAAGGGCCTTCAAAGCCCTAAGCAAGTATAAGATACTTAGCCCCTGCACAAGGACTGCAAGACTTCATCTTACATCTGTTGAATGCAAATCAAACACTTTAATTAAGCTA
Translation
tRNA-Trp
tRNA-Ala
Mylodon_darwinii_Slater GGGGCTTAGGATAACAATAGACCAAGGGCCTTCAAAGCCCTAAGCAAGTATAAGATACTTAGCCCCTGCACAAGGACTGCAAGACTTCATCTTACATCTGTTGAATGCAAATCAAACACTTTAATTAAGCTA
Translation
tRNA-Trp
tRNA-Ala
5,150 5,160 5,170 5,180 5,190 5,200 5,210 5,220 5,230 5,240 5,250 5,260 5,270 5,280
Consensus AGTCCTCACTAGATTGATGGGTTTTGATCCCATGAAATTTTAGTTAACAGCTAAATACCCTAACACACTGGCTTCAATCTACTTCTCCCGCCGCGAAAAAAAAAGGCGGGAGAAGCCCCGGCAGGGTT-AAG
Translation
Identity
Choloepus_didactylus_NC AGTCCTCACTAGATTGATGGGGTTCAATCCCACGAAATTTTAGTTAACAGCTAAACGCCCTAACACACTGGCCTCAATCTACTTCTCCCGCCTCGGGGAAAAAAGGCGGGAGAAGCCCCGGCAGAATTGAAG
Translation
origin of L-strand replication rep origin
tRNA-Asn
tRNA-Cys
Mylodon_darwinii_Delsuc AGTCCTCACTAGATTGATGGGTTTTGATCCCATGAAATTTTAGTTAACAGCTAAATACCCTAACACACTGGCTTCAATCTACTTCTCCCGCCGCGAAAAAAAAAGGCGGGAGAAGCCCCGGCAGGGTT-AAG
Translation
origin of L-strand replication rep origin
tRNA-Asn
tRNA-Cys
Mylodon_darwinii_Slater AGTCCTCACTAGATTGATGGGTTTTGATCCCATGAAATTTTAGTTAACAGCTAAATACCCTAACACACTGGCTTCAATCTACTTCTCCCGCCGCGAAAAAAAAAGGCGGGAGAAGCCCCGGCAGGGTT-AAG
Translation
origin of L-strand replication rep origin
tRNA-Asn
tRNA-Cys

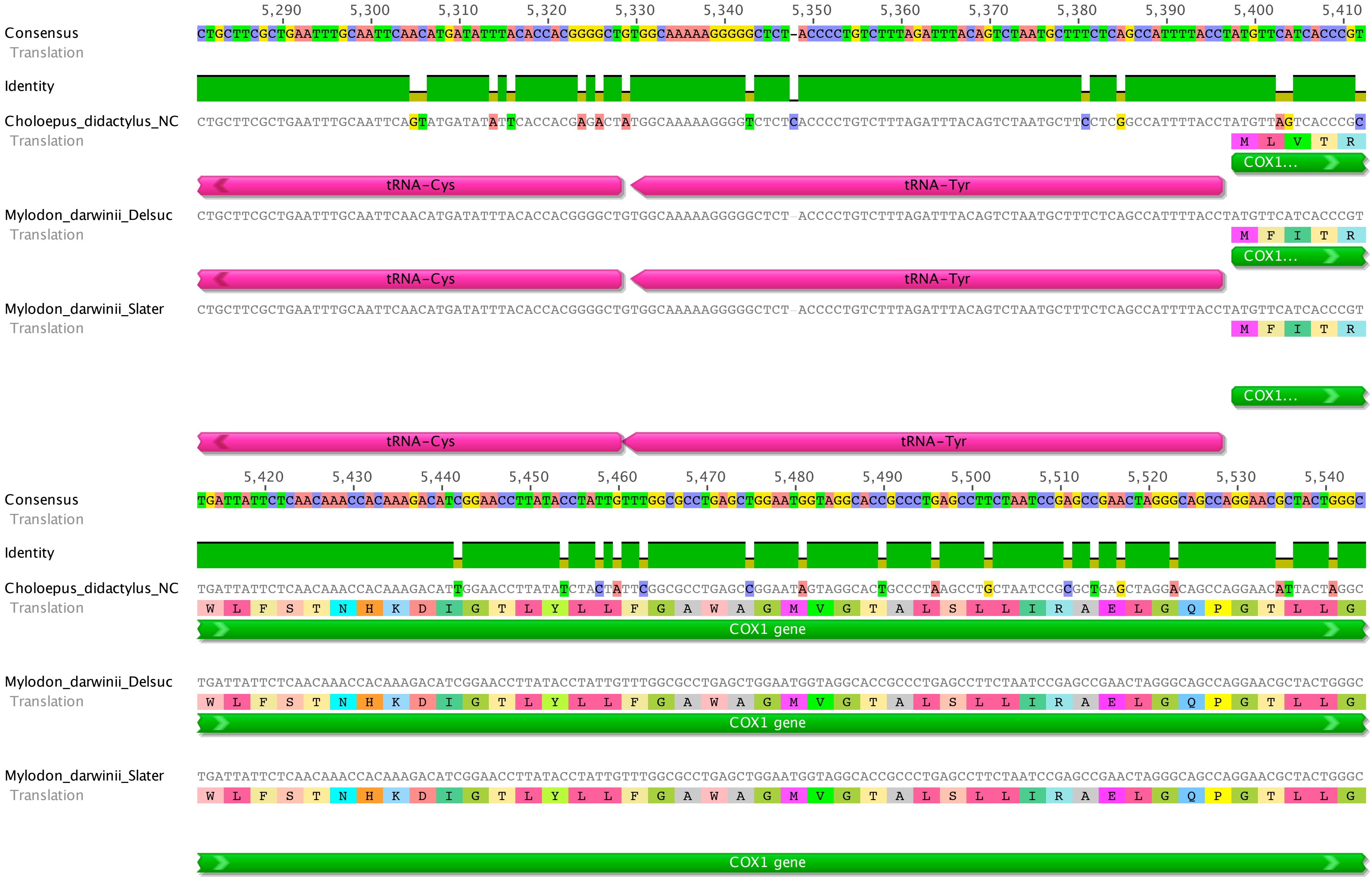
5,290 5,300 5,310 5,320 5,330 5,340 5,350 5,360 5,370 5,380 5,390 5,400 5,410
Consensus
Translation
Identity
Choloepus_didactylus_NC
Translation
COX1...
tRNA-Cys
tRNA-Tyr
Mylodon_darwinii_Delsuc
Translation
Mylodon_darwinii_Slater
Translation
5,420 5,430 5,440 5,450 5,460 5,470 5,480 5,490 5,500 5,510 5,520 5,530 5,540
COX1 gene

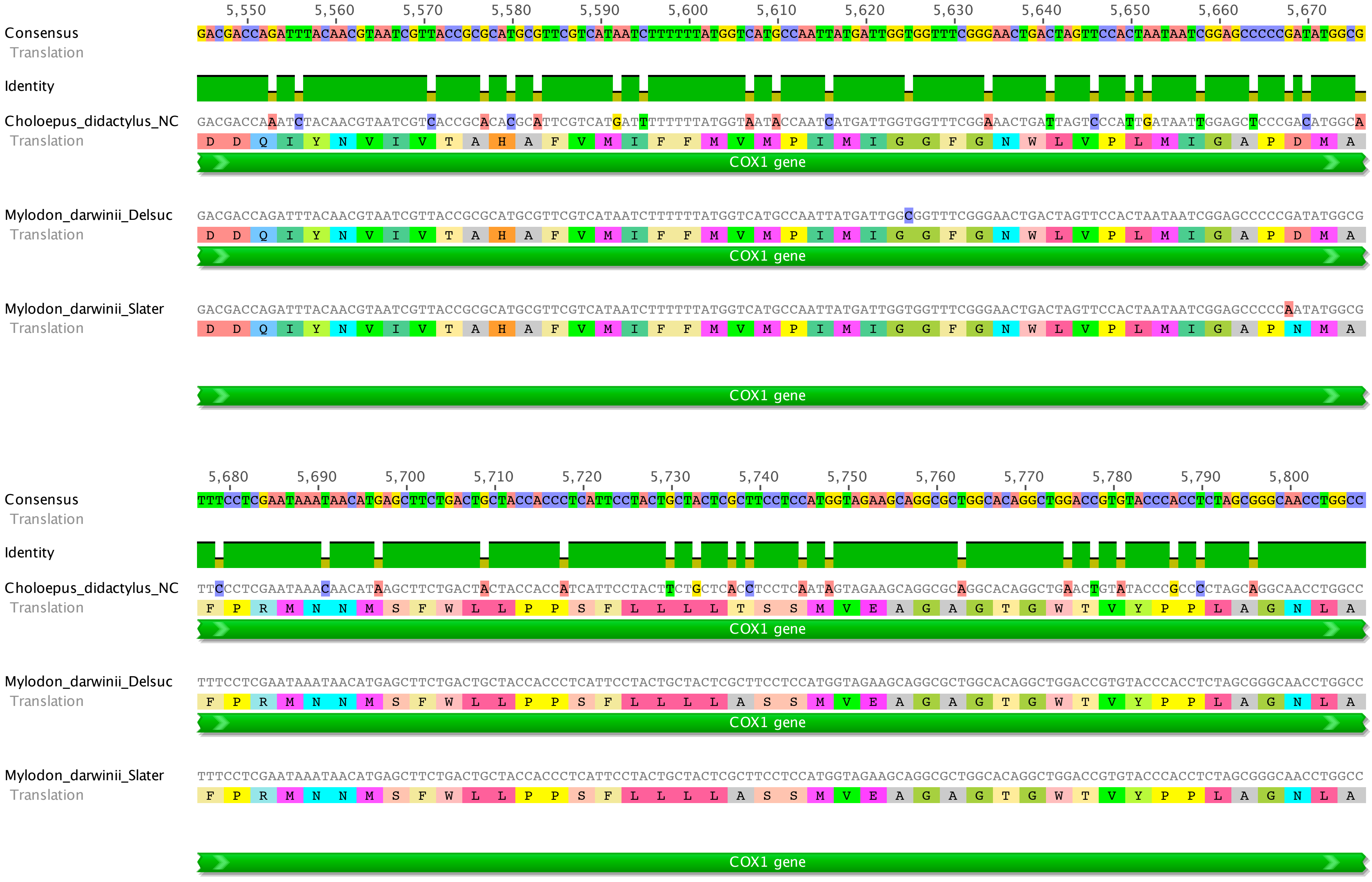
5,550 5,560 5,570 5,580 5,590 5,600 5,610 5,620 5,630 5,640 5,650 5,660 5,670
Consensus
Translation
Identity
Choloepus_didactylus_NC
Translation
COX1 gene
Mylodon_darwinii_Delsuc
Translation
COX1 gene
Mylodon_darwinii_Slater
Translation
COX1 gene
5,680 5,690 5,700 5,710 5,720 5,730 5,740 5,750 5,760 5,770 5,780 5,790 5,800
Consensus
Translation
Identity
Choloepus_didactylus_NC
Translation
COX1 gene
Mylodon_darwinii_Delsuc
Translation
COX1 gene
Mylodon_darwinii_Slater
Translation
COX1 gene

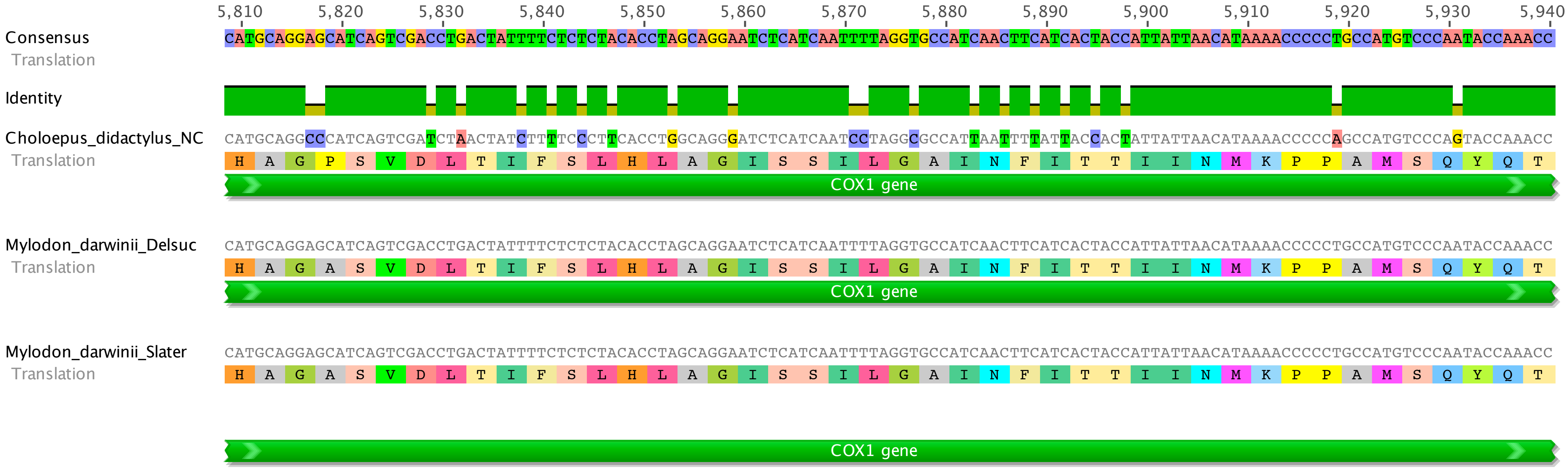
5,810 5,820 5,830 5,840 5,850 5,860 5,870 5,880 5,890 5,900 5,910 5,920 5,930 5,940
Consensus
CATGCAGGAGCATCAGTCGACCTGACTATTTTCTCTCTACACCTAGCAGGAATCTCATCAATTTTAGGTGCCATCAACTTCATCACTACCATTATTAACATAAAACCCCCTGCCATGTCCCAATACCAAACC
Translation
Identity
Choloepus_didactylus_NC
CATGCAGGCCCATCAGTCGATCTAACTATCTTTTCCCTTCACCTGGCAGGGATCTCATCAATCCTAGGCGCCATTAATTTTATTACCACTATTATTAACATAAAACCCCCAGCCATGTCCCAGTACCAAACC
Translation
H A G P S V D L T I F S L H L A G I S S I L G A I N F I T T I I N M K P P A M S Q Y Q T
COX1 gene
Mylodon_darwinii_Delsuc
CATGCAGGAGCATCAGTCGACCTGACTATTTTCTCTCTACACCTAGCAGGAATCTCATCAATTTTAGGTGCCATCAACTTCATCACTACCATTATTAACATAAAACCCCCTGCCATGTCCCAATACCAAACC
Translation
H A G A S V D L T I F S L H L A G I S S I L G A I N F I T T I I N M K P P A M S Q Y Q T
COX1 gene
Mylodon_darwinii_Slater
CATGCAGGAGCATCAGTCGACCTGACTATTTTCTCTCTACACCTAGCAGGAATCTCATCAATTTTAGGTGCCATCAACTTCATCACTACCATTATTAACATAAAACCCCCTGCCATGTCCCAATACCAAACC
Translation
H A G A S V D L T I F S L H L A G I S S I L G A I N F I T T I I N M K P P A M S Q Y Q T
COX1 gene

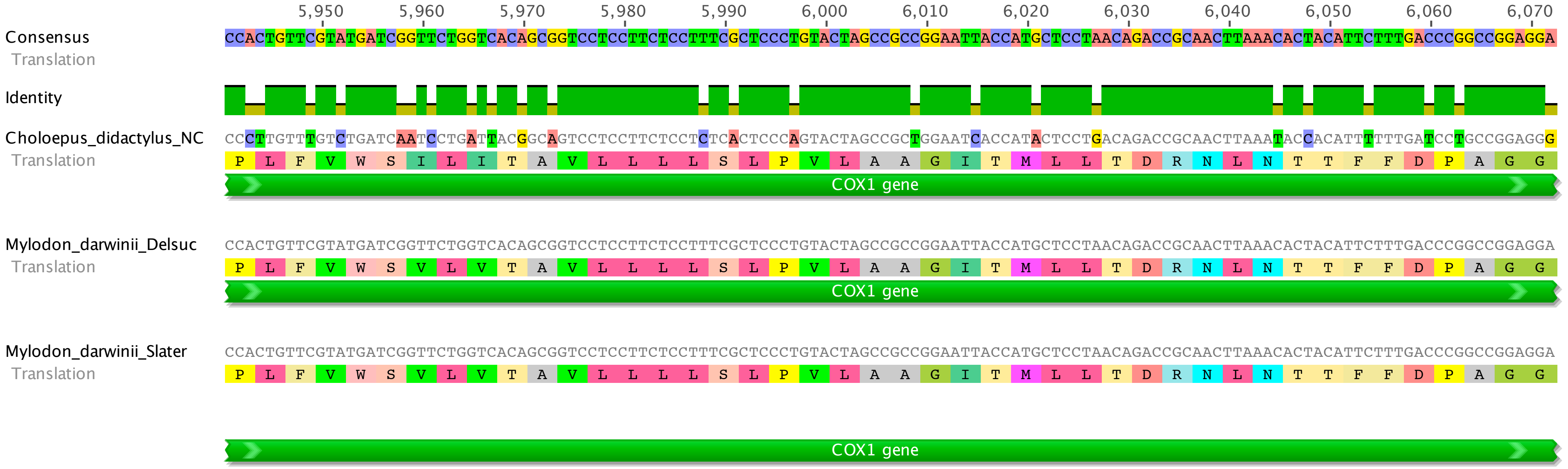
5,950 5,960 5,970 5,980 5,990 6,000 6,010 6,020 6,030 6,040 6,050 6,060 6,070
Consensus
CCACTGTTCGTATGATCGGTTCTGGTCACAGCGGTCCTCCTTCTCCTTTCGCTCCCTGTACTAGCCGCCGGAATTACCATGCTCCTAACAGACCGCAACTTAAACACTACATTCTTTGACCCGGCCGGAGGA
Translation
Identity
Choloepus_didactylus_NC
CCCTTGTTTGTCTGATCAATCCTGATTACGGCAGTCCTCCTTCTCCTCTCACTCCCAGTACTAGCCGCTGGAATCACCATACTCCTGACAGACCGCAACTTAAATACCACATTTTTTGATCCTGCCGGAGGG
Translation
P L F V W S I L I T A V L L L L S L P V L A A G I T M L L T D R N L N T T F F D P A G G
COX1 gene
Mylodon_darwinii_Delsuc
CCACTGTTCGTATGATCGGTTCTGGTCACAGCGGTCCTCCTTCTCCTTTCGCTCCCTGTACTAGCCGCCGGAATTACCATGCTCCTAACAGACCGCAACTTAAACACTACATTCTTTGACCCGGCCGGAGGA
Translation
P L F V W S V L V T A V L L L L S L P V L A A G I T M L L T D R N L N T T F F D P A G G
COX1 gene
Mylodon_darwinii_Slater
CCACTGTTCGTATGATCGGTTCTGGTCACAGCGGTCCTCCTTCTCCTTTCGCTCCCTGTACTAGCCGCCGGAATTACCATGCTCCTAACAGACCGCAACTTAAACACTACATTCTTTGACCCGGCCGGAGGA
Translation
P L F V W S V L V T A V L L L L S L P V L A A G I T M L L T D R N L N T T F F D P A G G
COX1 gene

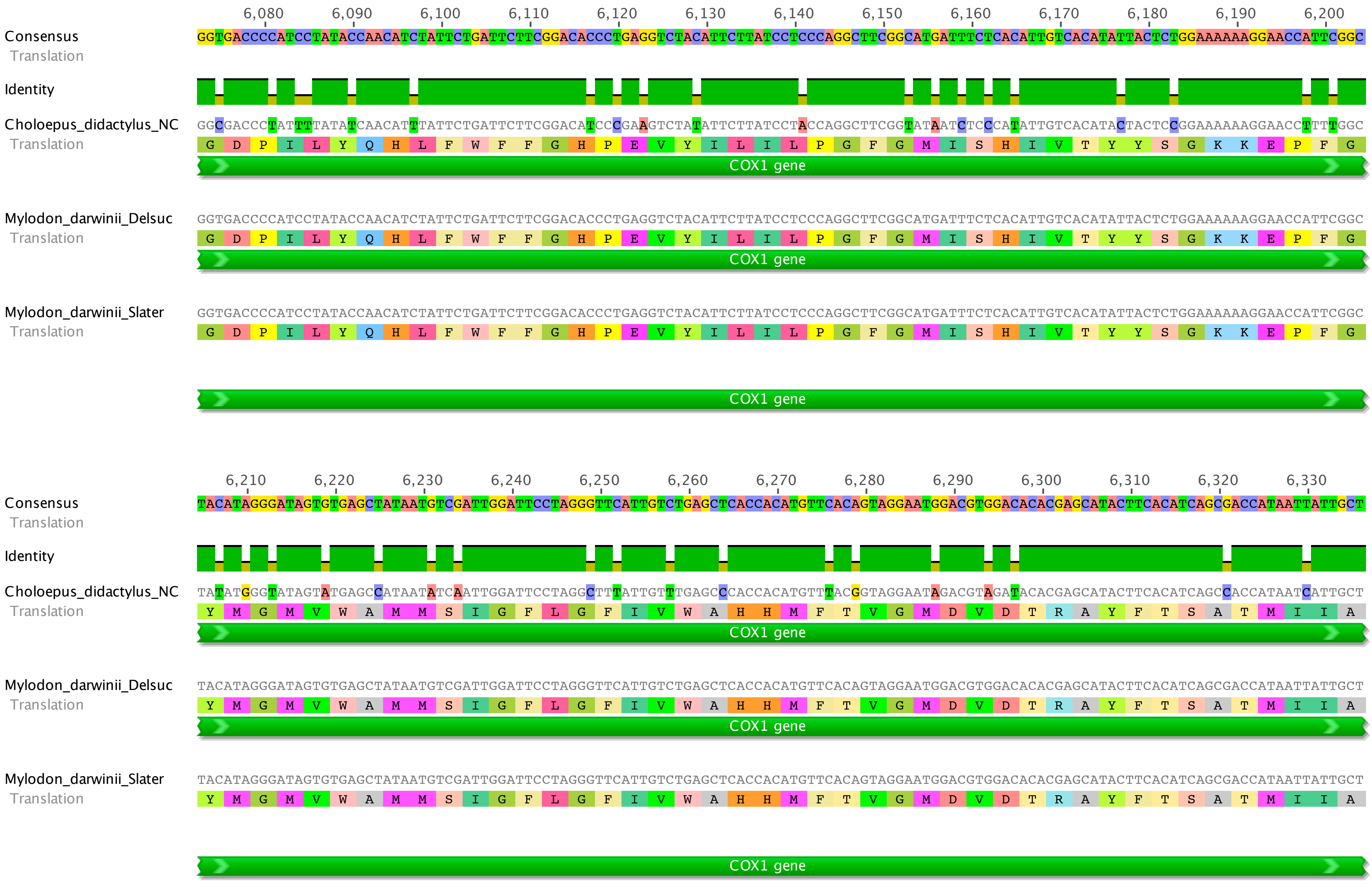
6,080 6,090 6,100 6,110 6,120 6,130 6,140 6,150 6,160 6,170 6,180 6,190 6,200
Consensus
GGTGACCCCATCCTATACCAACATCTATTCTGATTCTTCGGACACCCTGAGGTCTACATTCTTATCCTCCCAGGCTTCGGCATGATTTCTCACATTGTCACATATTACTCTGGAAAAAGGAACCATTCGGC
Translation
Identity
Choloepus_didactylus_NC
GGCGACCCTATTTTATATCAACATTTATTCTGATTCTTCGGACATCCCGAAGTCTATATTCTTATCCTACCAGGCTTCGGTATAATCTCCCATATTGTCACATACTACTCCGGAAAAAGGAACCTTTTGGC
Translation
G D P I L Y Q H L F W F F G H P E V Y I L I L P G F G M I S H I V T Y Y S G K K E P F G
COX1 gene
Mylodon_darwinii_Delsuc
GGTGACCCCATCCTATACCAACATCTATTCTGATTCTTCGGACACCCTGAGGTCTACATTCTTATCCTCCCAGGCTTCGGCATGATTTCTCACATTGTCACATATTACTCTGGAAAAAGGAACCATTCGGC
Translation
G D P I L Y Q H L F W F F G H P E V Y I L I L P G F G M I S H I V T Y Y S G K K E P F G
COX1 gene
Mylodon_darwinii_Slater
GGTGACCCCATCCTATACCAACATCTATTCTGATTCTTCGGACACCCTGAGGTCTACATTCTTATCCTCCCAGGCTTCGGCATGATTTCTCACATTGTCACATATTACTCTGGAAAAAGGAACCATTCGGC
Translation
G D P I L Y Q H L F W F F G H P E V Y I L I L P G F G M I S H I V T Y Y S G K K E P F G
COX1 gene
6,210 6,220 6,230 6,240 6,250 6,260 6,270 6,280 6,290 6,300 6,310 6,320 6,330
Consensus
TACATAGGGATAGTGTGAGCTATAATGTCGATTGGATTCCTAGGGTTCATTGTCTGAGCTCACCACATGTTCACAGTAGGAATGGACGTGGACACACGAGCATACTTCACATCAGCGACCATAATTATTGCT
Translation
Identity
Choloepus_didactylus_NC
TATATGGGTATAGTATGAGCCATAATATCAATTGGATTCCTAGGCTTTATTGTTTGAGCCCACCACATGTTTACGGTAGGAATAGACGTAGATACACGAGCATACTTCACATCAGCCACCATAATCATTGCT
Translation
Y M G M V W A M M S I G F L G F I V W A H H M F T V G M D V D T R A Y F T S A T M I I A
COX1 gene
Mylodon_darwinii_Delsuc
TACATAGGGATAGTGTGAGCTATAATGTCGATTGGATTCCTAGGGTTCATTGTCTGAGCTCACCACATGTTCACAGTAGGAATGGACGTGGACACACGAGCATACTTCACATCAGCGACCATAATTATTGCT
Translation
Y M G M V W A M M S I G F L G F I V W A H H M F T V G M D V D T R A Y F T S A T M I I A
COX1 gene
Mylodon_darwinii_Slater
TACATAGGGATAGTGTGAGCTATAATGTCGATTGGATTCCTAGGGTTCATTGTCTGAGCTCACCACATGTTCACAGTAGGAATGGACGTGGACACACGAGCATACTTCACATCAGCGACCATAATTATTGCT
Translation
Y M G M V W A M M S I G F L G F I V W A H H M F T V G M D V D T R A Y F T S A T M I I A
COX1 gene

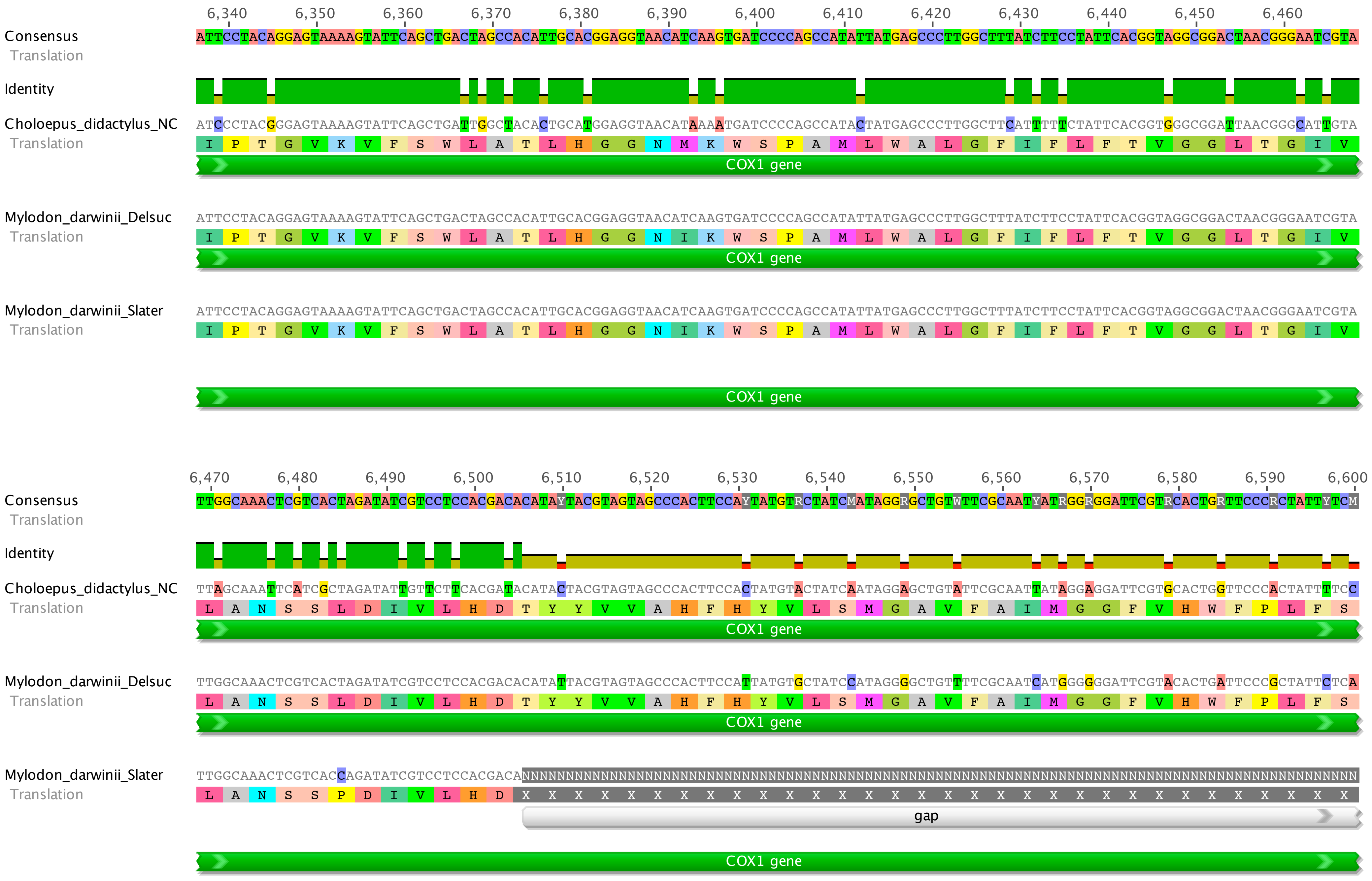

6,340 6,350 6,360 6,370 6,380 6,390 6,400 6,410 6,420 6,430 6,440 6,450 6,460
Consensus
Translation
Identity
Choloepus_didactylus_NC
Translation
COX1 gene
Mylodon_darwinii_Delsuc
Translation
COX1 gene
Mylodon_darwinii_Slater
Translation
COX1 gene
6,470 6,480 6,490 6,500 6,510 6,520 6,530 6,540 6,550 6,560 6,570 6,580 6,590 6,600
Consensus
Translation
Identity
Choloepus_didactylus_NC
Translation
COX1 gene
Mylodon_darwinii_Delsuc
Translation
COX1 gene
Mylodon_darwinii_Slater
Translation
gap
COX1 gene

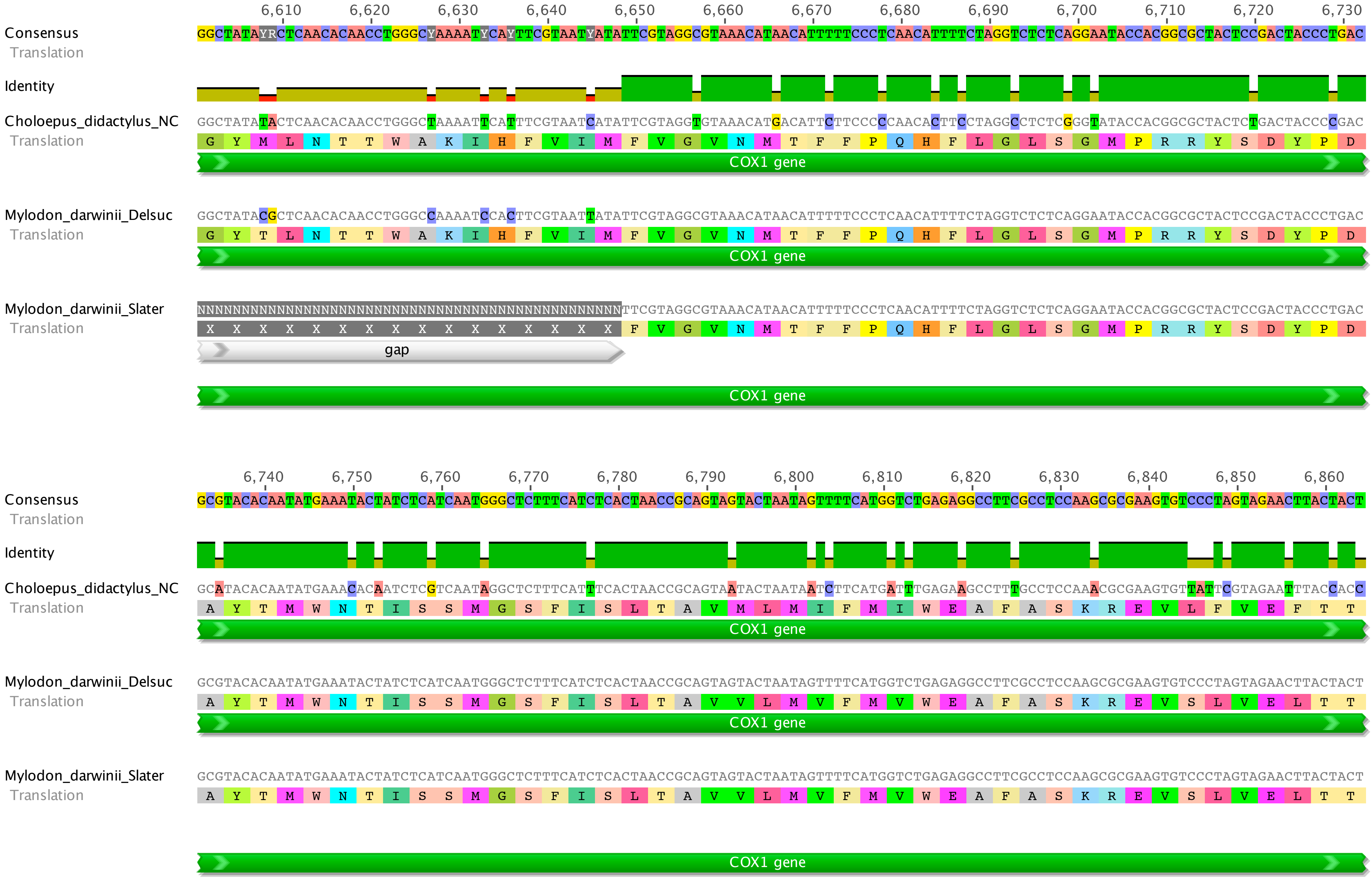
6,610
6,620
6,630
6,640
6,650
6,660
6,670
6,680
6,690
6,700
6,710
6,720
6,730
Consensus
Translation
Identity
Choloepus_didactylus_NC
Translation
COX1 gene
Mylodon_darwinii_Delsuc
Translation
COX1 gene
Mylodon_darwinii_Slater
Translation
gap
COX1 gene
6,740
6,750
6,760
6,770
6,780
6,790
6,800
6,810
6,820
6,830
6,840
6,850
6,860
Consensus
Translation
Identity
Choloepus_didactylus_NC
Translation
COX1 gene
Mylodon_darwinii_Delsuc
Translation
COX1 gene
Mylodon_darwinii_Slater
Translation
COX1 gene

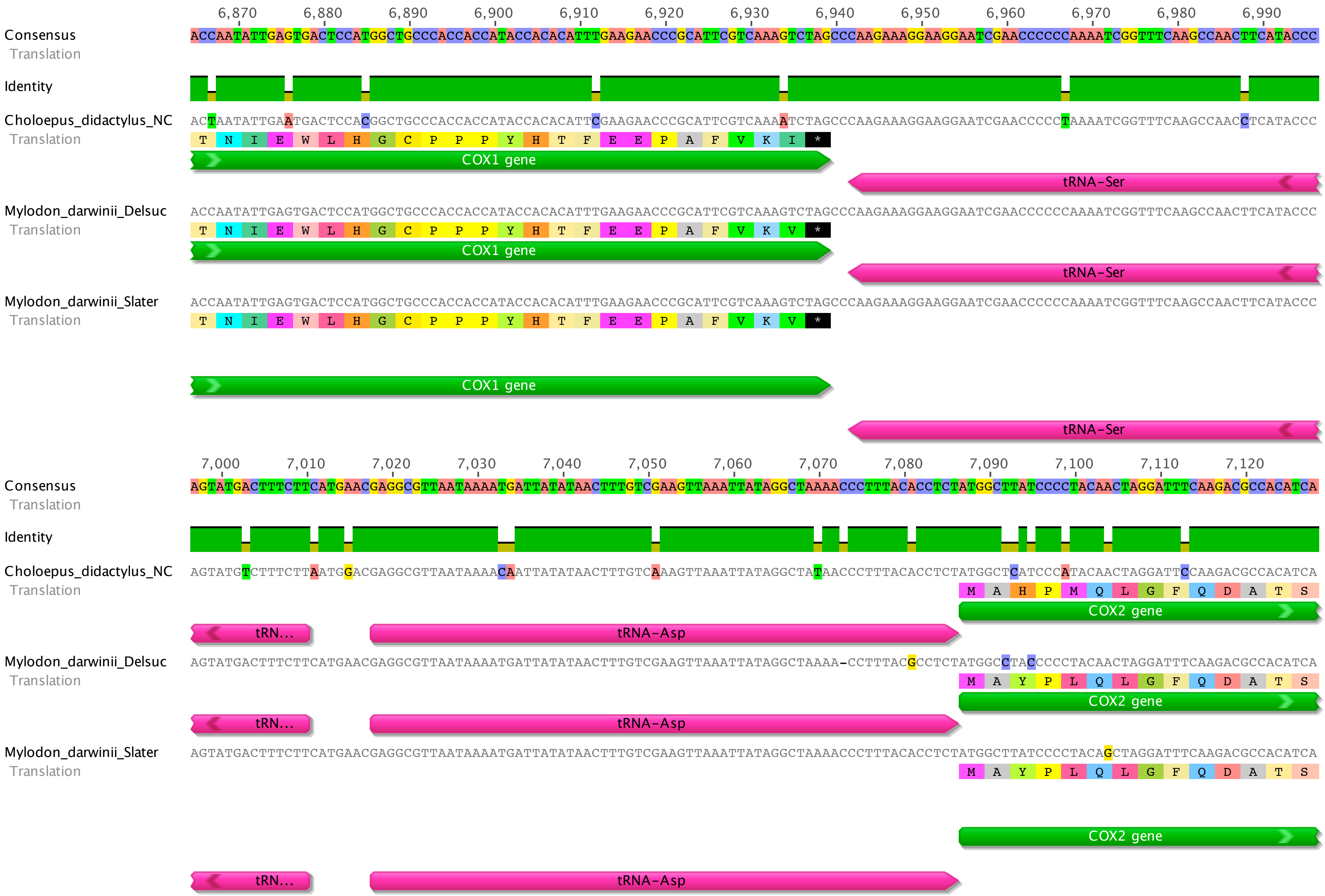

6,870 6,880 6,890 6,900 6,910 6,920 6,930 6,940 6,950 6,960 6,970 6,980 6,990
Consensus
Translation
Identity
Choloepus_didactylus_NC
Translation
COX1 gene
tRNA-Ser
Mylodon_darwinii_Delsuc
Translation
COX1 gene
tRNA-Ser
Mylodon_darwinii_Slater
Translation
COX1 gene
tRNA-Ser
7,000 7,010 7,020 7,030 7,040 7,050 7,060 7,070 7,080 7,090 7,100 7,110 7,120
Consensus
Translation
Identity
Choloepus_didactylus_NC
Translation
COX2 gene
tRN...
tRNA-Asp
Mylodon_darwinii_Delsuc
Translation
COX2 gene
tRN...
tRNA-Asp
Mylodon_darwinii_Slater
Translation
COX2 gene
tRN...
tRNA-Asp

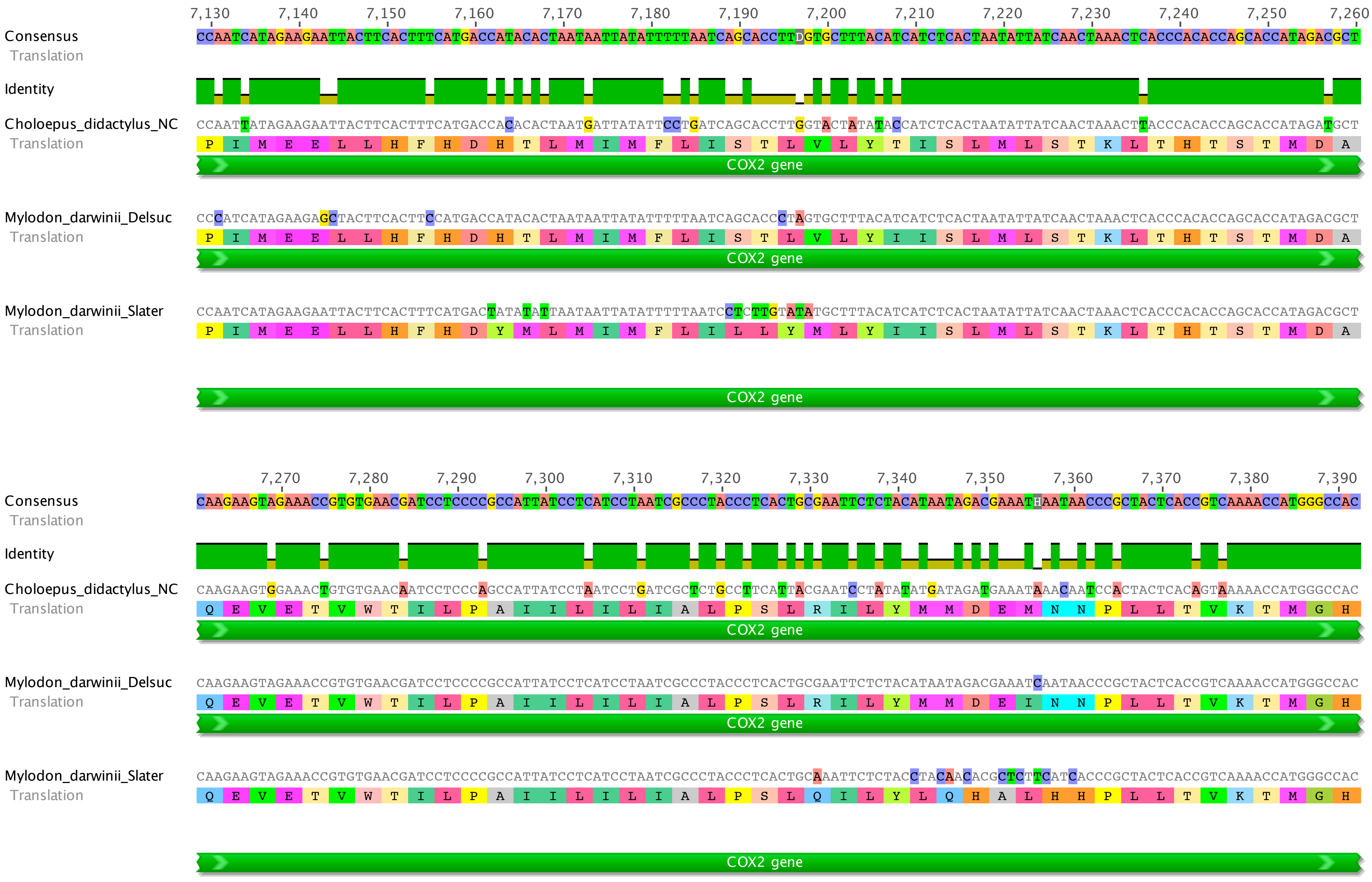

7,130 7,140 7,150 7,160 7,170 7,180 7,190 7,200 7,210 7,220 7,230 7,240 7,250 7,260
Consensus
Translation
Identity
Choloepus_didactylus_NC
Translation
COX2 gene
Mylodon_darwinii_Delsuc
Translation
COX2 gene
Mylodon_darwinii_Slater
Translation
COX2 gene
7,270 7,280 7,290 7,300 7,310 7,320 7,330 7,340 7,350 7,360 7,370 7,380 7,390
Consensus
Translation
Identity
Choloepus_didactylus_NC
Translation
COX2 gene
Mylodon_darwinii_Delsuc
Translation
COX2 gene
Mylodon_darwinii_Slater
Translation
COX2 gene

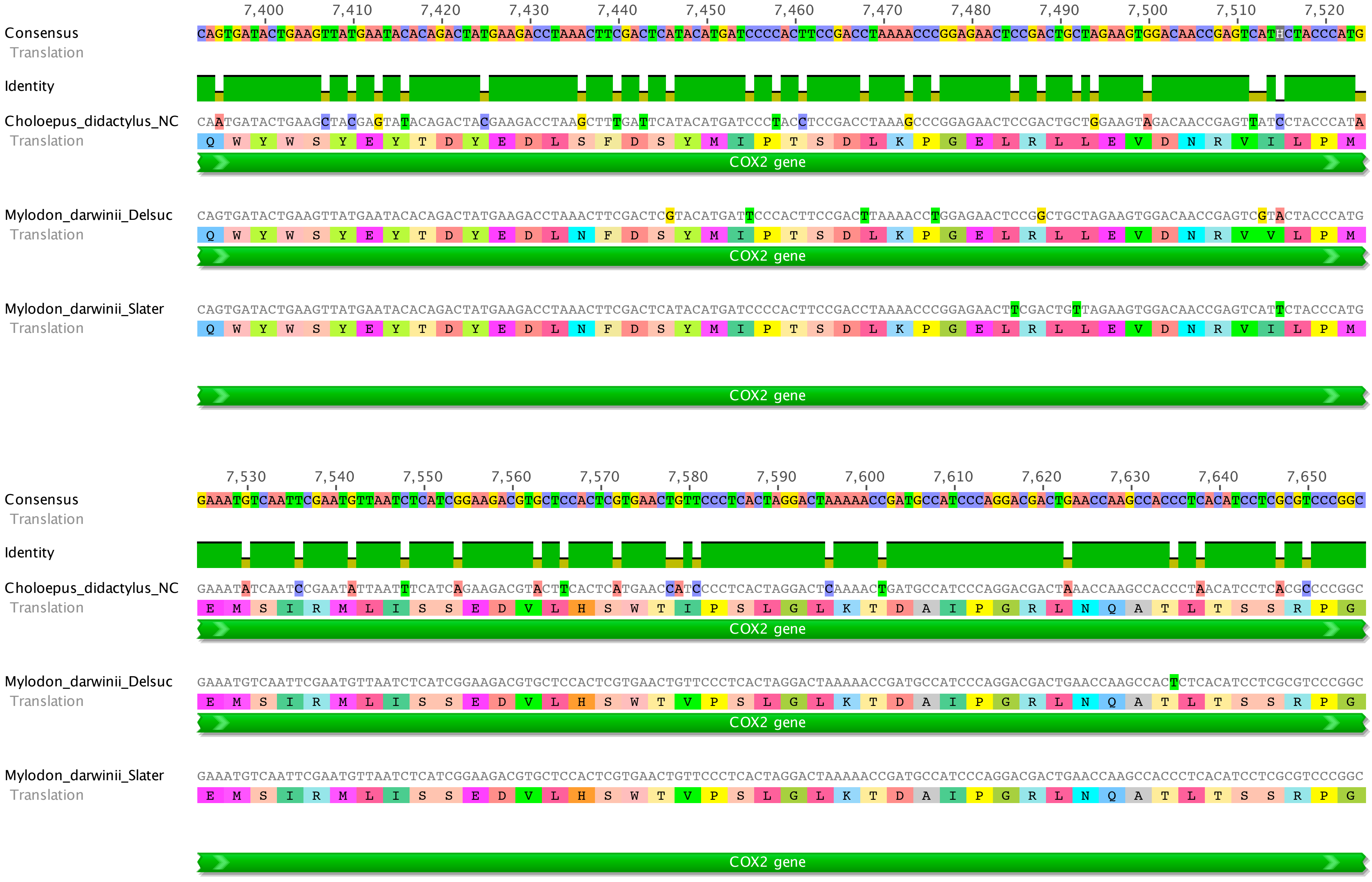
7,400 7,410 7,420 7,430 7,440 7,450 7,460 7,470 7,480 7,490 7,500 7,510 7,520
Consensus
CAGTGATACTGAAGTTATGAATACACAGACTATGAAGACCTAAACTTCGACTCATACATGATCCCCACTTCCGACCTAAAACCCGGAGAACTCCGACTGCTAGAAGTGGACAACCGAGTCATHCTACCCATG
Translation
Identity
Choloepus_didactylus_NC
CAATGATACTGAAGCTACGAGTATACAGACTACGAAGACCTAAGCTTTGATTCATACATGATCCCTACCTCCGACCTAAAGCCCGGAGAACTCCGACTGCTGGAAGTAGACAACCGAGTTATCCTACCCATA
Translation
Q W Y W S Y E Y T D Y E D L S F D S Y M I P T S D L K P G E L R L L E V D N R V I L P M
COX2 gene
Mylodon_darwinii_Delsuc
CAGTGATACTGAAGTTATGAATACACAGACTATGAAGACCTAAACTTCGACTCGTACATGATTCCCACTTCCGACTTAAAACCTGGAGAACTCCGGCTGCTAGAAGTGGACAACCGAGTCGTACTACCCATG
Translation
Q W Y W S Y E Y T D Y E D L N F D S Y M I P T S D L K P G E L R L L E V D N R V V L P M
COX2 gene
Mylodon_darwinii_Slater
CAGTGATACTGAAGTTATGAATACACAGACTATGAAGACCTAAACTTCGACTCATACATGATCCCCACTTCCGACCTAAAACCCGGAGAACTTCGACTGTTAGAAGTGGACAACCGAGTCATTCTACCCATG
Translation
Q W Y W S Y E Y T D Y E D L N F D S Y M I P T S D L K P G E L R L L E V D N R V I L P M
COX2 gene
7,530 7,540 7,550 7,560 7,570 7,580 7,590 7,600 7,610 7,620 7,630 7,640 7,650
Consensus
GAAATGTCAATTCGAATGTTAATCTCATCGGAAGACGTGCTCCACTCGTGAACTGTTCCCTCACTAGGACTAAAAACCGATGCCATCCCAGGACGACTGAACCAAGCCACCCTCACATCCTCGCGTCCCGGC
Translation
Identity
Choloepus_didactylus_NC
GAAATATCAATCCGAATATTAATTTCATCAGAAGACGTACTTCACTCATGAACCATCCCCTCACTAGGACTCAAAACTGATGCCATCCCAGGACGACTAAACCAAGCCACCCTAACATCCTCACGCCCCGGC
Translation
E M S I R M L I S S E D V L H S W T I P S L G L K T D A I P G R L N Q A T L T S S R P G
COX2 gene
Mylodon_darwinii_Delsuc
GAAATGTCAATTCGAATGTTAATCTCATCGGAAGACGTGCTCCACTCGTGAACTGTTCCCTCACTAGGACTAAAAACCGATGCCATCCCAGGACGACTGAACCAAGCCACTCTCACATCCTCGCGTCCCGGC
Translation
E M S I R M L I S S E D V L H S W T V P S L G L K T D A I P G R L N Q A T L T S S R P G
COX2 gene
Mylodon_darwinii_Slater
GAAATGTCAATTCGAATGTTAATCTCATCGGAAGACGTGCTCCACTCGTGAACTGTTCCCTCACTAGGACTAAAAACCGATGCCATCCCAGGACGACTGAACCAAGCCACCCTCACATCCTCGCGTCCCGGC
Translation
E M S I R M L I S S E D V L H S W T V P S L G L K T D A I P G R L N Q A T L T S S R P G
COX2 gene

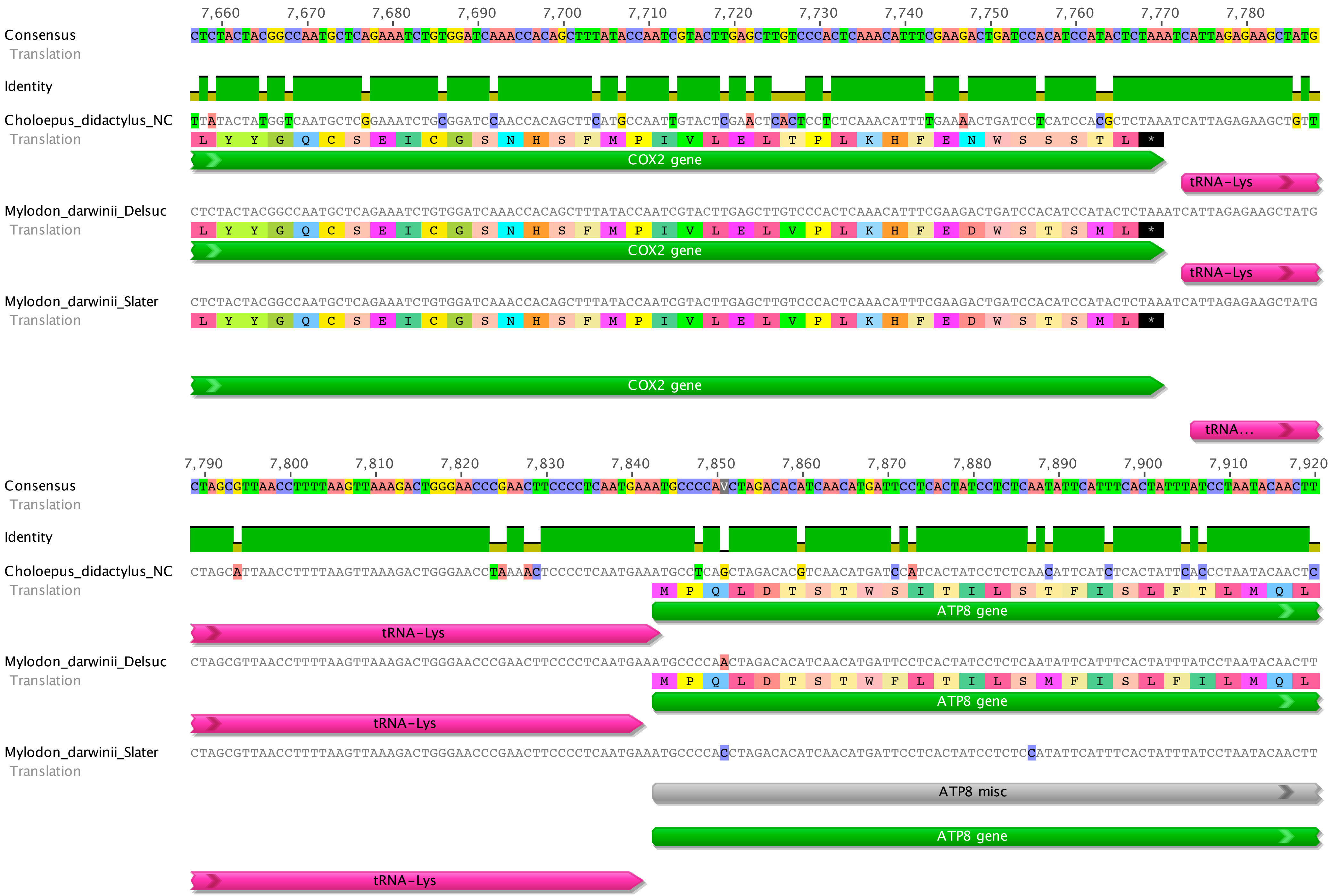
7,660 7,670 7,680 7,690 7,700 7,710 7,720 7,730 7,740 7,750 7,760 7,770 7,780
Consensus
Translation
Identity
Choloepus_didactylus_NC
Translation
COX2 gene
tRNA-Lys
Mylodon_darwinii_Delsuc
Translation
COX2 gene
tRNA-Lys
Mylodon_darwinii_Slater
Translation
COX2 gene
tRNA...
7,790 7,800 7,810 7,820 7,830 7,840 7,850 7,860 7,870 7,880 7,890 7,900 7,910 7,920
Consensus
Translation
Identity
Choloepus_didactylus_NC
Translation
ATP8 gene
tRNA-Lys
Mylodon_darwinii_Delsuc
Translation
ATP8 gene
tRNA-Lys
Mylodon_darwinii_Slater
Translation
ATP8 misc
ATP8 gene
tRNA-Lys

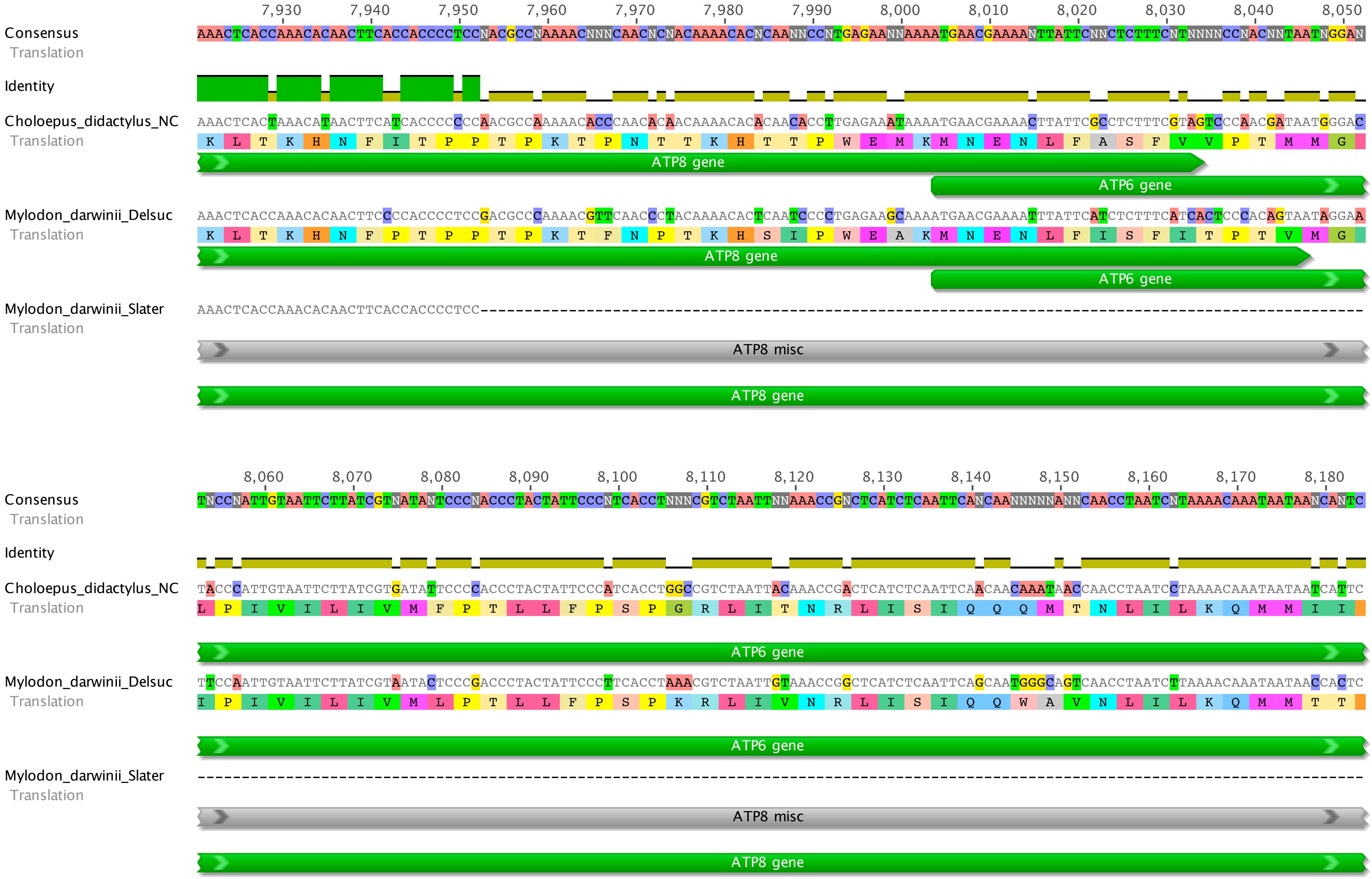

7,930 7,940 7,950 7,960 7,970 7,980 7,990 8,000 8,010 8,020 8,030 8,040 8,050
Consensus
Translation
Identity
Choloepus_didactylus_NC
Translation
ATP8 gene
ATP6 gene
Mylodon_darwinii_Delsuc
Translation
ATP8 gene
ATP6 gene
Mylodon_darwinii_Slater
AAACTCACCAAACACAACTTCACCACCCCTCC
Translation
ATP8 misc
ATP8 gene
8,060 8,070 8,080 8,090 8,100 8,110 8,120 8,130 8,140 8,150 8,160 8,170 8,180
Consensus
Translation
Identity
Choloepus_didactylus_NC
Translation
ATP6 gene
Mylodon_darwinii_Delsuc
Translation
ATP6 gene
Mylodon_darwinii_Slater
Translation
ATP8 misc
ATP8 gene

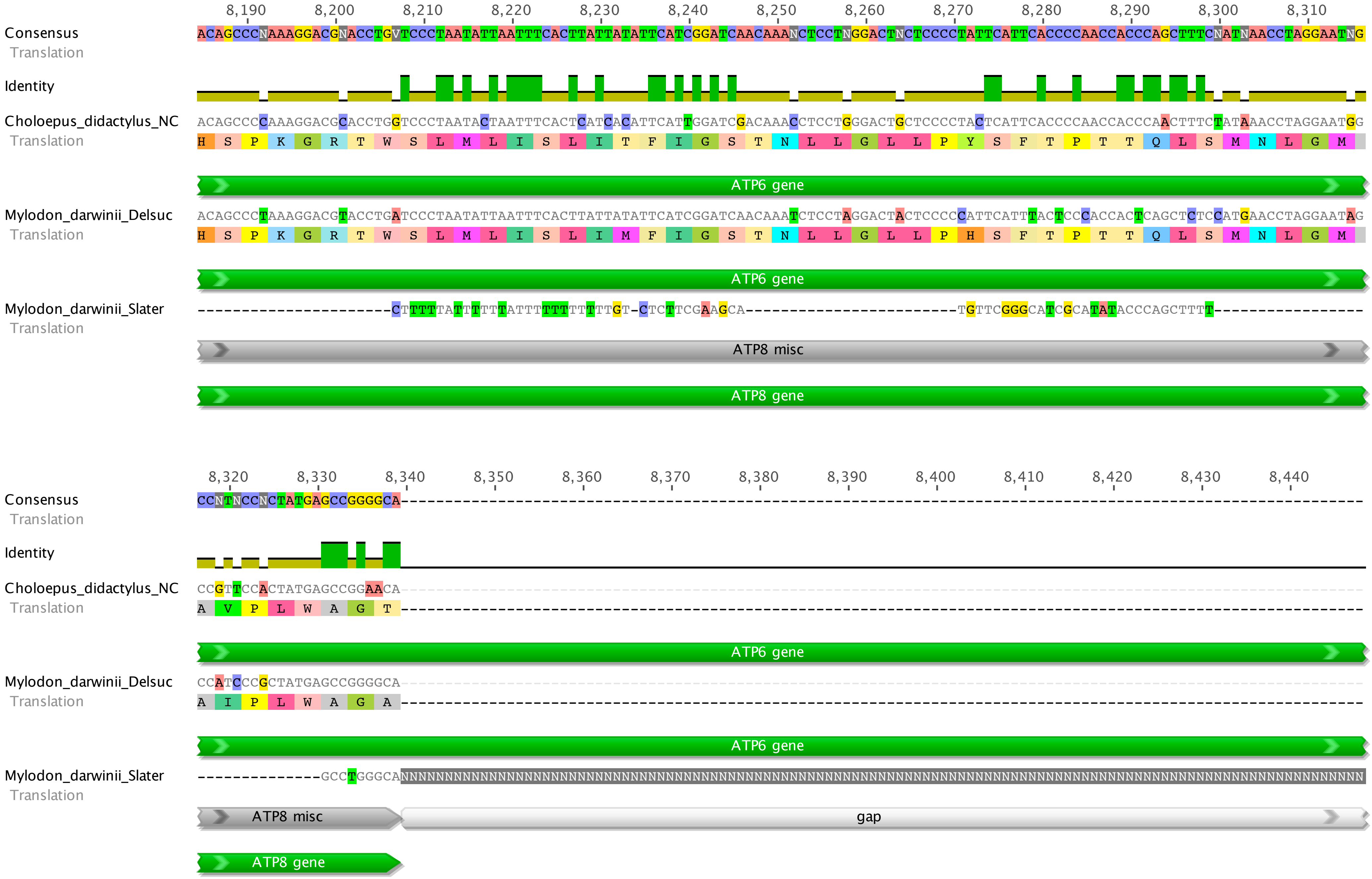
8,190
8,200
8,210
8,220
8,230
8,240
8,250
8,260
8,270
8,280
8,290
8,300
8,310
Consensus
Translation
Identity
Choloepus_didactylus_NC
Translation
ATP6 gene
Mylodon_darwinii_Delsuc
Translation
ATP6 gene
Mylodon_darwinii_Slater
Translation
ATP8 misc
ATP8 gene
8,320
8,330
8,340
8,350
8,360
8,370
8,380
8,390
8,400
8,410
8,420
8,430
8,440
Consensus
Translation
Identity
Choloepus_didactylus_NC
Translation
ATP6 gene
Mylodon_darwinii_Delsuc
Translation
ATP6 gene
Mylodon_darwinii_Slater
Translation
ATP8 misc
gap
ATP8 gene

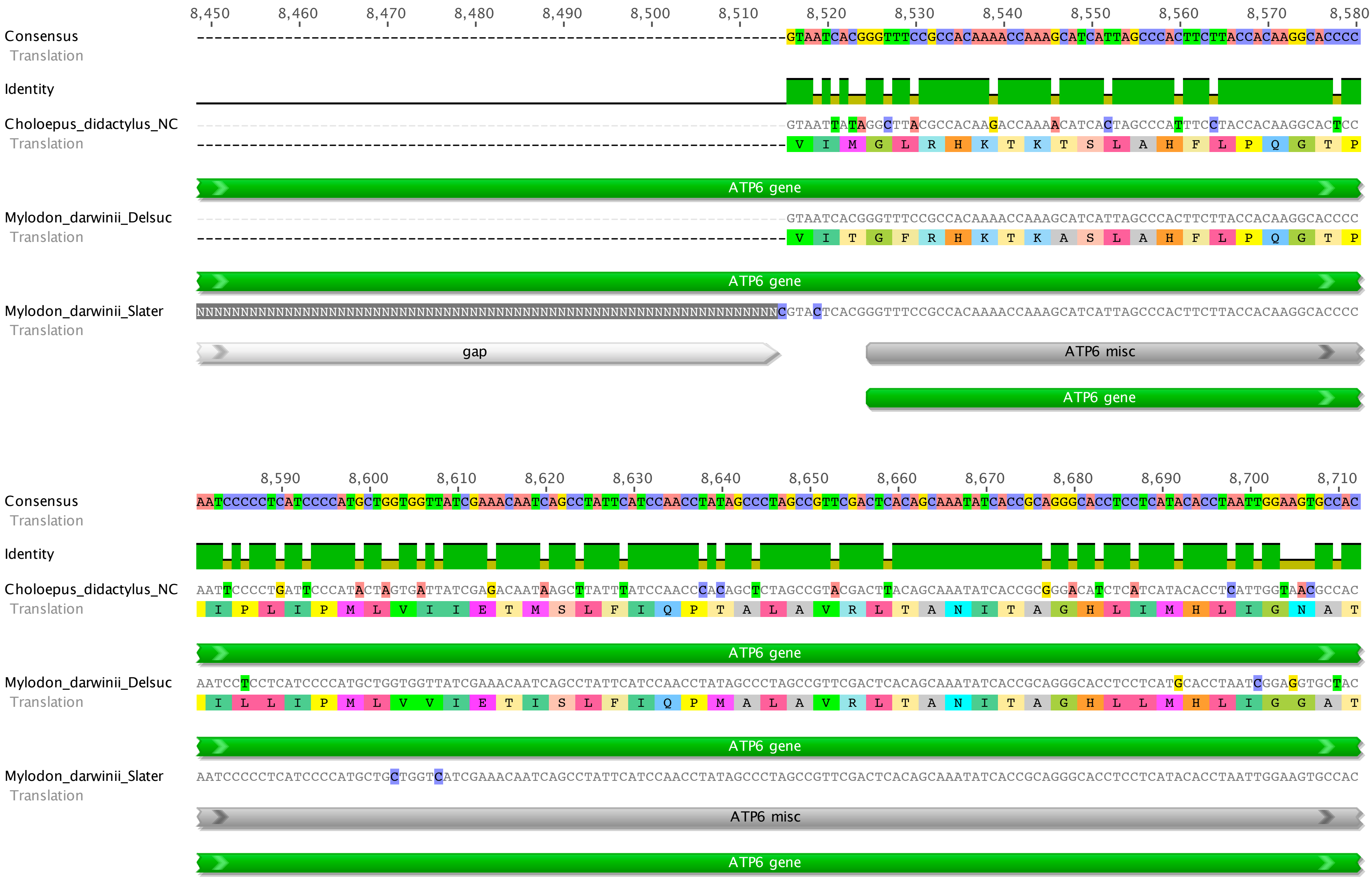
8,450 8,460 8,470 8,480 8,490 8,500 8,510 8,520 8,530 8,540 8,550 8,560 8,570 8,580
Consensus GTAATCACGGGTTTCCGCCACAAAACCAAAGCATCATTAGCCCACTTCTTACCACAAGGCACCCC
Translation
Identity
Choloepus_didactylus_NC GTAATTATAGGCTTACGCCACAAGACCAAAACATCACTAGCCCATTTCCTACCACAAGGCACTCC
Translation V I M G L R H K T K T S L A H F L P Q G T P
ATP6 gene
Mylodon_darwinii_Delsuc GTAATCACGGGTTTCCGCCACAAAACCAAAGCATCATTAGCCCACTTCTTACCACAAGGCACCCC
Translation V I T G F R H K T K A S L A H F L P Q G T P
ATP6 gene
Mylodon_darwinii_Slater CGTACTCACGGGTTTCCGCCACAAAACCAAAGCATCATTAGCCCACTTCTTACCACAAGGCACCCC
Translation
gap
ATP6 misc
ATP6 gene
8,590 8,600 8,610 8,620 8,630 8,640 8,650 8,660 8,670 8,680 8,690 8,700 8,710
Consensus AATCCCCCTCATCCCCATGCTGGTGGTTATCGAAACAATCAGCCTATTCATCCAACCTATAGCCCTAGCCGTTCGACTCACAGCAAATATCACCGCAGGGCACCTCCTCATACACCTAATTGGAAGTGCCAC
Translation
Identity
Choloepus_didactylus_NC AATTCCCCTGATTCCCATACTAGTGATTATCGAGACAATAAGCTTATTTATCCAACCCACAGCTCTAGCCGTACGACTTACAGCAAATATCACCGCGGGACATCTCATCATACACCTCATTGGTAACGCCAC
Translation I P L I P M L V I I E T M S L F I Q P T A L A V R L T A N I T A G H L I M H L I G N A T
ATP6 gene
Mylodon_darwinii_Delsuc AATCCTCCTCATCCCCATGCTGGTGGTTATCGAAACAATCAGCCTATTCATCCAACCTATAGCCCTAGCCGTTCGACTCACAGCAAATATCACCGCAGGGCACCTCCTCATGCACCTAATCGGAGGTGCTAC
Translation I L L I P M L V V I E T I S L F I Q P M A L A V R L T A N I T A G H L L M H L I G G A T
ATP6 gene
Mylodon_darwinii_Slater AATCCCCCTCATCCCCATGCTGCTGGTCATCGAAACAATCAGCCTATTCATCCAACCTATAGCCCTAGCCGTTCGACTCACAGCAAATATCACCGCAGGGCACCTCCTCATACACCTAATTGGAAGTGCCAC
Translation
ATP6 misc
ATP6 gene

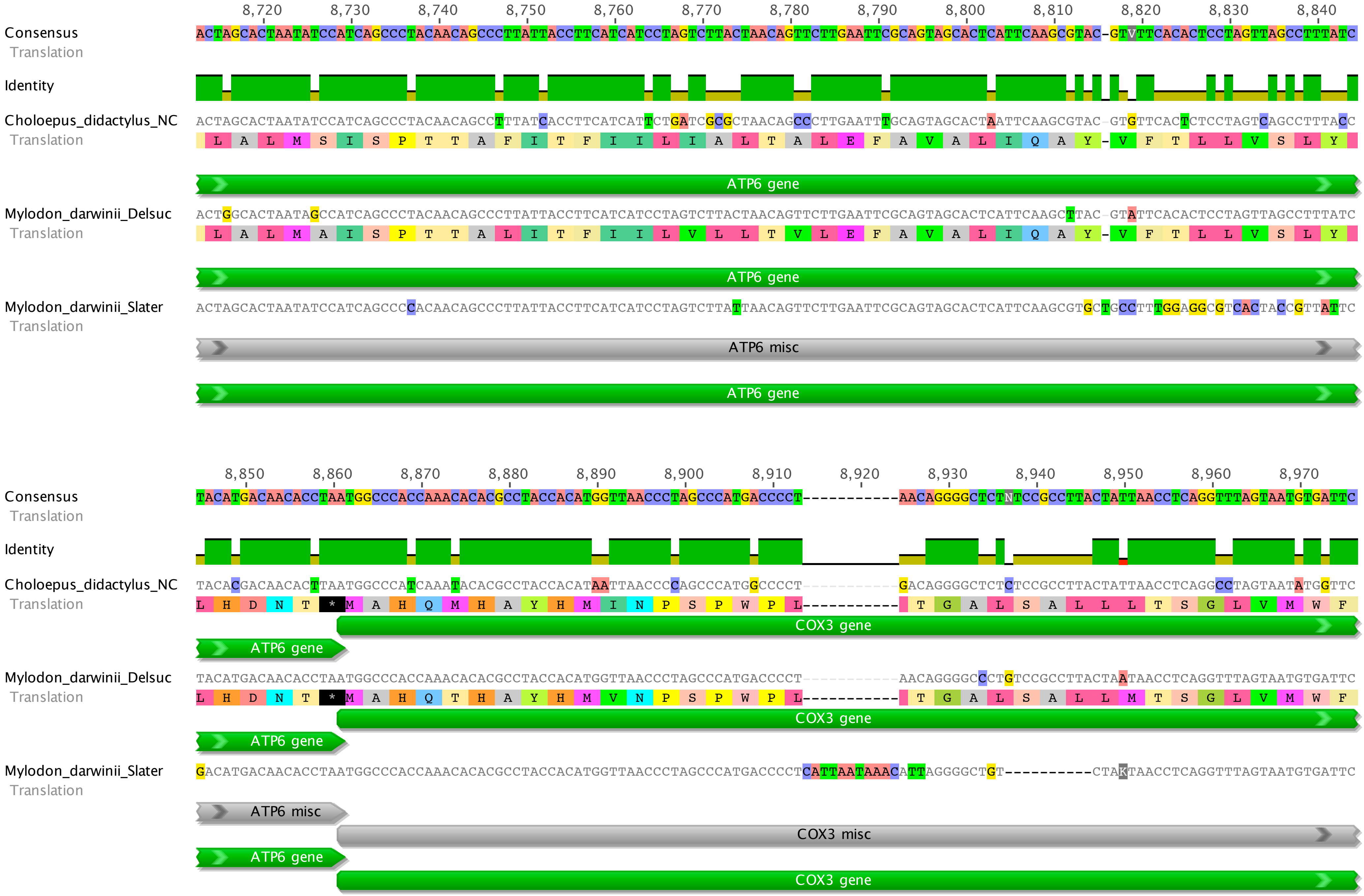

8,720
8,730
8,740
8,750
8,760
8,770
8,780
8,790
8,800
8,810
8,820
8,830
8,840
Consensus
Translation
Identity
Choloepus_didactylus_NC
Translation
ATP6 gene
Mylodon_darwinii_Delsuc
Translation
ATP6 gene
Mylodon_darwinii_Slater
Translation
ATP6 misc
ATP6 gene
8,850
8,860
8,870
8,880
8,890
8,900
8,910
8,920
8,930
8,940
8,950
8,960
8,970
Consensus
Translation
Identity
Choloepus_didactylus_NC
Translation
COX3 gene
ATP6 gene
Mylodon_darwinii_Delsuc
Translation
COX3 gene
ATP6 gene
Mylodon_darwinii_Slater
Translation
ATP6 misc
COX3 misc
ATP6 gene
COX3 gene

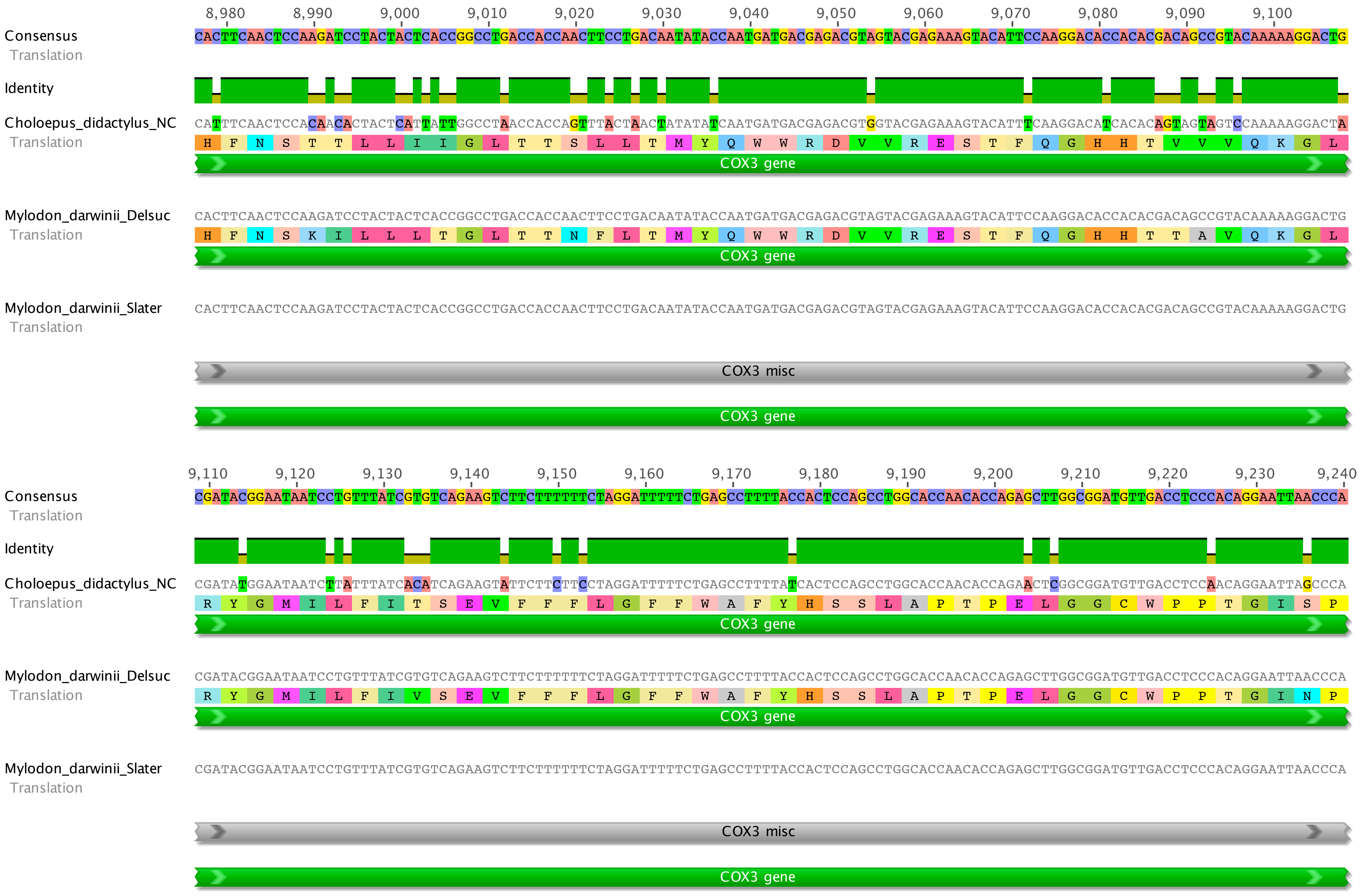
8,980 8,990 9,000 9,010 9,020 9,030 9,040 9,050 9,060 9,070 9,080 9,090 9,100
Consensus
CACTTCAACTCCAAGATCCTACTACTCACCGGCCTGACCACCAACTTCCTGACAATATACCAATGATGACGAGACGTAGTACGAGAAAGTACATTCCAAGGACACCACACGACAGCCGTACAAAAAGGACTG
Translation
Identity
Choloepus_didactylus_NC
CATTTCAACTCCACAACACTACTCATTATTGGCCTAACCACCAGTTTACTAACTATATATCAATGATGACGAGACGTGGTACGAGAAAGTACATTTCAAGGACATCACACAGTAGTAGTCCAAAAAGGACTA
Translation
H F N S T T L L I I G L T T S L L T M Y Q W W R D V V R E S T F Q G H H T V V V Q K G L
COX3 gene
Mylodon_darwinii_Delsuc
CACTTCAACTCCAAGATCCTACTACTCACCGGCCTGACCACCAACTTCCTGACAATATACCAATGATGACGAGACGTAGTACGAGAAAGTACATTCCAAGGACACCACACGACAGCCGTACAAAAAGGACTG
Translation
H F N S K I L L L T G L T T N F L T M Y Q W W R D V V R E S T F Q G H H T T A V Q K G L
COX3 gene
Mylodon_darwinii_Slater
CACTTCAACTCCAAGATCCTACTACTCACCGGCCTGACCACCAACTTCCTGACAATATACCAATGATGACGAGACGTAGTACGAGAAAGTACATTCCAAGGACACCACACGACAGCCGTACAAAAAGGACTG
Translation
COX3 misc
COX3 gene
9,110 9,120 9,130 9,140 9,150 9,160 9,170 9,180 9,190 9,200 9,210 9,220 9,230 9,240
Consensus
CGATACGGAATAATCCTGTTTATCGTGTCAGAAGTCTTCTTTTTTCTAGGATTTTTCTGAGCCTTTTACCACTCCAGCCTGGCACCAACACCAGAGCTTGGCGGATGTTGACCTCCCACAGGAATTAACCCA
Translation
Identity
Choloepus_didactylus_NC
CGATATGGAATAATCTTATTTATCACATCAGAAGTATTCTTCTTCCTAGGATTTTTCTGAGCCTTTTATCACTCCAGCCTGGCACCAACACCAGAACTCGGCGGATGTTGACCTCCAACAGGAATTAGCCCA
Translation
R Y G M I L F I T S E V F F F L G F F W A F Y H S S L A P T P E L G G C W P P T G I S P
COX3 gene
Mylodon_darwinii_Delsuc
CGATACGGAATAATCCTGTTTATCGTGTCAGAAGTCTTCTTTTTTCTAGGATTTTTCTGAGCCTTTTACCACTCCAGCCTGGCACCAACACCAGAGCTTGGCGGATGTTGACCTCCCACAGGAATTAACCCA
Translation
R Y G M I L F I V S E V F F F L G F F W A F Y H S S L A P T P E L G G C W P P T G I N P
COX3 gene
Mylodon_darwinii_Slater
CGATACGGAATAATCCTGTTTATCGTGTCAGAAGTCTTCTTTTTTCTAGGATTTTTCTGAGCCTTTTACCACTCCAGCCTGGCACCAACACCAGAGCTTGGCGGATGTTGACCTCCCACAGGAATTAACCCA
Translation
COX3 misc
COX3 gene

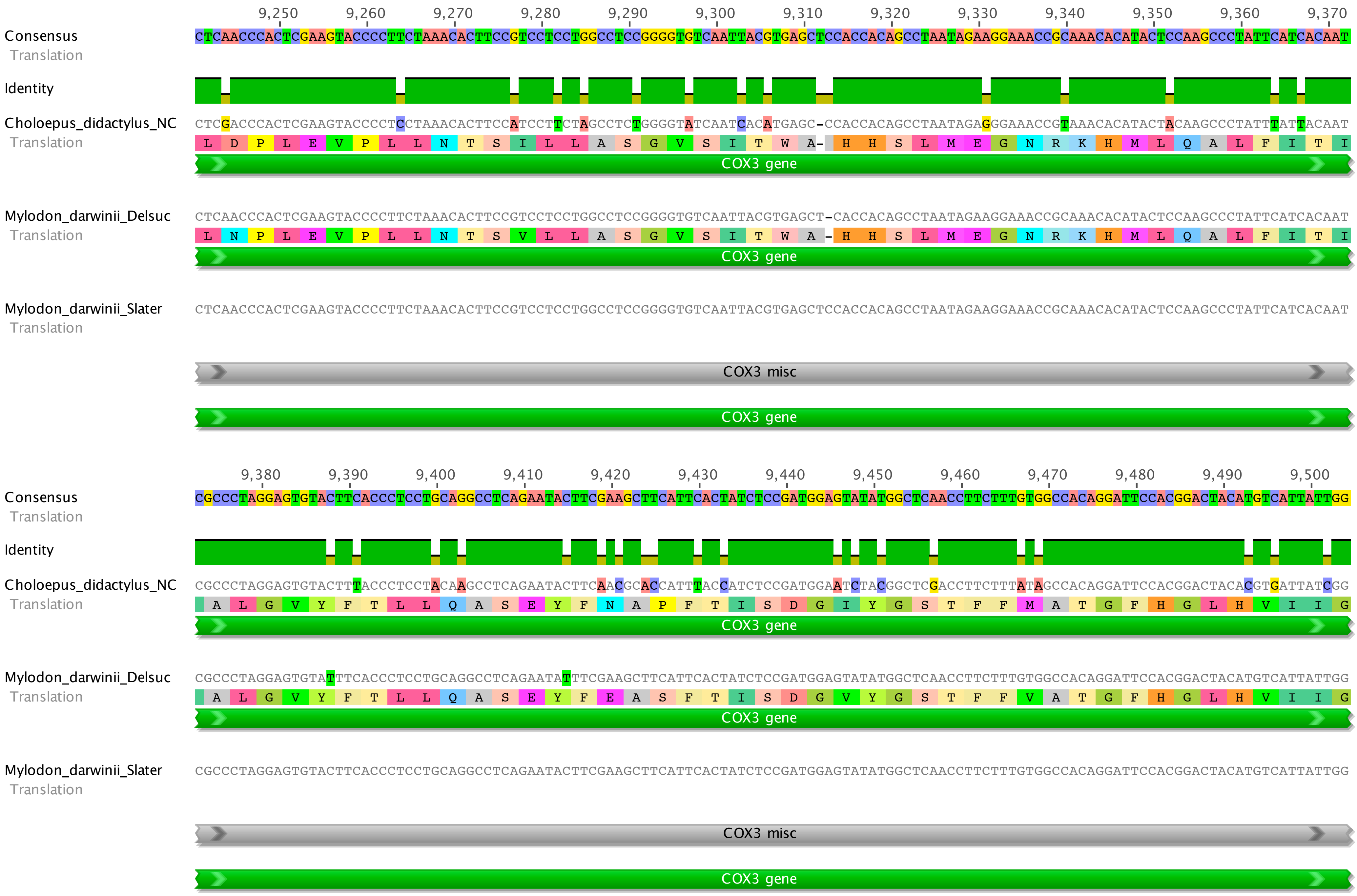
9,250 9,260 9,270 9,280 9,290 9,300 9,310 9,320 9,330 9,340 9,350 9,360 9,370
Consensus
CTCAACCCACTCGAAGTACCCCTTCTAAACACTTCCGTCCTCCTGGCCTCCGGGGTGTCAATTACGTGAGCTCCACCACAGCCTAATAGAAGGAAACCGCAAACACATACTCCAAGCCCTATTCATCACAAT
Translation
Identity
Choloepus_didactylus_NC
CTCGACCCACTCGAAGTACCCCTCCTAAACACTTCCATCCTTCTAGCCTCTGGGGTATCAATCACATGAGC-CCACCACAGCCTAATAGAGGGAAACCGTAAACACATACTACAAGCCCTATTTATTACAAT
Translation
L D P L E V P L L N T S I L L A S G V S I T W A- H H S L M E G N R K H M L Q A L F I T I
COX3 gene
Mylodon_darwinii_Delsuc
CTCAACCCACTCGAAGTACCCCTTCTAAACACTTCCGTCCTCCTGGCCTCCGGGGTGTCAATTACGTGAGCT-CACCACAGCCTAATAGAAGGAAACCGCAAACACATACTCCAAGCCCTATTCATCACAAT
Translation
L N P L E V P L L N T S V L L A S G V S I T W A - H H S L M E G N R K H M L Q A L F I T I
COX3 gene
Mylodon_darwinii_Slater
CTCAACCCACTCGAAGTACCCCTTCTAAACACTTCCGTCCTCCTGGCCTCCGGGGTGTCAATTACGTGAGCTCCACCACAGCCTAATAGAAGGAAACCGCAAACACATACTCCAAGCCCTATTCATCACAAT
Translation
COX3 misc
COX3 gene
9,380 9,390 9,400 9,410 9,420 9,430 9,440 9,450 9,460 9,470 9,480 9,490 9,500
Consensus
CGCCCTAGGAGTGTACTTCACCCTCCTGCAGGCCTCAGAATACTTCGAAGCTTCATTCACTATCTCCGATGGAGTATATGGCTCAACCTTCTTTGTGGCCACAGGATTCCACGGACTACATGTCATTATTGG
Translation
Identity
Choloepus_didactylus_NC
CGCCCTAGGAGTGTACTTTACCCTCCTACAAGCCTCAGAATACTTCAACGCACCATTTACCATCTCCGATGGAATCTACGGCTCGACCTTCTTTATAGCCACAGGATTCCACGGACTACACGTGATTATCGG
Translation
A L G V Y F T L L Q A S E Y F N A P F T I S D G I Y G S T F F M A T G F H G L H V I I G
COX3 gene
Mylodon_darwinii_Delsuc
CGCCCTAGGAGTGTATTTCACCCTCCTGCAGGCCTCAGAATATTTCGAAGCTTCATTCACTATCTCCGATGGAGTATATGGCTCAACCTTCTTTGTGGCCACAGGATTCCACGGACTACATGTCATTATTGG
Translation
A L G V Y F T L L Q A S E Y F E A S F T I S D G V Y G S T F F V A T G F H G L H V I I G
COX3 gene
Mylodon_darwinii_Slater
CGCCCTAGGAGTGTACTTCACCCTCCTGCAGGCCTCAGAATACTTCGAAGCTTCATTCACTATCTCCGATGGAGTATATGGCTCAACCTTCTTTGTGGCCACAGGATTCCACGGACTACATGTCATTATTGG
Translation
COX3 misc
COX3 gene

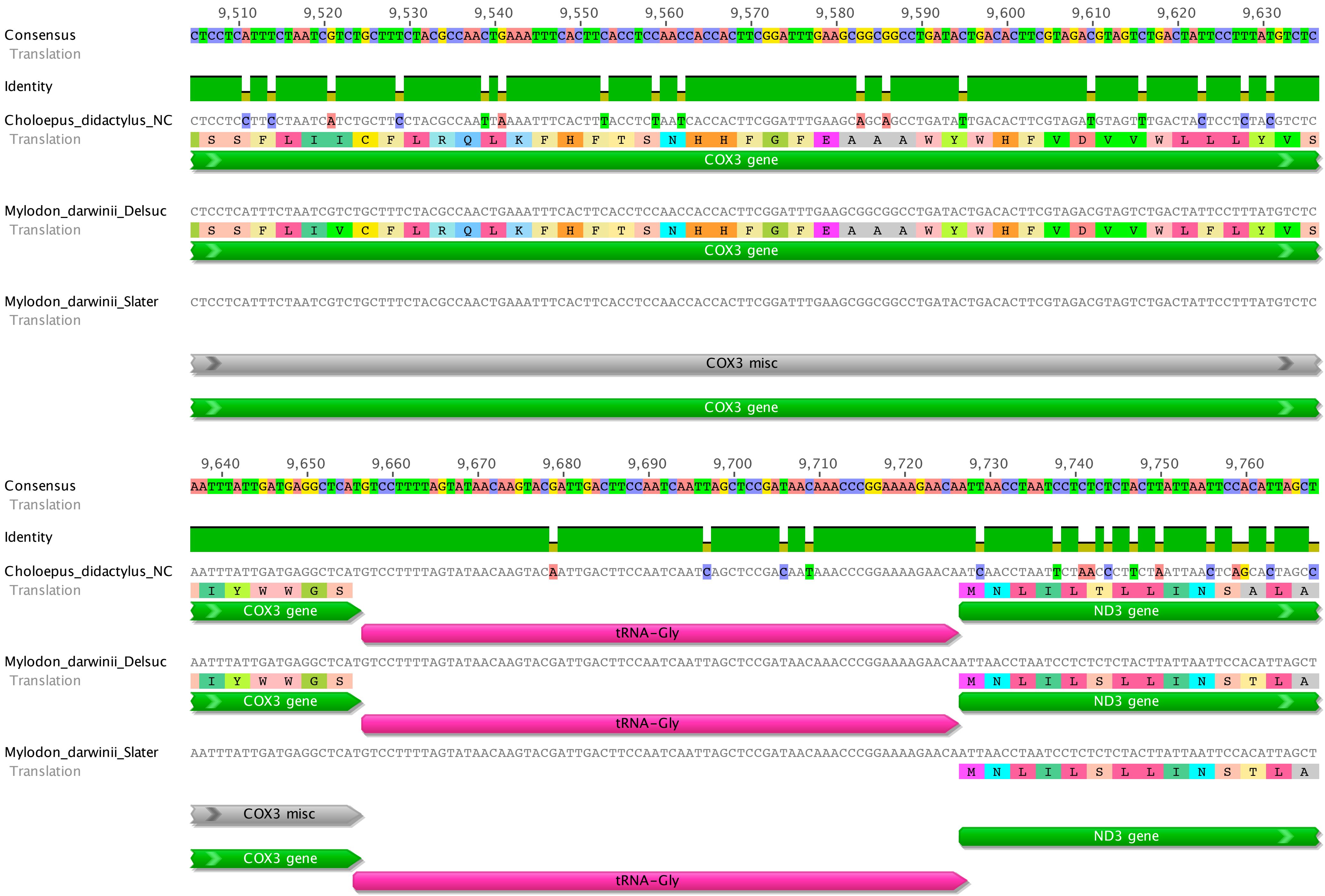

Consensus
Translation
Identity
Choloepus_didactylus_NC
Translation
COX3 gene
Mylodon_darwinii_Delsuc
Translation
COX3 gene
Mylodon_darwinii_Slater
Translation
COX3 misc
COX3 gene
Consensus
Translation
Identity
Choloepus_didactylus_NC
Translation
COX3 gene
tRNA-Gly
ND3 gene
Mylodon_darwinii_Delsuc
Translation
COX3 gene
tRNA-Gly
ND3 gene
Mylodon_darwinii_Slater
Translation
COX3 misc
ND3 gene
COX3 gene
tRNA-Gly

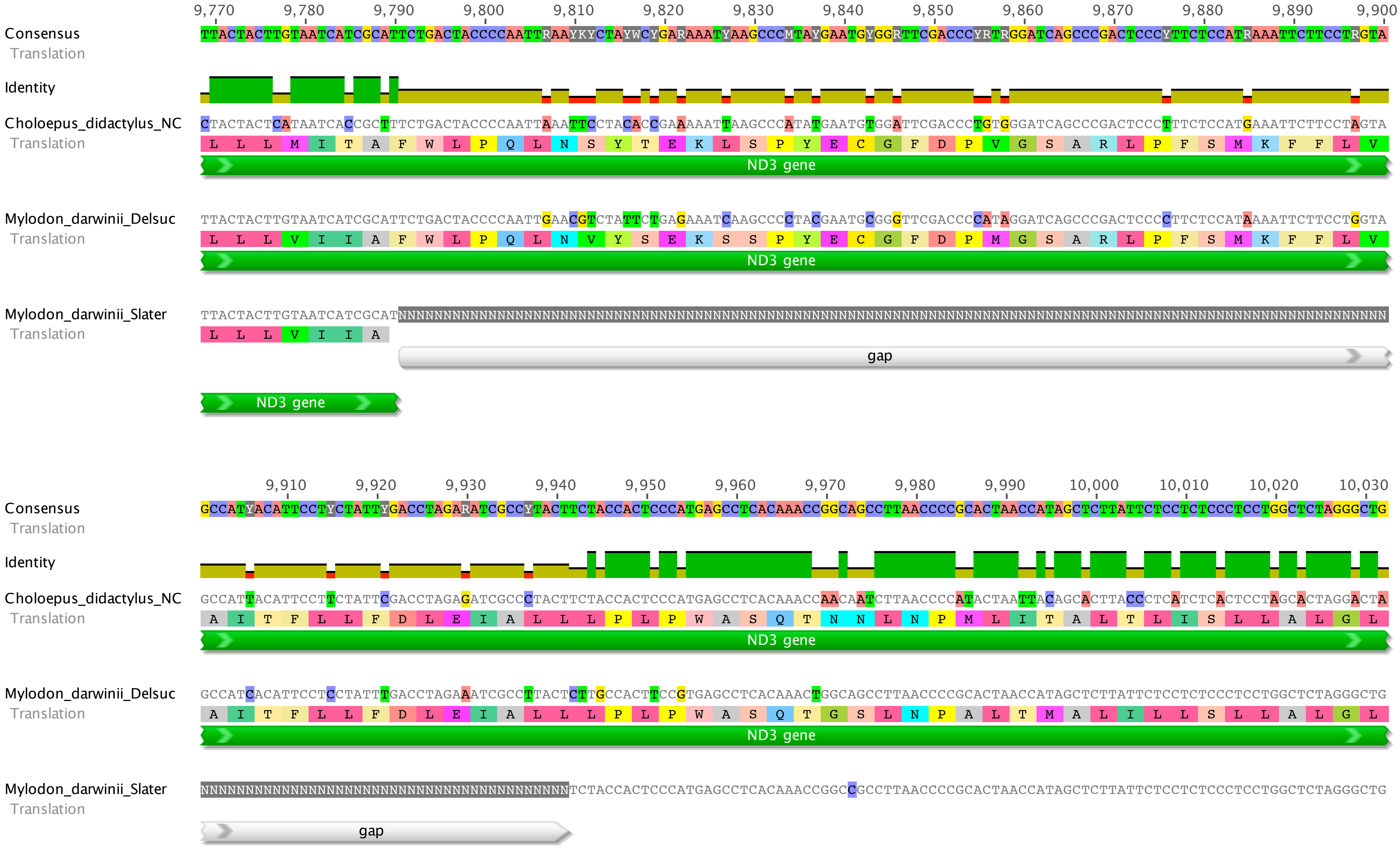
9,770
9,780
9,790
9,800
9,810
9,820
9,830
9,840
9,850
9,860
9,870
9,880
9,890
9,900
Consensus
Translation
Identity
Choloepus_didactylus_NC
Translation
ND3 gene
Mylodon_darwinii_Delsuc
Translation
ND3 gene
Mylodon_darwinii_Slater
Translation
gap
ND3 gene
9,910
9,920
9,930
9,940
9,950
9,960
9,970
9,980
9,990
10,000
10,010
10,020
10,030
Consensus
Translation
Identity
Choloepus_didactylus_NC
Translation
ND3 gene
Mylodon_darwinii_Delsuc
Translation
ND3 gene
Mylodon_darwinii_Slater
Translation
gap

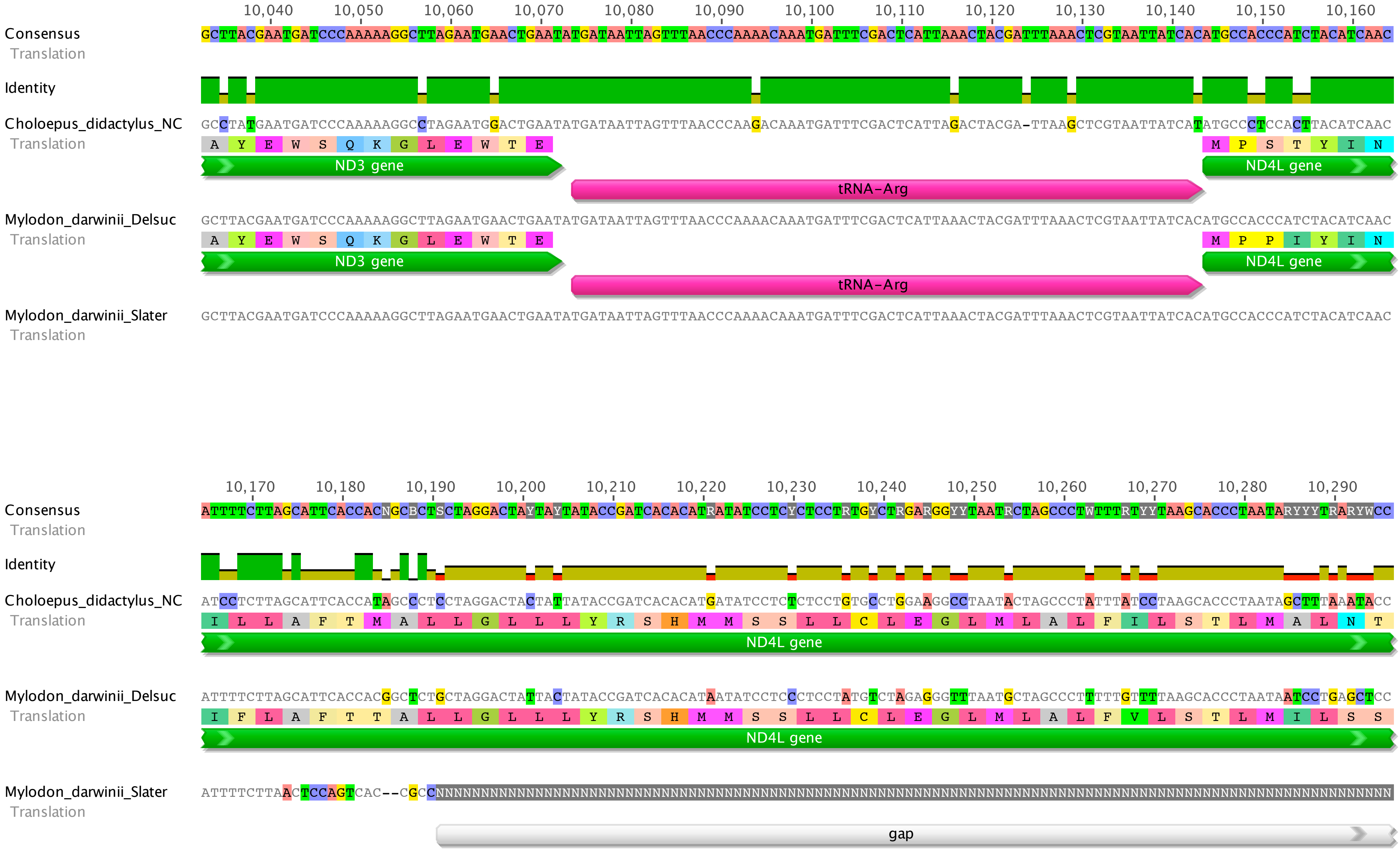
10,040
10,050
10,060
10,070
10,080
10,090
10,100
10,110
10,120
10,130
10,140
10,150
10,160
Consensus
Translation
Identity
Choloepus_didactylus_NC
Translation
ND3 gene
ND4L gene
tRNA-Arg
Mylodon_darwinii_Delsuc
Translation
ND3 gene
ND4L gene
tRNA-Arg
Mylodon_darwinii_Slater
Translation
10,170
10,180
10,190
10,200
10,210
10,220
10,230
10,240
10,250
10,260
10,270
10,280
10,290
Consensus
Translation
Identity
Choloepus_didactylus_NC
Translation
ND4L gene
Mylodon_darwinii_Delsuc
Translation
ND4L gene
Mylodon_darwinii_Slater
Translation
gap

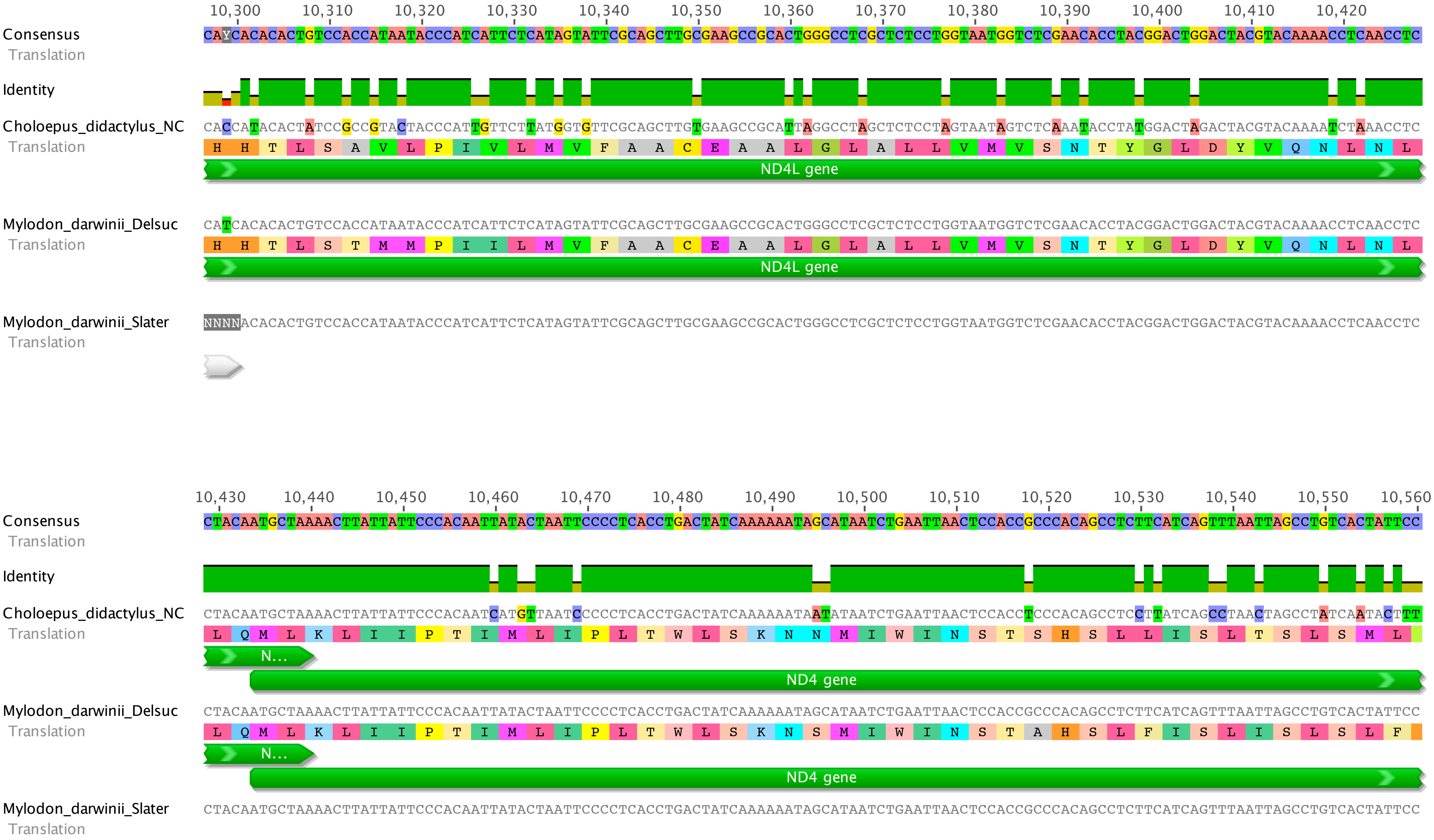
10,300 10,310 10,320 10,330 10,340 10,350 10,360 10,370 10,380 10,390 10,400 10,410 10,420
Consensus
Translation
Identity
Choloepus_didactylus_NC
Translation
ND4L gene
Mylodon_darwinii_Delsuc
Translation
ND4L gene
Mylodon_darwinii_Slater
Translation
10,430 10,440 10,450 10,460 10,470 10,480 10,490 10,500 10,510 10,520 10,530 10,540 10,550 10,560
Consensus
Translation
Identity
Choloepus_didactylus_NC
Translation
N...
ND4 gene
Mylodon_darwinii_Delsuc
Translation
N...
ND4 gene
Mylodon_darwinii_Slater
Translation

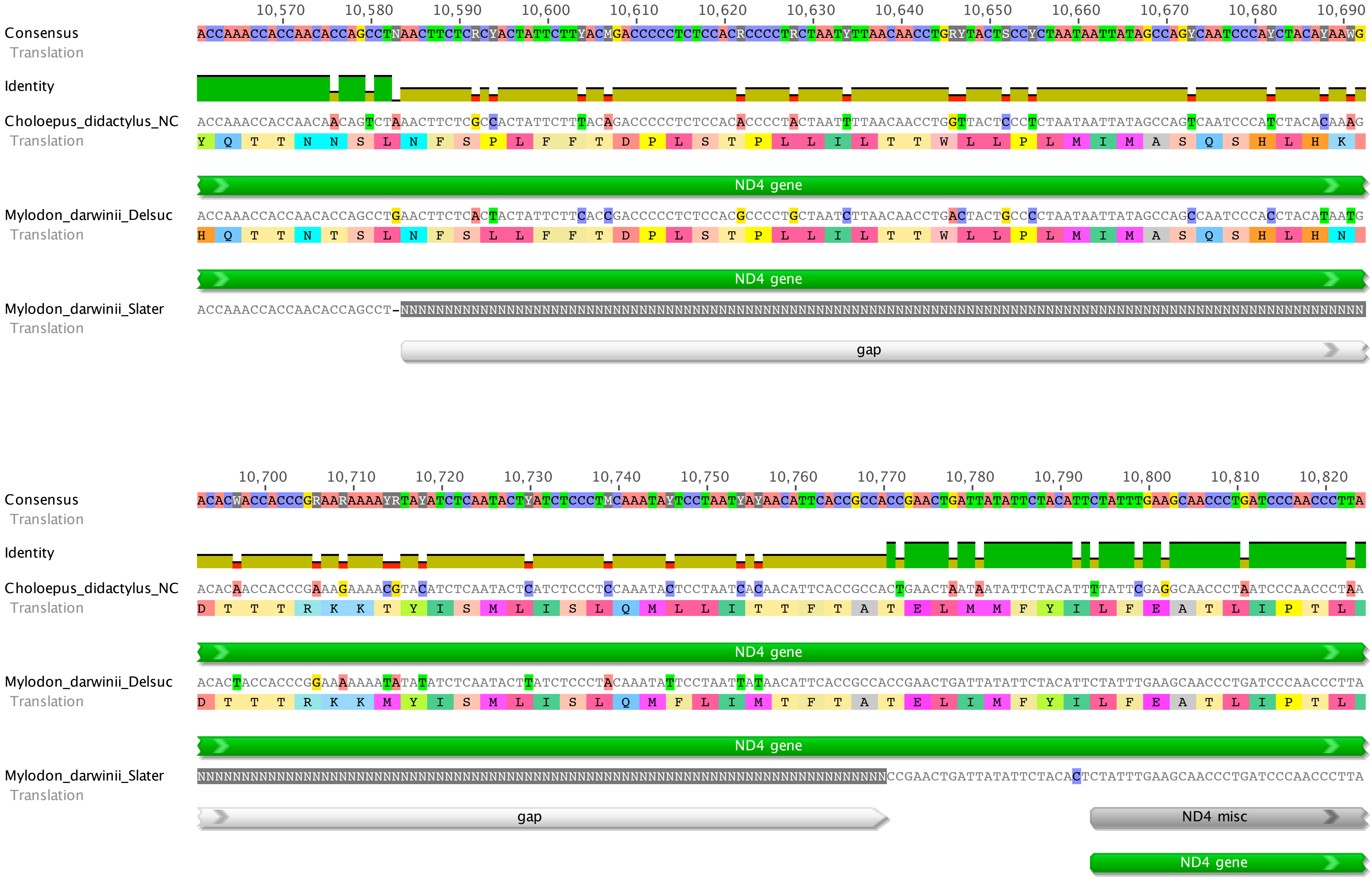

10,570
10,580
10,590
10,600
10,610
10,620
10,630
10,640
10,650
10,660
10,670
10,680
10,690
Consensus
Translation
Identity
Choloepus_didactylus_NC
Translation
ND4 gene
Mylodon_darwinii_Delsuc
Translation
ND4 gene
Mylodon_darwinii_Slater
Translation
gap
10,700
10,710
10,720
10,730
10,740
10,750
10,760
10,770
10,780
10,790
10,800
10,810
10,820
Consensus
Translation
Identity
Choloepus_didactylus_NC
Translation
ND4 gene
Mylodon_darwinii_Delsuc
Translation
ND4 gene
Mylodon_darwinii_Slater
Translation
gap
ND4 misc
ND4 gene

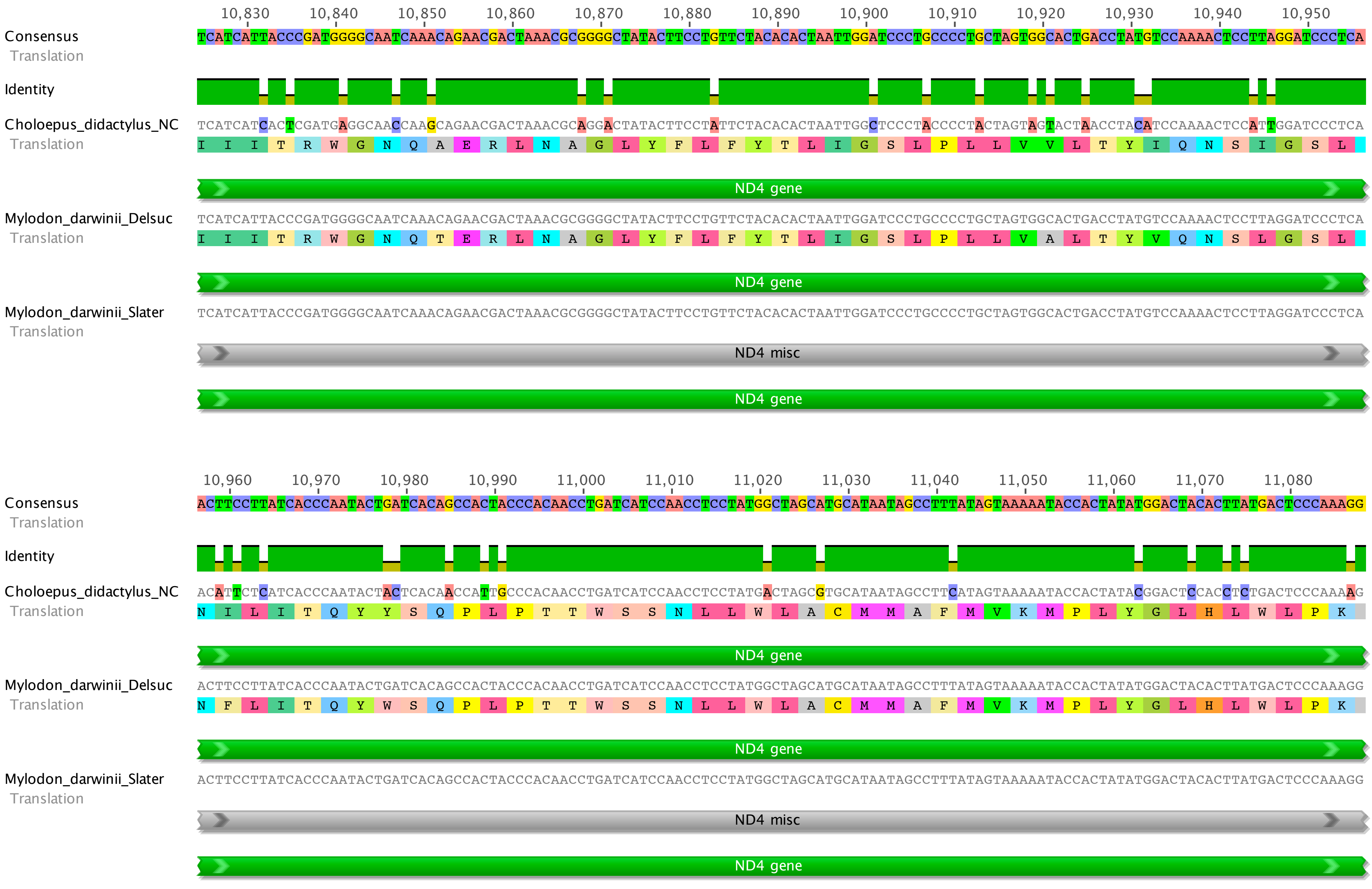
10,830 10,840 10,850 10,860 10,870 10,880 10,890 10,900 10,910 10,920 10,930 10,940 10,950
Consensus
Translation
Identity
Choloepus_didactylus_NC
Translation
ND4 gene
Mylodon_darwinii_Delsuc
Translation
ND4 gene
Mylodon_darwinii_Slater
Translation
ND4 misc
ND4 gene
10,960 10,970 10,980 10,990 11,000 11,010 11,020 11,030 11,040 11,050 11,060 11,070 11,080
Consensus
Translation
Identity
Choloepus_didactylus_NC
Translation
ND4 gene
Mylodon_darwinii_Delsuc
Translation
ND4 gene
Mylodon_darwinii_Slater
Translation
ND4 misc
ND4 gene

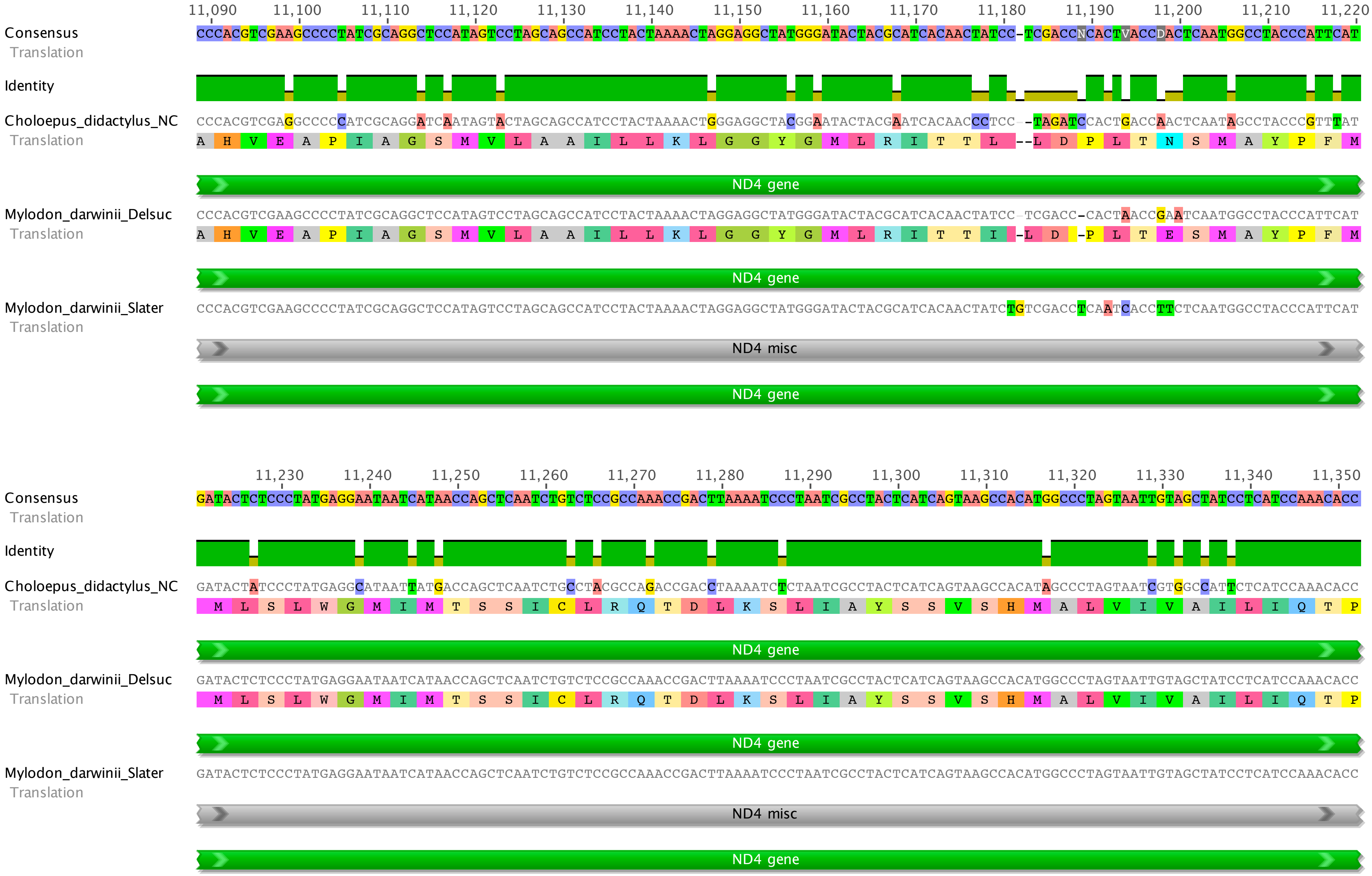
Consensus
Translation
Identity
Choloepus_didactylus_NC
Translation
ND4 gene
Mylodon_darwinii_Delsuc
Translation
ND4 gene
Mylodon_darwinii_Slater
Translation
ND4 misc
ND4 gene
Consensus
Translation
Identity
Choloepus_didactylus_NC
Translation
ND4 gene
Mylodon_darwinii_Delsuc
Translation
ND4 gene
Mylodon_darwinii_Slater
Translation
ND4 misc
ND4 gene

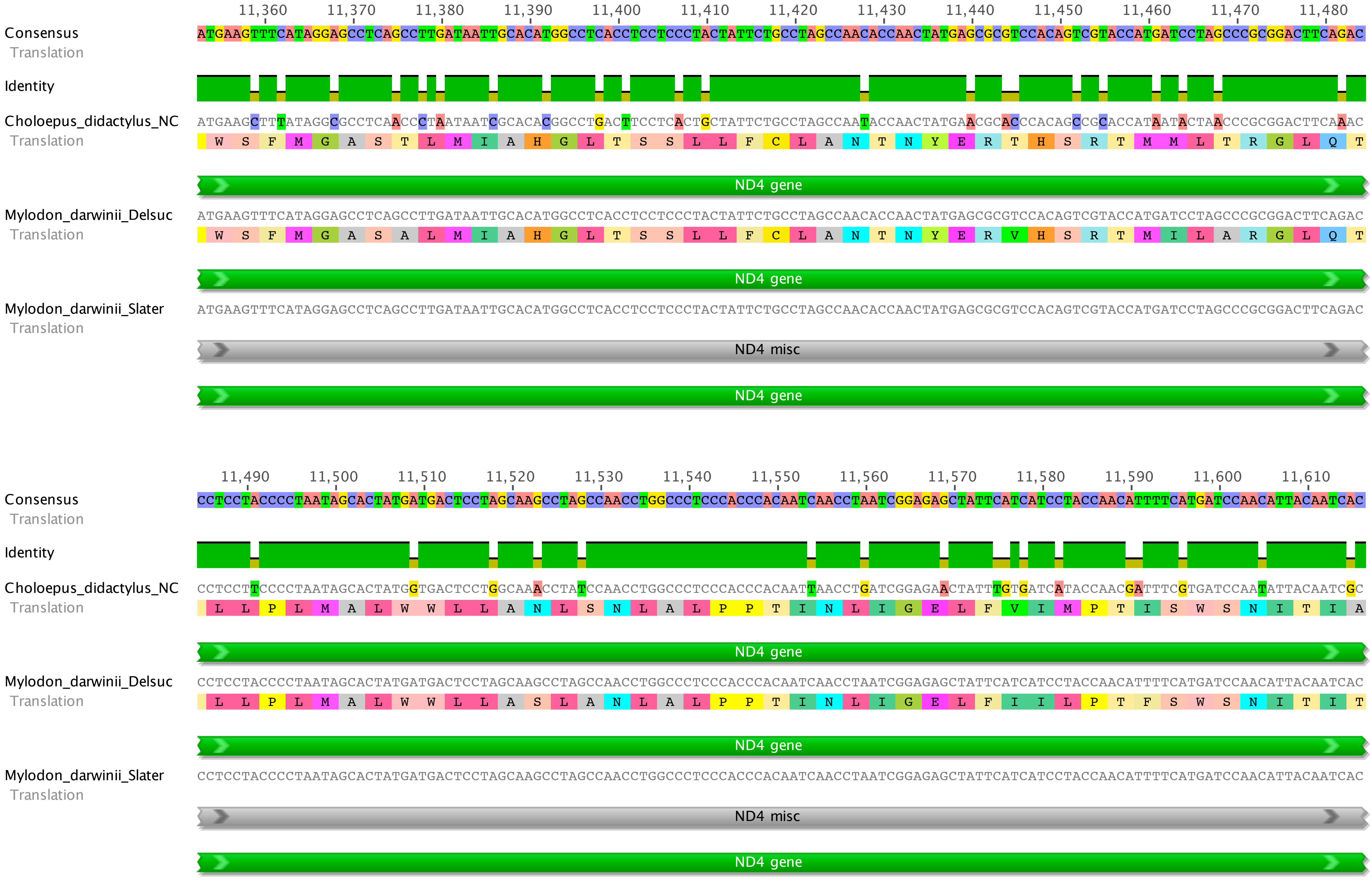

11,360 11,370 11,380 11,390 11,400 11,410 11,420 11,430 11,440 11,450 11,460 11,470 11,480
Consensus
ATGAAGTTTCATAGGAGCCTCAGCCTTGATAATTGCACATGGCCTCACCTCCTCCCTACTATTCTGCCTAGCCAACACCAACTATGAGCGCGTCCACAGTCGTACCATGATCCTAGCCCGCGGACTTCAGAC
Translation
Identity
Choloepus_didactylus_NC
ATGAAGCTTTTATAGGCGCCTCAACCCTAATAATCGCACACGGCCTGACTTCCTCACTGCTATTCTGCCTAGCCAATACCAACTATGAACGCACCCACAGCCGCACCATAATACTAACCCGCGGACTTCAAAC
Translation
W S F M G A S T L M I A H G L T S S L L F C L A N T N Y E R T H S R T M M L T R G L Q T
ND4 gene
Mylodon_darwinii_Delsuc
ATGAAGTTTCATAGGAGCCTCAGCCTTGATAATTGCACATGGCCTCACCTCCTCCCTACTATTCTGCCTAGCCAACACCAACTATGAGCGCGTCCACAGTCGTACCATGATCCTAGCCCGCGGACTTCAGAC
Translation
W S F M G A S A L M I A H G L T S S L L F C L A N T N Y E R V H S R T M I L A R G L Q T
ND4 gene
Mylodon_darwinii_Slater
ATGAAGTTTCATAGGAGCCTCAGCCTTGATAATTGCACATGGCCTCACCTCCTCCCTACTATTCTGCCTAGCCAACACCAACTATGAGCGCGTCCACAGTCGTACCATGATCCTAGCCCGCGGACTTCAGAC
Translation
ND4 misc
ND4 gene
11,490 11,500 11,510 11,520 11,530 11,540 11,550 11,560 11,570 11,580 11,590 11,600 11,610
Consensus
CCTCCTACCCCTAATAGCACTATGATGACTCCTAGCAAGCCTAGCCAACCTGGCCCTCCCACCCACAATCAACCTAATCGGAGAGCTATTCATCATCCTACCAACATTTTCATGATCCAACATTACAATCAC
Translation
Identity
Choloepus_didactylus_NC
CCTCCTTCCCCTAATAGCACTATGGTGACTCCTGGCAAACCTATCCAACCTGGCCCTCCCACCCACAATTAACCTGATCGGAGAACTATTTGTGATCATACCAACGATTTCGTGATCCAATATTACAATCGC
Translation
L L P L M A L W W L L A N L S N L A L P P T I N L I G E L F V I M P T I S W S N I T I A
ND4 gene
Mylodon_darwinii_Delsuc
CCTCCTACCCCTAATAGCACTATGATGACTCCTAGCAAGCCTAGCCAACCTGGCCCTCCCACCCACAATCAACCTAATCGGAGAGCTATTCATCATCCTACCAACATTTTCATGATCCAACATTACAATCAC
Translation
L L P L M A L W W L L A S L A N L A L P P T I N L I G E L F I I L P T F S W S N I T I T
ND4 gene
Mylodon_darwinii_Slater
CCTCCTACCCCTAATAGCACTATGATGACTCCTAGCAAGCCTAGCCAACCTGGCCCTCCCACCCACAATCAACCTAATCGGAGAGCTATTCATCATCCTACCAACATTTTCATGATCCAACATTACAATCAC
Translation
ND4 misc
ND4 gene

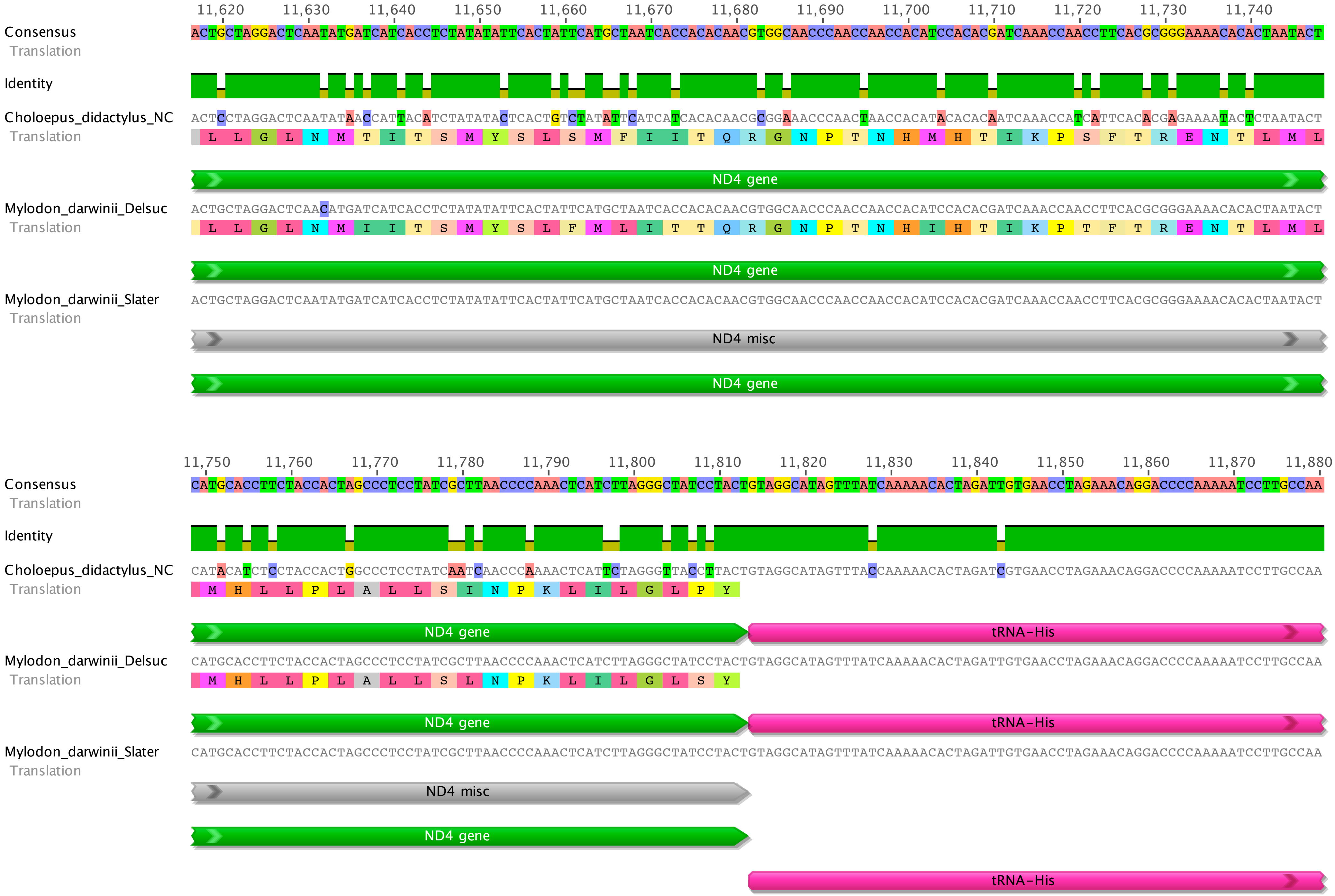

11,620 11,630 11,640 11,650 11,660 11,670 11,680 11,690 11,700 11,710 11,720 11,730 11,740
Consensus
ACTGCTAGGACTCAATATGATCATCACCTCTATATATTCACTATTCATGCTAATCACCACACAACGTGGCAACCCAACCAACCACATCCACACGATCAAACCAACCTTCACGCGGGAAAACACACTAATACT
Translation
Identity
Choloepus_didactylus_NC
ACTCCTAGGACTCAATATAACCATTACATCTATATACTCACTGTCTATATTCATCATCACACAACGCGGAAACCCAACTAACCACATACACACAATCAAACCATCATTCACACGAGAAAATACTCTAATACT
Translation
L L G L N M T I T S M Y S L S M F I I T Q R G N P T N H M H T I K P S F T R E N T L M L
ND4 gene
Mylodon_darwinii_Delsuc
ACTGCTAGGACTCAACATGATCATCACCTCTATATATTCACTATTCATGCTAATCACCACACAACGTGGCAACCCAACCAACCACATCCACACGATCAAACCAACCTTCACGCGGGAAAACACACTAATACT
Translation
L L G L N M I I T S M Y S L F M L I T T Q R G N P T N H I H T I K P T F T R E N T L M L
ND4 gene
Mylodon_darwinii_Slater
ACTGCTAGGACTCAATATGATCATCACCTCTATATATTCACTATTCATGCTAATCACCACACAACGTGGCAACCCAACCAACCACATCCACACGATCAAACCAACCTTCACGCGGGAAAACACACTAATACT
Translation
ND4 misc
ND4 gene
11,750 11,760 11,770 11,780 11,790 11,800 11,810 11,820 11,830 11,840 11,850 11,860 11,870 11,880
Consensus
CATGCACCTTCTACCACTAGCCCTCCTATCGCTTAACCCCAAACTCATCTTAGGGCTATCCTACTGTAGGCATAGTTTATCAAAAACACTAGATTGTGAACCTAGAAACAGGACCCCAAAAATCCTTGCCAA
Translation
Identity
Choloepus_didactylus_NC
CATACATCTCCTACCACTGGCCCTCCTATCAATCAACCCAAAACTCATTCTAGGGTTACCTTACTGTAGGCATAGTTTACCAAAAACACTAGATCGTGAACCTAGAAACAGGACCCCAAAAATCCTTGCCAA
Translation
M H L L P L A L L S I N P K L I L G L P Y
ND4 gene
tRNA-His
Mylodon_darwinii_Delsuc
CATGCACCTTCTACCACTAGCCCTCCTATCGCTTAACCCCAAACTCATCTTAGGGCTATCCTACTGTAGGCATAGTTTATCAAAAACACTAGATTGTGAACCTAGAAACAGGACCCCAAAAATCCTTGCCAA
Translation
M H L L P L A L L S L N P K L I L G L S Y
ND4 gene
tRNA-His
Mylodon_darwinii_Slater
CATGCACCTTCTACCACTAGCCCTCCTATCGCTTAACCCCAAACTCATCTTAGGGCTATCCTACTGTAGGCATAGTTTATCAAAAACACTAGATTGTGAACCTAGAAACAGGACCCCAAAAATCCTTGCCAA
Translation
ND4 misc
ND4 gene
tRNA-His

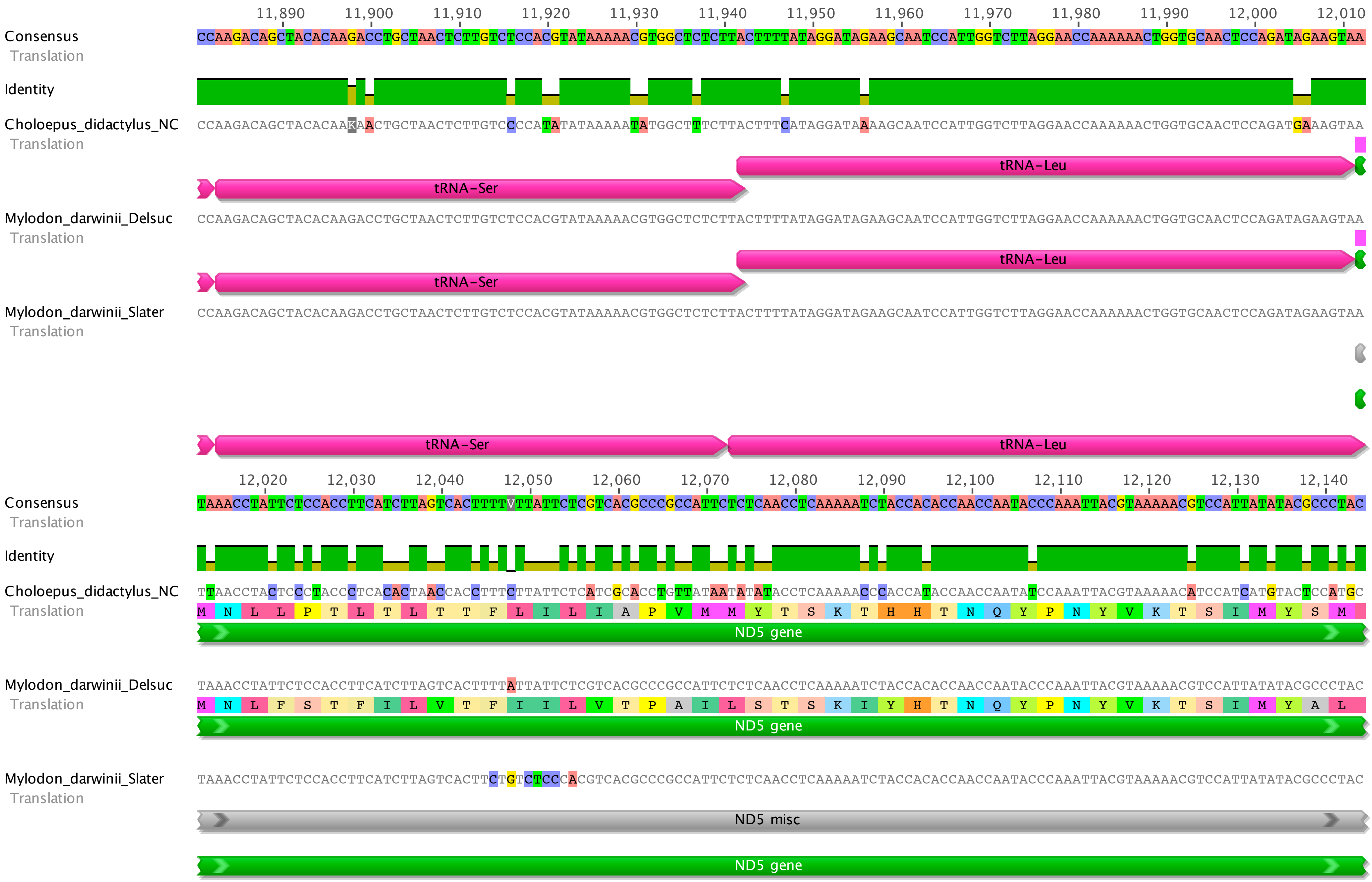
11,890
11,900
11,910
11,920
11,930
11,940
11,950
11,960
11,970
11,980
11,990
12,000
12,010
Consensus
Translation
Identity
Choloepus_didactylus_NC
Translation
tRNA-Leu
tRNA-Ser
Mylodon_darwinii_Delsuc
Translation
tRNA-Leu
tRNA-Ser
Mylodon_darwinii_Slater
Translation
tRNA-Ser
tRNA-Leu
12,020
12,030
12,040
12,050
12,060
12,070
12,080
12,090
12,100
12,110
12,120
12,130
12,140
Consensus
Translation
Identity
Choloepus_didactylus_NC
Translation
ND5 gene
Mylodon_darwinii_Delsuc
Translation
ND5 gene
Mylodon_darwinii_Slater
Translation
ND5 misc
ND5 gene

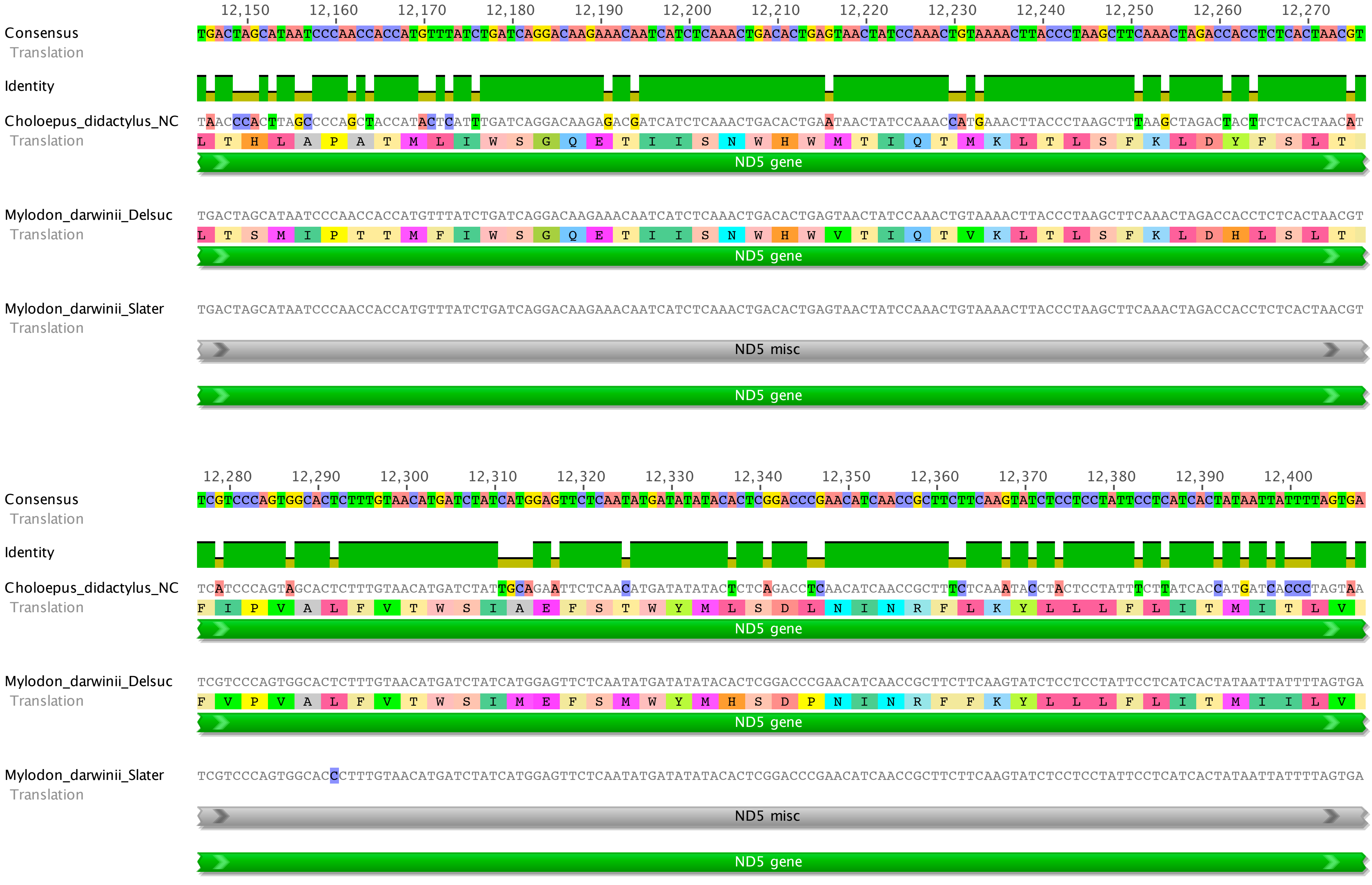
12,150 12,160 12,170 12,180 12,190 12,200 12,210 12,220 12,230 12,240 12,250 12,260 12,270
Consensus
TGACTAGCATAATCCCAACCACCATGTTTATCTGATCAGGACAAGAAACAATCATCTCAAACTGACACTGAGTAACTATCCAAACTGTAAAACTTACCCTAAGCTTCAAACTAGACCACCTCTCACTAACGT
Translation
Identity
Choloepus_didactylus_NC
TAACCCACTTAGCCCCAGCTACCATACTCATTTGATCAGGACAAGAGACGATCATCTCAAACTGACACTGAATAACTATCCAAACCATGAAACTTACCCTAAGCTTTAAGCTAGACTACTTCTCACTAACAT
Translation
L T H L A P A T M L I W S G Q E T I I S N W H W M T I Q T M K L T L S F K L D Y F S L T
ND5 gene
Mylodon_darwinii_Delsuc
TGACTAGCATAATCCCAACCACCATGTTTATCTGATCAGGACAAGAAACAATCATCTCAAACTGACACTGAGTAACTATCCAAACTGTAAAACTTACCCTAAGCTTCAAACTAGACCACCTCTCACTAACGT
Translation
L T S M I P T T M F I W S G Q E T I I S N W H W V T I Q T V K L T L S F K L D H L S L T
ND5 gene
Mylodon_darwinii_Slater
TGACTAGCATAATCCCAACCACCATGTTTATCTGATCAGGACAAGAAACAATCATCTCAAACTGACACTGAGTAACTATCCAAACTGTAAAACTTACCCTAAGCTTCAAACTAGACCACCTCTCACTAACGT
Translation
ND5 misc
ND5 gene
12,280 12,290 12,300 12,310 12,320 12,330 12,340 12,350 12,360 12,370 12,380 12,390 12,400
Consensus
TCGTCCCAGTGGCACTCTTTGTAACATGATCTATCATGGAGTTCTCAATATGATATATACACTCGGACCCGAACATCAACCGCTTCTTCAAGTATCTCCTCCTATTCCTCATCACTATAATTATTTTAGTGA
Translation
Identity
Choloepus_didactylus_NC
TCATCCCAGTAGCACTCTTTGTAACATGATCTATTGCAGAATTCTCAACATGATATATACTCTCAGACCTCAACATCAACCGCTTTCTCAAATACCTACTCCTATTTCTTATCACCATGATCACCCTAGTAA
Translation
F I P V A L F V T W S I A E F S T W Y M L S D L N I N R F L K Y L L L F L I T M I T L V
ND5 gene
Mylodon_darwinii_Delsuc
TCGTCCCAGTGGCACTCTTTGTAACATGATCTATCATGGAGTTCTCAATATGATATATACACTCGGACCCGAACATCAACCGCTTCTTCAAGTATCTCCTCCTATTCCTCATCACTATAATTATTTTAGTGA
Translation
F V P V A L F V T W S I M E F S M W Y M H S D P N I N R F F K Y L L L F L I T M I I L V
ND5 gene
Mylodon_darwinii_Slater
TCGTCCCAGTGGCACCCTTTGTAACATGATCTATCATGGAGTTCTCAATATGATATATACACTCGGACCCGAACATCAACCGCTTCTTCAAGTATCTCCTCCTATTCCTCATCACTATAATTATTTTAGTGA
Translation
ND5 misc
ND5 gene

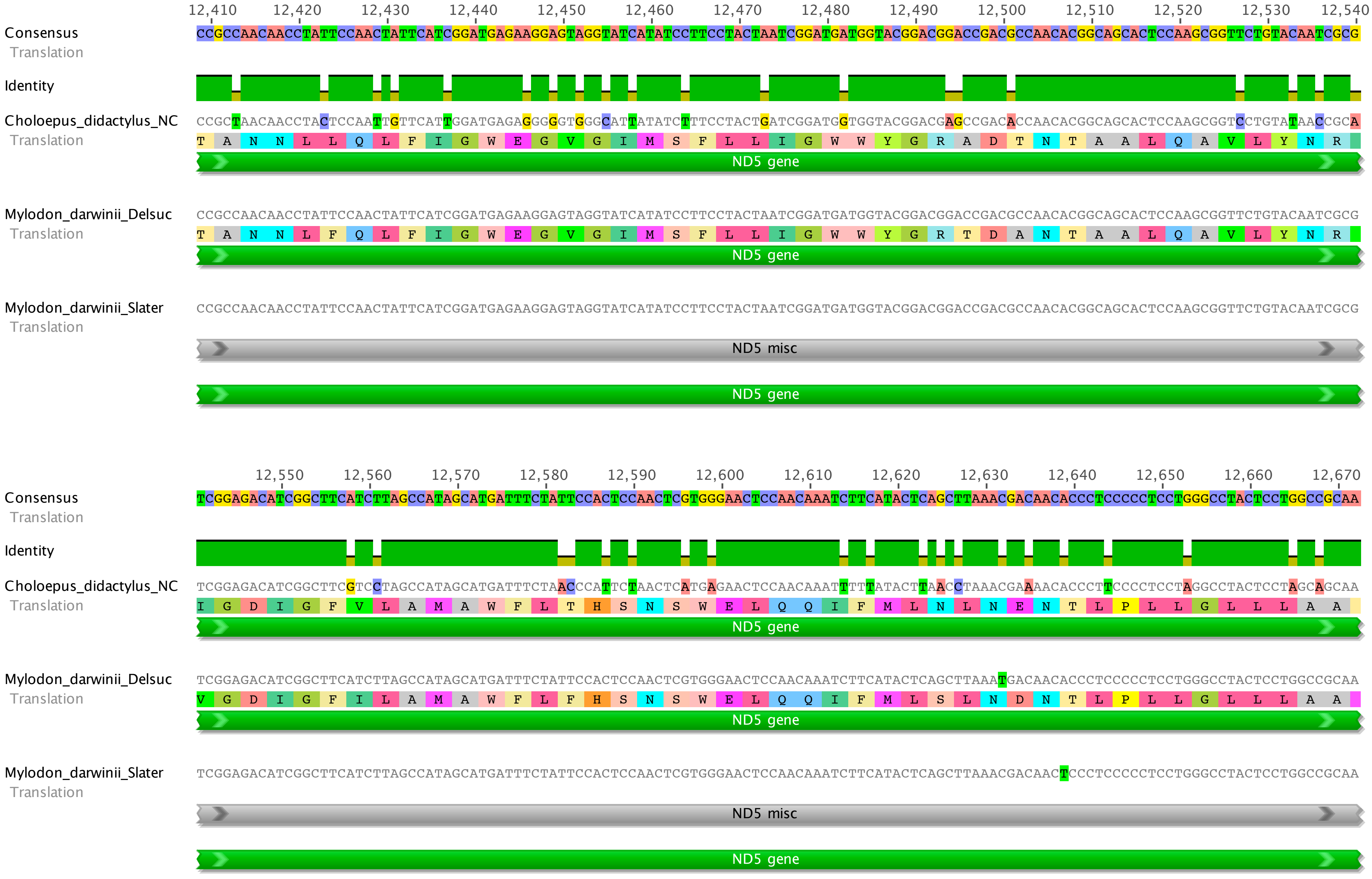
12,410 12,420 12,430 12,440 12,450 12,460 12,470 12,480 12,490 12,500 12,510 12,520 12,530 12,540
Consensus
CCGCCAACAACCTATTCCAACTATTCATCGGATGAGAAGGAGTAGGTATCATATCCTTCCTACTAATCGGATGATGGTACGGACGGACCGACGCCAACACGGCAGCACTCCAAGCGGTTCTGTACAATCGCG
Translation
Identity
Choloepus_didactylus_NC
CCGCTAACAACCTACTCCAATTGTTCATTGGATGAGAGGGGGTGGGCATTATATCTTTCCTACTGATCGGATGGTGGTACGGACGAGCCGACACCAACACGGCAGCACTCCAAGCGGTCCTGTATAACCGCA
Translation
T A N N L L Q L F I G W E G V G I M S F L L I G W W Y G R A D T N T A A L Q A V L Y N R
ND5 gene
Mylodon_darwinii_Delsuc
CCGCCAACAACCTATTCCAACTATTCATCGGATGAGAAGGAGTAGGTATCATATCCTTCCTACTAATCGGATGATGGTACGGACGGACCGACGCCAACACGGCAGCACTCCAAGCGGTTCTGTACAATCGCG
Translation
T A N N L F Q L F I G W E G V G I M S F L L I G W W Y G R T D A N T A A L Q A V L Y N R
ND5 gene
Mylodon_darwinii_Slater
CCGCCAACAACCTATTCCAACTATTCATCGGATGAGAAGGAGTAGGTATCATATCCTTCCTACTAATCGGATGATGGTACGGACGGACCGACGCCAACACGGCAGCACTCCAAGCGGTTCTGTACAATCGCG
Translation
ND5 misc
ND5 gene
12,550 12,560 12,570 12,580 12,590 12,600 12,610 12,620 12,630 12,640 12,650 12,660 12,670
Consensus
TCGGAGACATCGGCTTCATCTTAGCCATAGCATGATTTCTATTCCACTCCAACTCGTGGGAACTCCAACAAATCTTCATACTCAGCTTAAACGACAACACCCTCCCCCTCCTGGGCCTACTCCTGGCCGCAA
Translation
Identity
Choloepus_didactylus_NC
TCGGAGACATCGGCTTCGTCCTAGCCATAGCATGATTTCTAACCCATTCTAACTCATGAGAACTCCAACAAATTTTTATACTTAACCTAAACGAAAACACCCTTCCCCTCCTAGGCCTACTCCTAGCAGCAA
Translation
I G D I G F V L A M A W F L T H S N S W E L Q Q I F M L N L N E N T L P L L G L L L A A
ND5 gene
Mylodon_darwinii_Delsuc
TCGGAGACATCGGCTTCATCTTAGCCATAGCATGATTTCTATTCCACTCCAACTCGTGGGAACTCCAACAAATCTTCATACTCAGCTTAAATGACAACACCCTCCCCCTCCTGGGCCTACTCCTGGCCGCAA
Translation
V G D I G F I L A M A W F L F H S N S W E L Q Q I F M L S L N D N T L P L L G L L L A A
ND5 gene
Mylodon_darwinii_Slater
TCGGAGACATCGGCTTCATCTTAGCCATAGCATGATTTCTATTCCACTCCAACTCGTGGGAACTCCAACAAATCTTCATACTCAGCTTAAACGACAACTCCCTCCCCCTCCTGGGCCTACTCCTGGCCGCAA
Translation
ND5 misc
ND5 gene

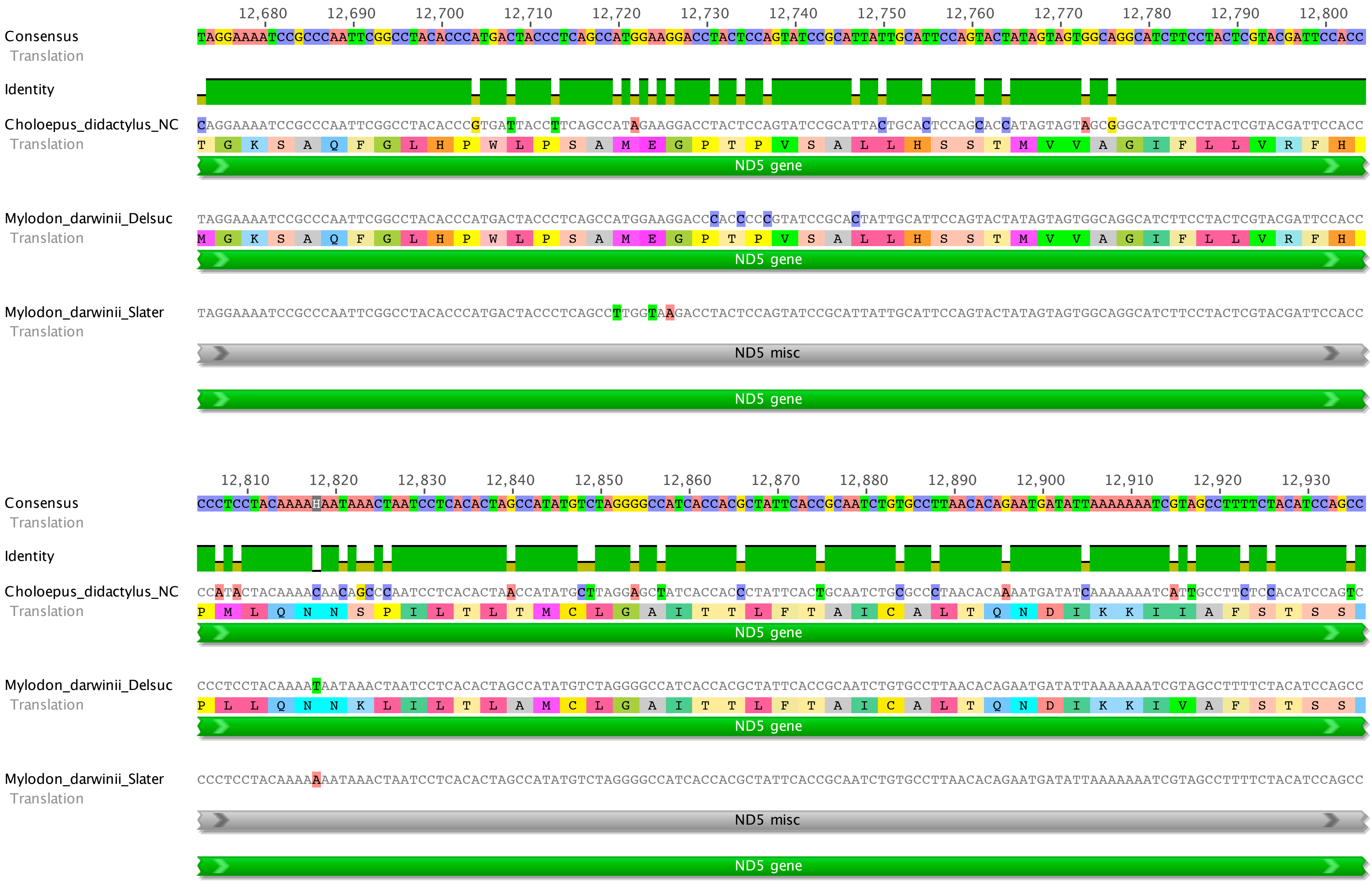

12,680 12,690 12,700 12,710 12,720 12,730 12,740 12,750 12,760 12,770 12,780 12,790 12,800
Consensus
Translation
Identity
Choloepus_didactylus_NC
Translation
ND5 gene
Mylodon_darwinii_Delsuc
Translation
ND5 gene
Mylodon_darwinii_Slater
Translation
ND5 misc
ND5 gene
12,810 12,820 12,830 12,840 12,850 12,860 12,870 12,880 12,890 12,900 12,910 12,920 12,930
Consensus
Translation
Identity
Choloepus_didactylus_NC
Translation
ND5 gene
Mylodon_darwinii_Delsuc
Translation
ND5 gene
Mylodon_darwinii_Slater
Translation
ND5 misc
ND5 gene

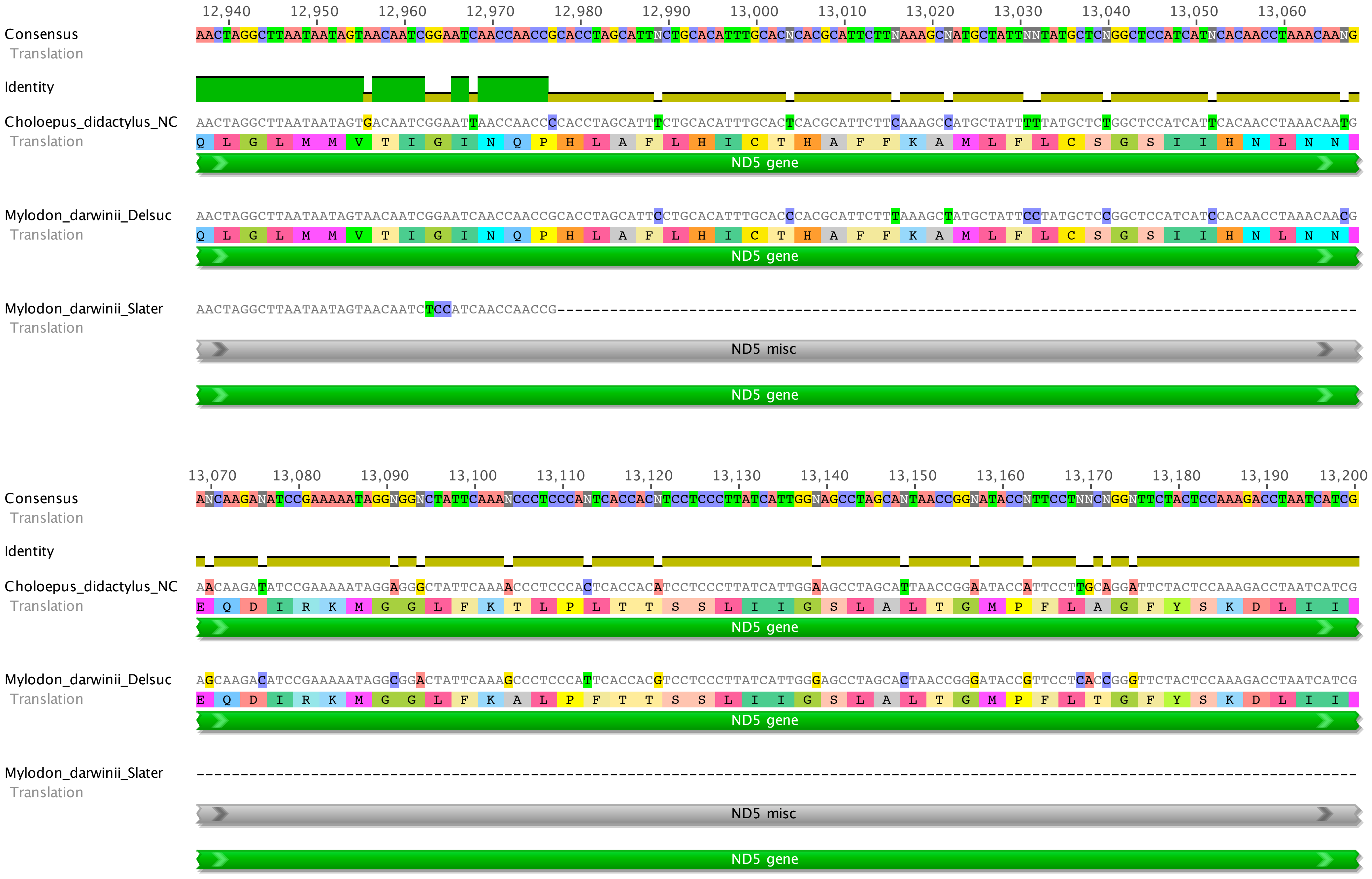
12,940 12,950 12,960 12,970 12,980 12,990 13,000 13,010 13,020 13,030 13,040 13,050 13,060
Consensus
AACTAGGCTTAATAATAGTAACAATCGGAATCAACCAACCGCACCTAGCATTNCTGCACATTTGCACNCACGCATTCTTNAAAGCNATGCTATTNNTATGCTCNGGCTCCATCATNCACAACCTAAACAANG
Translation
Identity
Choloepus_didactylus_NC
AACTAGGCTTAATAATAGTGACAATCGGAATTAACCAACCCCACCTAGCATTTCTGCACATTTGCACTCACGCATTCTTCAAAGCCATGCTATTTTTATGCTCTGGCTCCATCATTCACAACCTAAACAATG
Translation
Q L G L M M V T I G I N Q P H L A F L H I C T H A F F K A M L F L C S G S I I H N L N N
ND5 gene
Mylodon_darwinii_Delsuc
AACTAGGCTTAATAATAGTAACAATCGGAATCAACCAACCGCACCTAGCATTCCTGCACATTTGCACCCACGCATTCTTTAAAGCTATGCTATTCCTATGCTCCGGCTCCATCATCCACAACCTAAACAACG
Translation
Q L G L M M V T I G I N Q P H L A F L H I C T H A F F K A M L F L C S G S I I H N L N N
ND5 gene
Mylodon_darwinii_Slater
AACTAGGCTTAATAATAGTAACAATCTCCATCAACCAACCG---
Translation
ND5 misc
ND5 gene
13,070 13,080 13,090 13,100 13,110 13,120 13,130 13,140 13,150 13,160 13,170 13,180 13,190 13,200
Consensus
ANCAAGANATCCGAAAAATAGGNGGNCTATTCAAANCCCTCCCANTCACCACNTCCTCCCTTATCATTGGNAGCCTAGCANTAACCGGNATACCNTTCCTNNCNGGNTTCTACTCCAAAGACCTAATCATCG
Translation
Identity
Choloepus_didactylus_NC
AACAAGATATCCGAAAAATAGGAGGGCTATTCAAAACCCTCCCACTCACCACATCCTCCCTTATCATTGGAAGCCTAGCATTAACCGGAATACCATTCCTTGCAGGATTCTACTCCAAAGACCTAATCATCG
Translation
E Q D I R K M G G L F K T L P L T T S S L I I G S L A L T G M P F L A G F Y S K D L I I
ND5 gene
Mylodon_darwinii_Delsuc
AGCAAGACATCCGAAAAATAGGCGGACTATTCAAAGCCCTCCCATTCACCACGTCCTCCCTTATCATTGGGAGCCTAGCACTAACCGGGATACCGTTCCTCACCGGGTTCTACTCCAAAGACCTAATCATCG
Translation
E Q D I R K M G G L F K A L P F T T S S L I I G S L A L T G M P F L T G F Y S K D L I I
ND5 gene
Mylodon_darwinii_Slater

Translation
ND5 misc
ND5 gene

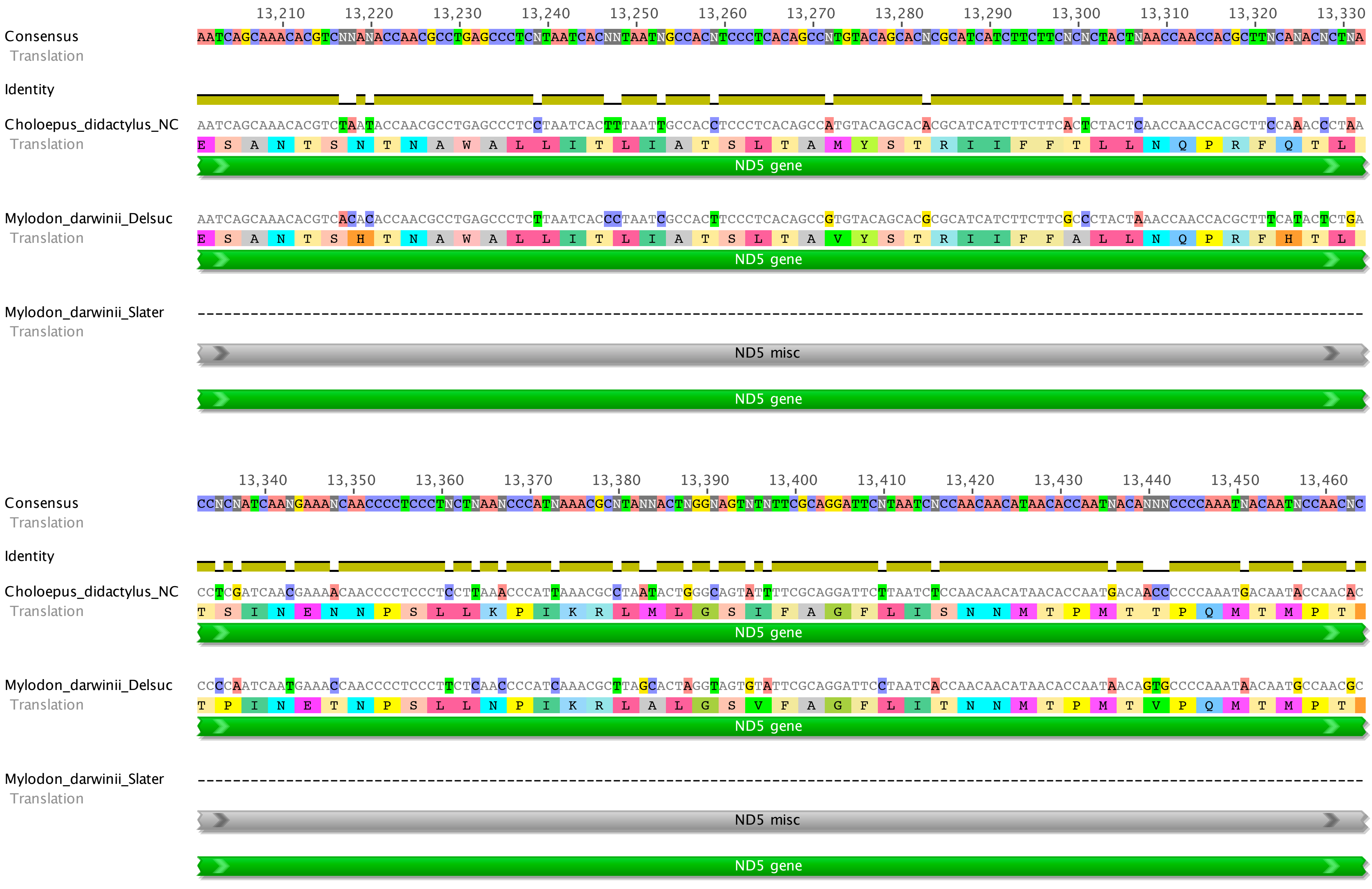
13,210 13,220 13,230 13,240 13,250 13,260 13,270 13,280 13,290 13,300 13,310 13,320 13,330
Consensus
Translation
Identity
Choloepus_didactylus_NC
Translation
ND5 gene
Mylodon_darwinii_Delsuc
Translation
ND5 gene
Mylodon_darwinii_Slater
Translation
ND5 misc
ND5 gene
13,340 13,350 13,360 13,370 13,380 13,390 13,400 13,410 13,420 13,430 13,440 13,450 13,460
Consensus
Translation
Identity
Choloepus_didactylus_NC
Translation
ND5 gene
Mylodon_darwinii_Delsuc
Translation
ND5 gene
Mylodon_darwinii_Slater
Translation
ND5 misc
ND5 gene

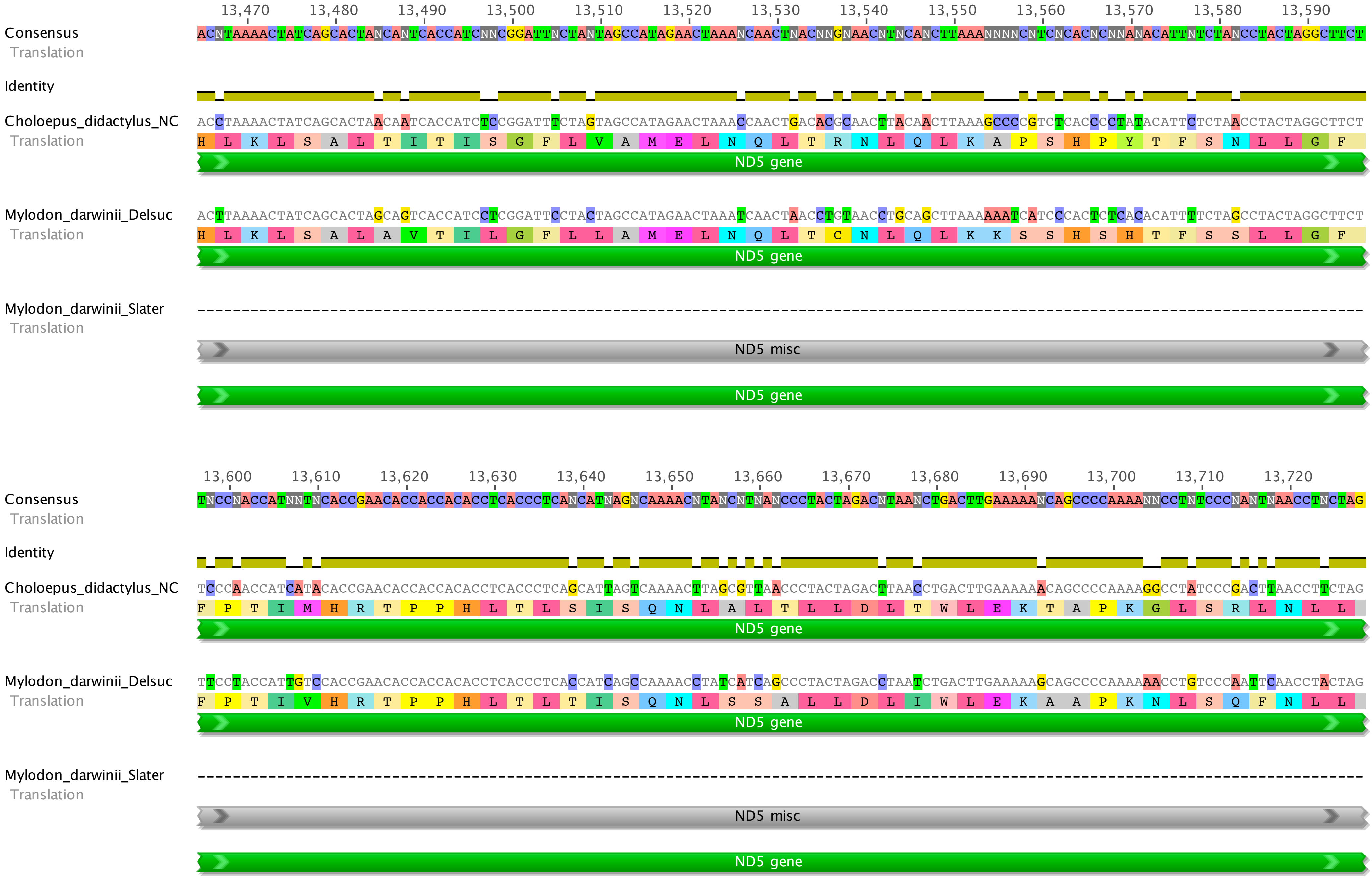
13,470 13,480 13,490 13,500 13,510 13,520 13,530 13,540 13,550 13,560 13,570 13,580 13,590
Consensus ACNTAAAACTATCAGCACTANCANTCACCATCNNCGGATTNCTANTAGCCATAGAACTAAANCAACTNACNNGNAACNTNCANCTTAAANNNNCNTCNCACNCNNANACATTNTCTANCCTACTAGGCTTCT
Translation
Identity
Choloepus_didactylus_NC ACCTAAAACTATCAGCACTAACAATCACCATCTCCGGATTTCTAGTAGCCATAGAACTAAACCAACTGACACGCAACTTACAACTTAAAGCCCCGTCTCACCCCTATACATTCTCTAACCTACTAGGCTTCT
Translation H L K L S A L T I T I S G F L V A M E L N Q L T R N L Q L K A P S H P Y T F S N L L G F
ND5 gene
Mylodon_darwinii_Delsuc ACTTAAAACTATCAGCACTAGCAGTCACCATCCTCGGATTCCTACTAGCCATAGAACTAAATCAACTAACCTGTAACCTGCAGCTTAAAAAATCATCCCACTCTCACACATTTTCTAGCCTACTAGGCTTCT
Translation H L K L S A L A V T I L G F L L A M E L N Q L T C N L Q L K K S S H S H T F S S L L G F
ND5 gene
Mylodon_darwinii_Slater
Translation
ND5 misc
ND5 gene
13,600 13,610 13,620 13,630 13,640 13,650 13,660 13,670 13,680 13,690 13,700 13,710 13,720
Consensus TNCCNACCATNNTNCACCGAACACCACCACACCTCACCCTCANCATNAGNCAAAACNTANCNTNANCCCTACTAGACNTAANCTGACTTGAAAAANCAGCCCCAAAANNCCTNTCCCNANTNAACCTNCTAG
Translation
Identity
Choloepus_didactylus_NC TCCCAACCATCATACACCGAACACCACCACACCTCACCCTCAGCATTAGTCAAAACTTAGCGTTAACCCTACTAGACTTAACCTGACTTGAAAAAACAGCCCCAAAAGGCCTATCCCGACTTAACCTTCTAG
Translation F P T I M H R T P P H L T L S I S Q N L A L T L L D L T W L E K T A P K G L S R L N L L
ND5 gene
Mylodon_darwinii_Delsuc TTCCTACCATTGTCCACCGAACACCACCACACCTCACCCTCACCATCAGCCAAAACCTATCATCAGCCCTACTAGACCTAATCTGACTTGAAAAAGCAGCCCCAAAAAACCTGTCCCAATTCAACCTACTAG
Translation F P T I V H R T P P H L T L T I S Q N L S S A L L D L I W L E K A A P K N L S Q F N L L
ND5 gene
Mylodon_darwinii_Slater
Translation
ND5 misc
ND5 gene

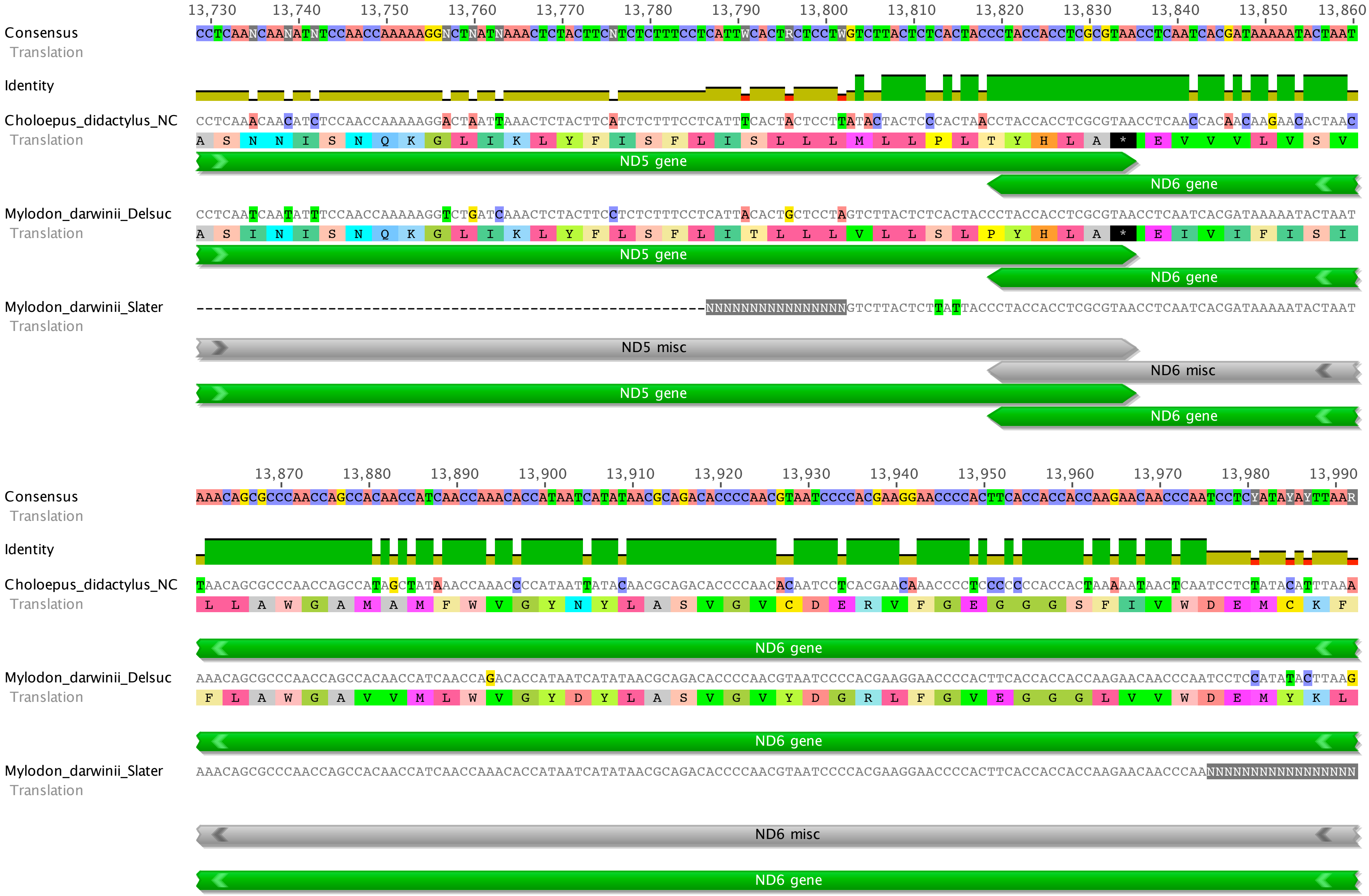
13,730 13,740 13,750 13,760 13,770 13,780 13,790 13,800 13,810 13,820 13,830 13,840 13,850 13,860
Consensus
CCTCAANCAANATNTCCAACCAAAAAGGNCTNATNAAACTCTACTTCNTCTCTTTCCTCATTWCACTRCTCCTWGTCTTACTCTCACTACCCTACCACCTCGCGTAACCTCAATCACGATAAAAATACTAAT
Translation
Identity
Choloepus_didactylus_NC
CCTCAAACAACATCTCCAACCAAAAAGGACTAATTAAACTCTACTTCATCTCTTTCCTCATTTCACTACTCCTTATACTACTCCCACTAACCTACCACCTCGCGTAACCTCAACCACAACAAGAACACTAAC
Translation
A S N N I S N Q K G L I K L Y F I S F L I S L L L M L L P L T Y H L A * E V V V L V S V
ND5 gene
ND6 gene
Mylodon_darwinii_Delsuc
CCTCAATCAATATTTCCAACCAAAAAGGTCTGATCAAACTCTACTTCCTCTCTTTCCTCATTACACTGCTCCTAGTCTTACTCTCACTACCCTACCACCTCGCGTAACCTCAATCACGATAAAAATACTAAT
Translation
A S I N I S N Q K G L I K L Y F L S F L I T L L L V L L S L P Y H L A * E I V I F I S I
ND5 gene
ND6 gene
Mylodon_darwinii_Slater
--NNNNNNNNNNNNNNNNGTCTTACTCTTATTACCCTACCACCTCGCGTAACCTCAATCACGATAAAAATACTAAT
Translation
ND5 misc
ND6 misc
ND5 gene
ND6 gene
13,870 13,880 13,890 13,900 13,910 13,920 13,930 13,940 13,950 13,960 13,970 13,980 13,990
Consensus
AAACAGCGCCCAACCAGCCACAACCATCAACCAAACACCATAATCATATAACGCAGACACCCCAACGTAATCCCCACGAAGGAACCCCACTTCACCACCACCAAGAACAACCCAATCCTCYATAYAYTTAAR
Translation
Identity
Choloepus_didactylus_NC
TAACAGCGCCCAACCAGCCATAGCTATAAACCAAACCCCATAATTATACAACGCAGACACCCCAACACAATCCTCACGAACAAACCCCTCCCCCCACCACTAAAAATAACTCAATCCTCTATACATTTAAA
Translation
L L A W G A M A M F W V G Y N Y L A S V G V C D E R V F G E G G G S F I V W D E M C K F
ND6 gene
Mylodon_darwinii_Delsuc
AAACAGCGCCCAACCAGCCACAACCATCAACCAGACACCATAATCATATAACGCAGACACCCCAACGTAATCCCCACGAAGGAACCCCACTTCACCACCACCAAGAACAACCCAATCCTCCATATACTTAAG
Translation
F L A W G A V V M L W V G Y D Y L A S V G V Y D G R L F G V E G G G L V V W D E M Y K L
ND6 gene
Mylodon_darwinii_Slater
AAACAGCGCCCAACCAGCCACAACCATCAACCAAACACCATAATCATATAACGCAGACACCCCAACGTAATCCCCACGAAGGAACCCCACTTCACCACCACCAAGAACAACCCAANNNNNNNNNNNNNNNNN
Translation
ND6 misc
ND6 gene

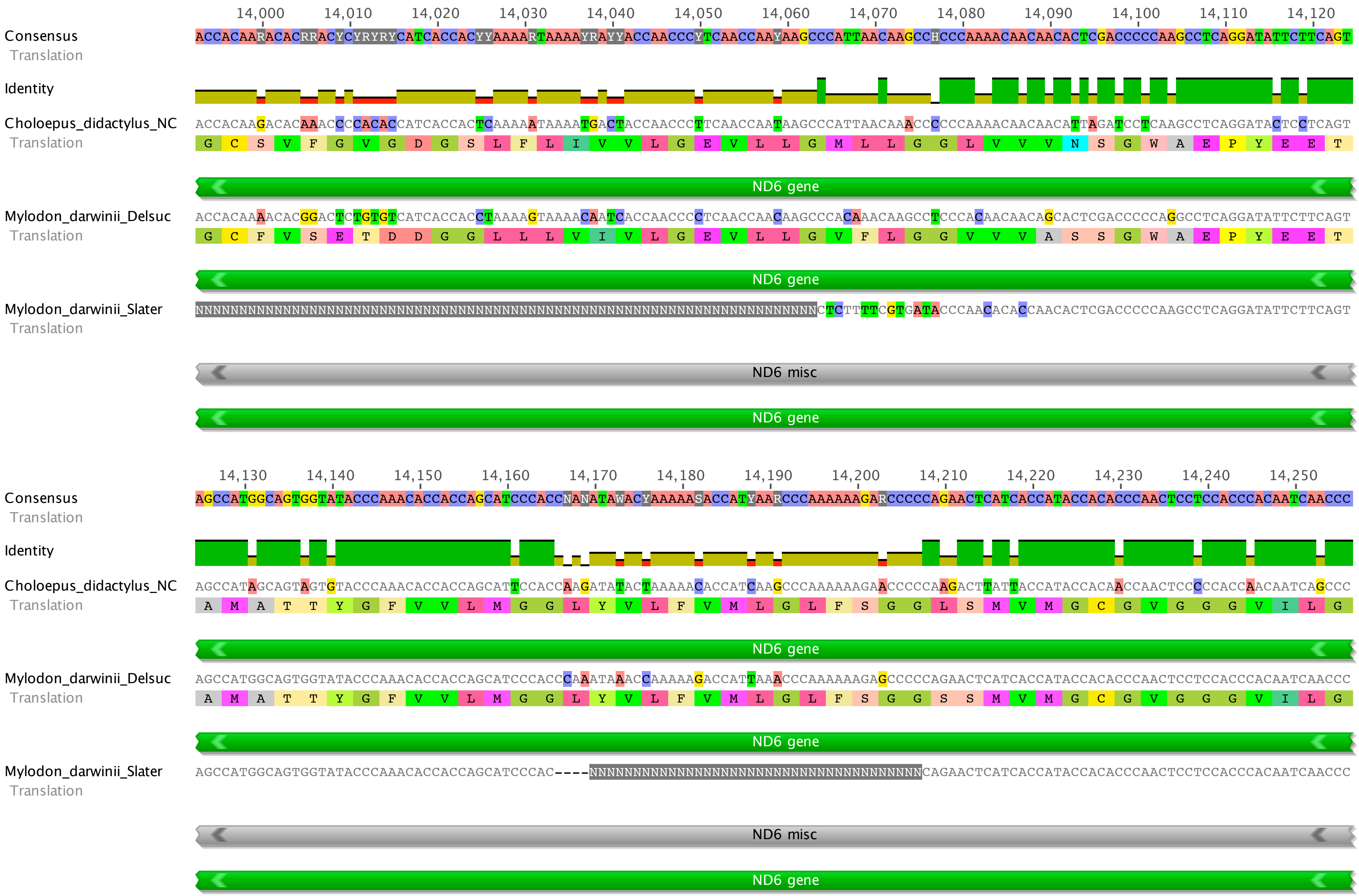
14,000 14,010 14,020 14,030 14,040 14,050 14,060 14,070 14,080 14,090 14,100 14,110 14,120
Consensus ACCACAARACACRRACYCYRYRYCATCACCACYYAAAARTAAAAYRAYYACCAACCCYTCAACCAAYAAGCCCATTAACAAGCCHCCCAAAACAACAACACTCGACCCCCAAGCCTCAGGATATTCTTCAGT
Translation
Identity
Choloepus_didactylus_NC ACCACAAGACACAAACCCACACCATCACCACTCAAAAATAAAATGACTACCAACCCTTCAACCAATAAGCCCATTAACAAACCCCCCAAAACAACAACATTAGATCCTCAAGCCTCAGGATACTCCTCAGT
Translation G C S V F G V G D G S L F L I V V L G E V L L G M L L G G L V V V N S G W A E P Y E E T
ND6 gene
Mylodon_darwinii_Delsuc ACCACAAAACACGGACTCTGTGTCATCACCACCTAAAAGTAAAACAATCACCAACCCCTCAACCAACAAGCCCACAAACAAGCCTCCCACAACAACAGCACTCGACCCCCAGGCCTCAGGATATTCTTCAGT
Translation G C F V S E T D D G G L L L V I V L G E V L L G V F L G G V V V A S S G W A E P Y E E T
ND6 gene
Mylodon_darwinii_Slater NNNNNNNNNNNNNNNNNNNNNNNNNNNNNNNNNNNNNNNNNNNNNNNNNNNNNNNNNNNNNNNNNNNNNNNCTCTTTTCGTGATACCCAACACACCAACACTCGACCCCCAAGCCTCAGGATATTCTTCAGT
Translation
ND6 misc
ND6 gene
14,130 14,140 14,150 14,160 14,170 14,180 14,190 14,200 14,210 14,220 14,230 14,240 14,250
Consensus AGCCATGGCAGTGGTATACCCAAACACCACCAGCATCCCACCNANATAWACYAAAAASACCATYAARCCCAAAAAAGARCCCCCAGAACTCATCACCATACCACACCCAACTCCTCCACCCACAATCAACCC
Translation
Identity
Choloepus_didactylus_NC AGCCATAGCAGTAGTGTACCCAAACACCACCAGCATTCCACCAAGATATACTAAAAACACCATCAAGCCCAAAAAAGAACCCCCAAGACTTATTACCATACCACAACCAACTCCCCCACCAACAATCAGCCC
Translation A M A T T Y G F V V L M G G L Y V L F V M L G L F S G G L S M V M G C G V G G G V I L G
ND6 gene
Mylodon_darwinii_Delsuc AGCCATGGCAGTGGTATACCCAAACACCACCAGCATCCCACCCAAATAAACCAAAAAGACCATTAAACCCAAAAAAGAGCCCCCAGAACTCATCACCATACCACACCCAACTCCTCCACCCACAATCAACCC
Translation A M A T T Y G F V V L M G G L Y V L F V M L G L F S G G S S M V M G C G V G G G V I L G
ND6 gene
Mylodon_darwinii_Slater AGCCATGGCAGTGGTATACCCAAACACCACCAGCATCCCAC----NNNNNNNNNNNNNNNNNNNNNNNNNNNNNNNNNNNNNNCAGAACTCATCACCATACCACACCCAACTCCTCCACCCACAATCAACCC
Translation
ND6 misc
ND6 gene

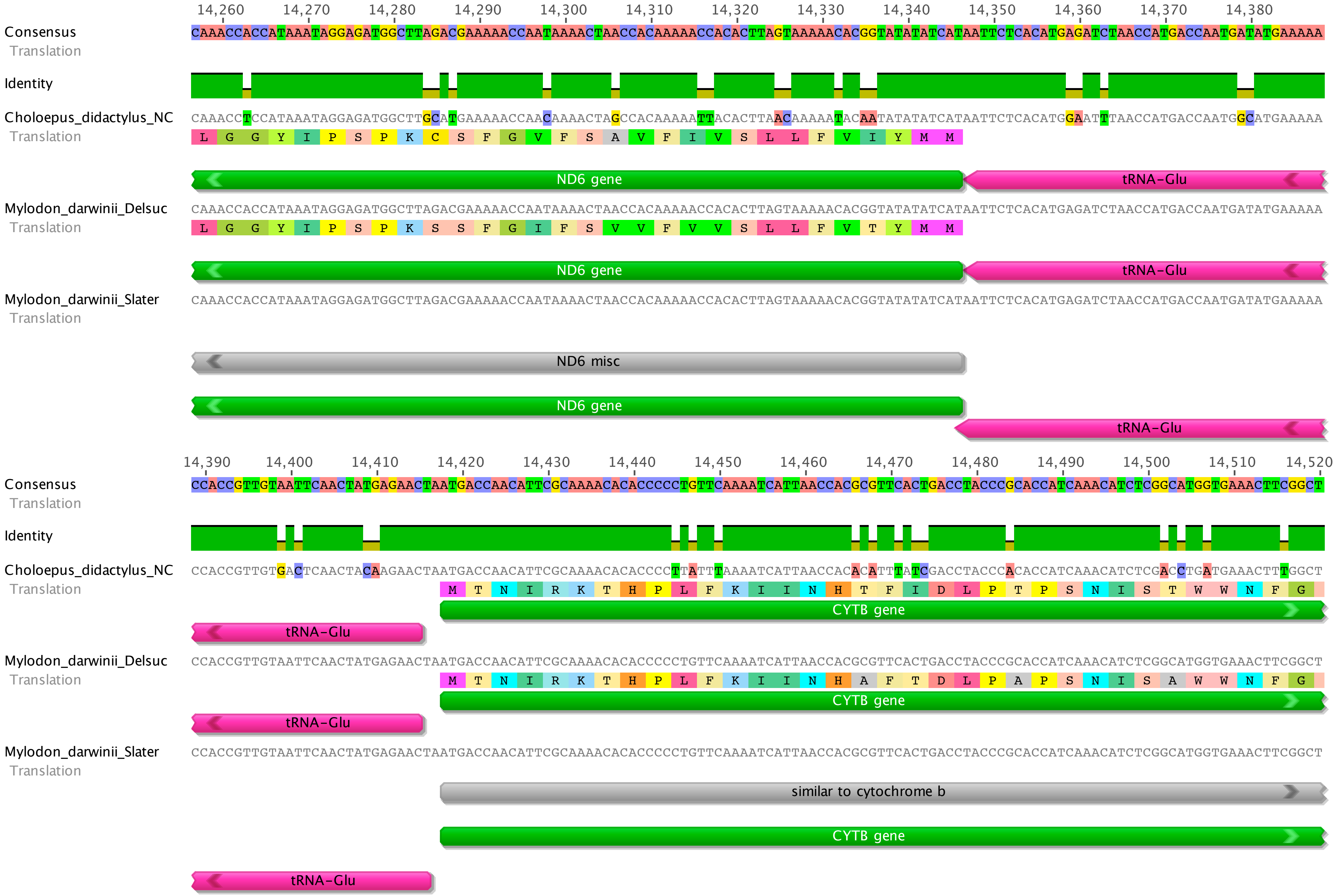

14,260 14,270 14,280 14,290 14,300 14,310 14,320 14,330 14,340 14,350 14,360 14,370 14,380
Consensus
CAAACCACCATAAATAGGAGATGGCTTAGACGAAAAACCAATAAAACTAACCACAAAAACCACACTTAGTAAAAACACGGTATATATCATAATTCTCACATGAGATCTAACCATGACCAATGATATGAAAAA
Translation
Identity
Choloepus_didactylus_NC
CAAACCTCCATAAATAGGAGATGGCTTGCATGAAAAACCAACAAAACTAGCCACAAAAATTACACTTAACAAAAATACAATATATATCATAATTCTCACATGGAATTTAACCATGACCAATGGCATGAAAAA
Translation
L G G Y I P S P K C S F G V F S A V F I V S L L F V I Y M M
ND6 gene
tRNA-Glu
Mylodon_darwinii_Delsuc
CAAACCACCATAAATAGGAGATGGCTTAGACGAAAAACCAATAAAACTAACCACAAAAACCACACTTAGTAAAAACACGGTATATATCATAATTCTCACATGAGATCTAACCATGACCAATGATATGAAAAA
Translation
L G G Y I P S P K S S F G I F S V V F V V S L L F V T Y M M
ND6 gene
tRNA-Glu
Mylodon_darwinii_Slater
CAAACCACCATAAATAGGAGATGGCTTAGACGAAAAACCAATAAAACTAACCACAAAAACCACACTTAGTAAAAACACGGTATATATCATAATTCTCACATGAGATCTAACCATGACCAATGATATGAAAAA
Translation
ND6 misc
ND6 gene
tRNA-Glu
14,390 14,400 14,410 14,420 14,430 14,440 14,450 14,460 14,470 14,480 14,490 14,500 14,510 14,520
Consensus
CCACCGTTGTAATTCAACTATGAGAACTAATGACCAACATTCGCAAAACACACCCCCTGTTCAAAATCATTAACCACGCGTTCACTGACCTACCCGCACCATCAAACATCTCGGCATGGTGAAACTTCGGCT
Translation
Identity
Choloepus_didactylus_NC
CCACCGTTGTGACTCAACTACAAGAACTAATGACCAACATTCGCAAAACACACCCCTTATTTAAAATCATTAACCACACATTTATCGACCTACCCACACCATCAAACATCTCGACCTGATGAAACTTTGGCT
Translation
M T N I R K T H P L F K I I N H T F I D L P T P S N I S T W W N F G
CYTB gene
tRNA-Glu
Mylodon_darwinii_Delsuc
CCACCGTTGTAATTCAACTATGAGAACTAATGACCAACATTCGCAAAACACACCCCCTGTTCAAAATCATTAACCACGCGTTCACTGACCTACCCGCACCATCAAACATCTCGGCATGGTGAAACTTCGGCT
Translation
M T N I R K T H P L F K I I N H A F T D L P A P S N I S A W W N F G
CYTB gene
tRNA-Glu
Mylodon_darwinii_Slater
CCACCGTTGTAATTCAACTATGAGAACTAATGACCAACATTCGCAAAACACACCCCCTGTTCAAAATCATTAACCACGCGTTCACTGACCTACCCGCACCATCAAACATCTCGGCATGGTGAAACTTCGGCT
Translation
similar to cytochrome b
CYTB gene
tRNA-Glu

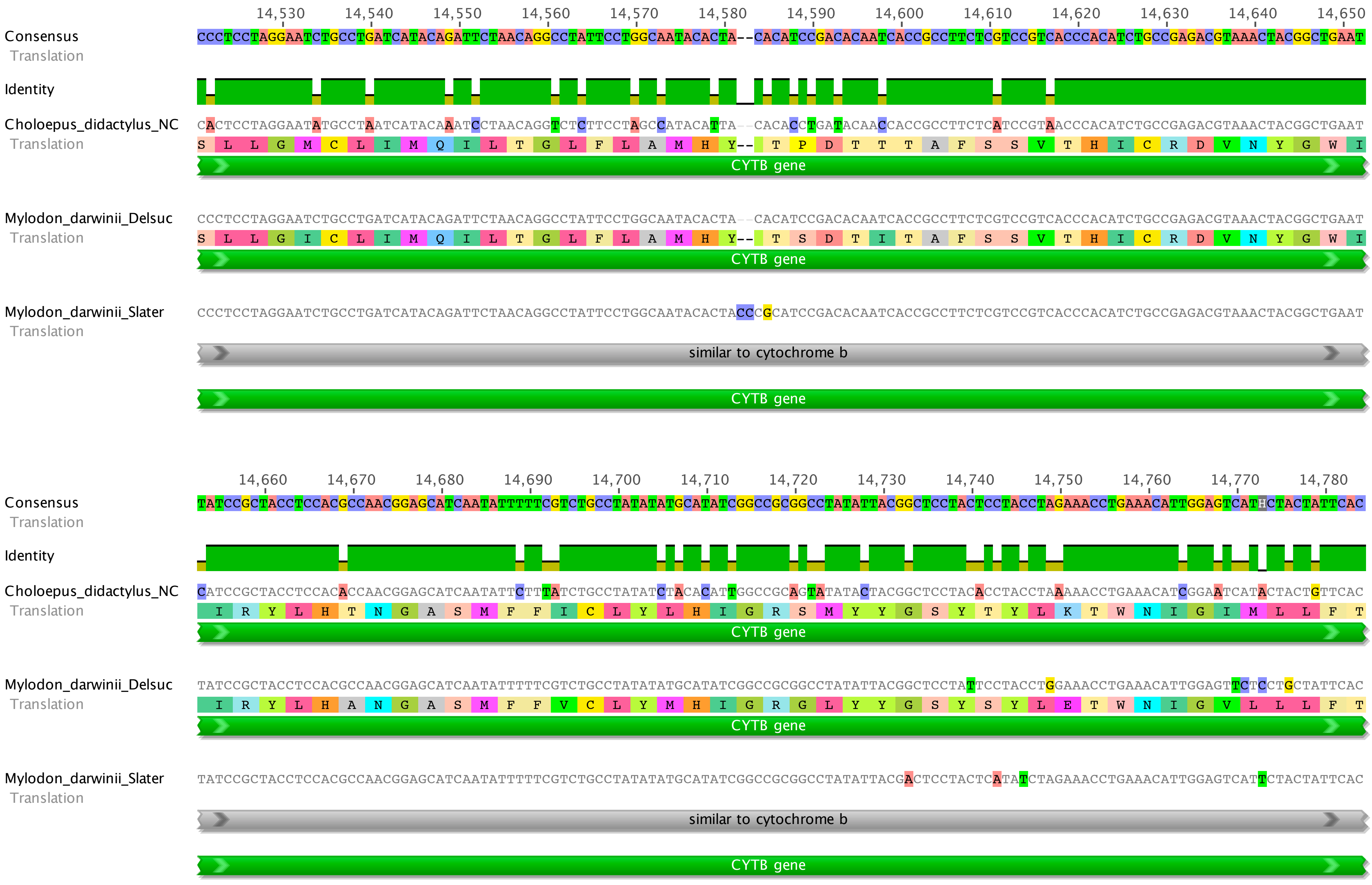
14,530
14,540
14,550
14,560
14,570
14,580
14,590
14,600
14,610
14,620
14,630
14,640
14,650
Consensus
Translation
Identity
Choloepus_didactylus_NC
Translation
CYTB gene
Mylodon_darwinii_Delsuc
Translation
CYTB gene
Mylodon_darwinii_Slater
Translation
similar to cytochrome b
CYTB gene
14,660
14,670
14,680
14,690
14,700
14,710
14,720
14,730
14,740
14,750
14,760
14,770
14,780
Consensus
Translation
Identity
Choloepus_didactylus_NC
Translation
CYTB gene
Mylodon_darwinii_Delsuc
Translation
CYTB gene
Mylodon_darwinii_Slater
Translation
similar to cytochrome b
CYTB gene

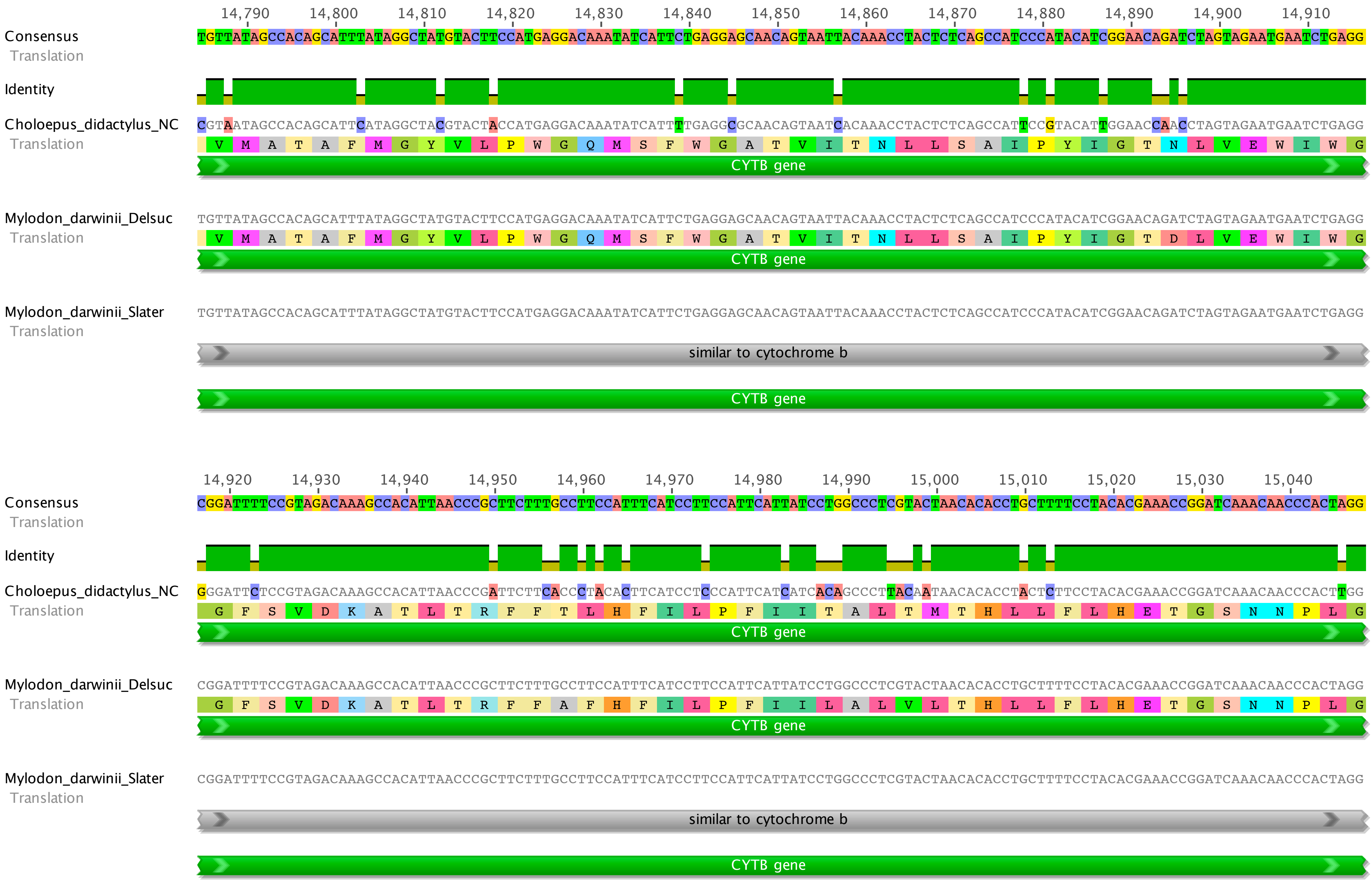
14,790 14,800 14,810 14,820 14,830 14,840 14,850 14,860 14,870 14,880 14,890 14,900 14,910
Consensus
TGTTATAGCCACAGCATTTATAGGCTATGTACTTCCATGAGGACAAATATCATTCTGAGGAGCAACAGTAATTACAAACCTACTCTCAGCCATCCCATACATCGGAACAGATCTAGTAGAATGAATCTGAGG
Translation
Identity
Choloepus_didactylus_NC
CGTAATAGCCACAGCATTCATAGGCTACGTACTACCATGAGGACAAATATCATTTTGAGGCGCAACAGTAATCACAAACCTACTCTCAGCCATTCCGTACATTGGAACCAACCTAGTAGAATGAATCTGAGG
Translation
V M A T A F M G Y V L P W G Q M S F W G A T V I T N L L S A I P Y I G T N L V E W I W G
CYTB gene
Mylodon_darwinii_Delsuc
TGTTATAGCCACAGCATTTATAGGCTATGTACTTCCATGAGGACAAATATCATTCTGAGGAGCAACAGTAATTACAAACCTACTCTCAGCCATCCCATACATCGGAACAGATCTAGTAGAATGAATCTGAGG
Translation
V M A T A F M G Y V L P W G Q M S F W G A T V I T N L L S A I P Y I G T D L V E W I W G
CYTB gene
Mylodon_darwinii_Slater
TGTTATAGCCACAGCATTTATAGGCTATGTACTTCCATGAGGACAAATATCATTCTGAGGAGCAACAGTAATTACAAACCTACTCTCAGCCATCCCATACATCGGAACAGATCTAGTAGAATGAATCTGAGG
Translation
similar to cytochrome b
CYTB gene
14,920 14,930 14,940 14,950 14,960 14,970 14,980 14,990 15,000 15,010 15,020 15,030 15,040
Consensus
CGGATTTTCCGTAGACAAAGCCACATTAACCCGCTTCTTTGCCTTCCATTTCATCCTTCCATTCATTATCCTGGCCCTCGTACTAACACACCTGCTTTTCCTACACGAAACCGGATCAAACAACCCACTAGG
Translation
Identity
Choloepus_didactylus_NC
GGGATTCTCCGTAGACAAAGCCACATTAACCCGATTCTTCACCCTACACTTCATCCTCCCATTCATCATCACAGCCCTTACAATAACACACCTACTCTTCCTACACGAAACCGGATCAAACAACCCACTTGG
Translation
G F S V D K A T L T R F F T L H F I L P F I I T A L T M T H L L F L H E T G S N N P L G
CYTB gene
Mylodon_darwinii_Delsuc
CGGATTTTCCGTAGACAAAGCCACATTAACCCGCTTCTTTGCCTTCCATTTCATCCTTCCATTCATTATCCTGGCCCTCGTACTAACACACCTGCTTTTCCTACACGAAACCGGATCAAACAACCCACTAGG
Translation
G F S V D K A T L T R F F A F H F I L P F I I L A L V L T H L L F L H E T G S N N P L G
CYTB gene
Mylodon_darwinii_Slater
CGGATTTTCCGTAGACAAAGCCACATTAACCCGCTTCTTTGCCTTCCATTTCATCCTTCCATTCATTATCCTGGCCCTCGTACTAACACACCTGCTTTTCCTACACGAAACCGGATCAAACAACCCACTAGG
Translation
similar to cytochrome b
CYTB gene

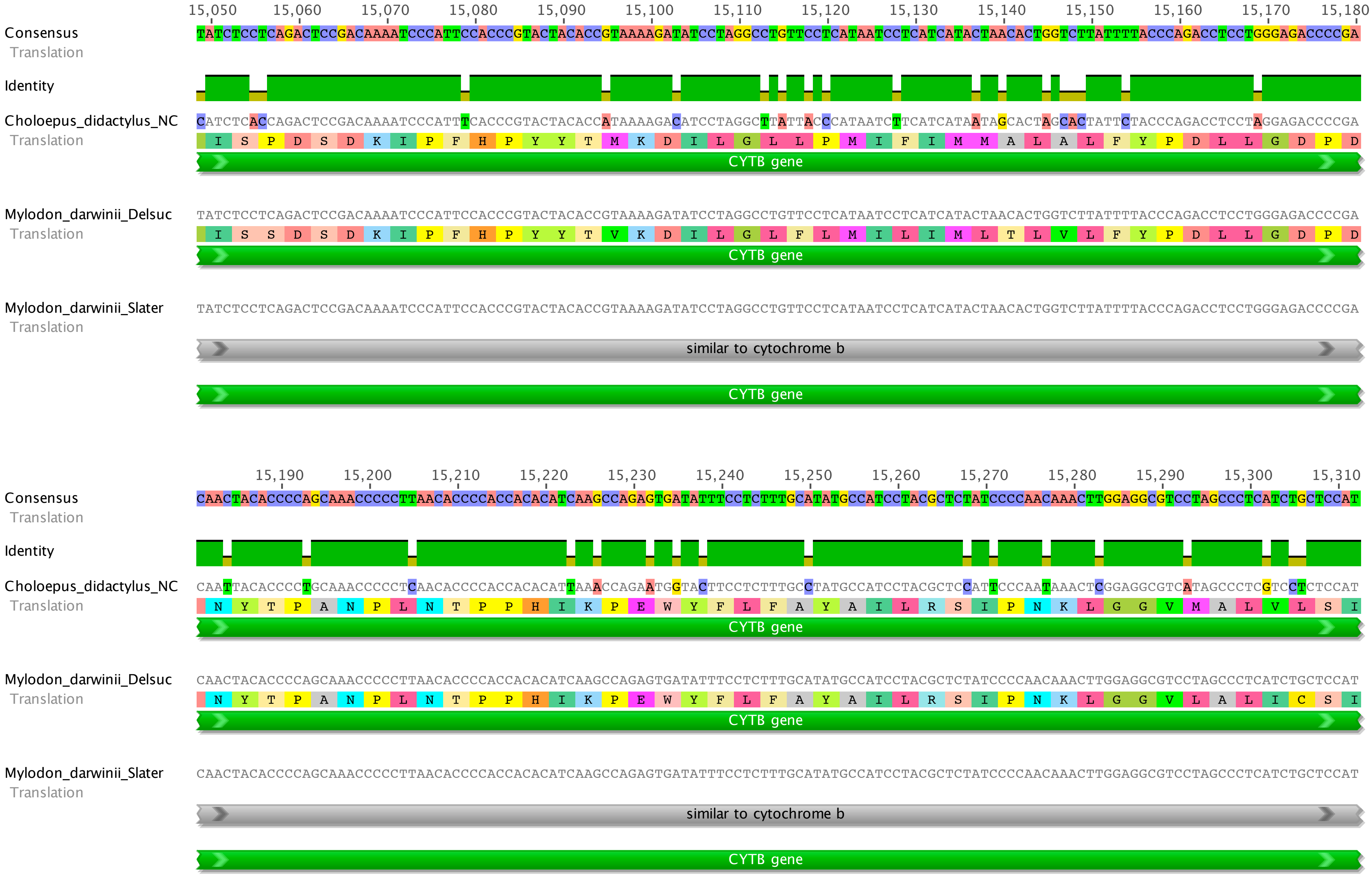
15,050 15,060 15,070 15,080 15,090 15,100 15,110 15,120 15,130 15,140 15,150 15,160 15,170 15,180
Consensus
TATCTCCTCAGACTCCGACAAAATCCCATTCCACCCGTACTACACCGTAAAAGATATCCTAGGCCTGTTCCTCATAATCCTCATCATACTAACACTGGTCTTATTTTACCCAGACCTCCTGGGAGACCCCGA
Translation
Identity
Choloepus_didactylus_NC
CATCTCACCAGACTCCGACAAAATCCCATTTCACCCGTACTACACCATAAAAGACATCCTAGGCTTATTACCCATAATCTTCATCATAATAGCACTAGCACTATTCTACCCAGACCTCCTAGGAGACCCCGA
Translation
I S P D S D K I P F H P Y Y T M K D I L G L L P M I F I M M A L A L F Y P D L L G D P D
CYTB gene
Mylodon_darwinii_Delsuc
TATCTCCTCAGACTCCGACAAAATCCCATTCCACCCGTACTACACCGTAAAAGATATCCTAGGCCTGTTCCTCATAATCCTCATCATACTAACACTGGTCTTATTTTACCCAGACCTCCTGGGAGACCCCGA
Translation
I S S D S D K I P F H P Y Y T V K D I L G L F L M I L I M L T L V L F Y P D L L G D P D
CYTB gene
Mylodon_darwinii_Slater
TATCTCCTCAGACTCCGACAAAATCCCATTCCACCCGTACTACACCGTAAAAGATATCCTAGGCCTGTTCCTCATAATCCTCATCATACTAACACTGGTCTTATTTTACCCAGACCTCCTGGGAGACCCCGA
Translation
similar to cytochrome b
CYTB gene
15,190 15,200 15,210 15,220 15,230 15,240 15,250 15,260 15,270 15,280 15,290 15,300 15,310
Consensus
CAACTACACCCCAGCAAACCCCCTTAACACCCCACCACACATCAAGCCAGAGTGATATTTCCTCTTTGCATATGCCATCCTACGCTCTATCCCCAACAAACTTGGAGGCGTCCTAGCCCTCATCTGCTCCAT
Translation
Identity
Choloepus_didactylus_NC
CAATTACACCCCTGCAAACCCCCTCAACACCCCACCACACATTAAACCAGAATGGTACTTCCTCTTTGCCTATGCCATCCTACGCTCCATTCCCAATAAACTCGGAGGCGTCATAGCCCTCGTCCTCTCCAT
Translation
N Y T P A N P L N T P P H I K P E W Y F L F A Y A I L R S I P N K L G G V M A L V L S I
CYTB gene
Mylodon_darwinii_Delsuc
CAACTACACCCCAGCAAACCCCCTTAACACCCCACCACACATCAAGCCAGAGTGATATTTCCTCTTTGCATATGCCATCCTACGCTCTATCCCCAACAAACTTGGAGGCGTCCTAGCCCTCATCTGCTCCAT
Translation
N Y T P A N P L N T P P H I K P E W Y F L F A Y A I L R S I P N K L G G V L A L I C S I
CYTB gene
Mylodon_darwinii_Slater
CAACTACACCCCAGCAAACCCCCTTAACACCCCACCACACATCAAGCCAGAGTGATATTTCCTCTTTGCATATGCCATCCTACGCTCTATCCCCAACAAACTTGGAGGCGTCCTAGCCCTCATCTGCTCCAT
Translation
similar to cytochrome b
CYTB gene

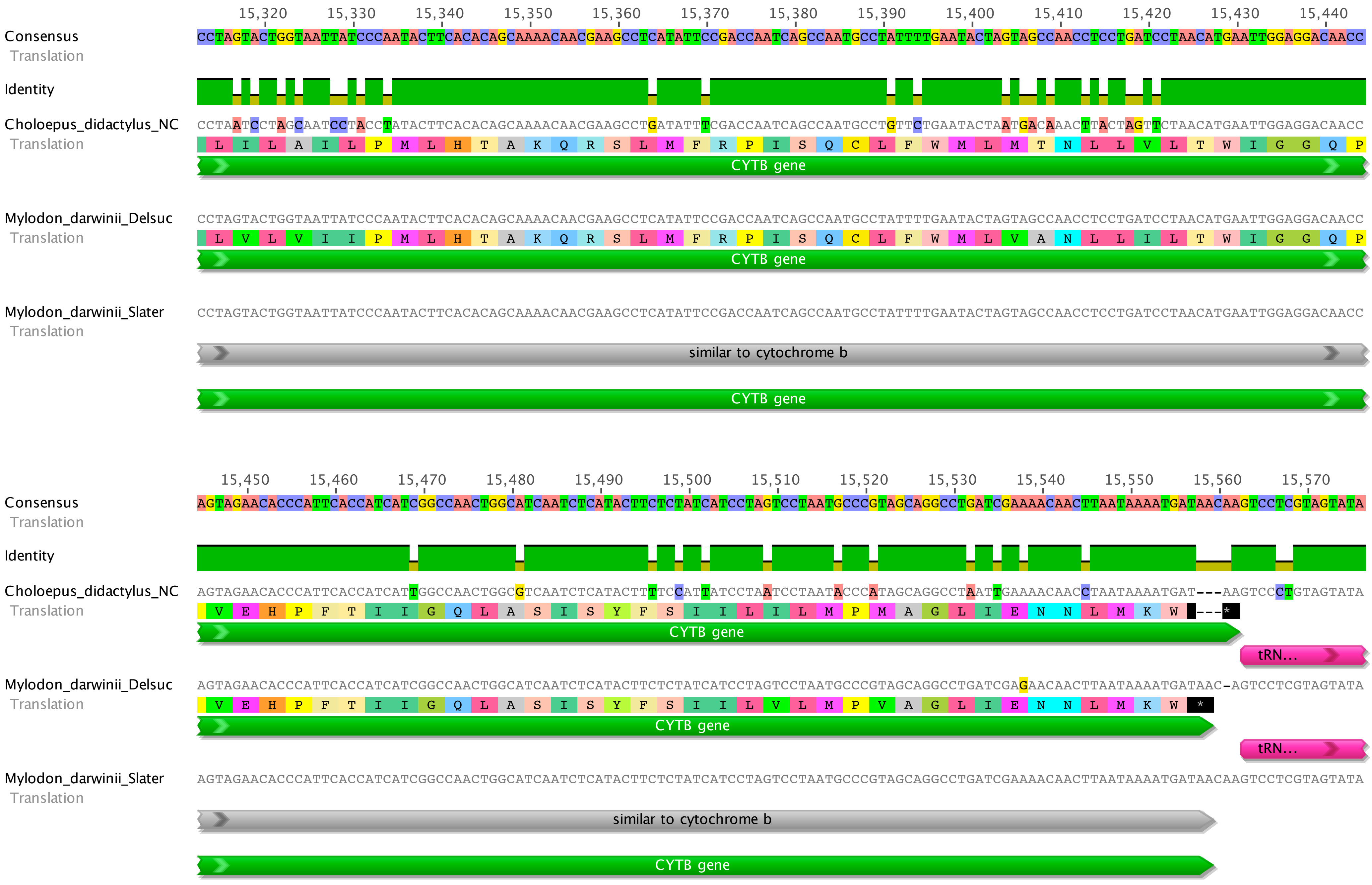
15,320 15,330 15,340 15,350 15,360 15,370 15,380 15,390 15,400 15,410 15,420 15,430 15,440
Consensus
Translation
Identity
Choloepus_didactylus_NC
Translation
CYTB gene
Mylodon_darwinii_Delsuc
Translation
CYTB gene
Mylodon_darwinii_Slater
Translation
similar to cytochrome b
CYTB gene
15,450 15,460 15,470 15,480 15,490 15,500 15,510 15,520 15,530 15,540 15,550 15,560 15,570
Consensus
Translation
Identity
Choloepus_didactylus_NC
Translation
CYTB gene
tRN...
Mylodon_darwinii_Delsuc
Translation
CYTB gene
tRN...
Mylodon_darwinii_Slater
Translation
similar to cytochrome b
CYTB gene

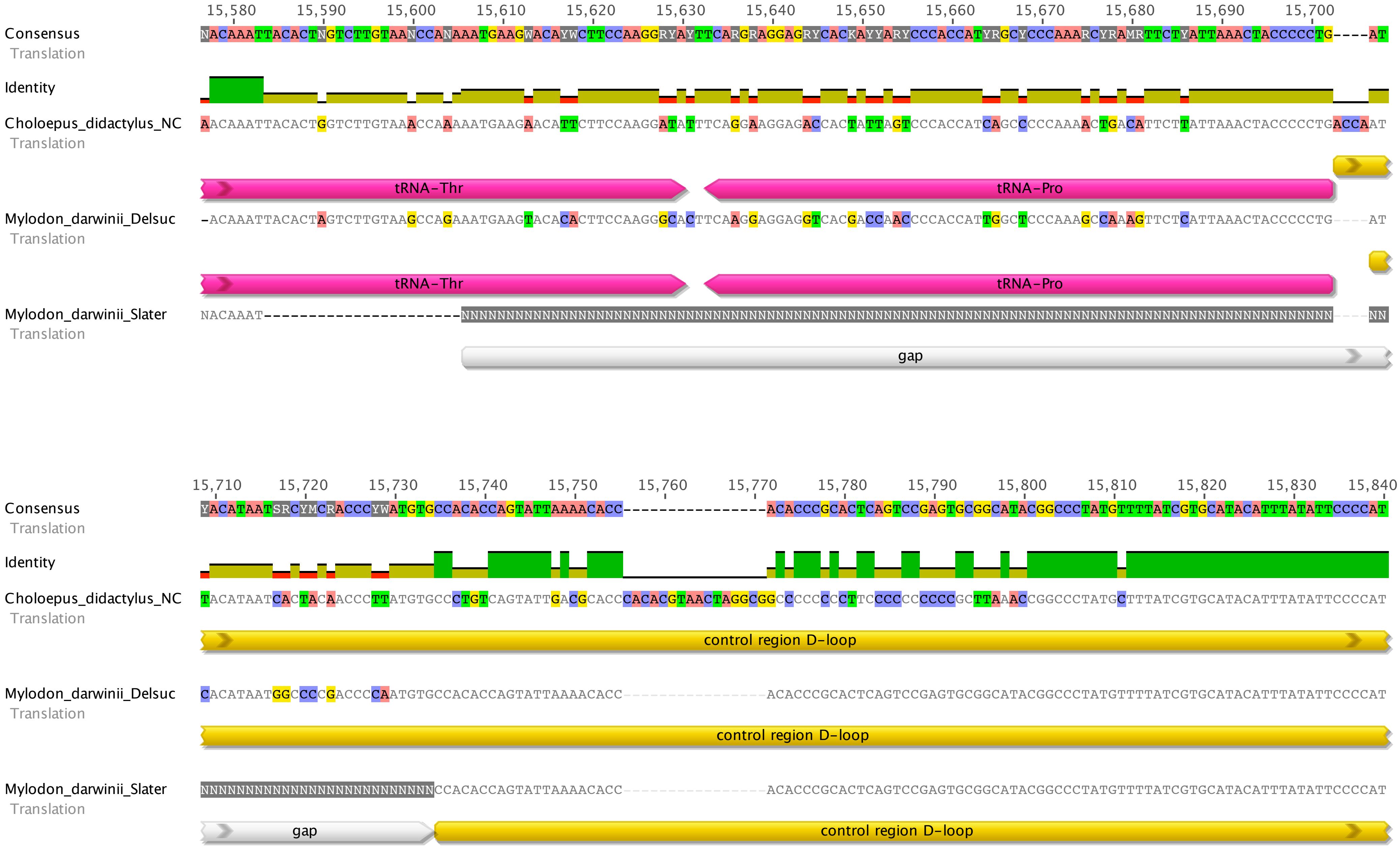
15,580 15,590 15,600 15,610 15,620 15,630 15,640 15,650 15,660 15,670 15,680 15,690 15,700
Consensus
Translation
Identity
Choloepus_didactylus_NC
Translation
tRNA-Thr
tRNA-Pro
Mylodon_darwinii_Delsuc
Translation
tRNA-Thr
tRNA-Pro
Mylodon_darwinii_Slater
Translation
gap
15,710 15,720 15,730 15,740 15,750 15,760 15,770 15,780 15,790 15,800 15,810 15,820 15,830 15,840
Consensus
Translation
Identity
Choloepus_didactylus_NC
Translation
control region D-loop
Mylodon_darwinii_Delsuc
Translation
control region D-loop
Mylodon_darwinii_Slater
Translation
gap
control region D-loop

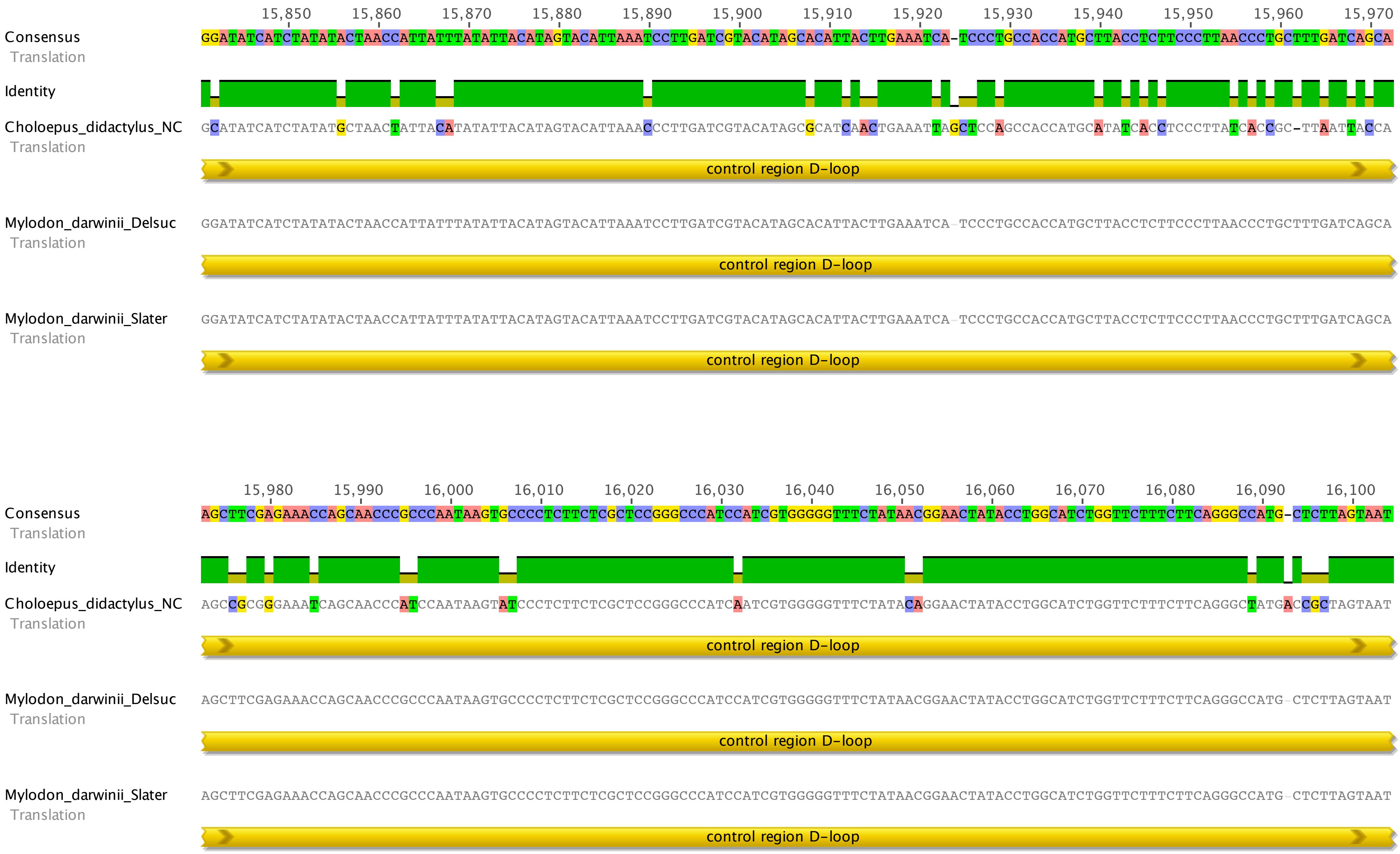
15,850 15,860 15,870 15,880 15,890 15,900 15,910 15,920 15,930 15,940 15,950 15,960 15,970
Consensus GGATATCATCTATATACTAACCATTATTTATATTACATAGTACATTAAATCCTTGATCGTACATAGCACATTACTTGAAATCA-TCCCTGCCACCATGCTTACCTCTTCCCTTAACCCTGCTTTGATCAGCA
Translation
Identity
Choloepus_didactylus_NC GCATATCATCTATATGCTAACTATTACATATATTACATAGTACATTAAACCCTTGATCGTACATAGCGCATCAACTGAAATTAGCTCCAGCCACCATGCATATCACCTCCCTTATCACCGC-TTAATTACCA
Translation
control region D-loop
Mylodon_darwinii_Delsuc GGATATCATCTATATACTAACCATTATTTATATTACATAGTACATTAAATCCTTGATCGTACATAGCACATTACTTGAAATCA-TCCCTGCCACCATGCTTACCTCTTCCCTTAACCCTGCTTTGATCAGCA
Translation
control region D-loop
Mylodon_darwinii_Slater GGATATCATCTATATACTAACCATTATTTATATTACATAGTACATTAAATCCTTGATCGTACATAGCACATTACTTGAAATCA-TCCCTGCCACCATGCTTACCTCTTCCCTTAACCCTGCTTTGATCAGCA
Translation
control region D-loop
15,980 15,990 16,000 16,010 16,020 16,030 16,040 16,050 16,060 16,070 16,080 16,090 16,100
Consensus AGCTTCGAGAAACCAGCAACCCGCCCAATAAGTGCCCCTCTTCTCGCTCCGGGCCCATCCATCGTGGGGGTTTCTATAACGGAACTATACCTGGCATCTGGTTCTTTCTTCAGGGCCATG-CTCTTAGTAAT
Translation
Identity
Choloepus_didactylus_NC AGCCGCGGGAAATCAGCAACCCATCCAATAAGTATCCCTCTTCTCGCTCCGGGCCCATCAATCGTGGGGGTTTCTATACAGGAACTATACCTGGCATCTGGTTCTTTCTTCAGGGCTATGACCGCTAGTAAT
Translation
control region D-loop
Mylodon_darwinii_Delsuc AGCTTCGAGAAACCAGCAACCCGCCCAATAAGTGCCCCTCTTCTCGCTCCGGGCCCATCCATCGTGGGGGTTTCTATAACGGAACTATACCTGGCATCTGGTTCTTTCTTCAGGGCCATG-CTCTTAGTAAT
Translation
control region D-loop
Mylodon_darwinii_Slater AGCTTCGAGAAACCAGCAACCCGCCCAATAAGTGCCCCTCTTCTCGCTCCGGGCCCATCCATCGTGGGGGTTTCTATAACGGAACTATACCTGGCATCTGGTTCTTTCTTCAGGGCCATG-CTCTTAGTAAT
Translation
control region D-loop

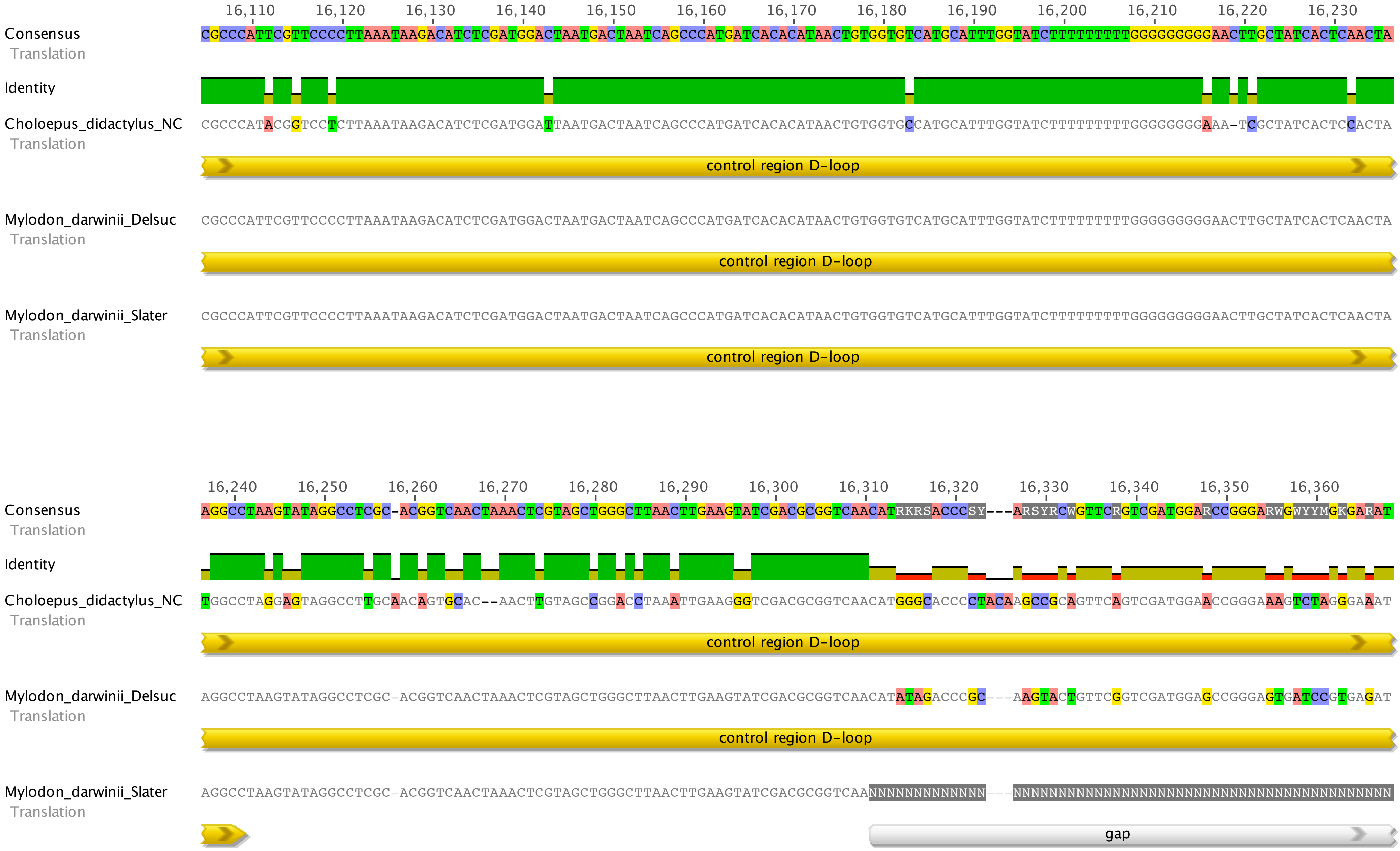
16,110 16,120 16,130 16,140 16,150 16,160 16,170 16,180 16,190 16,200 16,210 16,220 16,230
Consensus
CGCCCATTCGTTCCCCTTAAATAAGACATCTCGATGGACTAATGACTAATCAGCCCATGATCACACATAACTGTGGTGTCATGCATTTGGTATCTTTTTTTTTGGGGGGGGGAACTTGCTATCACTCAACTA
Translation
Identity
Choloepus_didactylus_NC
CGCCCATACGGTCCTCTTAAATAAGACATCTCGATGGATTAATGACTAATCAGCCCATGATCACACATAACTGTGGTGCCATGCATTTGGTATCTTTTTTTTTGGGGGGGAAA-TCGCTATCACTCCACTA
Translation
control region D-loop
Mylodon_darwinii_Delsuc
CGCCCATTCGTTCCCCTTAAATAAGACATCTCGATGGACTAATGACTAATCAGCCCATGATCACACATAACTGTGGTGTCATGCATTTGGTATCTTTTTTTTTGGGGGGGGGAACTTGCTATCACTCAACTA
Translation
control region D-loop
Mylodon_darwinii_Slater
CGCCCATTCGTTCCCCTTAAATAAGACATCTCGATGGACTAATGACTAATCAGCCCATGATCACACATAACTGTGGTGTCATGCATTTGGTATCTTTTTTTTTGGGGGGGGGAACTTGCTATCACTCAACTA
Translation
control region D-loop
16,240 16,250 16,260 16,270 16,280 16,290 16,300 16,310 16,320 16,330 16,340 16,350 16,360
Consensus
AGGCCTAAGTATAGGCCTCGC-ACGGTCAACTAAACTCGTAGCTGGGCTTAACTTGAAGTATCGACGCGGTCAACATRKRSACCCSY---ARSYRCWGTTCRGTCGATGGARCCGGGARWGWYYMGKGARAT
Translation
Identity
Choloepus_didactylus_NC
TGGCCTAGGAGTAGGCCTTGCAACAGTGCAC--AACTTGTAGCCGGACCTAAATTGAAGGGTCGACGCGGTCAACATGGGCACCCCTACAAGCCGCAGTTCAGTCGATGGAACCGGGAAAGTCTAGGGAAAT
Translation
control region D-loop
Mylodon_darwinii_Delsuc
AGGCCTAAGTATAGGCCTCGC-ACGGTCAACTAAACTCGTAGCTGGGCTTAACTTGAAGTATCGACGCGGTCAACATATAGACCCGC---AAGTACTGTTCGGTCGATGGAGCCGGGAGTGATCCGTGAGAT
Translation
control region D-loop
Mylodon_darwinii_Slater
AGGCCTAAGTATAGGCCTCGC-ACGGTCAACTAAACTCGTAGCTGGGCTTAACTTGAAGTATCGACGCGGTCAANNNNNNNNNNNNN---NNNNNNNNNNNNNNNNNNNNNNNNNNNNNNNNNNNNNNNNNN
Translation
gap

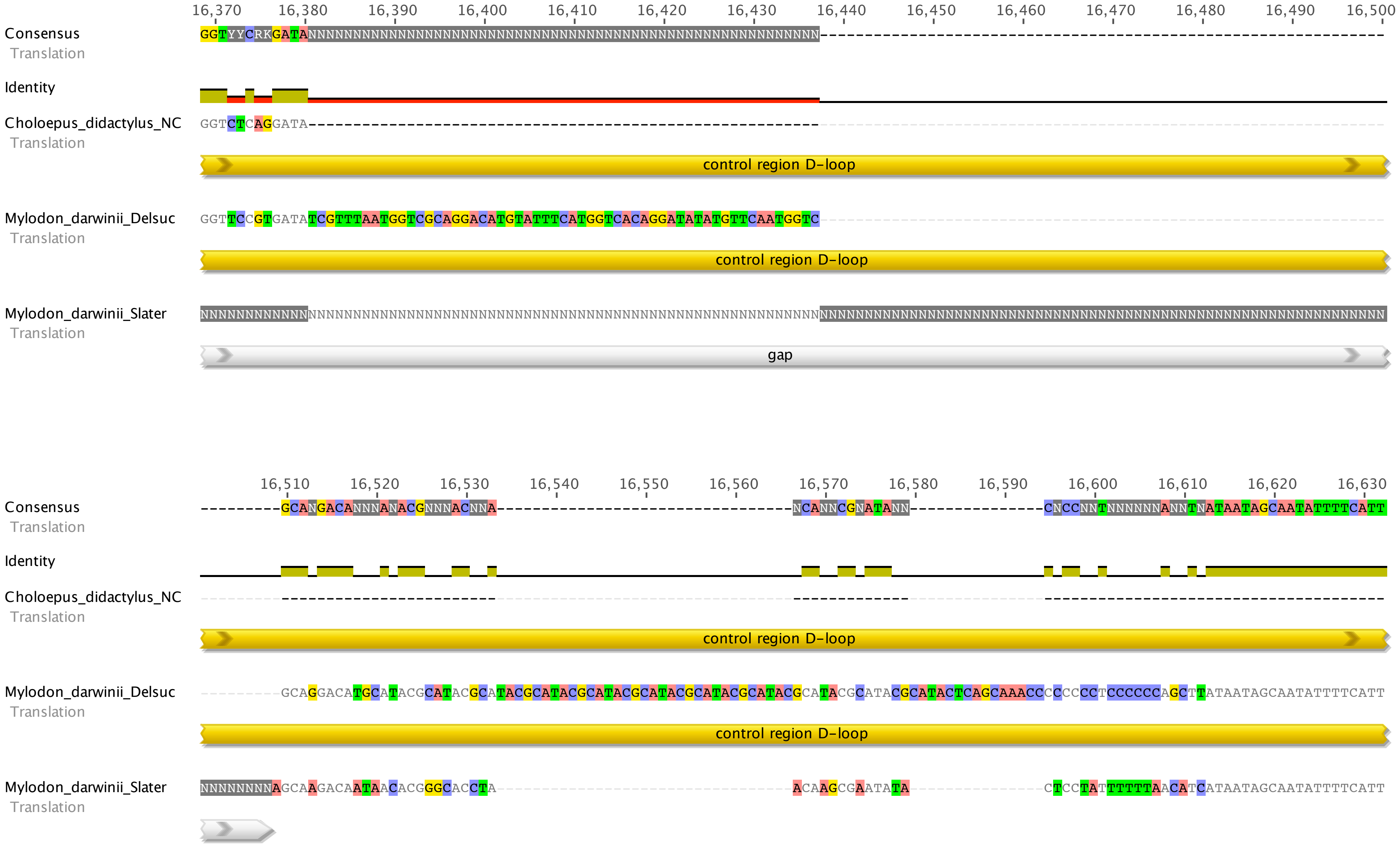

16,370 16,380 16,390 16,400 16,410 16,420 16,430 16,440 16,450 16,460 16,470 16,480 16,490 16,500
Consensus
GGTYYCRKGATANNNNNNNNNNNNNNNNNNNNNNNNNNNNNNNNNNNNNNNNNNNNNNNNNNNNN
Translation
Identity
Choloepus_didactylus_NC
GGTCTCAGGATA
Translation
control region D-loop
Mylodon_darwinii_Delsuc
GGTTCCGTGATATCGTTTAATGGTCGCAGGACATGTATTTCATGGTCACAGGATATATGTTCAATGGTC
Translation
control region D-loop
Mylodon_darwinii_Slater
NNNNNNNNNNNNNNNNNNNNNNNNNNNNNNNNNNNNNNNNNNNNNNNNNNNNNNNNNNNNNNNNNNNNNNNNNNNNNNNNNNNNNNNNNNNNNNNNNNNNNNNNNNNNNNNNNNNNNNNNNNNNNNNNNNN
Translation
gap
16,510 16,520 16,530 16,540 16,550 16,560 16,570 16,580 16,590 16,600 16,610 16,620 16,630
Consensus
GCANGACANNNANACGNNNACNNA
NCANNCGNATANN
CNCCNNTNNNNNNANNTNATAATAGCAATATTTTCATT
Translation
Identity
Choloepus_didactylus_NC
Translation
control region D-loop
Mylodon_darwinii_Delsuc
GCAGGACATGCATACGCATACGCATACGCATACGCATACGCATACGCATACGCATACGCATACGCATACGCATACTCAGCAAACCCCCCCTCCCCCCAGCTTATAATAGCAATATTTTCATT
Translation
control region D-loop
Mylodon_darwinii_Slater
NNNNNNNNAGCAAGACAATAACACGGGCACCTA
ACAAGCGAATATA
CTCCTATTTTTTAACATCATAATAGCAATATTTTCATT
Translation

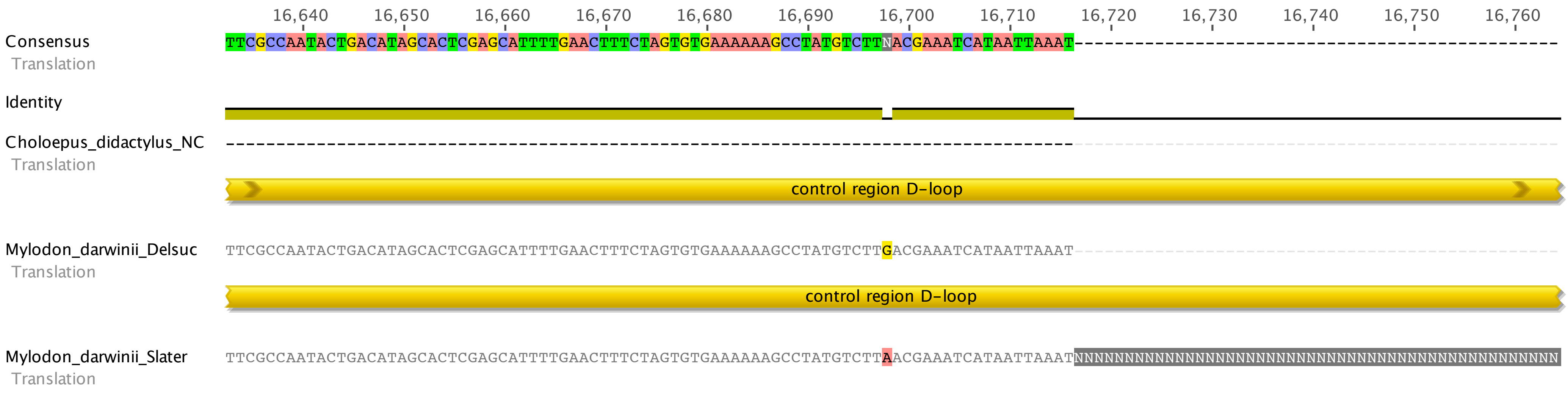
16,640
16,650
16,660
16,670
16,680
16,690
16,700
16,710
16,720
16,730
16,740
16,750
16,760
Consensus
TTCGCCAATACTGACATAGCACTCGAGCATTTTGAACTTTCTAGTGTGAAAAAAGCCTATGTCTTNACGAAATCATAATTAAAT
Translation
Identity
Choloepus_didactylus_NC
Translation
control region D-loop
Mylodon_darwinii_Delsuc
TTCGCCAATACTGACATAGCACTCGAGCATTTTGAACTTTCTAGTGTGAAAAAAGCCTATGTCTTGACGAAATCATAATTAAAT
Translation
control region D-loop
Mylodon_darwinii_Slater
TTCGCCAATACTGACATAGCACTCGAGCATTTTGAACTTTCTAGTGTGAAAAAAGCCTATGTCTTAACGAAATCATAATTAAAT
Translation

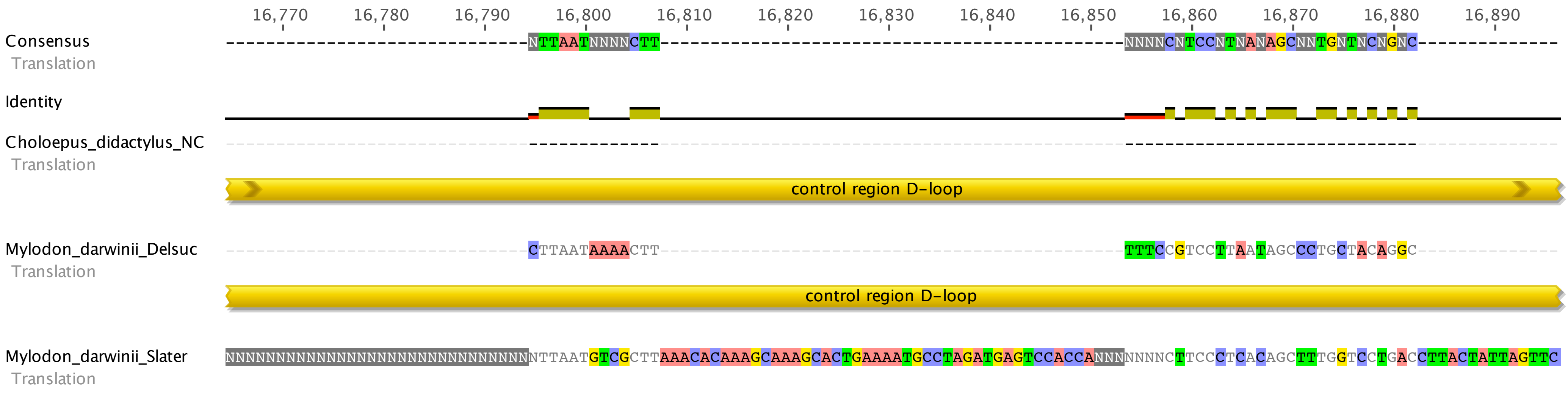
16,770
16,780
16,790
16,800
16,810
16,820
16,830
16,840
16,850
16,860
16,870
16,880
16,890
Consensus
NTTAATNNNNCTT
NNNNCNTCCNTNANAGCNNTGNTNCNGNC
Translation
Identity
Choloepus_didactylus_NC
Translation
control region D-loop
Mylodon_darwinii_Delsuc
CTTAATAAAACTT
TTTCCGTCCTTAATAGCCCTGCTACAGGC
Translation
control region D-loop
Mylodon_darwinii_Slater
Translation

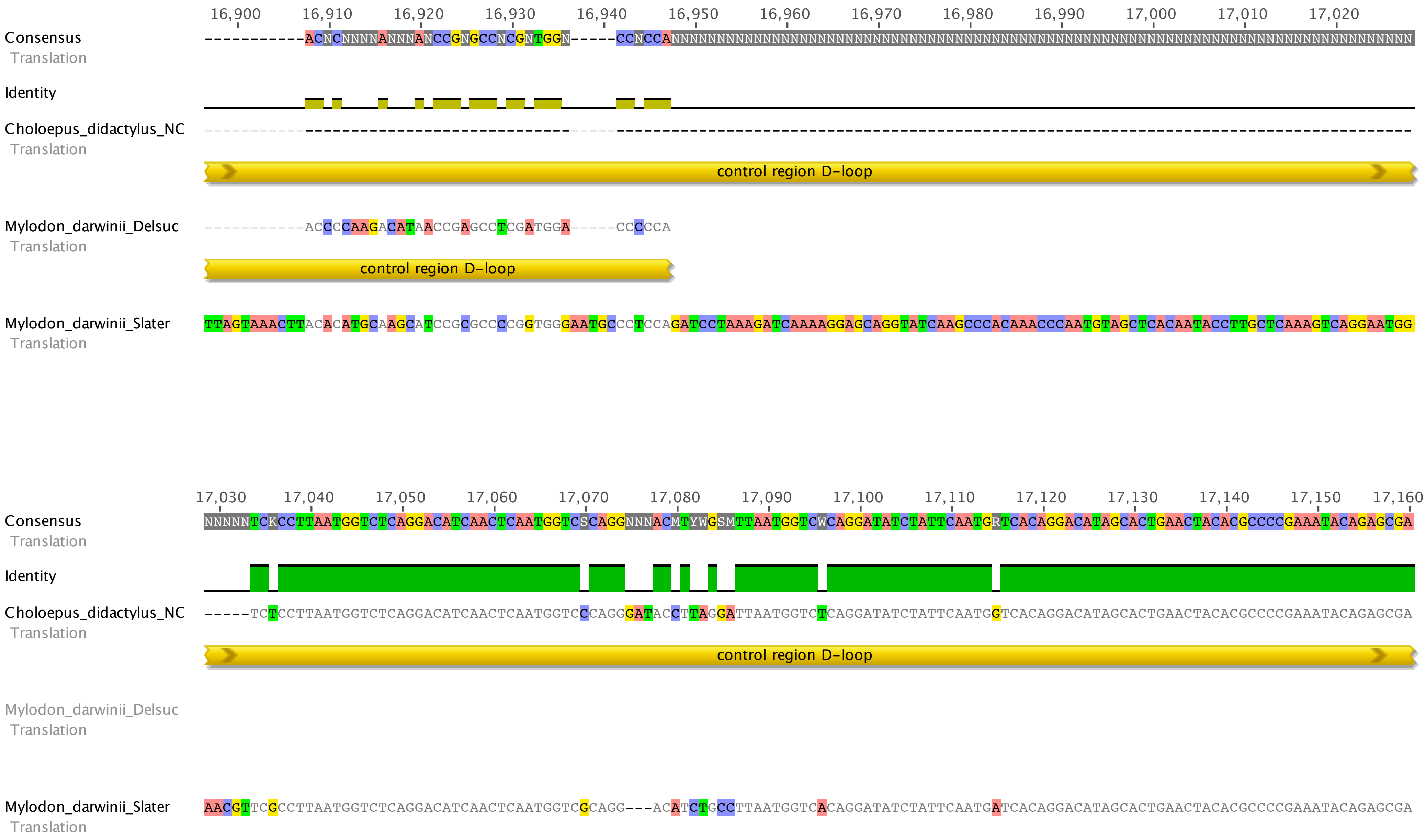
16,900 16,910 16,920 16,930 16,940 16,950 16,960 16,970 16,980 16,990 17,000 17,010 17,020
Consensus
-----------ACNCNNNNANNNANCCGNGCCNCGNTGGN-----CCNCCANNNNNNNNNNNNNNNNNNNNNNNNNNNNNNNNNNNNNNNNNNNNNNNNNNNNNNNNNNNNNNNNNNNNNNNNNNNNNNNNN
Translation
Identity
Choloepus_didactylus_NC
Translation
control region D-loop
Mylodon_darwinii_Delsuc
-----------ACCCCAAGACATAACCGAGCCTCGATGGA-----CCCCCA
Translation
control region D-loop
Mylodon_darwinii_Slater
TTAGTAAACTTACACATGCAAGCATCCGCGCCCCGGTGGGAATGCCCTCCAGATCCTAAAGATCAAAAGGAGCAGGTATCAAGCCCACAAACCCAATGTAGCTCACAATACCTTGCTCAAAGTCAGGAATGG
Translation
17,030 17,040 17,050 17,060 17,070 17,080 17,090 17,100 17,110 17,120 17,130 17,140 17,150 17,160
Consensus
NNNNNTCKCCTTAATGGTCTCAGGACATCAACTCAATGGTCSCAGGNNNACMTYWGSMTTAATGGTCWCAGGATATCTATTCAATGRTCACAGGACATAGCACTGAACTACACGCCCCGAAATACAGAGCGA
Translation
Identity
Choloepus_didactylus_NC
-----TCTCCTTAATGGTCTCAGGACATCAACTCAATGGTCCCAGGGATACCTTAGGATTAATGGTCTCAGGATATCTATTCAATGGTCACAGGACATAGCACTGAACTACACGCCCCGAAATACAGAGCGA
Translation
control region D-loop
Mylodon_darwinii_Delsuc
Translation
Mylodon_darwinii_Slater
AACGTTCGCCTTAATGGTCTCAGGACATCAACTCAATGGTCGCAGG---ACATCTGCCTTAATGGTCACAGGATATCTATTCAATGATCACAGGACATAGCACTGAACTACACGCCCCGAAATACAGAGCGA
Translation

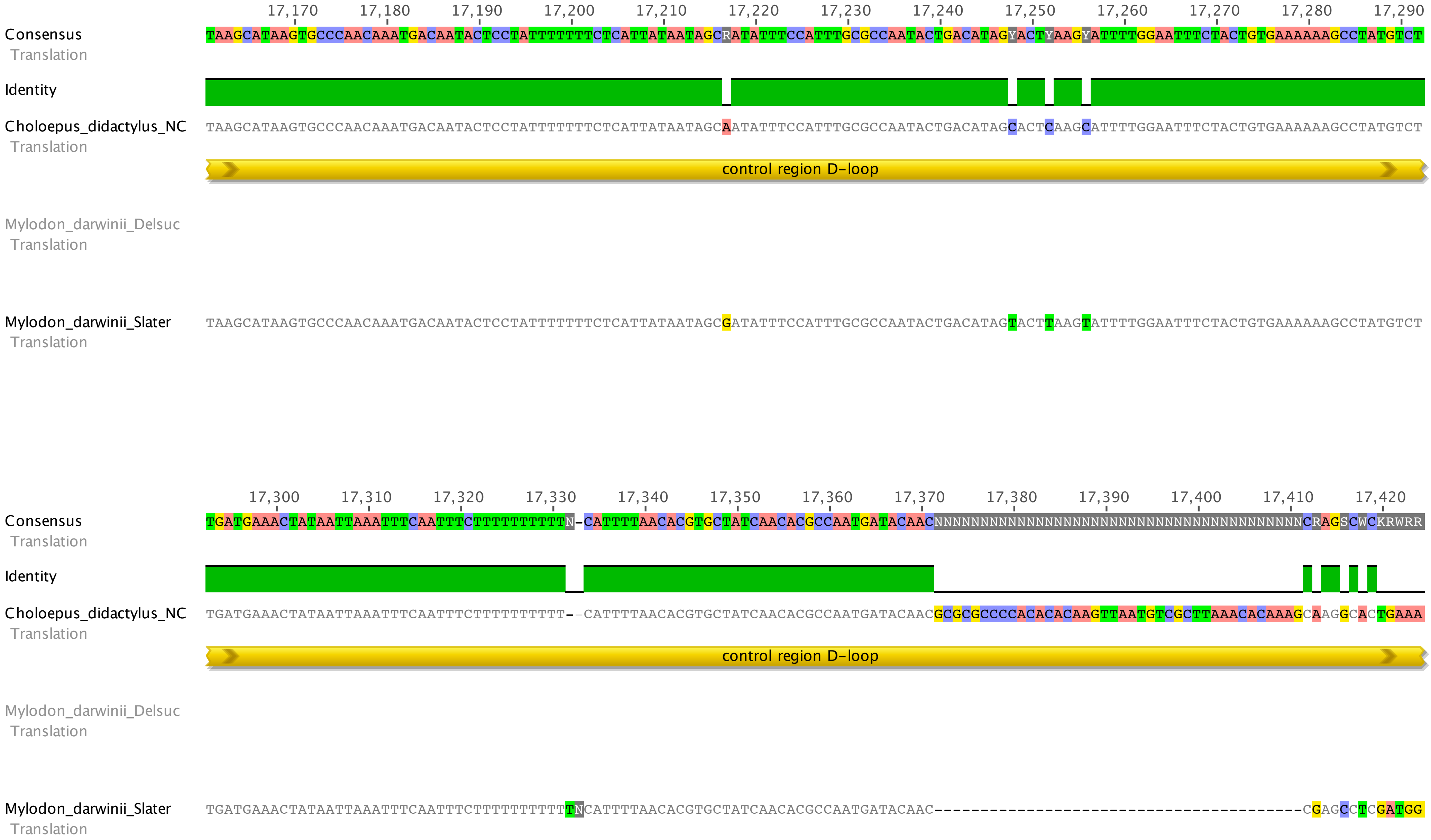

17,170 17,180 17,190 17,200 17,210 17,220 17,230 17,240 17,250 17,260 17,270 17,280 17,290
Consensus
TAAGCATAAGTGCCCAACAAATGACAATACTCCTATTTTTTTCTCATTATAATAGCRATATTTCCATTTGCGCCAATACTGACATAGYACTYAAGYATTTTGGAATTTCTACTGTGAAAAAAGCCTATGTCT
Translation
Identity
Choloepus_didactylus_NC
TAAGCATAAGTGCCCAACAAATGACAATACTCCTATTTTTTTCTCATTATAATAGCAATATTTCCATTTGCGCCAATACTGACATAGCACTCAAGCATTTTGGAATTTCTACTGTGAAAAAAGCCTATGTCT
Translation
control region D-loop
Mylodon_darwinii_Delsuc
Translation
Mylodon_darwinii_Slater
TAAGCATAAGTGCCCAACAAATGACAATACTCCTATTTTTTTCTCATTATAATAGCGATATTTCCATTTGCGCCAATACTGACATAGTACTTAAGTATTTTGGAATTTCTACTGTGAAAAAAGCCTATGTCT
Translation
17,300 17,310 17,320 17,330 17,340 17,350 17,360 17,370 17,380 17,390 17,400 17,410 17,420
Consensus
TGATGAAACTATAATTAAATTTCAATTTCTTTTTTTTTTTN-CATTTTAACACGTGCTATCAACACGCCAATGATACAACNNNNNNNNNNNNNNNNNNNNNNNNNNNNNNNNNNNNNNNNCRAGSCWCKRWRR
Translation
Identity
Choloepus_didactylus_NC
TGATGAAACTATAATTAAATTTCAATTTCTTTTTTTTTTT--CATTTTAACACGTGCTATCAACACGCCAATGATACAACGCGCGCCCCACACACAAGTTAATGTCGCTTAAACACAAAGCAAGGCACTGAAA
Translation
control region D-loop
Mylodon_darwinii_Delsuc
Translation
Mylodon_darwinii_Slater
TGATGAAACTATAATTAAATTTCAATTTCTTTTTTTTTTTTNCATTTTAACACGTGCTATCAACACGCCAATGATACAAC--CGAGCCTCGATGG
Translation

17,430 17,444

Consensus AYSCYYAGATGAGTACATCA
Translation

Identity

Choloepus_didactylus_NC ATGCTTAGATGAGTACATCA
Translation

control regio...

Mylodon_darwinii_Delsuc
Translation

Mylodon_darwinii_Slater ACCCCCA
Translation

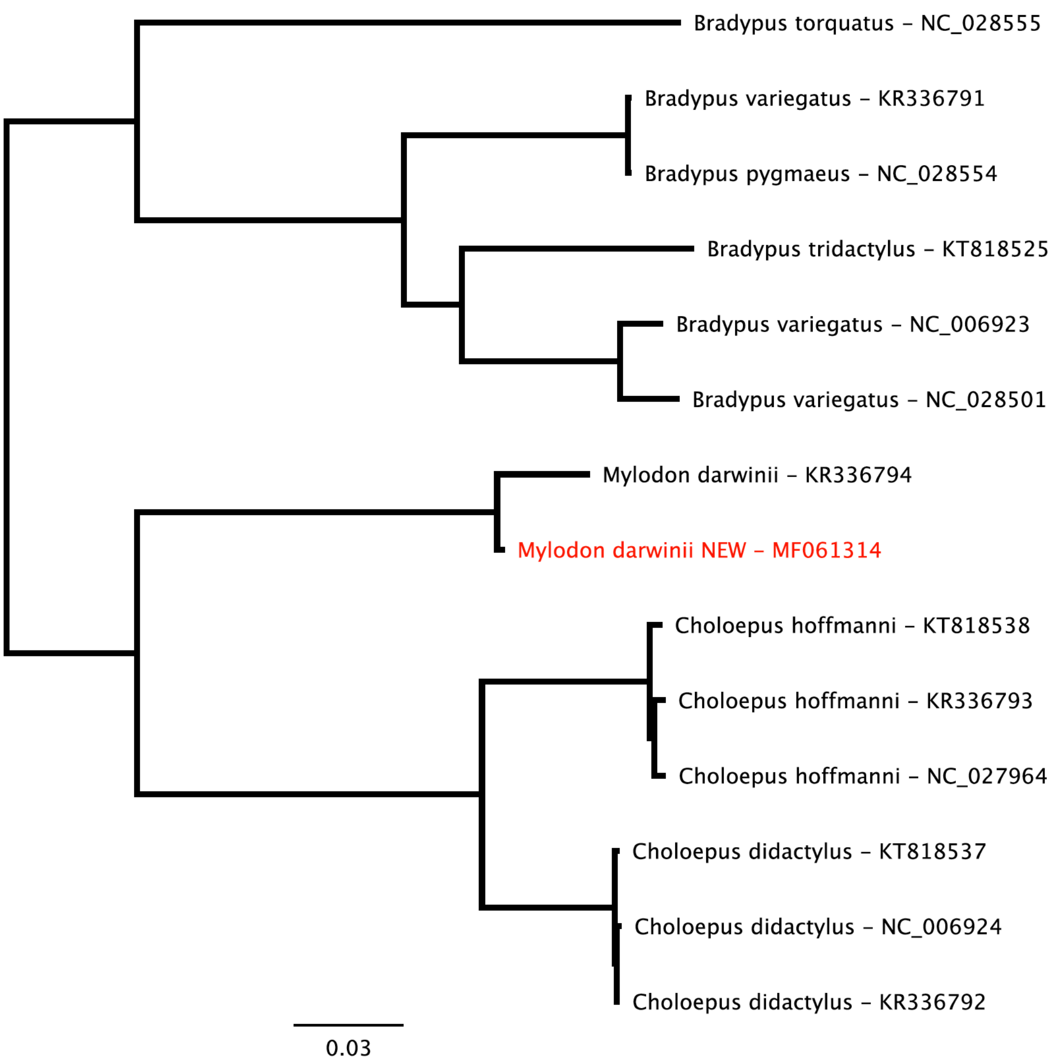

Figure S12: Maximum likelihood tree of available sloth mitogenomes obtained with RAxML under a single GTR+G model. Our new Mylodon mitogenome sequence is indicated in red to illustrate its much shorter branch length relative to the one previously obtained by Slater et al. (2017). Branch lengths represent mean number of substitutions per site.
